# Global, regional, and national burden of neurological disorders in 204 countries and territories worldwide

**DOI:** 10.7189/jogh.13.04160

**Published:** 2023-11-29

**Authors:** Yi Huang, Yongan Li, Haiyan Pan, Liyuan Han

**Affiliations:** 1 Department of Neurosurgery, The First Affiliated Hospital of Ningbo University, Ningbo, Zhejiang, China; 2 Department of Neurology, Suzhou Xiangcheng People's Hospital, Jiangsu, China; 3 The First Dongguan Affiliated Hospital，Guangdong Medical University; 4 School of Public Health, Guangdong Medical University, Dongguan, Guangdong, PR China; 5 Center for Cardiovascular and Cerebrovascular Epidemiology and Translational Medicine, Ningbo Institute of Life and Health Industry, University of Chinese Academy of Sciences, Ningbo, China

## Abstract

**Background:**

We aimed to determine the incidence and disability-adjusted life-years (DALYs) of neurological disorders worldwide from 1990 to 2019.

**Methods:**

We obtained age-standardised incidence and DALY rates of neurological disorders in 204 countries and territories from 1990 to 2019 from the Global Burden of Disease (GBD) database. We determined trends stratified by age, sex, region, country, and Social Development Index (SDI) and the risk factors contributing to DALYs associated with these neurological disorders.

**Results:**

The largest increases in the age-standardised incidence rates of neurological disorders in 1990-2019 occurred in four regions (East Asia: estimated annual percentage change (EAPC) = 0.19, tropical Latin America: EAPC = 0.07, Southern Latin America: EAPC = 0.03, Western Europe: EAPC = 0.03) and three countries (China: EAPC = 0.20, Ecuador: EAPC = 0.13, Italy: EAPC = 0.13). We observed the largest increases in age-standardised incidence rates for Parkinson disease, idiopathic epilepsy, and bipolar disorder, and in age-standardised DALY rates for Alzheimer disease and other dementias. High-SDI regions showed the highest EAPC for age-standardised incidence rates of Parkinson disease, depression, and motor neuron disease, and age-standardised DALY rates of neurological disorders.

**Conclusions:**

There is a need to control the increase in age-standardised incidence rates of neurological disorders in East Asia, tropical Latin America, Southern Latin America, and Western Europe, particularly in China, Ecuador, and Italy.

Neurological disorders, the most common of which are cerebrovascular, neurodegenerative, autoimmune, and spinal cord diseases, intracranial tumors, and craniocerebral trauma, cause significant damage to cognitive functioning, resulting in one of the highest rates of morbidity, disability, and mortality globally [1-6]. Their epidemiological patterns have changed considerably over time, primarily due to population growth and ageing, urbanisation, and extension of life expectancy, raising concerns about increases in the number of neurological disorder cases and the related global burden of disease [7-9]. In 2019 alone, neurological disorders were responsible for nearly 10 million deaths and 349 DALYs lost globally [10,11]. Meanwhile, stroke caused the most loss in DALYs of all diseases, while among neurologic disorders, migraine, meningitis, Alzheimer disease, autoimmune disorders, Parkinson disease, and other types of dementia and epilepsy all caused more than 10 million DALYs each [12-14]. Global, regional, and national data has shown that the burden of all non-communicable neurological diseases, the absolute number of DALYs, or persons who died due to non-communicable neurological diseases has increased significantly in all countries worldwide [15-18]. Despite the recent rapid development of various surgical, pharmacologic and interventional interventions, most patients with neurological disorders are clinically diagnosed only when nerve damage is very severe, losing the optimal treatment time. The prognosis of patients is still poor overall, while most drugs may have adverse side effects.

Reducing the burden of neurological disorders is integral to the United Nations’ third Sustainable Development Goal to cut down premature mortality from chronic non-communicable diseases by one-third by 2030 [19,20]. Due to the continued ageing and extensions in life expectancy, neurological disorder diagnoses are expected to increase significantly in the coming decades. However, studies have not yet estimated the extent of this increase based on data from the Global Burden of Disease (GBD) study.

To address this gap, we used the latest GBD 2019 data set to analyse the incidence and DALYs of neurological disorders stratified by age, sex, region, country, and Social Development Index (SDI), hoping that our findings could inform decision-making and efforts in preventing, treating, and managing neurological disorders worldwide.

## METHODS

### Overview

We obtained data for 204 countries and territories worldwide from the GBD database, covering the global burden of 369 diseases and injuries and their risk factors. The GBD regularly publish refined data on the incidence, mortality, and DALYs of various diseases stratified by age, sex, region, country, and SDI [13]. We searched its database via the Global Health Data Exchange to collect age-standardised incidence and age-standardised DALY rates (and the corresponding 95% uncertainty intervals (UIs)) for neurological disorders. As this data are publicly available, we did not seek ethical approval. Patients or the public were not involved in the design, or conduct, or reporting, or dissemination plans of our research. We conducted this study per the Guidelines for Accurate and Transparent Health Estimates Reporting [21].

The SDI is a composite indicator that measures the level of a country’s development based on a combination of lag distributed income (LDI) per capita, average educational attainment of people over 15 years of age, and total fertility rate [22]. The possible values of these three indicators range from 0 to 1, where 0 represents the lowest LDI, the lowest average educational attainment, and the highest total fertility rate, respectively. We grouped countries into low-, low-middle, middle, high-middle, and high-SDI categories.

### Estimation framework

We used the DisMod-MR 2.1 Bayesian meta-regression modeling tool to measure the incidence and DALYs, estimated as the sum of years lived with disability and the years of life lost, and their respective 95% UIs [13].

The GBD 2019 calculated the attributable mortality and DALYs of 87 risk factors and their combinations at the global and regional levels [23]. We employed a comparative risk-assessment framework to estimate the proportion of DALYs caused by three known risk factors, organised into a four-level hierarchy. Level 1 of this hierarchy comprises three categories of risk factors (behavioural, environmental and occupational factors, and metabolic factors) distributed into 20 level-2 risk factors [23].

### Case definitions of neurological disorders

We based the case definitions of disorders in the neurological disorders category in the GBD on the International Classification of Diseases (ICD), 9^th^ and 10^th^ edition codes (Table S1 in the **Online Supplementary Document**) [22].



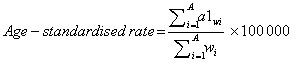



where a1 is the age-specific rate for the age group, *w_i_* is the number of people in the corresponding *i*^th^ age subgroup in the selected reference standard population, and A is the number of age groups.

Age standardisation aims to eliminate the impact of population age composition and ensure comparability of research indicators. The age-standardised rate in the GBD database is estimated using the world-population age standard [24]. Direct standardisation results in standardised or age-adjusted rates are weighted averages of specific age rates for each population being compared. The weight (standard) is used to represent the relative age distribution of an external population and serves as a summary rate for each population. This rate reflects the number of events that can be expected to occur if the populations being compared have the same age distribution.

EAPC is a widely accepted quantitative indicator used for estimating the annual average change in age-standardised rate within a given period. We performed fitting based on the natural logarithm of time variables and their corresponding observations, thereby ensuring that each observation contributed to the calculation of EAPCs, which estimates and quantifies the long-term trend in disease burden indicators, such as the incidence rate and mortality of diseases.

We established a regression model to describe the relationship between the natural logarithm (ln) of age-standardised rate and time:

y = b0 + βx + c, y = In (ASIR)

where x is the calendar year, b0 is the constant term, c is the false term, and β is the meaning of the negative or positive tendency of the selected age-standardised rate. We calculated EAPC using the following formula: EAPC = 100 × (exp [β] − 1) and obtained its 95% confidence interval (CI) from the linear regression model.

An age-standardised rate is regarded to be statistically increasing if both EAPC and its lower 95% CI limit are greater than 0, and as statistically decreasing if both values are lower. Otherwise, the change in age-standardised rate is regarded as stable (not significant).

We also compared the estimated incidences and DALYs of neurological disorders with those that were predicted based on SDIs. We performed all analyses using R, version 3.5.1 (R Core Team, Vienna, Austria).

## RESULTS

### Global level

**Figure 1:**
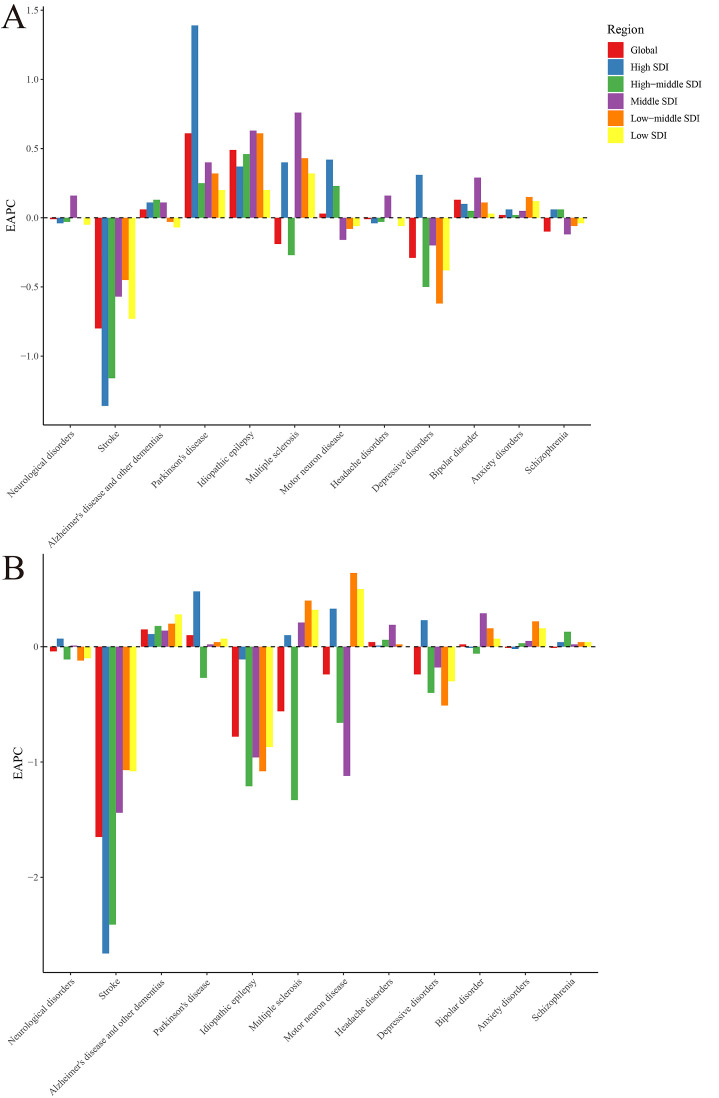
EAPC of neurological disorders and its main subtypes, with age-standardised rates from 1990 to 2019: **Panel A.** EAPC of age-standardised incidence rate. **Panel B.** EAPC of age-standardisbahed DALY rate. EAPC – estimated annual percentage change, DALY – disability-adjusted life-year.

In 2019, there were 805.17 million neurological disorder cases with an age-standardised incidence rate of 10 259.50 per 100 000 population (95% UI = 9223.20, 11 324.16). This rate remained stable on a global level from 1990 to 2019, corresponding to an EAPC of -0.01 (95% UI = -0.02 to 0.01), but was higher in women than in men in 2019, with a male-to-female ratio of 0.90. Regarding specific neurological disorders, we observed the largest increases in age-standardised incidence rates from 1990 to 2019 for Parkinson disease (EAPC = 0.61), idiopathic epilepsy (EAPC = 0.49), and bipolar disorder (EAPC = 0.13) (**Figure 1**, Panel A and Table S2 in the **Online Supplementary Document**).

Neurological disorders caused 97.72 million DALYs in 2019, with an age-standardised DALY rate of 1253.56 (95% UI = 719.70, 2039.81) per 100 000 population. The age-standardised rate on a global level was 1253.56 (95% UI = 719.70, 2039.81), corresponding to an EAPC decrease since 1990 of -0.04 (95% UI = -0.05, -0.04), and was higher in women than in men from 1990 to 2019, as demonstrated by the male-to-female ratio of 0.81. In terms of specific neurological disorders, we found largest increases in the same period for Alzheimer disease and other dementias (EAPC = 0.15), Parkinson disease (EAPC = 0.10), and headache disorders (EAPC = 0.04) (**Figure 1**, Panel B and Table S2 in the **Online Supplementary Document**).

### Regional level

**Figure 2:**
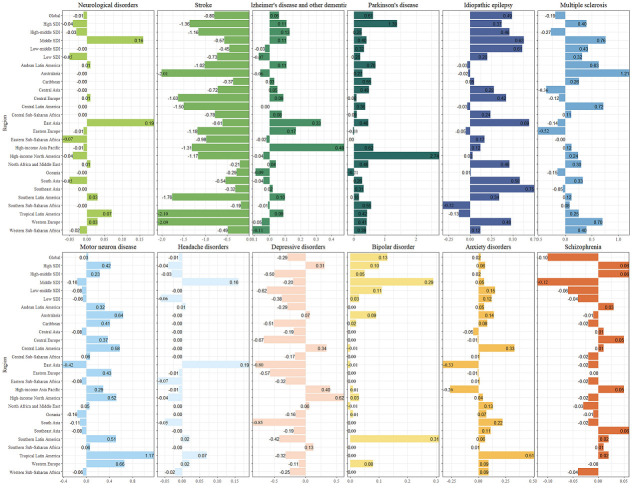
EAPC of neurological disorders and its main subtypes, with age-standardised incidence rate from 1990 to 2019 by region.

In 2019, at a regional level, North America (n = 13 183.68), Eastern Europe (n = 12 706.10), and Western Europe (n = 12 526.35) had the highest age-standardised incidence rates of neurological disorders. The rate increased the most in East Asia (EAPC = 0.19), tropical Latin America (EAPC = 0.07), and Southern Latin America and Western Europe (EAPC = 0.03) (**Figure 2**, Figures S1 and Table S3-S4 in the **Online Supplementary Document**).

**Figure 3:**
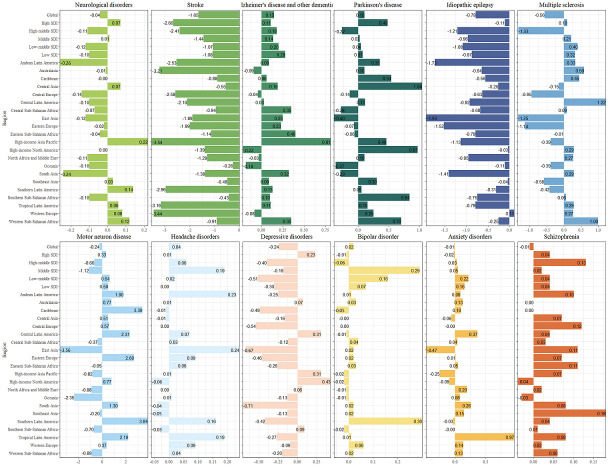
EAPC of neurological disorders and its main subtypes, with age-standardised DALY rate from 1990 to 2019 by region. DALY – disability adjusted life-year.

Meanwhile, tropical Latin America (n = 1471.72), Western Europe (n = 1432.56), and Western sub-Saharan Africa (n = 1425.08) had the highest age-standardised DALY rates of neurological disorders in 2019, while high-income Asia-Pacific (EAPC = 0.22), Southern Latin America (EAPC = 0.14), and Western sub-Saharan Africa (EAPC = 0.12) saw the highest increases in 1990-2019 (**Figure 3**, Figure S2 and Tables S4-6 in the **Online Supplementary Document**).

### National level

In 2019, the age-standardised incidence rates of neurological disorders ranged from 7295.60 in Ethiopia to 13 540.71 in Italy, with highest rates observed in Italy (n = 13 540.71), Norway (13 508.33), United States of America (n = 13 278.76). The largest increases from 1990 to 2019 occurred in China (EAPC = 0.20), Ecuador (EAPC = 0.13), and Italy (EAPC = 0.13) (Figure S3 and Tables S7-S8 in the **Online Supplementary Document**).

Meanwhile, the age-standardised DALY rates in 2019 ranged from 916.57 in Singapore to 1613.40 in Kiribati, and were the highest in Kiribati (n = 1613.40), Belgium (n = 1578.75), and Tajikistan (n = 1543.39) (Table S8 in the Online Supplementary Document). From 1990 to 2019, the EAPC in the age-standardised DALY rates of neurological disorders differed considerably between countries, with the largest increases occurring in Lesotho (EAPC = 0.57), Japan (EAPC = 0.40), and Equatorial Guinea (EAPC = 0.38) (Figure S4 and Table S8 in the **Online Supplementary Document**).

Moreover, the age-standardised incidence rate of Parkinson disease significantly increased in 159 countries from 1990 to 2019, with the greatest increase occurring in the USA (EAPC = 2.87), as did its DALY rates in 122 countries and territories from 1990 to 2019, with the greatest increase occurring in Tajikistan (EAPC = 2.65) (Tables S9-S10 in the **Online Supplementary Document**).

### Age- and sex-specific patterns

**Figure 4:**
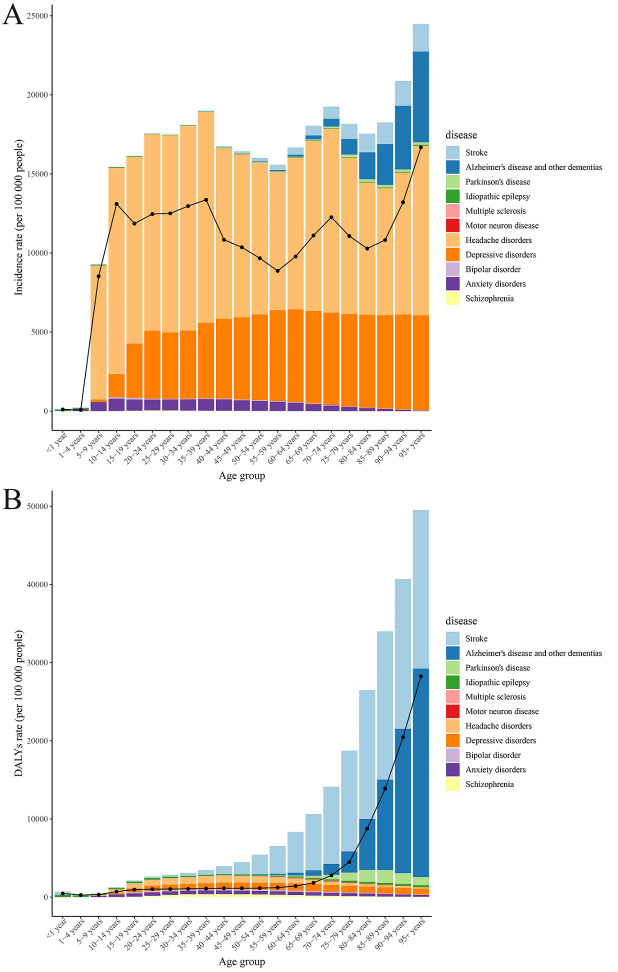
The incidence and DALY rates of neurological disorders and its main subtypes among different age groups.

In 2019, the incidence rate of neurological disorders was highest in both men and women aged 70-74 years. The global DALY rate was higher in women than in men and increased with age, while the number of DALY cases was highest in 80-84-year-old women and men. The population with headache and depressive disorders had the highest incidence, while the population with stroke, Alzheimer disease and other dementias had the highest DALY rate (**Figure 4**, Panels A-B and Figure S5, Panels A-B in the **Online Supplementary Document**).

### Burden of the neurological disorders by SDI

The EAPCs in the age-standardised incidence rates of neurological disorders from 1990 to 2019 were highest in middle-SDI regions (EAPC = 0.42).

Regarding specific diseases, the rates of Alzheimer disease and other dementias (EAPC = 0.13) were the highest in high-middle SDI regions, while those of Parkinson disease (EAPC = 1.39), depressive disorders (EAPC = 0.31), and motor neuron disease (EAPC = 0.42) were the highest in high SDI regions (**Figure 1**, Panel A and Table S11 in the **Online Supplementary Document**).

However, the EAPCs in the age-standardised DALY rates of neurological disorders (EAPC = 0.07), and specifically in Parkinson disease (EAPC = 0.48) and depressive disorders (EAPC = 0.23), were highest in high-SDI regions. Conversely, the related EAPCs of Alzheimer disease and other dementias were highest in low-SDI regions (EAPC = 0.28) (**Figure 1**, Panel B and Table S12 in the **Online Supplementary Document**).

From 1990 to 2019, high-income Asia-Pacific and North America, tropical Latin America, Central Asia, and Central Europe had higher than expected age-standardised incidence rates of neurological disorders, while Western sub-Saharan Africa, Western Europe, North Africa and the Middle East, tropical Latin America, and Central Asia had higher than expected DALY rates. Countries such as Italy, Norway, Sweden, and the USA had a much higher incidence rate of neurological disorders than expected, and countries such as Kiribati, Belgium, Tajikistan, and Germany had a much higher DALY rate (Figures S6-S7 in the **Online Supplementary Document**).

### Risk factors contributing to neurological disorder-associated DALYs

**Figure 5:**
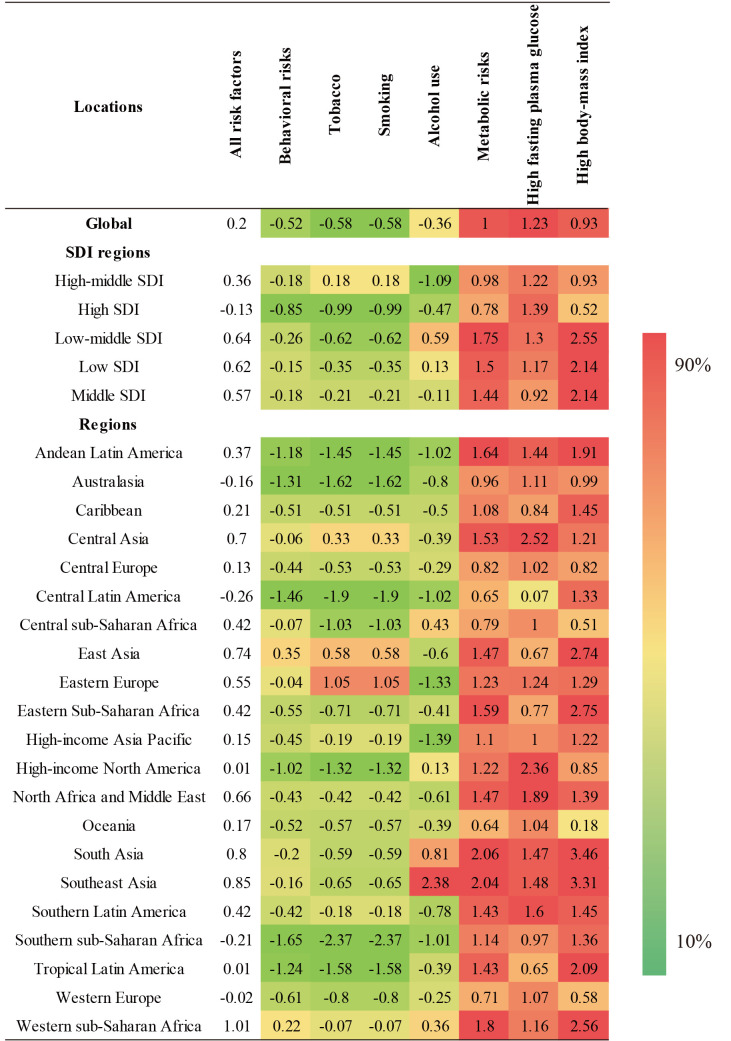
The ranking of risk factors for age-standardised DALY rate attributable to neurological disorders according to region.

Globally, behavioural risks and metabolic risk-factor clusters were the main factors related to the burden of neurological disorders, as they contributed to 50.95% and 57.76% of DALYs, respectively. From 1990 to 2019, the disease burden of neurological disorders attributable to all of the risk factors trended upwards globally and in all except in the high-SDI quintile. In the same period and in all of the SDI quintiles and in 21 regions, metabolic risks – mainly high fasting plasma glucose and high body-mass index – were the leading increased risk factors for the development of neurological disorders. Likewise, from 1990 to 2019, the age-standardised DALY rate of neurological disorders attributable to behavioural risk factors (including tobacco and smoking) trended downwards globally and in all but the high-middle SDI quintile (**Figure 5** and Table S13 in the **Online Supplementary Document**). Meanwhile, the age-standardised DALY rate of neurological disorders attributable to alcohol use trended upward in low-middle-SDI and low-SDI regions.

We observed the largest increase in the age-standardised DALY rate for neurological disorders attributable to metabolic risk factors (EAPC = 4.56) and high body-mass index in Equatorial Guinea (EAPC = 8.16), and largest increase in this rate attributable to high fasting plasma glucose occurred in Luxembourg (EAPC = 3.78) (Figure S8 in the **Online Supplementary Document**).

## DISCUSSION

Our findings show that the global age-standardised incidence rate of neurological disorders remained stable during the study period, while the age-standardised DALY rate decreased slightly and the numbers of DALY and incident cases increased overall. In terms of specific neurological disorders, we observed the largest increases in age-standardised incidence rates for Parkinson disease, idiopathic epilepsy, and bipolar disorder, and the largest increases in age-standardised DALY rates for Alzheimer disease and other dementias, Parkinson disease, and headache disorders. Globally, from 1990 to 2019, in all of the SDI quintiles and in 21 regions, metabolic risks – mainly high fasting plasma glucose and high body-mass index – were the leading increased risk factors associated with the neurological disorders that exhibited increased DALY rates.

The current global increase in the incidence of these neurological disorders may be related to population growth, population ageing, increased life expectancy, and increased exposure to associated risk factors [25]. For example, from 1990 to 2019, the age-standardised incidence rate increased the most in East Asia, tropical Latin America, and Southern Latin America. This suggests that with socioeconomic development, the pattern of disease burdens in the above-mentioned regions is gradually shifting to a predominantly chronic non-communicable disease pattern, leading to a continuous increase in the incidence of chronic diseases. The increase may also be related to environmental pollution [26], lifestyle changes [27], and advances in diagnostic methods in those regions [28]. Metabolic risk factors and poor lifestyles (eg, smoking and a lack of exercise) are now prevalent in these regions, concurrent with a significant increase in the incidence of obesity, diabetes, and hypertension [29]. All of the above findings indicate that in these regions, the strategies used for the primary prevention of neurological disorders are not sufficiently effective and should thus be strengthened.

The slight decrease in the global age-standardised DALY rate of neurological disorders may be related to global economic and social development, improved health care, and improved treatment technology. However, the rate increased the most in regions such as high-income Asia-Pacific, Southern Latin America, and Western sub-Saharan Africa, and countries such as Lesotho, Japan, and Equatorial Guinea. Considering the correlation between neurological disorders and age, the high ranking of Japan’s age-standardised DALY rate can be attributed to the longer life expectancy in Japan compared to many other countries [30]. The increase in the age-standardised DALY rates in Lesotho and Equatorial Guinea suggests that low-income countries should increase their attention to the improvement of medical care to prevent and treat these neurological disorders.

Parkinson disease contributed the most to the global age-standardised incidence of neurological disorders and had the second-largest contribution to the global age-standardised DALYs, especially in high-SDI regions where it is growing the most rapidly. Compared with low-SDI regions, people in high-SDI regions are more aware of their health and may look for medical help earlier. Moreover, SDI can be used as an indicator of educational attainment. Higher levels of education are associated with a higher risk of developing Parkinson disease [31]. However, Parkinson disease has no obvious clinical symptoms in its early stages, its differential diagnosis takes a long time, and it is prone to neglect, misdiagnosis, and missed diagnosis. We found the age-standardised incidence rate of Parkinson disease in some developing countries to be relatively low. However, as its occurrence is significantly associated with increasing age, and as people in developing countries have relatively limited access to health care services for older adults, it is possible we underestimated the disease’s incidence [32]. Moreover, the incidence of idiopathic epilepsy and bipolar disorder is growing rapidly worldwide, as are the DALYs of Alzheimer disease and other dementias, suggesting an urgent need for research to identify effective methods for the prevention and treatment of these neurological disorders.

The EAPCs of the age-standardised DALY rates of neurological disorders were highest in high-SDI regions. This may be related to the higher level of ageing in high-income than in low-income regions [33]. Older adults are susceptible to these disorders [34], and the rapid ageing process in the societies of high-SDI regions has led to them comprising a progressively larger proportion of the population in these areas. Likewise, the increased DALYs of Parkinson disease in high-income countries may be associated with improved treatment options that prolong the duration of the disease [35]. Moreover, the burden of these neurological disorders will increase further if the ageing of the populations in countries and regions grows with future economic and social development.

Notably, high-income countries had a higher age-standardised incidence rate of neurological disorders in 2019, with the highest rates occurring in Italy and Norway. In these countries, people are more informed about their health and may seek medical help earlier than people in low-SDI countries such as China, which had the largest increases in age-standardised incidence rates. This may be because China improved its medical care in recent years, while conducting cerebrovascular disease-screening programs and thus strengthening their early detection and intervention [36]. The country0s medical standards have also improved and a regional network of cerebrovascular disease-screening and prevention-focused hospitals, a national stroke center, and a green channel for neurological disorder emergency care have been founded, increasing the detection of cerebrovascular disease [37]. Moreover, the Chinese government went on to expand primary care coverage to improve the accessibility, availability, and affordability of health care, thus ensuring that an increased number of patients with potential neurological disorders are identified and given appropriate interventions [38].

The incidence rate of neurological disorders in 2019 peaked in both men and women aged 70-74 years, suggesting that age is an important factor in their development. Thus, the quality and intensity of screening and intervention programs for groups at high risk of developing these neurological disorders (especially those aged ≥70 years) should be improved. In view of incidence for all age groups, we should focus on patients with headache and depression disorders, while regarding DALY rates of all ages, more attention should be paid to the treatment and improvement of stroke and Alzheimer disease and other dementias.

We also found gender differences in the disease burden of neurological disorders, as the incidence and DALY rates in women were higher than those in men. This may be related to several factors, such as women’s higher estrogen concentrations, longer life expectancy, lower education and socioeconomic status compared to men [39], and the fact that women constitute the majority of the ageing population, which to some extent increases the risk of developing neurological disorders. Therefore, interventions should be provided in a gender-specific manner [40].

Our results also show that the disease burden of neurological disorders attributable to metabolic risk factors is rising rapidly, highlighting metabolic factors as an increasingly important cause. Global efforts should focus on controlling metabolic risk factors to prevent and treat these disorders in the future. Interventions for primary prevention should include reducing exposure to metabolic risk factors (such as by screening and appropriate management of blood glucose and weight), reducing behavioural risk factors (such as by providing abstinence programs), strengthening the monitoring of neurological disorder risk factors, and screening high-risk groups [41,42].

We used up-to-date data to provide a comprehensive assessment of the patterns and trends of the age-standardised incidence and DALYs of neurological disorders in 204 countries and territories at the global, regional, and national levels. Our findings could be used to explore the aetiology of these disorders in local areas and develop early-prevention strategies to reduce their incidence and the associated DALYs. We also discovered geographical differences in neurological disorders and their risk factors, suggesting that country-specific priorities and strategies, and those focused on specific disorders, should be developed and implemented to decrease their burden in different geographical locations.

This study also has some shortcomings. The inclusion of raw data from GBD 2019 may have introduced bias, as some countries may have incomplete or low-quality data. Furthermore, the internal economic development in a country or region may not be balanced and thus may not completely demonstrate the differences between countries or regions. Likewise, the diagnostic accuracy of neurological disorders potentially limits the estimation of GBD incidence and DALYs, as diagnostic opportunities differ between countries and might not allow for similar detection rates, and as diagnosis and classification criteria between countries and time periods might be different, further complicating the burden analysis. Also, most regional- and national-level data suggests problems in diagnostics and counting of cases across different countries; however, we observed no patterns that would explain why some countries show up as having high estimates and others in the same region are quite different from those. Finally, as the GBD study provides aggregate data, all those risk factors were applied in an ecological study design, so our findings require more validation through future research.

## CONCLUSIONS

From 1990 to 2019, the burden of neurological disorders significantly differed between countries and regions. However, due to the ageing of populations, the number of incident and DALY cases increased significantly. Due to current limitations in medical resources and health systems, this burden remains an important challenge globally and locally. Future efforts should target older adults and women, while improved strategies to control modifiable risk factors, including (among others) high fasting plasma glucose and high body-mass index, could be used to effectively decrease the associated DALYs. International organisations and governments should strive to narrow the gap between countries at different levels of development, according to their most prevalent neurological disorders and their associated risk factors.

## Additional material


Online Supplementary Document
