# Supplementary material for: Global, regional, and national burden of neurological disorders in 204 countries and territories worldwide
Source: J Glob Health. 2023 Nov 29;13:04160. doi: 10.7189/jogh.13.04160 (PMC10685084; doi:10.7189/jogh.13.04160)
Supplement: Online Supplementary Document [file jogh-13-04160-s001.pdf]

Supplementary figure

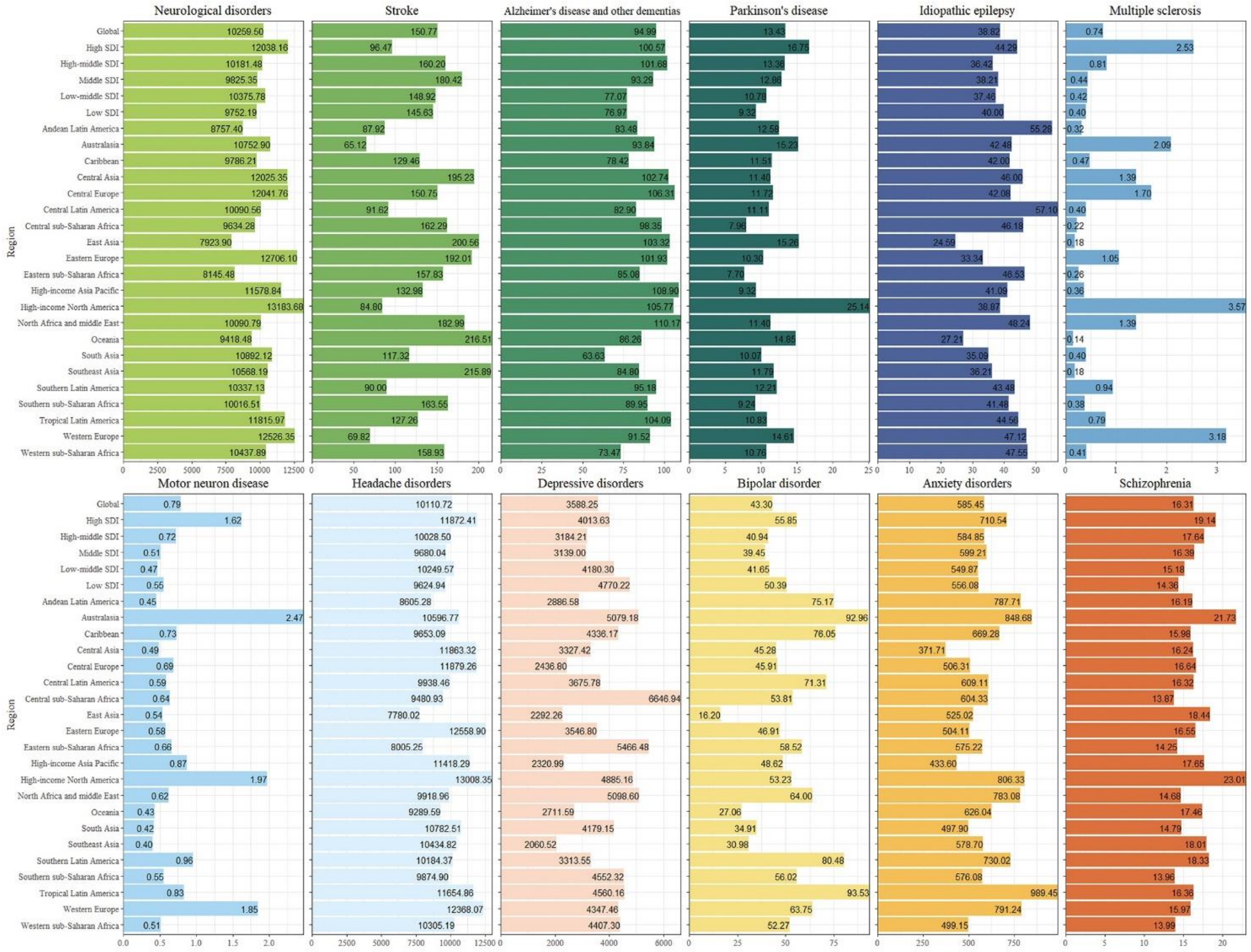

Figure S1. The age-standardized incidence rate of neurological disorders and its main subtypes in 2019 by region.

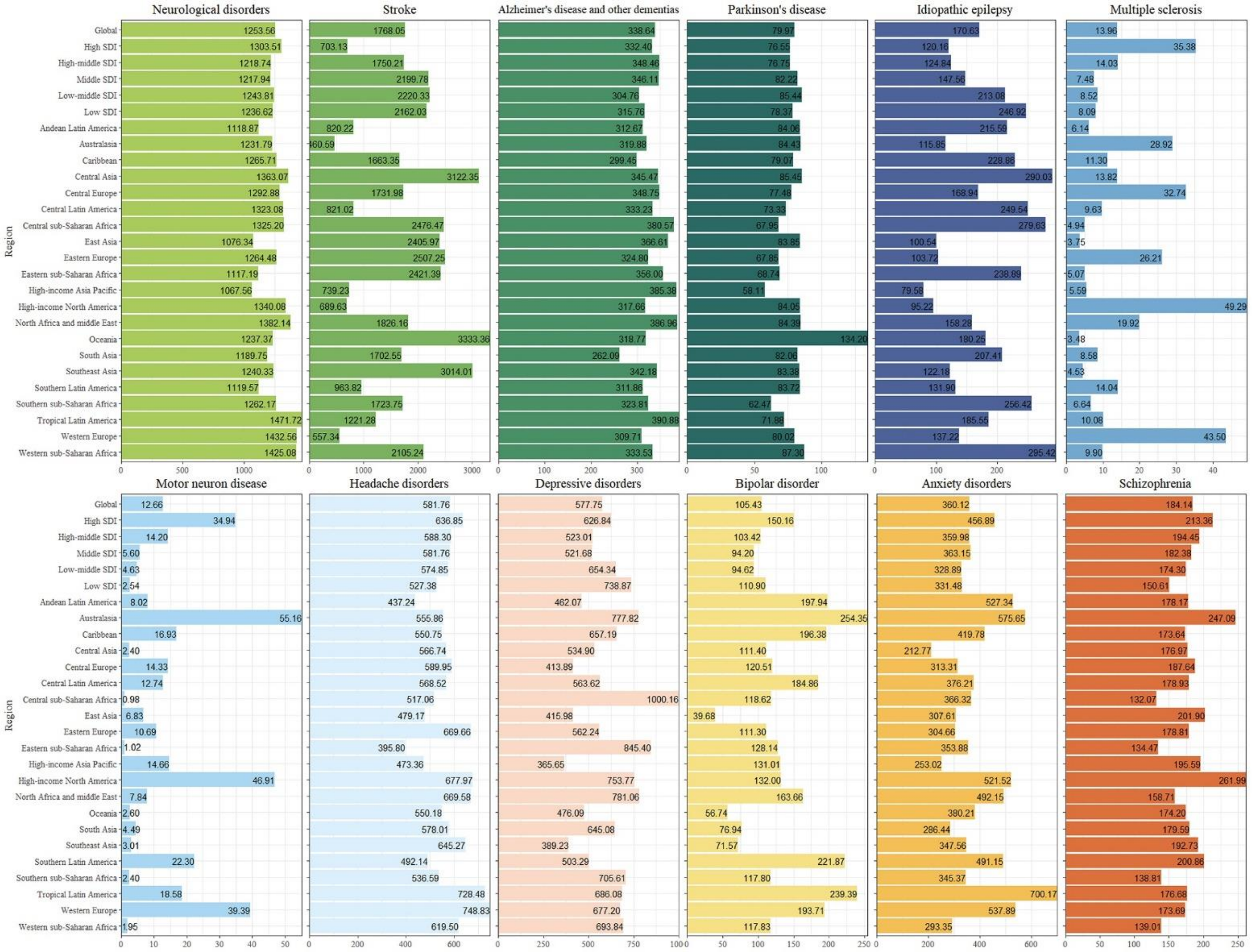

Figure S2. The age-standardized DALY rate of neurological disorders and its main subtypes in 2019 by region.

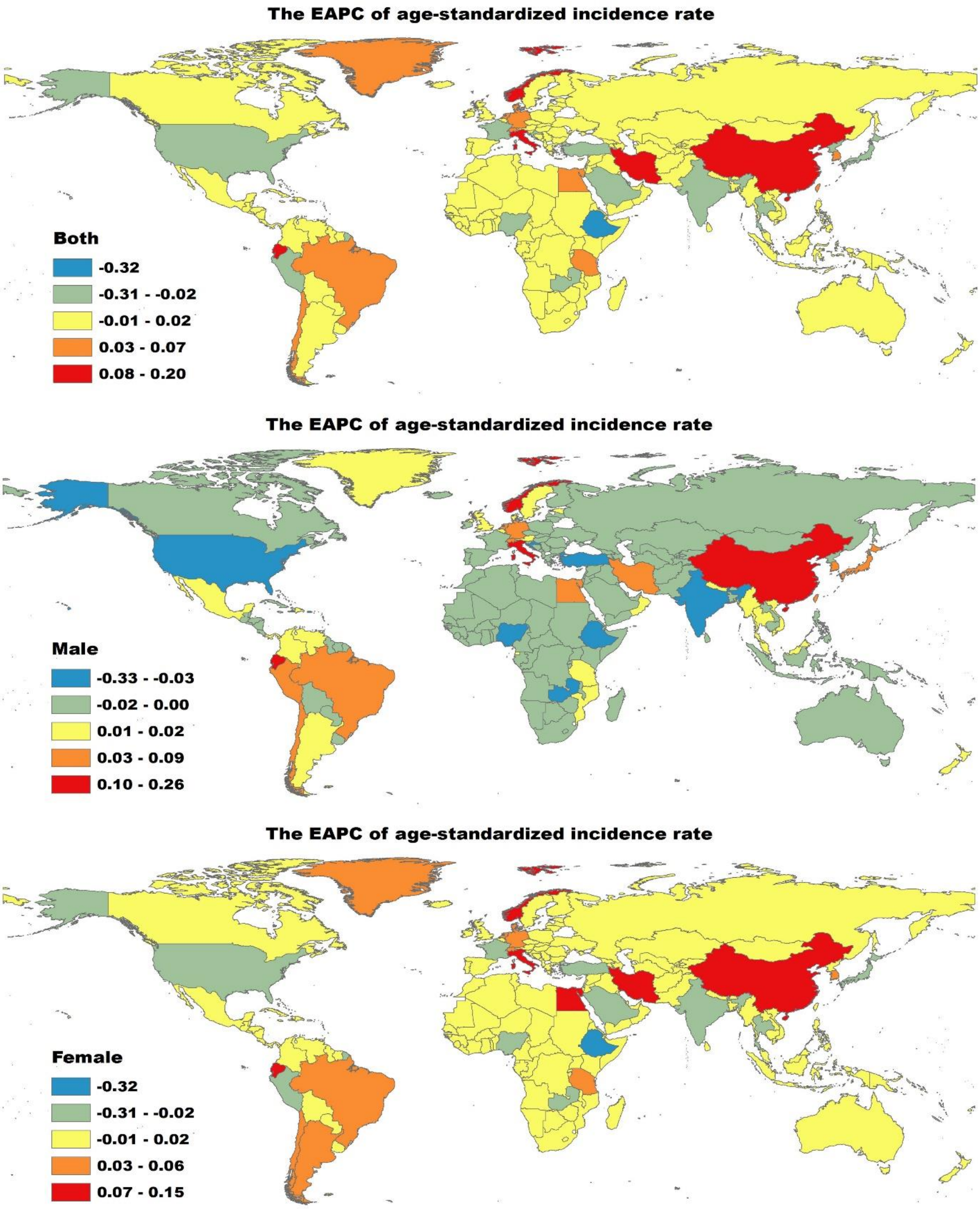

Figure S3. The global map of neurological disorders EAPC of age-standardized incidence rate. EAPC = estimated annual percentage change.

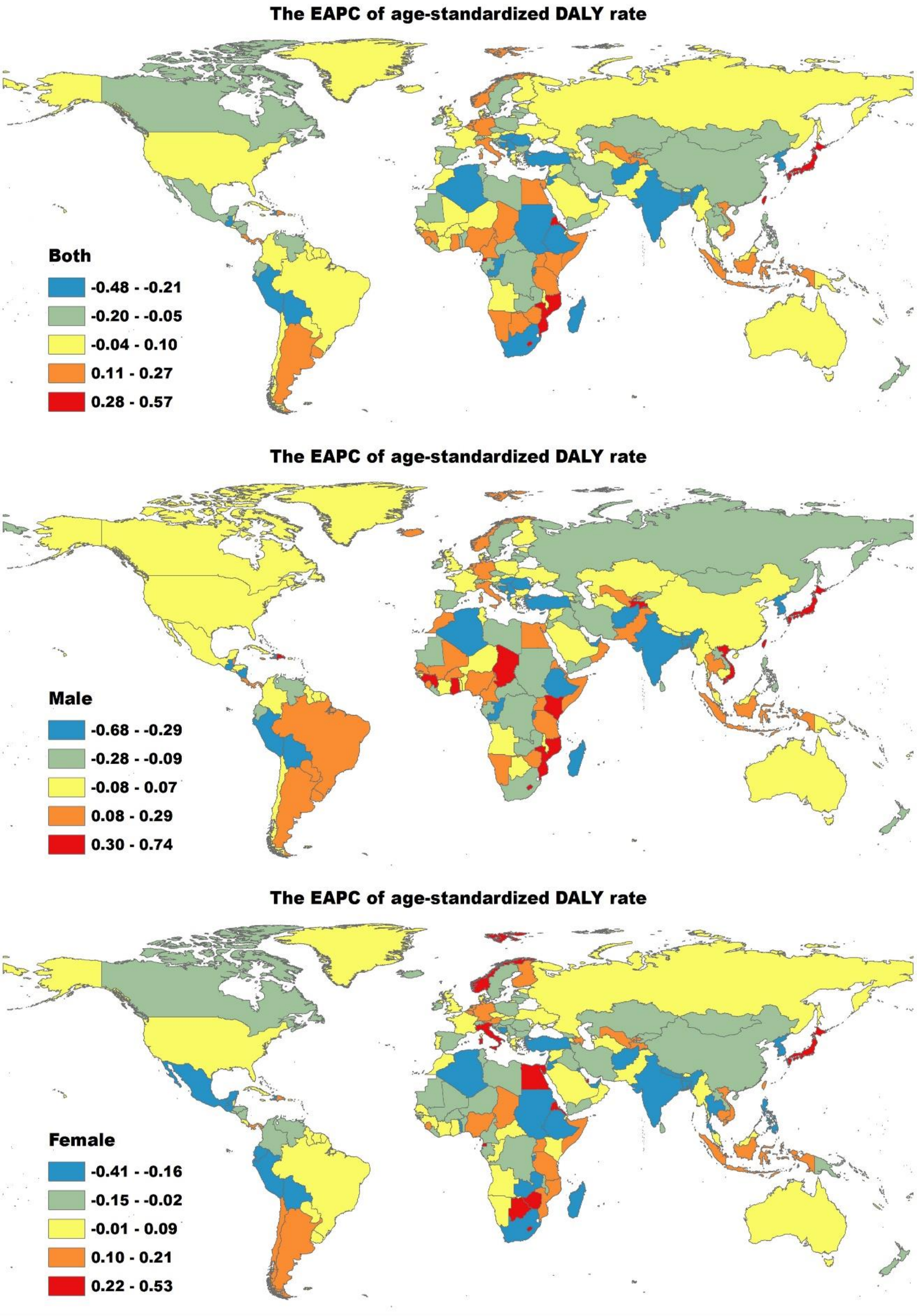

Figure S4. The global map of neurological disorders EAPC of age-standardized DALY rate. EAPC = estimated annual percentage change; DALY = disability adjusted life-year.

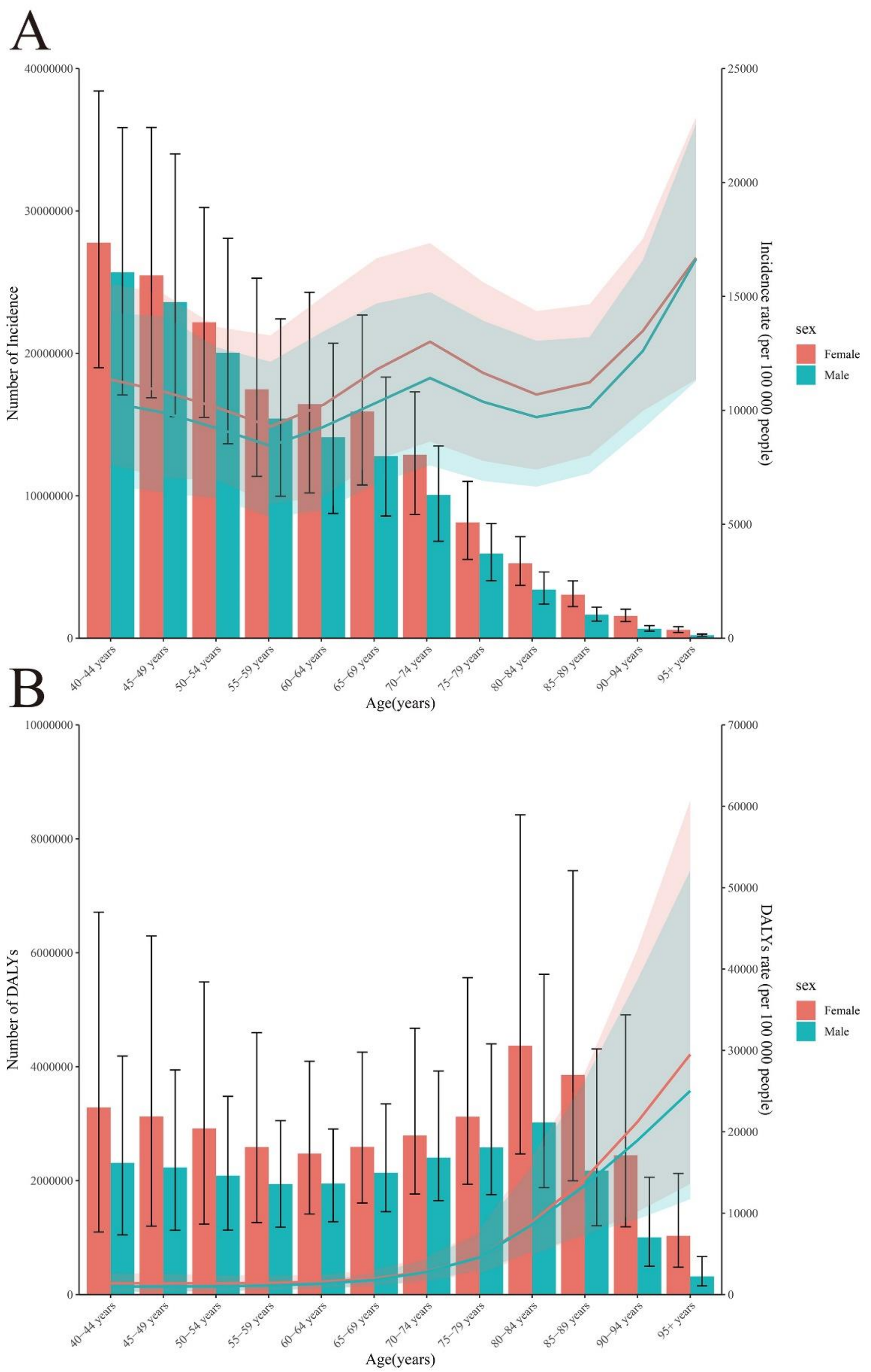

Figure S5. Global number of incidences, DALY cases, incident and DALY estimates of neurological disorders per 100 000 population by age and sex, 2019. Dotted and dashed lines indicate 95% upper and lower uncertainty intervals, respectively: **Panel A.** incident cases, and incident estimates. **Panel B.** DALY cases, and DALY estimates. DALY = disability adjusted life-year.

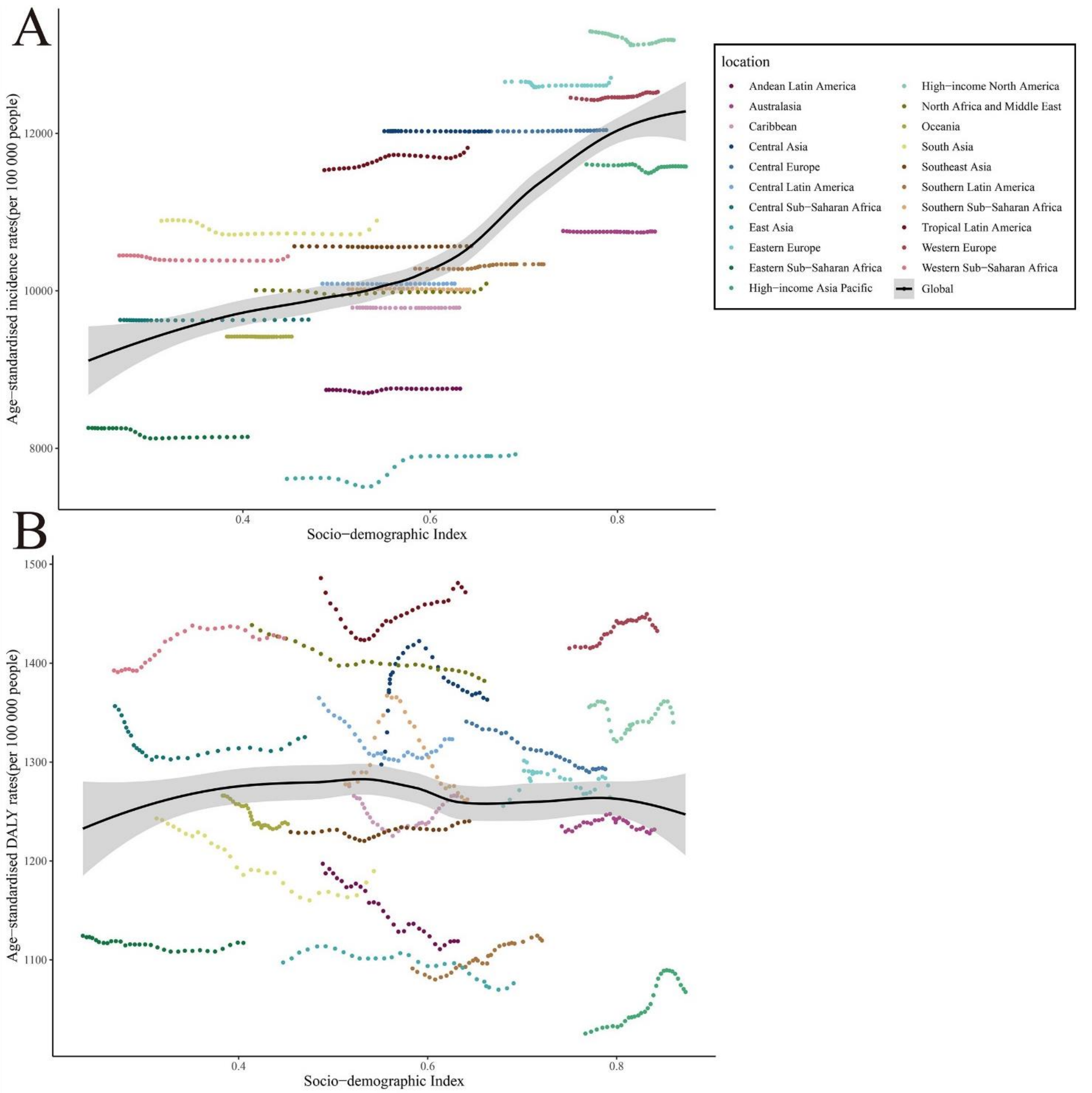

Figure S6. The age-standardized rates for neurological disorders for 21 GBD regions by Sociodemographic Index, 1990–2019: **Panel A.** The age-standardized incidence rate. **Panel B.** The age-standardized DALY rate. DALY = disability adjusted life-year.

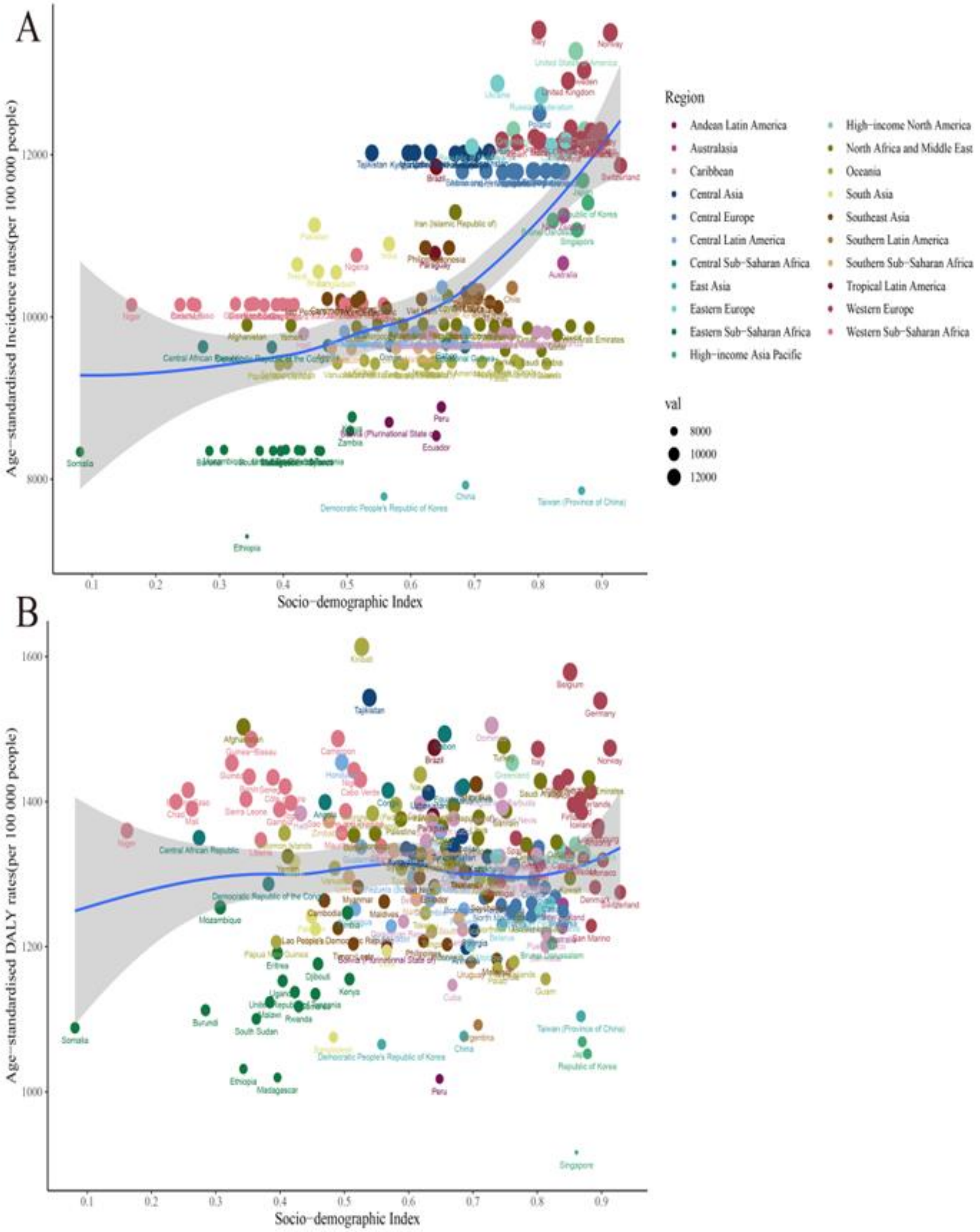

Figure S7. The age-standardized rates for neurological disorders according to country-specific Sociodemographic Index: **Panel A.** The age-standardized incidence rate. **Panel B.** The age-standardized DALY rate. DALY = disability adjusted life-year.

Locations	Tobacco	Smoking	Alcohol use	Metabolic risks	High fasting plasma glucose	High body-mass index
**Countries**						
Afghanistan	0.64	0.64	17.14	1.89	1.74	2.41
Albania	0.23	0.23	2.12	0.97	0.98	1.04
Algeria	-0.65	-0.65	1.35	1.57	1.64	1.74
American Samoa	-0.29	-0.29	0.9	0.34	0.81	-0.03
Andorra	-0.74	-0.74	-1.05	0.66	1.47	0.38
Angola	-0.09	-0.09	3.23	2.03	1.23	3.97
Antigua and Barbuda	0.34	0.34	0.64	1.26	1.07	1.68
Argentina	-0.11	-0.11	-1	1.34	1.25	1.5
Armenia	0.4	0.4	-0.8	2.33	2.09	2.63
Australia	-1.5	-1.5	-0.66	0.95	1.03	1.02
Austria	0.97	0.97	-0.95	0.99	1.83	0.69
Azerbaijan	1.49	1.49	-0.65	2.03	2.58	1.92
Bahamas	-0.39	-0.39	-2.19	0.76	0.89	0.76
Bahrain	-0.45	-0.45	-4.17	0.97	1.42	0.8
Bangladesh	-1.16	-1.16	2.99	1.47	0.87	3.68
Barbados	-0.19	-0.19	-0.79	0.66	0.8	0.63
Belarus	-0.29	-0.29	-0.69	1.09	0.53	1.31
Belgium	-0.79	-0.79	0.25	0.52	0.87	0.41
Belize	-0.56	-0.56	0.37	1.63	1.36	1.98
Benin	-1.52	-1.52	0.71	1.62	1.15	2.33
Bermuda	0.29	0.29	-2.26	0.7	0.84	0.71
Bhutan	0.26	0.26	-5.01	2.62	2.12	3.51
Bolivia (Plurinational State of)	-1.8	-1.8	-1.29	1.84	1.22	2.53
Bosnia and Herzegovina	0.81	0.81	-0.04	1.73	1.92	1.81
Botswana	-0.8	-0.8	-0.32	2.4	1.68	3.28
Brazil	-1.59	-1.59	-0.37	1.42	0.63	2.1
Brunei Darussalam	-1.01	-1.01	-5.97	0.94	0.52	2.42
Bulgaria	-0.65	-0.65	0.06	0.23	0.94	-0.08
Burkina Faso	-1.67	-1.67	0.53	1.68	0.76	3.22
Burundi	-1.94	-1.94	-2.89	1.17	0.89	1.76
Cabo Verde	-1.97	-1.97	0.53	2.38	2.04	2.96
Cambodia	0.58	0.58	4.67	3.42	3.32	3.92
Cameroon	-0.71	-0.71	0.89	1.09	1.15	1.17
Canada	-1.71	-1.71	-0.65	0.89	1	0.93
Central African Republic	-1.38	-1.38	-1.46	1.13	1.01	1.52
Chad	-0.34	-0.34	3.48	1.53	1.2	2.4
Chile	-0.26	-0.26	-0.73	1.41	1.94	1.24
China	0.6	0.6	-0.6	1.48	0.64	2.81
Colombia	-1.82	-1.82	-1.16	0.76	-0.04	1.68
Comoros	-0.23	-0.23	1.57	1.27	0.83	1.73
Congo	0.27	0.27	-0.25	1.8	1.33	2.44
Cook Islands	-0.4	-0.4	5	0.41	0.7	0.33
Costa Rica	1.16	1.16	1.11	0.76	0.38	1.2
Croatia	-0.85	-0.85	-0.99	1.12	0.91	1.35
Cuba	-0.67	-0.67	-0.82	1.01	0.56	1.57
Cyprus	-0.54	-0.54	-1.26	-0.09	-0.7	0.77
Czechia	-0.38	-0.38	-0.65	1.2	1.55	1.14
Côte d'Ivoire	0.38	0.38	0.53	1.16	1.27	1.14
Democratic People's Republic of Korea	0.2	0.2	-1.17	0.71	1.08	0.06
Democratic Republic of the Congo	-1.79	-1.79	-1.19	0.31	0.91	-0.65
Denmark	-0.98	-0.98	-0.16	1.07	1.88	0.79
Djibouti	0.07	0.07	-4.12	2.14	1.02	4.13
Dominica	-0.83	-0.83	-0.77	1.07	1.11	1.21
Dominican Republic	0.21	0.21	0.77	2.06	1.91	2.31
Ecuador	-2.54	-2.54	-0.23	1.58	1.76	1.63
Egypt	1.09	1.09	0.99	1.41	2.41	1.1
El Salvador	-0.48	-0.48	-0.36	1.63	1.46	1.95
Equatorial Guinea	0.46	0.46	3.29	4.56	1.97	8.16
Eritrea	0.15	0.15	-0.79	2.56	1.62	4.02
Estonia	0.73	0.73	0.65	1.77	1.59	1.91
Eswatini	-1.39	-1.39	1.06	0.65	0.92	0.6
Ethiopia	-0.63	-0.63	-0.61	1.4	0.34	3.45
Fiji	-1.41	-1.41	0.6	0.67	0.88	0.63
Finland	-0.38	-0.38	-0.26	0.55	0.71	0.53
France	-0.36	-0.36	-0.86	1.18	1.73	1.05
Gabon	0.27	0.27	-1.18	1.86	1.01	2.85
Gambia	-1.16	-1.16	2.55	1.68	1.39	2.09
Georgia	0.64	0.64	0.78	0.95	2.49	0.36
Germany	-1.41	-1.41	0.92	0.02	0.18	-0.01
Ghana	-1.51	-1.51	0.4	2.92	1.64	4.58
Greece	0.05	0.05	-0.53	0.84	1.26	0.7
Greenland	-0.37	-0.37	-0.78	1.25	2.89	0.84
Grenada	-0.74	-0.74	-0.84	1.53	1.32	2.02
Guam	0.17	0.17	2.98	0.24	0.28	0.3
Guatemala	-1.15	-1.15	-1.48	1.66	1.4	2.29
Guinea	0.19	0.19	1.87	1.45	1.41	1.59
Guinea-Bissau	-1.26	-1.26	0.34	1.36	1.25	1.61
Guyana	-1.1	-1.1	-1	1.17	0.95	1.69
Haiti	-0.77	-0.77	-1.05	1.44	1.47	1.54
Honduras	-0.76	-0.76	-1.38	1.37	0.98	1.99
Hungary	-0.65	-0.65	-2.24	0.76	1.23	0.61
Iceland	-1.17	-1.17	1.71	0.79	1.67	0.49
India	-0.17	-0.17	0.71	2.25	1.58	3.85
Indonesia	0.5	0.5	-0.43	2.42	2.02	3.25
Iran (Islamic Republic of)	-0.36	-0.36	5.41	1.78	2.05	1.8
Iraq	-0.53	-0.53	-3.7	0.99	1.48	0.82
Ireland	-1.48	-1.48	-0.86	1.43	3.57	0.78
Israel	-0.92	-0.92	2.24	1.1	1.9	0.72
Italy	0.41	0.41	-1.29	1.62	2.1	1.39
Jamaica	0.17	0.17	0.67	1.45	1.44	1.65
Japan	-0.08	-0.08	-0.89	1.07	0.98	1.17
Jordan	-0.65	-0.65	0.46	0.88	0.6	1.2
Kazakhstan	0.03	0.03	-1.64	0.96	1.96	0.64
Kenya	-1.08	-1.08	-0.4	1.6	0.5	2.65
Kiribati	1.48	1.48	-1.4	1.14	1.76	0.41
Kuwait	-0.26	-0.26	16.24	1.01	0.89	1.28
Kyrgyzstan	0.57	0.57	-0.03	0.93	1.56	0.74
Lao People's Democratic Republic	-0.22	-0.22	0.08	2.13	1.73	3.5
Latvia	-0.3	-0.3	0.37	1.24	1.88	1.14
Lebanon	0.85	0.85	-2.46	1.19	1.45	1.2
Lesotho	0.13	0.13	2.49	1.62	1.16	2.12
Liberia	-0.86	-0.86	0.02	1.38	1.23	1.77
Libya	-1.05	-1.05	2.52	1.16	1.72	0.99
Lithuania	-0.69	-0.69	0.05	1.17	0.98	1.28
Luxembourg	-0.39	-0.39	-0.34	1.33	3.78	0.27
Madagascar	-3.16	-3.16	-1.88	1.31	0.65	2.32
Malawi	-0.75	-0.75	1.19	2.12	0.85	4.11
Malaysia	-0.69	-0.69	-1.77	1.63	1.22	2.45
Maldives	-0.92	-0.92	-0.12	1.9	1.26	3.64
Mali	1.57	1.57	0.66	1.7	1.29	2.38
Malta	-0.44	-0.44	0.81	0.81	0.93	0.82
Marshall Islands	0.25	0.25	0.38	0.8	1.01	0.54
Mauritania	-0.04	-0.04	-20.06	1.38	0.49	1.83
Mauritius	-0.94	-0.94	0.58	1.94	2.03	2.15
Mexico	-2.02	-2.02	-0.73	0.41	-0.25	1.15
Micronesia (Federated States of)	-0.3	-0.3	-1.25	1.24	1.99	0.54
Monaco	-0.45	-0.45	0.58	0.78	1.92	0.43
Mongolia	0.4	0.4	2.62	0.96	1.59	0.83
Montenegro	0.32	0.32	-1.03	0.75	1.1	0.67
Morocco	-1.03	-1.03	-1.62	1.8	2.04	1.82
Mozambique	-0.83	-0.83	3.69	2.02	1.08	3.86
Myanmar	-2.02	-2.02	5.66	2.38	1.84	4.32
Namibia	-0.82	-0.82	1.28	1.29	0.71	1.85
Nauru	-0.25	-0.25	0.2	0.14	0.94	-0.52
Nepal	-0.1	-0.1	6.46	2.85	2.06	4.9
Netherlands	-1.31	-1.31	-0.35	0.31	0.39	0.3
New Zealand	-2.03	-2.03	-1.39	0.99	1.56	0.83
Nicaragua	-1.35	-1.35	-0.89	1.32	0.76	2.07
Niger	1.63	1.63	1.13	1.86	2.16	1.66
Nigeria	0.51	0.51	0.15	1.74	0.78	2.73
Niue	-0.42	-0.42	-1.14	0.54	0.8	0.46
North Macedonia	0.1	0.1	-2.26	1.33	1.75	1.22
Northern Mariana Islands	-0.45	-0.45	1.31	0.22	1.26	-0.34
Norway	1.41	1.41	0.18	0.45	0.33	0.61
Oman	-0.99	-0.99	0.8	2.5	1.7	3.44
Pakistan	-0.93	-0.93	1.41	2.38	1.92	3.25
Palau	-0.41	-0.41	0.63	0.57	1.14	0.22
Palestine	-0.55	-0.55	-0.08	1.27	1.71	0.97
Panama	-1.71	-1.71	1.04	1.53	0.85	2.59
Papua New Guinea	-0.5	-0.5	-0.66	0.76	1.21	-0.02
Paraguay	-0.92	-0.92	-1.08	1.28	1.18	1.47
Peru	-0.33	-0.33	-1.41	1.64	1.33	1.92
Philippines	-1.53	-1.53	0.14	0.16	-0.59	1.46
Poland	-0.83	-0.83	1.13	0.61	0.76	0.64
Portugal	0.71	0.71	0.72	1.21	1.54	1.07
Puerto Rico	-0.15	-0.15	-2.87	0.76	0.7	0.95
Qatar	1.66	1.66	-1.28	1.33	1.58	1.44
Republic of Korea	-0.57	-0.57	-2.09	0.83	0.71	1.05
Republic of Moldova	0.59	0.59	-2.74	1.1	0.83	1.3
Romania	-0.68	-0.68	-0.83	1.1	1.12	1.18
Russian Federation	1.63	1.63	-1.86	1.33	1.32	1.39
Rwanda	1.48	1.48	-2.31	2.27	1.23	3.77
Saint Kitts and Nevis	-0.38	-0.38	-3.14	0.62	0.37	0.96
Saint Lucia	-0.33	-0.33	-1.5	0.74	0.43	1.32
Saint Vincent and the Grenadines	0.03	0.03	0.01	0.95	0.33	2.02
Samoa	0.03	0.03	-0.2	0.36	1.02	0.17
San Marino	-0.77	-0.77	-0.3	0.7	1.53	0.4
Sao Tome and Principe	0.92	0.92	1.46	1.58	1.15	2.12
Saudi Arabia	0.01	0.01	-0.74	1.48	1.32	1.86
Senegal	-0.61	-0.61	-0.61	1.61	1.43	2.01
Serbia	0.61	0.61	-1.03	0.93	1.2	0.89
Seychelles	-0.09	-0.09	0.32	1.63	2.14	0.96
Sierra Leone	-1.32	-1.32	-0.04	1.78	1.94	1.7
Singapore	-1.29	-1.29	-0.64	0.98	0.17	2.27
Slovakia	-0.7	-0.7	-0.65	0.65	0.69	0.69
Slovenia	0.03	0.03	-3.77	0.59	0.17	0.82
Solomon Islands	-0.42	-0.42	1.88	1.67	2.34	0.89
Somalia	-0.68	-0.68	0	1	1.03	0.95
South Africa	-2.77	-2.77	-1.39	1.11	0.96	1.32
South Sudan	-0.22	-0.22	-1.11	1.25	0.51	2.02
Spain	-1.37	-1.37	-0.71	-0.13	-0.1	-0.08
Sri Lanka	-2.71	-2.71	0.92	2.09	1.96	2.78
Sudan	0.02	0.02	-9.13	2.24	1.7	3.15
Suriname	-0.48	-0.48	-0.62	1.32	1.39	1.46
Sweden	-0.34	-0.34	-1.15	0.5	0.84	0.39
Switzerland	-0.45	-0.45	-1.56	0.58	0.87	0.45
Syrian Arab Republic	-0.61	-0.61	-2.68	1.23	1.45	1.22
Taiwan (Province of China)	-0.5	-0.5	-0.49	1.4	1.53	1.35
Tajikistan	-1.57	-1.57	-1.49	1.92	2.92	1.06
Thailand	-1.65	-1.65	1.85	1.79	0.97	3.19
Timor-Leste	-0.31	-0.31	2.53	2.08	2.17	1.91
Togo	-1.7	-1.7	-0.46	1.57	1.09	2.09
Tokelau	-0.73	-0.73	0.6	0.84	0.87	0.99
Tonga	-0.74	-0.74	1.68	0.79	1.13	0.6
Trinidad and Tobago	-1.19	-1.19	-0.54	0.79	0.49	1.23
Tunisia	-0.8	-0.8	1.15	1.48	1.7	1.51
Turkey	-0.95	-0.95	-0.2	1.22	2.03	1
Turkmenistan	-1.25	-1.25	2.38	1.35	1.72	1.31
Tuvalu	0.05	0.05	0.57	0.94	1.33	0.59
Uganda	0.1	0.1	0.15	1.83	0.96	3.66
Ukraine	0.44	0.44	-0.51	1.02	1.21	1.01
United Arab Emirates	0.53	0.53	3.59	1.04	0.82	1.5
United Kingdom	-1.02	-1.02	-0.52	1.05	1.87	0.69
United Republic of Tanzania	-0.53	-0.53	-0.31	1.52	1.27	1.8
United States of America	-1.28	-1.28	0.21	1.28	2.5	0.86
United States Virgin Islands	-0.46	-0.46	-0.43	0.9	1.09	0.92
Uruguay	0.34	0.34	0.77	1.69	3.12	1.33
Uzbekistan	0.47	0.47	0.13	1.9	3.42	1.36
Vanuatu	-0.56	-0.56	0.19	0.99	1.42	0.59
Venezuela (Bolivarian Republic of)	-2.16	-2.16	-1.89	0.8	0.63	1.07
Viet Nam	0.25	0.25	9.29	2.63	2.02	4.69
Yemen	0.39	0.39	-3.54	1.89	1.71	2.32
Zambia	0.35	0.35	0.27	1.58	0.86	2.58
Zimbabwe	-0.7	-0.7	-0.18	0.69	0.95	0.46

90%

10%

Figure S8. The ranking of risk factors for age-standardized DALY rate attributable to neurological disorders according to country.

Supplementary tables

Table S1. List of International Classification of Diseases (ICD) codes mapped to the Global Burden of Disease cause list for neurological disorders

Cause	ICD10	ICD9
Neurological disorders	F00-F02.0,F02.2-F02.3,F02.8-F03.9, G10-G13.8,G20-G20.9,G23-G24, G24.1-G25.0, G25.2-G25.3, G25.5, G25.8-G26.0,G30-G31.1,G31.8-G31.9, G35-G37.9,G40-G41.9,G61-G61.9, G70-G71.1, G71.3-G72, G72.2-G73.7, G90-G90.9, G95-G95.9, M33-M33.9	290-290.9,294.1-294.9,330-331.2, 331.5-332.0,333-337.9,340-341.9, 345-345.9,349,349.2-349.8,353.8-353.9,356-356.9,357.0-357.1,357.3-357.4,357.7, 358-359.9, 775.2
Stroke	G45-G46.8, I60-I63.9, I65-I66.9, I67.0-I67.3, I67.5-I67.6, I68.1-I68.2, I69.0-I69.3	430-435.9, 437.0-437.2, 437.5-437.8
Alzheimer's disease and other dementias	F00-F02.0, F02.8-F03.9, G30-G31.1, G31.8-G31.9	290-290.9, 294.1-294.9, 331-331.2

Parkinson's disease	F02.3, G20-G20.9	332-332.0
Idiopathic epilepsy	G40-G41.9	345-345.9
Multiple sclerosis	G35-G35.9	340-340.9
Headache disorders	-	-
Depressive disorders	F32.0–9, F33.0–9	-
Motor neuron disease	G12.2-G12.9	335-335.2, 335.8-335.9
Bipolar disorder	F30.0-F30.9, F31.0–F31.6, F31.8–F31.9, F34.0	-
Anxiety disorders	F40-42, F43.0, F43.1, F93.0-93.2, F93.8	-

Autism spectrum disorders	F84.0-F84.5, F84.8-F84.9	-

Table S2. The age-standardized rates of neurological disorders and its main subtypes in 1990 and 2019 and its temporal trends

	**Absolute numbers**		**Age-standardized rate**				
	**2019(95% UI)**	**EAPC (95% CI) (1990-2019)**	**2019(95% UI)**	**EAPC (95% CI) (1990-2019)**	**males, 2019 (95% UI)**	**females, 2019(95% UI)**	**male-to-female ratio**
**Neurological disorders**							
Incidence	805178458.07(725838130.61,888847391.91)	1.37(1.34,1.40)	10259.50(9223.20,11324.16)	-0.01(-0.02,0.01)	9737.45(8714.78,10772.62)	10784.59(9731.93,11861.19)	0.902904833
DALY	97724411.73(55942819.26,159416792.55)	1.83(1.82,1.84)	1253.56(719.70,2039.81)	-0.04(-0.05,-0.04)	1125.09(696.53,1754.77)	1377.77(725.51,2310.39)	0.816603261
**Stroke**							
Incidence	12224551.25(11041816.20,13589311.67)	1.70(1.63,1.77)	150.77(136.52,167.46)	-0.80(-0.86,-0.75)	151.10(136.90,167.54)	149.75(135.58,166.56)	1.009022131
DALY	143232184.23(133095808.90,153241823.73)	0.83(0.75,0.91)	1768.05(1640.65,1889.39)	-1.65(-1.76,-1.54)	2024.28(1852.42,2195.62)	1531.27(1397.07,1667.60)	1.321958922
**Alzheimer's disease and other dementias**							
Incidence	7236385.00(6217238.89,8232671.54)	3.19(3.12,3.27)	94.99(81.59,107.86)	0.06(0.03,0.08)	83.67(71.06,95.65)	103.45(89.24,117.15)	0.808841636
DALY	25276988.90(11204523.03,54558243.14)	3.40(3.35,3.46)	338.64(151.02,731.27)	0.15(0.13,0.16)	304.47(130.97,679.76)	361.20(162.80,767.72)	0.842959976
**Parkinson's disease**							
Incidence	1081722.68(953264.97,1211202.15)	3.34(3.28,3.40)	13.43(11.84,15.02)	0.61(0.57,0.65)	17.79(15.75,19.70)	9.95(8.77,11.18)	1.788421852
DALY	6292615.61(5769209.75,6827206.91)	2.94(2.91,2.98)	79.97(73.25,86.60)	0.10(0.03,0.17)	106.44(97.18,115.48)	59.91(53.22,65.43)	1.776626761

**Idiopathic epilepsy**							
Incidence	2898222.19(2098717.68,3823375.56)	1.48(1.45,1.52)	38.82(27.99,51.28)	0.49(0.46,0.52)	41.83(30.36,55.18)	35.78(25.63,47.63)	1.169110642
DALY	13077624.47(9986729.55,16734085.84)	0.35(0.30,0.40)	170.63(130.42,218.26)	-0.78(-0.82,-0.73)	187.41(145.35,237.74)	153.81(115.82,197.91)	1.218448606
**Multiple sclerosis**							
Incidence	59345.44(51817.76,66942.57)	1.29(1.27,1.31)	0.74(0.65,0.83)	-0.19(-0.24,-0.13)	0.55(0.48,0.63)	0.93(0.82,1.05)	0.593453788
DALY	1159831.84(1001179.90,1381870.17)	1.57(1.52,1.61)	13.96(12.05,16.63)	-0.56(-0.60,-0.52)	10.84(9.25,13.57)	16.98(14.32,20.53)	0.638408286
**Motor neuron disease**							
Incidence	63700.04(57295.90,71343.33)	2.06(1.96,2.16)	0.79(0.72,0.88)	0.03(-0.03,0.10)	0.90(0.82,1.00)	0.70(0.62,0.78)	1.298170335
DALY	1034606.59(979910.92,1085401.11)	1.70(1.57,1.82)	12.66(11.98,13.29)	-0.24(-0.29,-0.18)	14.89(13.97,15.68)	10.59(9.92,11.22)	1.405987018
**Headache disorders**							
Incidence	793839082.71(714299832.41,877018808.16)	1.35(1.32,1.38)	10110.72(9070.72,11167.13)	-0.01(-0.02,0.00)	9592.71(8567.82,10624.91)	10633.79(9577.73,11709.00)	0.902097177
DALY	46619354.78(9772903.22,100161725.60)	1.57(1.54,1.61)	581.76(119.58,1255.58)	0.04(0.02,0.06)	439.81(98.68,933.42)	724.63(139.40,1587.77)	0.606944248
**Depressive disorders**							
Incidence	290185741.58(256024052.35,328260552.75)	1.42(1.31,1.53)	3588.25(3152.71,4060.42)	-0.29(-0.38,-0.20)	2750.27(2419.66,3104.07)	4416.34(3886.90,5015.49)	0.622747273
DALY	46863642.09(32929363.02,63797315.23)	1.50(1.41,1.59)	577.75(405.79,788.88)	-0.24(-0.31,-0.16)	452.17(316.79,618.13)	702.08(492.30,963.58)	0.644040468
**Bipolar disorder**							
Incidence	3388806.16(2835180.38,4029561	1.43(1.39,1.48)	43.30(36.07,51.49)	0.13(0.11,0.14)	42.79(35.77,50.99)	43.85(36.42,52.18)	0.975806366

	.23)						
DALY	8502426.95(5200032.62,13046630.22)	1.64(1.61,1.67)	105.43(64.33,162.04)	0.02(0.01,0.02)	101.46(61.68,156.25)	109.43(66.67,167.46)	0.92715641
**Anxiety disorders**							
Incidence	45821085.57(37137079.53,55624209.44)	1.31(1.27,1.36)	585.45(474.21,709.53)	0.02(0.00,0.04)	490.50(401.07,592.36)	683.76(549.05,836.15)	0.717360957
DALY	28676050.45(19858079.22,39315123.17)	1.50(1.44,1.55)	360.12(248.60,494.44)	-0.01(-0.04,0.02)	275.20(191.39,377.98)	444.89(307.26,609.32)	0.618581229
**Autism spectrum disorders**							
Incidence	603789.86(501680.12,720096.53)	0.13(0.07,0.19)	9.32(7.75,11.12)	0.06(0.04,0.07)	13.72(11.48,16.25)	4.61(3.69,5.58)	2.972741777
DALY	4306615.38(2821511.91,6232360.47)	1.14(1.12,1.16)	56.26(36.82,81.52)	-0.02(-0.03,0.00)	85.34(55.77,123.55)	26.75(17.54,39.16)	3.19035227

DALY - disability adjusted life-year, CI - confidence interval, EAPC - estimated annual percentage change, SDI - socio-demographic index, UI - uncertainty interval.

Table S3. The age-standardized DALY rate of neurological disorders in 1990 and 2019 and its temporal trends

All neurological disorders DALY	Absolute numbers 2019(95% UI)	EAPC (95% CI) (1990-2019)	Age-standardized rate 2019(95% UI)	EAPC(95% CI) (1990-2019)	males, 2019 (95% UI)	females, 2019(95% UI)	male-to-female ratio
High-middle SDI	20676710.10(11919901.38,33556099.45)	1.55(1.54,1.57)	1218.74(691.84,1981.10)	-0.11(-0.13,-0.09)	1082.73(670.43,1686.23)	1354.95(706.71,2268.98)	0.799095634
High SDI	19237221.62(11714530.22,30377786.65)	1.72(1.68,1.76)	1303.51(736.19,2111.81)	0.07(0.05,0.09)	1113.90(699.22,1736.58)	1495.57(773.61,2526.92)	0.74480178
Low-middle SDI	19243323.01(10170116.96,32046998.27)	1.85(1.82,1.87)	1243.81(695.95,2023.73)	-0.12(-0.14,-0.10)	1134.81(681.23,1774.12)	1352.13(711.61,2298.17)	0.839276598
Low SDI	10231802.11(5619983.15,17107547.80)	2.51(2.48,2.54)	1236.62(720.11,1946.39)	-0.10(-0.11,-0.09)	1156.37(717.53,1780.50)	1313.78(716.69,2180.33)	0.880187407
Middle SDI	28280338.62(15309773.36,46788483.78)	1.90(1.88,1.92)	1217.94(673.68,2029.28)	0.01(0.00,0.02)	1089.16(653.16,1719.00)	1343.31(686.21,2312.50)	0.810800693
Andean Latin America	669928.47(403703.92,1061895.01)	2.11(2.09,2.14)	1118.87(681.97,1755.16)	-0.26(-0.28,-0.24)	1008.46(651.77,1505.68)	1228.46(698.85,2022.48)	0.820918567
Australasia	491658.92(307915.31,765069.43)	2.15(2.13,2.17)	1231.79(729.88,1943.21)	-0.01(-0.03,0.01)	1110.65(715.09,1717.78)	1344.86(737.59,2213.06)	0.825847725
Caribbean	622830.34(369601.00,1004427.45)	1.54(1.53,1.56)	1265.71(750.69,2029.87)	0.00(-0.05,0.05)	1155.76(755.46,1728.85)	1374.81(726.63,2302.72)	0.840673047
Central Asia	1088663.96(621623.26,1739682.86)	1.17(1.11,1.23)	1363.07(825.00,2139.12)	0.07(-0.02,0.16)	1276.25(843.47,1898.27)	1454.27(807.01,2392.51)	0.877585173
Central Europe	2074727.71(1273406.36,3301261.70)	0.72(0.67,0.76)	1292.88(771.72,2054.27)	-0.14(-0.15,-0.13)	1164.43(744.71,1784.90)	1423.57(795.60,2355.52)	0.817968742
Central Latin America	3235562.19(1842436.00,5218607.81)	2.10(2.08,2.12)	1323.08(766.24,2115.09)	-0.10(-0.15,-0.05)	1171.73(756.37,1778.44)	1466.62(780.61,2451.02)	0.798937269

Central sub-Saharan Africa	1205481.24(664567.40,1995338.87)	2.83(2.78,2.89)	1325.20(778.64,2094.20)	-0.07(-0.11,-0.03)	1260.10(783.84,1952.56)	1381.57(763.57,2272.71)	0.912077395
East Asia	18034421.21(10031195.66,30122084.33)	1.73(1.70,1.76)	1076.34(597.98,1781.78)	-0.12(-0.15,-0.10)	969.14(590.57,1525.14)	1178.77(601.40,2042.84)	0.82216066
Eastern Europe	3416892.42(2071105.57,5379221.32)	0.42(0.38,0.46)	1264.48(739.10,2029.07)	-0.02(-0.06,0.02)	1097.24(689.52,1718.01)	1425.61(790.73,2369.16)	0.769664397
Eastern sub-Saharan Africa	3064703.98(1823369.55,4849527.73)	2.60(2.56,2.64)	1117.19(685.41,1725.53)	-0.04(-0.05,-0.02)	1051.47(678.04,1584.86)	1175.36(685.28,1861.66)	0.894595345
High-income Asia Pacific	3967450.24(2400554.50,6645145.72)	2.88(2.80,2.96)	1067.56(616.47,1753.82)	0.22(0.18,0.26)	939.55(574.22,1509.74)	1188.99(639.00,1994.03)	0.790203366
High-income North America	6570271.74(4013981.95,10380241.11)	1.46(1.43,1.49)	1340.08(757.36,2177.42)	0.00(-0.04,0.04)	1114.30(722.32,1714.52)	1562.89(777.33,2706.29)	0.712976229
North Africa and middle East	7156040.57(3769688.41,12184398.02)	2.31(2.28,2.34)	1382.14(776.61,2273.81)	-0.11(-0.13,-0.09)	1232.61(745.16,1943.39)	1545.47(812.66,2649.12)	0.797565334
Oceania	120254.91(58684.87,206952.44)	2.69(2.68,2.71)	1237.37(697.50,2026.43)	-0.10(-0.11,-0.08)	1178.79(730.85,1842.18)	1297.77(650.26,2232.28)	0.908318006
South Asia	19068926.20(9725695.87,32172757.88)	1.91(1.87,1.95)	1189.75(648.45,1975.49)	-0.24(-0.28,-0.21)	1085.03(635.80,1723.62)	1298.88(669.81,2211.15)	0.835352232
Southeast Asia	7705142.85(3582954.02,13824792.52)	1.96(1.93,1.98)	1240.33(627.59,2145.96)	0.03(0.02,0.04)	1088.04(592.15,1805.62)	1380.21(625.93,2529.64)	0.788317246
Southern Latin America	848095.86(519744.05,1335733.82)	1.92(1.89,1.95)	1119.57(663.45,1775.37)	0.14(0.12,0.15)	1013.74(664.48,1536.87)	1223.56(661.47,2027.86)	0.828515318
Southern sub-Saharan Africa	843682.55(482667.68,1356713.19)	1.51(1.32,1.70)	1262.17(755.95,1978.92)	-0.10(-0.21,0.01)	1215.26(781.79,1841.35)	1299.66(724.23,2117.62)	0.935057931
Tropical Latin America	3367762.36(1750228.68,5686900.12)	2.16(2.13,2.19)	1471.72(769.26,2492.03)	0.06(0.01,0.11)	1305.49(763.21,2118.38)	1634.52(778.32,2913.02)	0.798695203

Western Europe	9417304.06(5678674.45,14885141.28)	1.33(1.31,1.36)	1432.56(777.08,2368.95)	0.08(0.06,0.10)	1199.05(720.26,1881.47)	1664.25(825.21,2921.31)	0.720472109
Western sub-Saharan Africa	4754609.96(2619701.88,7979177.21)	3.04(3.00,3.09)	1425.08(834.27,2270.80)	0.12(0.09,0.15)	1364.81(851.83,2084.69)	1477.36(791.24,2463.63)	0.923817143

CI - confidence interval, EAPC - estimated annual percentage change, SDI - socio-demographic index, UI - uncertainty interval.

Table S4. The age-standardized DALY rate of neurological disorders in 1990 and 2019 and its temporal trends

All neurological disorders DALY	Absolute numbers 2019(95% UI)	EAPC (95% CI) (1990-2019)	Age-standardized rate 2019(95% UI)	EAPC(95% CI) (1990-2019)	males, 2019 (95% UI)	females, 2019(95% UI)	male-to-female ratio
High-middle SDI	20676710.10(11919901.38,33556099.45)	1.55(1.54,1.57)	1218.74(691.84,1981.10)	-0.11(-0.13,-0.09)	1082.73(670.43,1686.23)	1354.95(706.71,2268.98)	0.799095634
High SDI	19237221.62(11714530.22,30377786.65)	1.72(1.68,1.76)	1303.51(736.19,2111.81)	0.07(0.05,0.09)	1113.90(699.22,1736.58)	1495.57(773.61,2526.92)	0.74480178
Low-middle SDI	19243323.01(10170116.96,32046998.27)	1.85(1.82,1.87)	1243.81(695.95,2023.73)	-0.12(-0.14,-0.10)	1134.81(681.23,1774.12)	1352.13(711.61,2298.17)	0.839276598
Low SDI	10231802.11(5619983.15,17107547.80)	2.51(2.48,2.54)	1236.62(720.11,1946.39)	-0.10(-0.11,-0.09)	1156.37(717.53,1780.50)	1313.78(716.69,2180.33)	0.880187407
Middle SDI	28280338.62(15309773.36,46788483.78)	1.90(1.88,1.92)	1217.94(673.68,2029.28)	0.01(0.00,0.02)	1089.16(653.16,1719.00)	1343.31(686.21,2312.50)	0.810800693
Andean Latin America	669928.47(403703.92,1061895.01)	2.11(2.09,2.14)	1118.87(681.97,1755.16)	-0.26(-0.28,-0.24)	1008.46(651.77,1505.68)	1228.46(698.85,2022.48)	0.820918567
Australasia	491658.92(307915.31,765069.43)	2.15(2.13,2.17)	1231.79(729.88,1943.21)	-0.01(-0.03,0.01)	1110.65(715.09,1717.78)	1344.86(737.59,2213.06)	0.825847725
Caribbean	622830.34(369601.00,1004427.45)	1.54(1.53,1.56)	1265.71(750.69,2029.87)	0.00(-0.05,0.05)	1155.76(755.46,1728.85)	1374.81(726.63,2302.72)	0.840673047
Central Asia	1088663.96(621623.26,1739682.86)	1.17(1.11,1.23)	1363.07(825.00,2139.12)	0.07(-0.02,0.16)	1276.25(843.47,1898.27)	1454.27(807.01,2392.51)	0.877585173
Central Europe	2074727.71(1273406.36,3301261.70)	0.72(0.67,0.76)	1292.88(771.72,2054.27)	-0.14(-0.15,-0.13)	1164.43(744.71,1784.90)	1423.57(795.60,2355.52)	0.817968742
Central Latin America	3235562.19(1842436.00,5218607.81)	2.10(2.08,2.12)	1323.08(766.24,2115.09)	-0.10(-0.15,-0.05)	1171.73(756.37,1778.44)	1466.62(780.61,2451.02)	0.798937269
Central sub-Saharan Africa	1205481.24(664567.40,1995338.87)	2.83(2.78,2.89)	1325.20(778.64,2094.20)	-0.07(-0.11,-0.03)	1260.10(783.84,1952.56)	1381.57(763.57,2272.71)	0.912077395
East Asia	18034421.21(10031195.66,30122084.33)	1.73(1.70,1.76)	1076.34(597.98,1781.78)	-0.12(-0.15,-0.10)	969.14(590.57,1525.14)	1178.77(601.40,2042.84)	0.82216066
Eastern Europe	3416892.42(2071105.57,5379221.32)	0.42(0.38,0.46)	1264.48(739.10,2029.07)	-0.02(-0.06,0.02)	1097.24(689.52,1718.01)	1425.61(790.73,2369.16)	0.769664397
Eastern sub-Saharan Africa	3064703.98(1823369.55,4849527.73)	2.60(2.56,2.64)	1117.19(685.41,1725.53)	-0.04(-0.05,-0.02)	1051.47(678.04,1584.86)	1175.36(685.28,1861.66)	0.894595345
High-income Asia Pacific	3967450.24(2400554.50,6645145.72)	2.88(2.80,2.96)	1067.56(616.47,1753.82)	0.22(0.18,0.26)	939.55(574.22,1509.74)	1188.99(639.00,1994.03)	0.790203366
High-income North	6570271.74(4013981.95,10380241.11)	1.46(1.43,1.49)	1340.08(757.36,2177.42)	0.00(-0.04,0.04)	1114.30(722.32,1714.52)	1562.89(777.33,2706.29)	0.712976229

America							
North Africa and middle East	7156040.57(3769688.41,12184398.02)	2.31(2.28,2.34)	1382.14(776.61,2273.81)	-0.11(-0.13,-0.09)	1232.61(745.16,1943.39)	1545.47(812.66,2649.12)	0.797565334
Oceania	120254.91(58684.87,206952.44)	2.69(2.68,2.71)	1237.37(697.50,2026.43)	-0.10(-0.11,-0.08)	1178.79(730.85,1842.18)	1297.77(650.26,2232.28)	0.908318006
South Asia	19068926.20(9725695.87,32172757.88)	1.91(1.87,1.95)	1189.75(648.45,1975.49)	-0.24(-0.28,-0.21)	1085.03(635.80,1723.62)	1298.88(669.81,2211.15)	0.835352232
Southeast Asia	7705142.85(3582954.02,13824792.52)	1.96(1.93,1.98)	1240.33(627.59,2145.96)	0.03(0.02,0.04)	1088.04(592.15,1805.62)	1380.21(625.93,2529.64)	0.788317246
Southern Latin America	848095.86(519744.05,1335733.82)	1.92(1.89,1.95)	1119.57(663.45,1775.37)	0.14(0.12,0.15)	1013.74(664.48,1536.87)	1223.56(661.47,2027.86)	0.828515318
Southern sub-Saharan Africa	843682.55(482667.68,1356713.19)	1.51(1.32,1.70)	1262.17(755.95,1978.92)	-0.10(-0.21,0.01)	1215.26(781.79,1841.35)	1299.66(724.23,2117.62)	0.935057931
Tropical Latin America	3367762.36(1750228.68,5686900.12)	2.16(2.13,2.19)	1471.72(769.26,2492.03)	0.06(0.01,0.11)	1305.49(763.21,2118.38)	1634.52(778.32,2913.02)	0.798695203
Western Europe	9417304.06(5678674.45,14885141.28)	1.33(1.31,1.36)	1432.56(777.08,2368.95)	0.08(0.06,0.10)	1199.05(720.26,1881.47)	1664.25(825.21,2921.31)	0.720472109
Western sub-Saharan Africa	4754609.96(2619701.88,7979177.21)	3.04(3.00,3.09)	1425.08(834.27,2270.80)	0.12(0.09,0.15)	1364.81(851.83,2084.69)	1477.36(791.24,2463.63)	0.923817143

DALY - disability adjusted life-year, CI - confidence interval, EAPC - estimated annual percentage change, SDI - socio-demographic index, UI - uncertainty interval.

Table S5. The age-standardized incidence rate of other neurological disorders in 1990 and 2019 and its temporal trends, by regions

Incidence		Absolute numbers		Age-standardized rate				
	**Regions**	**2019(95% UI)**	**EAPC (95% CI) (1990-2019)**	**2019(95% UI)**	**EAPC(95% CI) (1990-2019)**	**2019(95% UI)**	**females, 2019(95% UI)**	**male-to-female ratio**
Stroke	Andean Latin America	50905.43(46863.16,55292.91)	1.98(1.92,2.05)	87.92(80.84,95.61)	-1.02(-1.09,-0.94)	86.24(79.18,94.04)	89.41(82.35,97.22)	0.964460819
Stroke	Australasia	30177.98(27219.54,33371.10)	0.41(0.27,0.55)	65.12(59.02,71.76)	-2.01(-2.16,-1.87)	60.49(54.67,66.63)	69.12(62.12,76.81)	0.875213551
Stroke	Caribbean	65932.66(61053.51,71393.94)	1.81(1.77,1.86)	129.46(119.82,140.14)	-0.37(-0.45,-0.28)	126.94(117.25,137.38)	131.65(121.84,142.69)	0.964258247
Stroke	Central Asia	143309.99(134324.54,153634.60)	0.73(0.67,0.79)	195.23(182.89,208.84)	-0.72(-0.88,-0.56)	212.19(199.28,227.12)	180.59(168.22,194.19)	1.174960064
Stroke	Central Europe	307696.67(277952.89,341580.33)	-0.32(-0.36,-0.28)	150.75(137.15,165.81)	-1.63(-1.69,-1.56)	154.79(141.20,169.45)	145.76(132.00,161.34)	1.061927171
Stroke	Central Latin America	217716.28(198722.89,239098.14)	1.68(1.55,1.81)	91.62(83.63,100.66)	-1.50(-1.66,-1.35)	88.12(80.44,96.73)	94.73(86.43,104.32)	0.930270828
Stroke	Central sub-Saharan Africa	98796.43(90494.46,108018.83)	2.18(2.09,2.26)	162.29(149.79,176.61)	-0.78(-0.83,-0.73)	161.44(148.58,176.36)	162.65(149.34,178.37)	0.992581188
Stroke	East Asia	4068857.93(3557532.88,4727907.12)	2.56(2.47,2.64)	200.56(176.98,230.24)	-0.61(-0.73,-0.49)	209.05(185.59,238.17)	194.22(169.77,224.73)	1.076363048
Stroke	Eastern Europe	629927.92(557738.12,715756.29)	-0.50(-0.55,-0.45)	192.01(171.50,216.19)	-1.18(-1.27,-1.10)	200.55(178.78,225.06)	181.31(161.32,204.77)	1.106155437
Stroke	Eastern sub-Saharan Africa	309261.98(282035.81,340679.38)	1.77(1.66,1.88)	157.83(145.08,172.61)	-0.98(-1.05,-0.91)	160.22(147.09,176.03)	155.60(142.83,170.28)	1.029691472
Stroke	High-income Asia Pacific	506161.61(458159.23,562656.50)	0.95(0.85,1.06)	132.98(120.36,147.16)	-1.31(-1.35,-1.26)	126.53(114.56,139.91)	137.30(123.14,152.90)	0.921564741

Stroke	High-income North America	498551.06(440176.50,569317.46)	0.65(0.55,0.75)	84.80(75.48,95.55)	-1.17(-1.24,-1.10)	74.03(66.14,83.28)	93.55(82.89,105.54)	0.791291472
Stroke	North Africa and middle East	829802.94(758352.03,912811.74)	2.89(2.83,2.95)	182.99(166.73,201.74)	-0.21(-0.24,-0.17)	171.75(156.30,189.46)	194.66(177.47,214.01)	0.882296571
Stroke	Oceania	18180.45(17015.38,19501.06)	2.80(2.78,2.82)	216.51(202.43,232.20)	-0.29(-0.30,-0.27)	218.45(203.93,234.54)	214.22(199.86,230.71)	1.019782675
Stroke	South Asia	1698563.07(1524227.95,1894096.28)	2.49(2.34,2.63)	117.32(105.90,130.41)	-0.54(-0.63,-0.45)	114.90(103.76,128.08)	119.64(108.04,133.13)	0.960343086
Stroke	Southeast Asia	1328397.33(1215681.61,1470865.39)	2.54(2.52,2.56)	215.89(197.75,238.19)	-0.32(-0.34,-0.29)	230.83(211.73,253.80)	201.35(183.44,222.89)	1.1464212
Stroke	Southern Latin America	72610.79(67102.15,78895.80)	0.20(0.08,0.32)	90.00(83.14,97.44)	-1.76(-1.88,-1.65)	91.17(84.31,98.50)	88.63(81.33,96.93)	1.028690216
Stroke	Southern sub-Saharan Africa	91734.11(81994.35,104139.70)	1.79(1.53,2.05)	163.55(145.06,186.32)	-0.19(-0.43,0.05)	158.07(140.03,180.12)	165.89(147.43,189.01)	0.952862291
Stroke	Tropical Latin America	303521.49(271616.58,340464.16)	0.92(0.83,1.02)	127.26(114.21,142.33)	-2.10(-2.20,-2.01)	138.57(123.47,157.52)	118.18(106.72,130.77)	1.172519052
Stroke	Western Europe	602980.84(545933.99,666263.50)	-0.61(-0.69,-0.53)	69.82(63.46,76.61)	-2.09(-2.16,-2.01)	69.45(63.49,76.42)	69.55(62.80,76.35)	0.998535485
Stroke	Western sub-Saharan Africa	351464.29(321419.96,383682.35)	2.38(2.33,2.44)	158.93(146.92,173.39)	-0.49(-0.53,-0.46)	145.62(134.54,158.86)	170.85(157.16,186.20)	0.852332784
Alzheimer's disease and other dementias	Andean Latin America	44044.29(37768.26,50205.86)	4.23(4.20,4.26)	83.48(71.56,95.52)	0.11(0.08,0.13)	79.56(67.59,91.31)	86.94(74.59,99.31)	0.91514177
Alzheimer's disease and other dementias	Australasia	52430.42(44896.19,59599.32)	3.13(3.10,3.17)	93.84(80.46,106.76)	-0.06(-0.07,-0.04)	76.33(64.26,87.23)	108.06(92.81,123.06)	0.70631151
Alzheimer's disease and other dementias	Caribbean	40874.64(35108.72,46713.37)	2.90(2.85,2.95)	78.42(67.22,89.79)	0.03(0.00,0.05)	75.10(63.50,86.18)	81.19(69.97,92.89)	0.924984541

Alzheimer's disease and other dementias	Central Asia	50654.95(43028.29,58476.61)	0.86(0.68,1.04)	102.74(87.44,117.42)	0.05(0.02,0.08)	96.76(81.20,110.97)	106.90(91.49,122.12)	0.905166871
Alzheimer's disease and other dementias	Central Europe	243264.41(203462.13,279724.99)	2.25(2.16,2.34)	106.31(89.80,121.85)	0.09(0.08,0.10)	96.94(79.82,111.56)	113.39(96.31,129.58)	0.854931194
Alzheimer's disease and other dementias	Central Latin America	182907.58(156452.90,209457.64)	4.38(4.34,4.42)	82.90(70.84,95.18)	0.00(-0.03,0.03)	78.60(67.00,90.25)	86.35(73.82,99.16)	0.910187696
Alzheimer's disease and other dementias	Central sub-Saharan Africa	32617.32(28308.83,37146.71)	3.24(3.19,3.29)	98.35(85.22,112.11)	0.08(0.07,0.09)	81.34(69.72,93.00)	109.62(94.86,124.63)	0.742008905
Alzheimer's disease and other dementias	East Asia	1861337.09(1570265.82,2153332.31)	4.23(4.14,4.32)	103.32(87.60,118.29)	0.33(0.26,0.39)	89.11(74.62,102.65)	114.65(97.18,131.81)	0.777209227
Alzheimer's disease and other dementias	Eastern Europe	359981.04(299239.56,417073.82)	1.63(1.48,1.78)	101.93(85.52,116.99)	0.17(0.13,0.20)	95.02(78.93,109.42)	105.88(89.28,121.71)	0.897472416
Alzheimer's disease and other dementias	Eastern sub-Saharan Africa	92584.92(80495.00,105549.13)	2.91(2.79,3.03)	85.08(73.42,96.76)	-0.02(-0.05,0.02)	73.14(62.51,83.57)	94.81(82.34,107.99)	0.771368834
Alzheimer's disease and other dementias	High-income Asia Pacific	628980.21(540697.49,713926.28)	4.81(4.72,4.89)	108.90(93.99,123.75)	0.48(0.45,0.52)	88.16(75.00,101.23)	123.27(106.65,139.99)	0.715147421
Alzheimer's disease and other dementias	High-income North America	740732.19(657407.20,816178.82)	2.01(1.96,2.06)	105.77(93.77,116.93)	-0.04(-0.09,0.00)	95.83(82.97,106.69)	112.61(100.87,123.93)	0.85099417
Alzheimer's disease and other dementias	North Africa and middle East	361192.21(309692.11,413138.21)	3.64(3.62,3.66)	110.17(93.94,125.62)	0.04(0.02,0.06)	105.52(89.78,120.78)	114.85(98.45,130.84)	0.918778851
Alzheimer's disease and other dementias	Oceania	3626.69(3088.88,4162.60)	3.03(2.99,3.08)	86.26(73.29,98.98)	-0.09(-0.12,-0.06)	76.19(64.25,87.96)	95.45(80.93,109.56)	0.798244524
Alzheimer's disease and other dementias	South Asia	678660.74(581647.41,781921.28)	4.05(3.85,4.26)	63.63(54.20,72.95)	-0.04(-0.10,0.02)	61.74(52.27,71.30)	65.41(56.15,74.73)	0.943860452
Alzheimer's disease and other dementias	Southeast Asia	402322.83(347099.90,456730.00)	3.42(3.36,3.48)	84.80(72.56,96.84)	0.02(-0.02,0.06)	71.72(60.96,82.10)	94.15(80.76,107.42)	0.761703204

Alzheimer's disease and other dementias	Southern Latin America	82527.00(70037.55,94751.14)	2.78(2.73,2.83)	95.18(80.96,109.17)	0.10(0.09,0.12)	84.66(70.70,97.85)	101.85(87.31,116.59)	0.831168012
Alzheimer's disease and other dementias	Southern sub-Saharan Africa	38365.43(33144.25,44024.38)	2.24(2.15,2.33)	89.95(77.28,102.68)	-0.01(-0.06,0.05)	77.35(65.82,88.61)	97.28(83.87,110.89)	0.795108203
Alzheimer's disease and other dementias	Tropical Latin America	236870.19(206086.09,268576.49)	4.37(4.31,4.43)	104.09(90.49,118.13)	0.09(0.07,0.10)	98.85(85.18,112.32)	107.95(94.14,122.73)	0.915732774
Alzheimer's disease and other dementias	Western Europe	1009029.26(857523.86,1155105.08)	2.08(2.02,2.13)	91.52(78.13,104.43)	-0.05(-0.07,-0.03)	77.24(65.06,88.99)	101.73(87.53,116.09)	0.759262361
Alzheimer's disease and other dementias	Western sub-Saharan Africa	93381.58(80981.36,106419.77)	2.53(2.40,2.65)	73.47(62.61,83.94)	-0.11(-0.18,-0.03)	66.36(56.32,75.95)	79.91(68.50,91.40)	0.83046516
Parkinson's disease	Andean Latin America	6841.07(6242.19,7439.13)	4.48(4.39,4.57)	12.58(11.50,13.68)	0.70(0.64,0.76)	15.65(14.22,17.17)	9.78(8.86,10.69)	1.600752087
Parkinson's disease	Australasia	7692.65(6777.89,8751.44)	2.90(2.73,3.06)	15.23(13.33,17.39)	0.27(0.08,0.46)	20.91(18.41,24.30)	10.26(8.81,11.72)	2.037000039
Parkinson's disease	Caribbean	5936.42(5409.05,6479.77)	3.05(2.97,3.14)	11.51(10.51,12.54)	0.55(0.47,0.63)	14.87(13.57,16.35)	8.65(7.82,9.47)	1.719771221
Parkinson's disease	Central Asia	7083.01(6329.94,7878.41)	1.65(1.57,1.73)	11.40(10.47,12.42)	0.48(0.44,0.52)	14.78(13.57,16.16)	9.32(8.44,10.21)	1.584934419
Parkinson's disease	Central Europe	26311.85(24211.05,28471.88)	1.59(1.55,1.62)	11.72(10.83,12.62)	0.08(0.05,0.10)	14.92(13.76,16.21)	9.60(8.88,10.38)	1.55328716
Parkinson's disease	Central Latin America	25324.31(22670.08,27958.64)	4.31(4.24,4.38)	11.11(9.95,12.26)	0.36(0.31,0.42)	14.27(12.72,15.85)	8.48(7.59,9.34)	1.683218849
Parkinson's disease	Central sub-Saharan Africa	3477.27(3018.28,3966.81)	3.06(2.96,3.15)	7.96(7.05,8.95)	0.09(0.04,0.14)	10.01(8.88,11.21)	6.58(5.80,7.49)	1.521176348
Parkinson's disease	East Asia	311857.34(260766.85,362952.62)	3.85(3.69,4.01)	15.26(12.88,17.70)	0.46(0.30,0.62)	19.74(16.77,22.79)	11.96(10.11,13.88)	1.649755302

Parkinson's disease	Eastern Europe	36226.94(30437.26,42265.84)	0.68(0.62,0.75)	10.30(8.70,11.96)	-0.03(-0.09,0.03)	14.36(12.06,16.61)	8.18(6.92,9.52)	1.754819545
Parkinson's disease	Eastern sub-Saharan Africa	10663.83(9416.73,12058.24)	2.73(2.61,2.84)	7.70(6.85,8.61)	0.00(-0.02,0.02)	9.57(8.55,10.62)	6.14(5.43,6.90)	1.558009767
Parkinson's disease	High-income Asia Pacific	44118.33(37490.11,50796.24)	3.77(3.66,3.88)	9.32(7.97,10.61)	0.62(0.53,0.71)	11.59(9.94,13.20)	7.66(6.59,8.75)	1.513643727
Parkinson's disease	High-income North America	164042.83(142420.62,186991.62)	4.70(4.14,5.26)	25.14(21.88,28.66)	2.74(2.28,3.19)	42.50(36.34,49.12)	10.96(9.83,12.14)	3.877965381
Parkinson's disease	North Africa and middle East	42804.29(38332.45,47338.11)	3.82(3.79,3.86)	11.40(10.28,12.54)	0.46(0.43,0.48)	13.60(12.37,14.93)	9.21(8.20,10.26)	1.477401223
Parkinson's disease	Oceania	868.85(762.96,976.83)	2.76(2.70,2.83)	14.85(13.33,16.54)	-0.21(-0.25,-0.18)	18.53(16.64,20.68)	11.34(10.14,12.73)	1.634408229
Parkinson's disease	South Asia	128493.85(108402.57,149539.21)	3.97(3.86,4.08)	10.07(8.50,11.60)	0.26(0.23,0.30)	11.98(10.15,13.81)	8.34(7.01,9.70)	1.436231242
Parkinson's disease	Southeast Asia	65111.21(57547.24,73055.80)	3.43(3.36,3.49)	11.79(10.52,13.17)	0.31(0.25,0.36)	13.78(12.25,15.30)	10.20(9.04,11.46)	1.351267393
Parkinson's disease	Southern Latin America	10428.21(9417.98,11482.21)	2.34(2.28,2.40)	12.21(11.03,13.45)	0.16(0.09,0.23)	18.04(16.28,20.10)	7.98(7.19,8.87)	2.260091597
Parkinson's disease	Southern sub-Saharan Africa	4527.54(3882.44,5218.19)	2.80(2.74,2.86)	9.24(7.94,10.58)	0.55(0.49,0.60)	11.89(10.12,13.60)	7.58(6.46,8.64)	1.569173971
Parkinson's disease	Tropical Latin America	25465.84(21496.03,29589.99)	4.14(4.06,4.22)	10.83(9.13,12.58)	0.42(0.34,0.49)	13.01(10.94,15.19)	9.06(7.64,10.49)	1.437067468
Parkinson's disease	Western Europe	138245.43(123835.01,150577.95)	2.06(1.96,2.16)	14.61(13.03,16.05)	0.41(0.32,0.50)	18.83(16.80,20.79)	11.38(10.17,12.44)	1.654369082
Parkinson's disease	Western sub-Saharan Africa	16201.61(14535.11,17990.81)	2.90(2.83,2.98)	10.76(9.67,11.83)	0.39(0.32,0.46)	11.77(10.56,13.01)	9.81(8.78,10.84)	1.199857668
Idiopathic epilepsy	Andean Latin America	35115.27(15969.65,53254.24)	1.36(1.26,1.46)	55.28(25.08,83.69)	-0.03(-0.12,0.07)	59.10(26.72,90.31)	51.39(23.30,77.98)	1.150021756

Idiopathic epilepsy	Australasia	11226.37(4314.62,17062.08)	1.09(1.04,1.15)	42.48(16.51,65.72)	-0.02(-0.05,0.02)	44.52(16.97,68.81)	40.47(16.04,63.07)	1.100141583
Idiopathic epilepsy	Caribbean	19155.45(11544.53,28018.34)	0.75(0.71,0.79)	42.00(25.04,61.48)	0.05(-0.02,0.11)	44.58(26.82,65.30)	39.46(23.22,58.89)	1.129637776
Idiopathic epilepsy	Central Asia	42939.27(25092.51,62687.92)	0.91(0.83,0.98)	46.00(27.06,66.90)	0.28(0.22,0.35)	52.62(31.09,76.09)	39.66(23.38,57.97)	1.326612299
Idiopathic epilepsy	Central Europe	45147.02(30176.83,60978.13)	0.11(0.05,0.16)	42.08(27.00,57.95)	0.42(0.39,0.45)	47.30(30.36,65.11)	36.91(23.60,51.75)	1.281552999
Idiopathic epilepsy	Central Latin America	139938.45(93562.03,194329.93)	1.00(0.97,1.03)	57.10(38.03,78.58)	-0.03(-0.10,0.03)	58.90(39.17,80.41)	55.33(36.99,76.43)	1.064458389
Idiopathic epilepsy	Central sub-Saharan Africa	69979.19(27626.91,120878.45)	3.22(3.05,3.40)	46.18(18.50,77.28)	0.24(0.07,0.42)	52.24(21.03,86.62)	40.71(16.01,68.42)	1.283215514
Idiopathic epilepsy	East Asia	316357.73(217738.27,422618.39)	0.71(0.40,1.02)	24.59(16.63,33.63)	0.69(0.39,0.99)	26.33(17.95,35.61)	22.82(15.51,31.55)	1.153949188
Idiopathic epilepsy	Eastern Europe	62845.70(43101.17,85367.80)	-0.48(-0.65,-0.32)	33.34(22.76,46.70)	-0.05(-0.13,0.03)	38.15(26.21,52.97)	28.91(19.60,40.95)	1.319441211
Idiopathic epilepsy	Eastern sub-Saharan Africa	211624.80(128354.69,307255.13)	2.72(2.67,2.77)	46.53(28.94,65.54)	0.17(0.06,0.29)	52.62(32.79,73.53)	41.38(25.49,58.94)	1.271596095
Idiopathic epilepsy	High-income Asia Pacific	61714.93(38904.92,84079.43)	0.01(-0.04,0.06)	41.09(24.77,58.18)	0.12(0.05,0.20)	44.50(26.88,63.13)	37.60(22.74,53.66)	1.183634901
Idiopathic epilepsy	High-income North America	131008.99(87228.31,176696.67)	0.93(0.78,1.08)	38.87(25.59,53.30)	0.03(-0.11,0.17)	42.46(27.88,58.33)	35.22(22.85,48.70)	1.205505351
Idiopathic epilepsy	North Africa and middle East	295453.09(195044.30,407681.64)	1.97(1.91,2.03)	48.24(32.23,66.36)	0.46(0.41,0.52)	51.92(34.67,71.26)	44.28(29.71,61.15)	1.172681822
Idiopathic epilepsy	Oceania	3902.15(1570.76,6815.48)	2.38(2.31,2.45)	27.21(11.29,46.92)	0.01(-0.06,0.08)	30.42(12.34,52.52)	23.76(9.95,41.19)	1.280434012

Idiopathic epilepsy	South Asia	628981.06(423198.09,854118.28)	1.92(1.81,2.04)	35.09(23.88,47.28)	0.58(0.47,0.69)	37.89(25.71,51.10)	32.14(21.97,43.38)	1.178958984
Idiopathic epilepsy	Southeast Asia	230788.59(159049.88,309242.09)	1.52(1.48,1.55)	36.21(25.02,48.62)	0.75(0.70,0.80)	37.79(25.96,50.97)	34.66(23.56,46.97)	1.090325278
Idiopathic epilepsy	Southern Latin America	27018.04(11574.57,41186.06)	1.05(0.99,1.11)	43.48(18.56,67.13)	0.34(0.28,0.40)	46.43(20.31,71.53)	40.59(17.10,63.09)	1.143804649
Idiopathic epilepsy	Southern sub-Saharan Africa	32104.43(20810.77,45193.44)	0.71(0.39,1.03)	41.48(27.07,57.53)	-0.32(-0.58,-0.07)	45.26(29.50,62.68)	37.91(24.57,52.58)	1.193755992
Idiopathic epilepsy	Tropical Latin America	95532.35(61961.05,131916.43)	0.87(0.51,1.22)	44.56(28.72,61.90)	-0.13(-0.52,0.26)	48.25(31.24,66.87)	41.00(26.47,57.49)	1.176821237
Idiopathic epilepsy	Western Europe	191243.01(121827.92,258707.73)	1.01(0.94,1.08)	47.12(29.21,66.21)	0.48(0.41,0.55)	49.94(31.00,69.89)	44.28(27.62,62.55)	1.127747236
Idiopathic epilepsy	Western sub-Saharan Africa	246146.29(156193.89,347836.92)	3.06(2.87,3.26)	47.55(31.62,64.79)	0.12(-0.03,0.28)	52.80(35.38,71.52)	42.53(28.34,58.67)	1.24152218
Multiple sclerosis	Andean Latin America	206.38(164.84,243.56)	3.04(2.94,3.14)	0.32(0.26,0.38)	0.63(0.57,0.70)	0.22(0.18,0.26)	0.43(0.34,0.50)	0.513450553
Multiple sclerosis	Australasia	592.83(519.83,664.45)	2.03(1.78,2.29)	2.09(1.82,2.35)	1.21(0.90,1.52)	1.36(1.18,1.56)	2.81(2.44,3.17)	0.485031346
Multiple sclerosis	Caribbean	230.34(188.65,270.65)	1.46(1.35,1.57)	0.47(0.38,0.55)	0.26(0.22,0.30)	0.30(0.25,0.36)	0.63(0.52,0.74)	0.47990487
Multiple sclerosis	Central Asia	1351.56(1176.19,1536.52)	1.71(1.60,1.83)	1.39(1.21,1.58)	-0.36(-0.39,-0.32)	1.43(1.25,1.63)	1.36(1.17,1.56)	1.047407149
Multiple sclerosis	Central Europe	1840.58(1648.33,2036.34)	-0.66(-0.81,-0.51)	1.70(1.52,1.88)	-0.12(-0.18,-0.06)	1.38(1.23,1.54)	2.04(1.82,2.25)	0.679354443
Multiple sclerosis	Central Latin America	1041.93(855.94,1222.14)	2.84(2.63,3.04)	0.40(0.33,0.47)	0.72(0.61,0.82)	0.28(0.23,0.33)	0.52(0.43,0.61)	0.539316706
Multiple sclerosis	Central sub-Saharan Africa	243.47(188.49,301.80)	3.39(3.32,3.46)	0.22(0.18,0.27)	0.11(0.06,0.16)	0.17(0.13,0.21)	0.28(0.22,0.34)	0.607352515

Multiple sclerosis	East Asia	3119.51(2554.89,3722.27)	1.41(1.36,1.46)	0.18(0.14,0.21)	-0.14(-0.23,-0.04)	0.16(0.14,0.19)	0.19(0.15,0.23)	0.870474315
Multiple sclerosis	Eastern Europe	2151.74(1832.34,2468.35)	-0.88(-1.02,-0.75)	1.05(0.89,1.22)	-0.52(-0.58,-0.46)	0.98(0.82,1.12)	1.13(0.96,1.31)	0.861843151
Multiple sclerosis	Eastern sub-Saharan Africa	876.22(680.74,1087.36)	3.01(2.91,3.10)	0.26(0.20,0.31)	0.00(-0.08,0.08)	0.19(0.15,0.24)	0.32(0.25,0.38)	0.617086326
Multiple sclerosis	High-income Asia Pacific	727.13(596.53,872.47)	0.31(0.26,0.36)	0.36(0.29,0.44)	0.12(0.09,0.16)	0.28(0.23,0.34)	0.44(0.36,0.54)	0.639005188
Multiple sclerosis	High-income North America	12057.75(11089.47,12990.11)	0.59(0.51,0.67)	3.57(3.29,3.84)	0.24(0.18,0.29)	2.23(2.05,2.40)	4.94(4.55,5.33)	0.451399516
Multiple sclerosis	North Africa and middle East	9217.90(7878.58,10525.49)	2.95(2.87,3.03)	1.39(1.20,1.58)	0.30(0.25,0.36)	1.02(0.88,1.17)	1.79(1.55,2.05)	0.570135021
Multiple sclerosis	Oceania	15.80(12.29,19.21)	2.67(2.65,2.69)	0.14(0.11,0.17)	-0.15(-0.16,-0.14)	0.12(0.10,0.14)	0.16(0.12,0.19)	0.776125249
Multiple sclerosis	South Asia	7446.55(5987.29,8964.68)	2.64(2.62,2.66)	0.40(0.33,0.48)	0.33(0.29,0.37)	0.32(0.26,0.38)	0.49(0.40,0.58)	0.6595536
Multiple sclerosis	Southeast Asia	1251.47(1010.26,1503.53)	1.90(1.85,1.95)	0.18(0.15,0.21)	-0.05(-0.06,-0.04)	0.16(0.13,0.19)	0.20(0.16,0.24)	0.800603997
Multiple sclerosis	Southern Latin America	656.96(550.24,760.04)	1.38(1.36,1.40)	0.94(0.79,1.10)	0.12(0.12,0.13)	0.82(0.68,0.95)	1.07(0.89,1.25)	0.764061745
Multiple sclerosis	Southern sub-Saharan Africa	302.24(239.86,365.26)	1.85(1.80,1.90)	0.38(0.31,0.46)	0.08(-0.02,0.19)	0.31(0.25,0.37)	0.45(0.37,0.54)	0.682221201
Multiple sclerosis	Tropical Latin America	1968.42(1637.50,2320.20)	2.32(2.21,2.42)	0.79(0.66,0.93)	0.25(0.16,0.34)	0.61(0.50,0.72)	0.98(0.82,1.14)	0.621187897
Multiple sclerosis	Western Europe	12497.31(11105.61,13902.64)	0.67(0.62,0.72)	3.18(2.81,3.55)	0.70(0.68,0.72)	2.20(1.94,2.46)	4.20(3.73,4.68)	0.523468514
Multiple sclerosis	Western sub-Saharan Africa	1549.38(1264.49,1846.06)	3.61(3.59,3.62)	0.41(0.34,0.48)	0.40(0.38,0.42)	0.25(0.20,0.29)	0.56(0.48,0.65)	0.437160639

Motor neuron disease	Andean Latin America	268.04(228.69,312.01)	2.68(2.61,2.76)	0.45(0.38,0.52)	0.32(0.27,0.37)	0.49(0.42,0.56)	0.41(0.35,0.48)	1.184817385
Motor neuron disease	Australasia	1108.53(1056.79,1154.97)	3.06(3.03,3.09)	2.47(2.36,2.58)	0.64(0.60,0.68)	3.03(2.89,3.16)	1.98(1.87,2.08)	1.530978432
Motor neuron disease	Caribbean	355.13(314.48,400.05)	2.04(1.99,2.08)	0.73(0.64,0.81)	0.41(0.36,0.45)	0.79(0.70,0.88)	0.67(0.59,0.75)	1.189156079
Motor neuron disease	Central Asia	431.58(355.63,525.64)	1.18(0.96,1.39)	0.49(0.41,0.59)	-0.08(-0.18,0.03)	0.50(0.42,0.61)	0.47(0.39,0.57)	1.070110981
Motor neuron disease	Central Europe	1098.89(968.04,1253.90)	1.07(0.95,1.18)	0.69(0.61,0.79)	0.37(0.30,0.44)	0.77(0.68,0.87)	0.63(0.55,0.71)	1.23732056
Motor neuron disease	Central Latin America	1414.86(1230.26,1621.14)	2.48(2.43,2.52)	0.59(0.51,0.67)	0.58(0.53,0.63)	0.65(0.57,0.74)	0.54(0.46,0.61)	1.210836706
Motor neuron disease	Central sub-Saharan Africa	539.21(437.93,660.28)	3.03(2.95,3.12)	0.64(0.53,0.81)	0.06(-0.04,0.17)	0.68(0.56,0.84)	0.61(0.50,0.77)	1.1124987
Motor neuron disease	East Asia	9432.66(7816.94,11699.79)	1.24(0.94,1.53)	0.54(0.46,0.65)	-0.42(-0.63,-0.21)	0.59(0.50,0.70)	0.49(0.41,0.59)	1.201826095
Motor neuron disease	Eastern Europe	1499.10(1284.01,1760.28)	0.76(0.58,0.94)	0.58(0.50,0.68)	0.43(0.33,0.53)	0.67(0.58,0.77)	0.51(0.43,0.60)	1.321206039
Motor neuron disease	Eastern sub-Saharan Africa	1735.36(1424.63,2131.85)	2.62(2.46,2.77)	0.66(0.55,0.83)	-0.08(-0.24,0.08)	0.68(0.56,0.84)	0.65(0.53,0.82)	1.046778459
Motor neuron disease	High-income Asia Pacific	3114.27(2840.71,3436.45)	2.51(2.46,2.57)	0.87(0.78,0.97)	0.29(0.26,0.33)	1.07(0.97,1.18)	0.69(0.61,0.78)	1.551445006
Motor neuron disease	High-income North America	11322.79(10817.90,11839.31)	2.53(2.46,2.60)	1.97(1.88,2.06)	0.52(0.49,0.56)	2.35(2.25,2.45)	1.64(1.55,1.72)	1.437826602
Motor neuron disease	North Africa and middle East	3409.38(2880.66,4056.06)	2.08(1.98,2.17)	0.62(0.53,0.73)	0.05(-0.03,0.13)	0.68(0.58,0.80)	0.56(0.47,0.66)	1.214894232

Motor neuron disease	Oceania	45.65(37.71,54.73)	2.65(2.54,2.77)	0.43(0.36,0.52)	-0.16(-0.28,-0.05)	0.46(0.38,0.54)	0.41(0.34,0.50)	1.117832189
Motor neuron disease	South Asia	6855.22(5615.30,8401.31)	1.93(1.76,2.10)	0.42(0.34,0.51)	-0.11(-0.29,0.08)	0.44(0.36,0.53)	0.39(0.32,0.48)	1.104258155
Motor neuron disease	Southeast Asia	2610.84(2121.40,3208.19)	1.95(1.79,2.11)	0.40(0.33,0.49)	-0.08(-0.26,0.09)	0.43(0.36,0.52)	0.37(0.31,0.45)	1.155955665
Motor neuron disease	Southern Latin America	734.86(664.39,806.44)	2.17(2.09,2.24)	0.96(0.87,1.06)	0.51(0.48,0.55)	1.12(1.01,1.23)	0.82(0.74,0.91)	1.356473105
Motor neuron disease	Southern sub-Saharan Africa	376.93(311.48,465.51)	1.80(1.59,2.01)	0.55(0.46,0.68)	0.06(-0.17,0.30)	0.57(0.47,0.70)	0.53(0.44,0.66)	1.064155156
Motor neuron disease	Tropical Latin America	1915.61(1705.55,2132.61)	3.36(3.27,3.45)	0.83(0.74,0.91)	1.17(1.11,1.22)	0.94(0.84,1.03)	0.73(0.64,0.81)	1.291477852
Motor neuron disease	Western Europe	13796.97(13037.65,14494.46)	2.09(2.05,2.14)	1.85(1.73,1.95)	0.66(0.62,0.70)	2.17(2.05,2.29)	1.55(1.45,1.65)	1.398706998
Motor neuron disease	Western sub-Saharan Africa	1634.14(1349.80,1970.13)	2.84(2.70,2.97)	0.51(0.42,0.63)	-0.06(-0.24,0.11)	0.54(0.45,0.66)	0.49(0.40,0.60)	1.1045657
Depressive disorders	Andean Latin America	1809801.79(1561707.15,2084373.40)	2.08(2.03,2.14)	2886.58(2499.39,3315.19)	-0.29(-0.34,-0.25)	2054.55(1770.45,2370.85)	3700.41(3190.58,4308.88)	0.555222162
Depressive disorders	Australasia	1539865.79(1329847.71,1771751.73)	1.30(1.15,1.45)	5079.18(4368.05,5925.80)	0.07(-0.06,0.21)	4048.78(3434.86,4728.67)	6106.23(5138.58,7143.98)	0.66305774
Depressive disorders	Caribbean	2156061.61(1859121.26,2484561.49)	0.95(0.92,0.99)	4336.17(3737.42,5007.13)	-0.51(-0.56,-0.45)	3076.94(2681.65,3541.95)	5548.26(4735.01,6457.54)	0.554576903
Depressive disorders	Central Asia	2980970.32(2577905.72,3464935.22)	1.35(1.29,1.41)	3327.42(2888.66,3825.36)	-0.19(-0.21,-0.16)	2378.82(2068.60,2743.67)	4150.25(3588.37,4786.17)	0.57317381
Depressive disorders	Central Europe	3557073.74(3134283.35,4043450.63)	-0.36(-0.43,-0.29)	2436.80(2132.45,2771.69)	-0.67(-0.74,-0.59)	1620.71(1430.04,1832.40)	3205.85(2781.52,3682.09)	0.505546599

Depressive disorders	Central Latin America	9412732.40(8221931.89,10719231.14)	2.67(2.57,2.77)	3675.78(3219.65,4181.96)	0.34(0.30,0.37)	2602.25(2291.45,2936.03)	4678.39(4035.21,5379.91)	0.556228247
Depressive disorders	Central sub-Saharan Africa	6714338.60(5590521.19,8062124.20)	3.02(2.98,3.06)	6646.94(5680.50,7819.84)	-0.17(-0.18,-0.15)	5652.53(4803.26,6652.23)	7602.29(6479.75,8941.38)	0.743529322
Depressive disorders	East Asia	42235925.65(37513978.83,47555038.18)	0.66(0.50,0.82)	2292.26(2043.67,2562.45)	-0.80(-0.97,-0.64)	1676.14(1492.76,1874.77)	2914.77(2588.17,3265.43)	0.575049218
Depressive disorders	Eastern Europe	9150637.27(7960748.76,10432657.56)	-0.50(-0.63,-0.38)	3546.80(3076.08,4062.82)	-0.57(-0.67,-0.47)	2935.39(2539.10,3365.64)	4089.62(3552.19,4695.28)	0.7177656
Depressive disorders	Eastern sub-Saharan Africa	16013047.42(13725943.72,18554112.67)	2.55(2.47,2.62)	5466.48(4781.02,6234.48)	-0.32(-0.39,-0.25)	4686.98(4100.00,5331.37)	6207.76(5417.65,7093.82)	0.755018615
Depressive disorders	High-income Asia Pacific	5193651.72(4652117.81,5735089.94)	0.95(0.77,1.13)	2320.99(2063.54,2600.80)	0.40(0.26,0.54)	1849.16(1633.04,2075.60)	2795.52(2483.18,3144.89)	0.661472095
Depressive disorders	High-income North America	18459875.73(16429393.26,20674356.56)	1.45(1.12,1.79)	4885.16(4308.48,5532.44)	0.62(0.31,0.94)	3437.52(3028.74,3908.49)	6334.27(5578.92,7179.34)	0.542687048
Depressive disorders	North Africa and middle East	31006694.64(26270019.48,36438429.27)	2.70(2.65,2.74)	5098.60(4378.86,5947.72)	0.06(0.03,0.09)	3905.33(3360.30,4535.30)	6393.39(5436.80,7471.78)	0.610839144
Depressive disorders	Oceania	328505.19(274947.17,393381.41)	2.57(2.56,2.58)	2711.59(2306.26,3193.17)	-0.16(-0.17,-0.15)	2392.38(2014.90,2846.97)	3050.36(2605.87,3600.40)	0.784293752
Depressive disorders	South Asia	71998403.39(62917271.37,81675123.37)	1.49(1.22,1.77)	4179.15(3668.72,4727.18)	-0.85(-1.11,-0.59)	3393.85(2980.80,3829.78)	4979.09(4361.93,5649.83)	0.681621042
Depressive disorders	Southeast Asia	14451056.29(12506179.66,16471186.03)	1.62(1.60,1.64)	2060.52(1797.73,2341.00)	-0.19(-0.25,-0.13)	1741.48(1511.53,1975.91)	2370.57(2072.52,2704.78)	0.734627283
Depressive disorders	Southern Latin America	2362145.63(2089296.57,2658887.42)	0.93(0.85,1.02)	3313.55(2925.62,3745.45)	-0.42(-0.50,-0.34)	2286.50(2007.23,2607.64)	4297.25(3780.89,4874.06)	0.532085704
Depressive disorders	Southern sub-Saharan Africa	3344012.24(2915269.39,3791826.34)	2.01(1.94,2.07)	4552.32(4015.91,5105.97)	0.13(0.02,0.25)	3429.98(3027.85,3869.59)	5520.75(4849.58,6201.20)	0.621288788

Depressive disorders	Tropical Latin America	10928341.80(9746995.22,12123339.82)	1.62(1.22,2.02)	4560.16(4084.43,5058.00)	-0.32(-0.63,0.00)	2879.85(2562.60,3220.57)	6148.91(5489.16,6819.12)	0.46835057
Depressive disorders	Western Europe	22312186.03(19873592.10,25015077.17)	0.51(0.47,0.55)	4347.46(3841.95,4912.74)	-0.11(-0.14,-0.08)	3081.89(2715.29,3481.52)	5610.84(4955.09,6364.00)	0.549273855
Depressive disorders	Western sub-Saharan Africa	14230414.33(12217180.97,16431647.66)	2.77(2.53,3.00)	4407.30(3851.35,5021.82)	-0.25(-0.44,-0.06)	3405.82(3002.54,3873.80)	5324.47(4653.62,6080.93)	0.639655368
Bipolar disorder	Andean Latin America	48821.54(38258.98,60908.12)	1.67(1.63,1.72)	75.17(59.13,92.98)	0.00(-0.01,0.00)	71.80(56.00,89.46)	78.78(61.96,97.84)	0.911350202
Bipolar disorder	Australasia	24510.38(20496.79,28724.32)	1.09(1.04,1.15)	92.96(76.78,110.03)	0.09(0.07,0.11)	100.17(80.94,121.01)	85.37(71.47,99.95)	1.173395882
Bipolar disorder	Caribbean	36114.76(28634.59,44533.20)	0.94(0.87,1.02)	76.05(59.57,94.33)	0.02(0.02,0.02)	72.40(56.88,90.02)	79.77(62.26,98.80)	0.907622377
Bipolar disorder	Central Asia	42221.69(33357.87,52657.68)	1.15(1.08,1.23)	45.28(35.92,56.06)	0.00(0.00,0.00)	44.76(35.39,55.64)	45.85(36.21,56.77)	0.976086028
Bipolar disorder	Central Europe	51353.31(42169.85,61958.76)	-0.52(-0.59,-0.44)	45.91(37.76,55.31)	0.00(-0.01,0.00)	44.65(36.59,53.72)	47.27(38.77,57.08)	0.944420249
Bipolar disorder	Central Latin America	184820.84(152899.13,220904.69)	1.41(1.32,1.49)	71.31(58.94,85.11)	-0.01(-0.01,-0.01)	68.18(56.30,81.50)	74.57(61.58,88.77)	0.914324909
Bipolar disorder	Central sub-Saharan Africa	69070.31(52512.01,88680.17)	3.21(3.21,3.22)	53.81(42.48,67.36)	0.00(0.00,0.00)	53.53(42.46,67.14)	54.09(42.55,67.49)	0.989541848
Bipolar disorder	East Asia	248787.35(208824.39,293120.67)	0.76(0.63,0.89)	16.20(13.68,18.85)	-0.01(-0.02,0.00)	16.21(13.64,18.84)	16.26(13.79,18.98)	0.996768975
Bipolar disorder	Eastern Europe	97229.86(81863.81,115235.13)	-0.49(-0.62,-0.36)	46.91(39.38,55.51)	0.00(0.00,0.00)	47.12(39.40,55.83)	46.80(39.41,55.22)	1.006938873
Bipolar disorder	Eastern sub-Saharan Africa	239589.62(189979.47,299136.03)	2.89(2.87,2.91)	58.52(47.75,71.03)	0.00(0.00,0.00)	57.91(47.43,70.65)	59.14(48.33,71.99)	0.979218278

Bipolar disorder	High-income Asia Pacific	86654.15(72327.86,104045.85)	-0.06(-0.08,-0.03)	48.62(40.52,57.55)	0.01(-0.02,0.04)	49.88(41.01,58.90)	47.30(39.55,55.89)	1.054588919
Bipolar disorder	High-income North America	181413.88(163796.97,198473.30)	0.91(0.84,0.98)	53.23(48.54,58.07)	0.03(0.02,0.03)	58.28(52.89,63.81)	47.93(43.46,52.44)	1.215964085
Bipolar disorder	North Africa and middle East	400594.22(318033.90,494966.53)	1.94(1.84,2.04)	64.00(51.04,78.88)	-0.01(-0.01,0.00)	59.92(47.95,73.86)	68.35(54.33,84.59)	0.876684972
Bipolar disorder	Oceania	3425.74(2671.80,4396.08)	2.66(2.62,2.69)	27.06(21.40,34.00)	0.01(0.01,0.01)	27.14(21.46,34.12)	26.95(21.22,33.89)	1.006925076
Bipolar disorder	South Asia	652810.17(547713.18,774133.07)	2.11(2.07,2.15)	34.91(29.26,41.11)	0.00(-0.01,0.00)	36.60(30.68,43.17)	33.13(27.79,39.02)	1.104602273
Bipolar disorder	Southeast Asia	217013.39(178813.90,259904.21)	1.46(1.40,1.52)	30.98(25.56,37.13)	0.00(0.00,0.00)	31.31(25.89,37.37)	30.73(25.33,36.72)	1.0187897
Bipolar disorder	Southern Latin America	52957.87(42492.99,64919.17)	1.24(1.15,1.33)	80.48(63.41,99.02)	0.31(0.25,0.36)	83.94(64.91,104.46)	76.82(60.84,94.65)	1.092752916
Bipolar disorder	Southern sub-Saharan Africa	44806.32(37119.18,54024.44)	1.34(1.20,1.47)	56.02(46.42,66.97)	0.00(0.00,0.00)	55.86(46.34,66.68)	56.22(46.71,67.37)	0.993603052
Bipolar disorder	Tropical Latin America	210371.52(177558.39,247453.89)	1.03(0.93,1.13)	93.53(78.51,110.55)	0.00(0.00,0.00)	90.26(75.52,106.97)	96.91(81.30,113.82)	0.931347256
Bipolar disorder	Western Europe	264358.77(217954.85,315334.42)	0.40(0.37,0.43)	63.75(52.45,75.48)	0.08(0.07,0.10)	56.27(46.12,67.03)	71.49(59.06,84.78)	0.787105158
Bipolar disorder	Western sub-Saharan Africa	231880.47(184339.68,286818.32)	3.18(3.16,3.19)	52.27(42.76,63.27)	0.00(0.00,0.00)	51.78(42.47,62.58)	52.73(43.16,63.84)	0.982036042
Anxiety disorders	Andean Latin America	509032.89(392207.07,650419.67)	1.87(1.84,1.89)	787.71(610.28,1006.92)	0.05(0.04,0.06)	678.24(532.29,854.26)	899.82(691.66,1163.30)	0.753757514
Anxiety disorders	Australasia	229065.57(180508.98,290351.13)	1.11(1.03,1.20)	848.68(661.32,1077.15)	0.14(0.07,0.20)	766.11(594.08,965.25)	932.62(721.84,1183.45)	0.821459005

Anxiety disorders	Caribbean	318495.53(249314.73,403204.23)	1.09(1.04,1.14)	669.28(520.30,846.20)	0.08(0.07,0.10)	557.33(438.86,699.44)	780.94(598.73,987.81)	0.713664392
Anxiety disorders	Central Asia	352797.54(273563.59,442324.78)	1.06(1.03,1.09)	371.71(290.94,464.99)	-0.05(-0.06,-0.04)	297.33(234.51,373.59)	446.26(345.18,557.73)	0.666262402
Anxiety disorders	Central Europe	574592.44(459075.54,701235.01)	-0.40(-0.43,-0.36)	506.31(403.70,624.14)	-0.01(-0.02,0.01)	398.74(319.37,491.15)	618.18(488.92,764.55)	0.645014259
Anxiety disorders	Central Latin America	1556826.46(1235585.57,1947173.59)	1.88(1.67,2.10)	609.11(484.47,757.86)	0.33(0.21,0.45)	492.56(397.84,605.36)	722.39(568.53,899.21)	0.681849552
Anxiety disorders	Central sub-Saharan Africa	791576.48(599126.11,1024026.24)	3.19(3.17,3.21)	604.33(465.78,768.91)	0.01(0.00,0.01)	548.22(423.78,694.93)	659.79(506.99,841.42)	0.830913663
Anxiety disorders	East Asia	7566860.96(6270421.21,9016514.60)	0.06(-0.09,0.22)	525.02(434.05,620.38)	-0.33(-0.41,-0.25)	427.96(357.35,505.42)	632.36(523.82,749.01)	0.676760033
Anxiety disorders	Eastern Europe	1052685.05(851907.11,1272747.61)	-0.44(-0.48,-0.41)	504.11(408.39,605.85)	-0.01(-0.04,0.02)	404.70(328.78,483.67)	602.50(488.31,728.32)	0.671705885
Anxiety disorders	Eastern sub-Saharan Africa	2340711.67(1784980.84,2961718.60)	2.89(2.86,2.92)	575.22(453.32,719.92)	0.01(-0.02,0.04)	502.61(399.63,625.89)	645.82(507.45,810.12)	0.778240301
Anxiety disorders	High-income Asia Pacific	740535.33(601239.35,894868.93)	-0.35(-0.45,-0.26)	433.60(351.23,528.98)	-0.26(-0.35,-0.16)	351.50(285.85,425.93)	520.24(418.82,641.06)	0.675664376
Anxiety disorders	High-income North America	2862836.73(2305277.03,3520031.51)	0.78(0.48,1.07)	806.33(643.52,987.22)	0.04(-0.22,0.30)	682.30(552.40,825.56)	933.00(734.38,1163.58)	0.731294903
Anxiety disorders	North Africa and middle East	4946197.06(3865717.07,6243494.11)	2.04(2.00,2.08)	783.08(615.84,980.97)	0.13(0.10,0.16)	657.27(523.69,816.05)	919.37(713.14,1151.20)	0.714918892
Anxiety disorders	Oceania	83783.46(64317.79,106607.32)	2.61(2.59,2.63)	626.04(488.65,786.47)	0.07(0.06,0.09)	516.40(403.50,650.85)	742.14(574.65,932.55)	0.695826028
Anxiety disorders	South Asia	9224066.41(7441436.58,11159430.02)	2.29(2.14,2.45)	497.90(403.59,596.56)	0.22(0.06,0.37)	430.01(350.92,512.97)	569.40(457.41,684.24)	0.755194236

Anxiety disorders	Southeast Asia	4040208.94(3239721.85, 4935644.04)	1.49(1.44,1.54)	578.70(464.32,710.45)	0.11(0.08,0.14)	454.64(370.01,553.83)	705.93(562.49,862.09)	0.644031689
Anxiety disorders	Southern Latin America	482374.55(398799.90,575568.28)	1.02(0.94,1.10)	730.02(604.80,871.88)	0.06(0.00,0.13)	570.22(466.35,674.58)	889.18(723.58,1076.32)	0.641288694
Anxiety disorders	Southern sub-Saharan Africa	464742.41(370130.24,570637.26)	1.38(1.26,1.50)	576.08(461.61,701.31)	0.01(-0.02,0.03)	507.06(410.75,618.60)	642.87(512.63,785.17)	0.788748644
Anxiety disorders	Tropical Latin America	2294069.87(1830909.17, 2807169.58)	1.92(1.60,2.23)	989.45(791.17,1208.28)	0.51(0.27,0.76)	825.38(668.15,988.11)	1155.38(915.77,1427.78)	0.714376358
Anxiety disorders	Western Europe	3087775.04(2492302.51, 3778631.70)	0.35(0.30,0.39)	791.24(629.18,977.02)	0.09(0.06,0.12)	642.94(516.34,790.63)	946.60(742.46,1175.23)	0.679207232
Anxiety disorders	Western sub-Saharan Africa	2301851.18(1805589.62, 2883921.58)	3.24(3.20,3.28)	499.15(401.76,612.78)	0.09(0.04,0.14)	451.54(363.33,554.96)	545.01(437.56,665.79)	0.828485411
Headache disorders	Andean Latin America	5492725.09(4844554.66, 6150383.48)	1.83(1.81,1.86)	8605.28(7604.98,9622.17)	0.01(0.01,0.02)	7846.05(6878.48,8824.58)	9373.75(8305.32,10445.17)	0.83702429
Headache disorders	Australasia	3130799.52(2812908.67, 3484955.11)	1.20(1.18,1.22)	10596.77(9446.71,11809.01)	0.00(0.00,0.00)	9727.75(8594.30,10879.24)	11462.80(10254.36, 12787.44)	0.848636738
Headache disorders	Caribbean	4614061.08(4099342.64, 5138643.14)	1.05(0.99,1.11)	9653.09(8551.90,10729.96)	0.00(0.00,0.00)	9120.43(8054.82,10177.81)	10180.16(9064.34,11284.11)	0.895902377
Headache disorders	Central Asia	11149140.95(9856635.82,12463753.88)	1.11(1.09,1.12)	11863.32(10502.98,13191.56)	0.00(0.00,0.00)	11400.83(10075.77, 12730.38)	12321.44(10959.94, 13703.51)	0.925284205
Headache disorders	Central Europe	13939951.55(12502216.42,15449011.30)	-0.29(-0.32,-0.25)	11879.26(10615.20,13170.33)	0.00(0.00,0.00)	11115.79(9910.21,12316.20)	12651.64(11307.52, 14034.07)	0.878605005
Headache disorders	Central Latin America	25317406.01(22511718.59,28114379.73)	1.61(1.53,1.69)	9938.46(8863.37,11017.65)	0.00(0.00,0.00)	9350.71(8299.09,10437.06)	10511.70(9408.13,11615.47)	0.889552888
Headache disorders	Central sub-Saharan Africa	11697095.06(10159050.10,13190458.47)	3.19(3.18,3.20)	9480.93(8395.22,10616.22)	0.00(0.00,0.00)	9232.48(8141.49,10359.19)	9725.61(8656.04,10867.60)	0.94929568

Headache disorders	East Asia	118202777.40(105953749.20,131350815.10)	0.85(0.74,0.96)	7780.02(6979.70,8649.08)	0.19(0.14,0.24)	6985.36(6204.98,7840.60)	8616.67(7774.01,9531.10)	0.810679153
Headache disorders	Eastern Europe	26759786.44(23987209.19,29703410.15)	-0.40(-0.45,-0.35)	12558.90(11198.47,13936.36)	-0.01(-0.02,0.00)	12144.31(10782.96,13510.01)	12964.01(11626.71,14387.87)	0.936771069
Headache disorders	Eastern sub-Saharan Africa	31068446.39(27074916.82,35205550.82)	2.82(2.80,2.84)	8005.25(7104.68,8955.80)	-0.07(-0.09,-0.06)	7891.94(6989.26,8875.07)	8117.99(7231.48,9073.78)	0.97215444
Headache disorders	High-income Asia Pacific	21896297.75(19675338.80,24343215.20)	0.18(0.16,0.20)	11418.29(10200.40,12666.31)	-0.01(-0.03,0.00)	10459.95(9315.29,11653.00)	12411.98(11087.81,13711.31)	0.842730139
Headache disorders	High-income North America	48233358.67(43410307.27,53334264.12)	0.85(0.83,0.86)	13008.35(11649.62,14384.03)	-0.04(-0.05,-0.03)	11997.87(10727.15,13344.70)	14024.23(12561.67,15523.20)	0.855510319
Headache disorders	North Africa and middle East	60830664.58(53891297.86,67676102.09)	2.16(2.12,2.21)	9918.96(8853.09,10989.52)	0.00(-0.01,0.02)	9572.46(8510.64,10647.65)	10285.01(9209.29,11397.67)	0.930719258
Headache disorders	Oceania	1190000.27(1043814.72,1335717.67)	2.58(2.56,2.60)	9289.59(8247.94,10334.21)	0.00(0.00,0.00)	8741.86(7698.45,9761.03)	9869.45(8775.42,10964.09)	0.885748957
Headache disorders	South Asia	199122873.70(177628004.40,221237218.80)	1.95(1.93,1.97)	10782.51(9709.01,11924.42)	-0.05(-0.07,-0.03)	10626.46(9549.20,11778.21)	10950.61(9865.07,12105.51)	0.970399505
Headache disorders	Southeast Asia	71934112.96(64370874.87,79911095.70)	1.38(1.32,1.44)	10434.82(9359.56,11575.58)	0.00(0.00,0.00)	9808.20(8761.90,10924.55)	11060.39(9913.39,12226.20)	0.88678659
Headache disorders	Southern Latin America	6966515.61(6204065.74,7768112.67)	1.15(1.13,1.17)	10184.37(9002.90,11388.94)	0.02(0.02,0.03)	9308.09(8140.84,10440.44)	11039.43(9772.26,12312.21)	0.843167565
Headache disorders	Southern sub-Saharan Africa	7854921.44(6978980.47,8784100.48)	1.44(1.34,1.54)	9874.90(8842.24,10966.46)	0.00(0.00,0.00)	9614.97(8530.38,10721.86)	10131.27(9081.36,11194.89)	0.949038446
Headache disorders	Tropical Latin America	26432990.08(23674060.19,29264779.09)	1.36(1.29,1.43)	11654.86(10452.75,12898.79)	0.07(0.05,0.08)	11217.09(10035.32,12455.29)	12088.50(10853.07,13398.16)	0.927913879
Headache disorders	Western Europe	54149385.95(48551566.91,59968570.24)	0.40(0.38,0.41)	12368.07(11046.77,13724.33)	0.02(0.02,0.03)	11658.97(10405.98,12988.70)	13091.26(11697.19,14471.07)	0.890592204

Headache disorders	Western sub-Saharan Africa	43855772.29(38535331.60,49016468.85)	3.12(3.11,3.14)	10305.19(9227.95,11419.46)	-0.02(-0.03,-0.01)	9978.91(8903.75,11059.89)	10616.99(9493.58,11758.85)	0.939899912

CI - confidence interval, EAPC - estimated annual percentage change, SDI - socio-demographic index, UI - uncertainty interval.

Table S6. The age-standardized DALY rate of other neurological disorders in 1990 and 2019 and its temporal trends, by regions

		Absolute numbers		Age-standardized rate				
	**Regions**	**2019(95% UI)**	**EAPC(95% CI) (1990-2019)**	**2019(95% UI)**	**EAPC(95% CI) (1990-2019)**	**males, 2019 (95% UI)**	**females, 2019(95% UI)**	**male-to-female ratio**
Alzheimer's disease and other dementias	Andean Latin America	163614.39(69801.85,362966.12)	4.28(4.23,4.32)	312.67(133.67, 691.68)	0.09(0.08,0.11)	303.85(126.20, 680.57)	320.04(138.14, 703.70)	0.949420107
Alzheimer's disease and other dementias	Australasia	183099.14(84033.60,390106.06)	3.34(3.26,3.41)	319.88(146.31, 681.98)	-0.09(-0.11,-0.07)	277.03(120.30, 606.43)	350.26(166.12, 723.53)	0.790912517
Alzheimer's disease and other dementias	Caribbean	155718.35(65947.18,342864.22)	2.97(2.90,3.03)	299.45(126.93, 661.71)	0.06(0.05,0.07)	294.73(122.29, 660.71)	303.11(129.59, 658.28)	0.97232672
Alzheimer's disease and other dementias	Central Asia	157279.19(70959.49,351464.69)	0.77(0.56,0.98)	345.47(155.28, 775.08)	0.19(0.16,0.21)	331.47(144.24, 772.10)	354.18(162.03, 780.78)	0.93588558
Alzheimer's disease and other dementias	Central Europe	794201.30(366490.15,1690233.70)	2.33(2.21,2.44)	348.75(160.87, 734.47)	-0.04(-0.05,-0.02)	321.03(141.81, 696.97)	366.07(172.54, 766.80)	0.876957744
Alzheimer's disease and other dementias	Central Latin America	728751.10(299639.48,1628645.90)	4.46(4.43,4.49)	333.23(136.24, 747.25)	0.03(0.01,0.05)	326.84(129.72, 741.14)	338.07(141.32, 745.77)	0.966802492
Alzheimer's disease and other dementias	Central sub-Saharan Africa	112381.78(48663.48,258388.28)	3.74(3.70,3.78)	380.57(165.07, 857.23)	0.35(0.33,0.37)	334.68(136.41, 801.28)	407.06(180.76, 895.00)	0.822188292
Alzheimer's disease and other dementias	East Asia	6201122.90(2778733.83,13575026.43)	4.21(4.16,4.27)	366.61(164.75, 789.10)	0.25(0.21,0.30)	317.73(139.52, 734.18)	398.25(183.78, 858.17)	0.79782708
Alzheimer's disease and other dementias	Eastern Europe	1141396.94(525883.20, 2486210.36)	1.86(1.68,2.04)	324.80(149.89, 705.23)	0.23(0.21,0.25)	304.80(137.61, 655.31)	334.41(155.11, 722.42)	0.911476561
Alzheimer's disease and other dementias	Eastern sub-Saharan Africa	354144.15(146346.70,817435.43)	3.39(3.31,3.47)	356.00(144.97, 829.93)	0.40(0.37,0.43)	311.92(122.38, 750.65)	387.62(161.73, 878.76)	0.804691808
Alzheimer's disease and other dementias	High-income Asia Pacific	2370758.88(1102292.18,4847729.51)	5.52(5.33,5.70)	385.38(179.30, 791.75)	0.81(0.69,0.93)	344.96(147.37, 766.77)	408.13(195.63, 799.74)	0.845231323

Alzheimer's disease and other dementias	High-income North America	2270344.77(1096609.81,4697147.85)	1.94(1.87,2.01)	317.66(154.02, 653.15)	-0.22(-0.27,-0.17)	288.89(140.08, 607.28)	335.17(162.72, 683.65)	0.861908681
Alzheimer's disease and other dementias	North Africa and middle East	1208063.92(532026.21, 2672826.01)	3.65(3.62,3.67)	386.96(171.95, 848.51)	-0.03(-0.04,-0.01)	365.12(161.68, 793.39)	408.67(180.27, 887.07)	0.893432006
Alzheimer's disease and other dementias	Oceania	12667.96(5280.75,29629.53)	2.94(2.90,2.97)	318.77(133.77, 726.91)	-0.18(-0.21,-0.14)	289.04(119.41, 667.54)	344.74(145.27, 777.47)	0.838412636
Alzheimer's disease and other dementias	South Asia	2617557.68(1061033.00,6225230.12)	4.58(4.33,4.83)	262.09(105.55, 617.38)	0.32(0.24,0.40)	253.43(100.61, 619.98)	269.85(107.74, 624.72)	0.939174414
Alzheimer's disease and other dementias	Southeast Asia	1551369.32(634692.96, 3426745.95)	3.46(3.43,3.50)	342.18(141.51, 765.11)	0.09(0.07,0.10)	291.04(117.08, 674.73)	376.01(156.89, 819.31)	0.774033799
Alzheimer's disease and other dementias	Southern Latin America	271092.70(123668.74,586533.94)	3.00(2.91,3.10)	311.86(142.12, 674.17)	0.13(0.11,0.14)	284.35(127.66, 636.49)	327.29(150.87, 699.26)	0.868809222
Alzheimer's disease and other dementias	Southern sub-Saharan Africa	130478.25(56315.53,298140.34)	2.32(2.24,2.40)	323.81(138.29, 740.97)	0.10(0.06,0.13)	287.16(117.70, 684.96)	342.53(148.80, 776.35)	0.838355312
Alzheimer's disease and other dementias	Tropical Latin America	873878.22(384944.32,1910951.23)	4.57(4.46,4.68)	390.88(172.01, 855.99)	0.11(0.07,0.15)	379.15(162.36, 849.72)	399.25(178.44, 864.81)	0.949656709
Alzheimer's disease and other dementias	Western Europe	3582943.93(1636179.06,7579612.38)	2.30(2.27,2.33)	309.71(141.53, 663.69)	-0.08(-0.11,-0.06)	272.52(120.13, 599.24)	332.74(155.00, 699.03)	0.819019803
Alzheimer's disease and other dementias	Western sub-Saharan Africa	396124.01(155275.18,929087.76)	2.98(2.92,3.04)	333.53(129.48, 774.76)	0.35(0.30,0.41)	303.75(115.15, 746.38)	358.96(139.21, 821.09)	0.846206811
Anxiety disorders	Andean Latin America	337478.04(224359.52,474258.54)	2.22(2.19,2.24)	527.34(351.37, 741.83)	0.09(0.07,0.11)	394.14(263.12, 557.20)	658.54(432.59, 925.39)	0.598501058
Anxiety disorders	Australasia	170387.35(114144.69,236834.74)	1.30(1.20,1.40)	575.65(383.79, 805.72)	0.13(0.04,0.22)	444.69(300.52, 633.57)	703.66(466.64, 984.37)	0.631966832
Anxiety disorders	Caribbean	204302.96(135496.04,287399.35)	1.32(1.28,1.36)	419.78(279.15, 587.13)	0.10(0.08,0.12)	312.22(209.55, 442.56)	524.07(343.05, 736.51)	0.595754912

Anxiety disorders	Central Asia	198488.13(133283.38,280515.99)	1.21(1.20,1.22)	212.77(142.78,298.07)	-0.06(-0.07,-0.06)	162.16(108.55,227.14)	260.51(174.05,366.29)	0.622471245
Anxiety disorders	Central Europe	397537.62(270194.36,551748.98)	-0.09(-0.13,-0.05)	313.31(212.34,435.32)	0.00(-0.01,0.00)	226.38(154.35,316.06)	398.77(270.82,551.09)	0.567694327
Anxiety disorders	Central Latin America	967646.83(658711.32,1344482.74)	2.21(1.96,2.45)	376.21(258.09,521.35)	0.37(0.20,0.53)	280.51(192.22,390.49)	466.22(317.53,643.18)	0.60166489
Anxiety disorders	Central sub-Saharan Africa	430841.70(282319.43,616899.21)	3.25(3.23,3.28)	366.32(244.62,516.87)	0.04(0.03,0.05)	310.85(206.32,440.03)	417.96(278.76,595.05)	0.743735976
Anxiety disorders	East Asia	4760419.40(3355574.18,6509793.64)	0.27(0.12,0.42)	307.61(215.22,421.59)	-0.47(-0.57,-0.37)	239.87(167.57,328.00)	379.30(266.14,520.61)	0.632396288
Anxiety disorders	Eastern Europe	689506.06(487799.87,934598.46)	-0.22(-0.25,-0.20)	304.66(214.57,414.36)	0.01(-0.03,0.04)	229.12(161.41,315.88)	372.88(261.33,507.63)	0.61447004
Anxiety disorders	Eastern sub-Saharan Africa	1292198.12(867010.28,1819179.63)	2.98(2.93,3.02)	353.88(241.11,487.70)	0.05(0.01,0.08)	288.99(195.62,401.95)	415.42(283.21,574.70)	0.695661847
Anxiety disorders	High-income Asia Pacific	488458.63(340172.56,668269.43)	-0.09(-0.18,0.00)	253.02(174.36,350.79)	-0.25(-0.33,-0.17)	195.22(135.18,269.64)	312.57(215.59,432.25)	0.624564583
Anxiety disorders	High-income North America	2034114.06(1427035.46,2752246.44)	0.81(0.46,1.16)	521.52(362.54,709.05)	-0.09(-0.40,0.23)	386.18(270.03,527.81)	654.68(454.94,888.15)	0.589873463
Anxiety disorders	North Africa and middle East	3068303.29(2070317.20,4310029.50)	2.41(2.34,2.48)	492.15(333.99,685.31)	0.20(0.16,0.23)	372.82(254.94,520.64)	621.57(416.67,869.07)	0.599795388
Anxiety disorders	Oceania	47668.36(31197.66,66823.94)	2.72(2.70,2.74)	380.21(253.59,531.82)	0.08(0.06,0.10)	290.85(195.95,410.45)	474.41(312.41,665.55)	0.613076999
Anxiety disorders	South Asia	5176081.62(3627582.53,7057263.48)	2.54(2.36,2.72)	286.44(200.71,390.78)	0.26(0.09,0.42)	235.29(164.60,321.47)	339.16(238.63,462.33)	0.693761336
Anxiety disorders	Southeast Asia	2434692.39(1683692.58,3344955.62)	1.80(1.76,1.84)	347.56(242.08,475.99)	0.15(0.12,0.19)	251.27(174.34,343.42)	442.28(306.86,610.21)	0.568140247

Anxiety disorders	Southern Latin America	340789.65(239428.10,460412.64)	1.17(1.13,1.21)	491.15(343.49,662.28)	-0.03(-0.06,0.00)	322.64(225.59,440.23)	652.26(456.97,879.33)	0.494647211
Anxiety disorders	Southern sub-Saharan Africa	271586.61(187329.03,374012.90)	1.56(1.43,1.68)	345.37(241.16,472.97)	0.00(-0.03,0.03)	283.81(195.56,387.46)	400.45(278.79,550.65)	0.708729488
Anxiety disorders	Tropical Latin America	1682365.69(1168793.94,2299370.10)	2.76(2.27,3.26)	700.17(487.24,953.94)	0.97(0.54,1.41)	482.75(338.33,659.52)	907.39(630.86,1236.74)	0.532022991
Anxiety disorders	Western Europe	2447055.38(1661754.26,3374216.76)	0.59(0.52,0.66)	537.89(364.59,748.20)	0.14(0.09,0.20)	372.62(255.30,517.89)	704.39(473.98,981.41)	0.528990727
Anxiety disorders	Western sub-Saharan Africa	1236128.59(839325.17,1718232.68)	3.28(3.24,3.32)	293.35(201.14,405.93)	0.13(0.07,0.19)	255.31(174.35,352.15)	328.97(225.48,453.89)	0.776070593
Bipolar disorder	Andean Latin America	127710.51(74826.43,199337.65)	2.23(2.20,2.27)	197.94(115.96,306.56)	0.01(0.00,0.01)	186.62(111.15,289.18)	209.31(122.02,324.78)	0.891598033
Bipolar disorder	Australasia	77641.00(47808.31,118197.31)	1.27(1.24,1.30)	254.35(152.96,389.42)	0.03(0.02,0.05)	248.76(149.99,382.42)	259.05(157.89,388.77)	0.960287827
Bipolar disorder	Caribbean	96733.20(56404.71,149158.00)	1.22(1.16,1.28)	196.38(115.42,302.63)	-0.05(-0.05,-0.05)	184.76(108.48,285.76)	207.81(122.45,321.11)	0.889093862
Bipolar disorder	Central Asia	106581.95(62510.96,165213.54)	1.55(1.50,1.59)	111.40(65.59,171.77)	0.00(0.00,0.00)	107.26(62.89,166.69)	115.35(67.51,176.60)	0.929906935
Bipolar disorder	Central Europe	155093.93(94627.80,237088.05)	-0.04(-0.11,0.03)	120.51(72.51,186.14)	0.00(0.00,0.01)	112.33(67.40,172.00)	128.73(77.69,199.89)	0.87263891
Bipolar disorder	Central Latin America	482092.04(291331.44,741088.49)	1.96(1.87,2.04)	184.86(111.94,283.81)	-0.01(-0.01,0.00)	169.61(102.26,262.76)	199.45(121.12,305.21)	0.850380455
Bipolar disorder	Central sub-Saharan Africa	129407.57(74092.21,204848.18)	3.29(3.26,3.32)	118.62(69.94,185.76)	0.04(0.03,0.05)	119.03(70.24,188.53)	118.22(70.02,184.27)	1.006807357
Bipolar disorder	East Asia	686681.13(420130.01,1047733.25)	1.21(1.12,1.30)	39.68(24.36,61.13)	0.02(0.01,0.03)	38.45(23.59,59.42)	41.07(25.14,62.47)	0.936368835

Bipolar disorder	Eastern Europe	262919.96(163454.59,398624.16)	-0.08(-0.14,-0.02)	111.30(68.99,170.17)	0.02(0.02,0.03)	105.17(64.90,160.80)	116.87(72.18,177.95)	0.899869089
Bipolar disorder	Eastern sub-Saharan Africa	441365.92(263188.95,693340.68)	3.01(2.99,3.03)	128.14(76.90,196.91)	0.03(0.03,0.04)	128.72(77.11,197.91)	127.60(76.73,195.91)	1.008838029
Bipolar disorder	High-income Asia Pacific	278696.13(171624.16,423394.18)	0.31(0.25,0.37)	131.01(79.47,201.19)	-0.02(-0.06,0.01)	127.11(77.18,195.76)	134.94(82.14,209.78)	0.941963614
Bipolar disorder	High-income North America	510564.39(328797.21,744334.81)	0.97(0.93,1.01)	132.00(84.19,192.34)	-0.02(-0.03,-0.01)	130.17(82.88,190.59)	133.45(85.12,194.40)	0.975408944
Bipolar disorder	North Africa and middle East	1023455.69(605191.93,1590701.16)	2.60(2.53,2.68)	163.66(96.85,253.47)	-0.01(-0.02,-0.01)	150.72(88.92,235.58)	177.71(104.74,272.83)	0.848138528
Bipolar disorder	Oceania	6802.27(3964.60,10762.45)	2.81(2.79,2.83)	56.74(33.40,88.39)	-0.01(-0.01,-0.01)	56.88(33.72,89.01)	56.57(33.46,89.47)	1.00547648
Bipolar disorder	South Asia	1394579.08(863412.02,2135967.72)	2.38(2.36,2.40)	76.94(47.71,118.03)	0.02(0.02,0.03)	82.39(50.72,125.60)	71.31(44.59,109.81)	1.155478514
Bipolar disorder	Southeast Asia	511591.44(310876.18,786394.15)	1.90(1.83,1.97)	71.57(43.43,109.78)	0.02(0.02,0.02)	70.70(42.56,108.48)	72.59(43.84,111.65)	0.974018187
Bipolar disorder	Southern Latin America	157416.69(91683.08,243598.84)	1.63(1.56,1.70)	221.87(129.38,342.66)	0.30(0.24,0.35)	215.64(126.07,333.88)	227.32(132.48,352.45)	0.948615194
Bipolar disorder	Southern sub-Saharan Africa	92724.80(56737.32,143338.66)	1.75(1.66,1.83)	117.80(72.91,180.02)	-0.02(-0.03,0.00)	118.65(73.44,180.69)	116.94(72.08,178.70)	1.014596282
Bipolar disorder	Tropical Latin America	577591.99(354660.69,886314.04)	1.74(1.65,1.82)	239.39(146.69,365.52)	0.01(0.00,0.01)	221.03(135.74,338.79)	257.13(157.25,391.23)	0.859574982
Bipolar disorder	Western Europe	941872.37(580479.76,1423756.00)	0.54(0.52,0.57)	193.71(118.31,294.75)	0.06(0.06,0.07)	167.32(101.34,257.40)	220.40(135.12,332.41)	0.759153428
Bipolar disorder	Western sub-Saharan Africa	440904.91(264945.30,686972.27)	3.18(3.17,3.19)	117.83(71.49,180.19)	0.02(0.01,0.02)	118.68(71.46,182.61)	117.06(71.26,179.32)	1.013840418

Depressive disorders	Andean Latin America	290671.12(198566.67,403423.00)	2.15(2.10,2.21)	462.07(318.12,640.03)	-0.25(-0.29,-0.21)	344.79(237.09,481.09)	576.78(394.37,796.50)	0.59779468
Depressive disorders	Australasia	237563.99(164168.64,330310.86)	1.31(1.17,1.45)	777.82(538.62,1094.07)	0.07(-0.05,0.20)	624.48(428.88,867.22)	930.34(641.70,1308.18)	0.671232632
Depressive disorders	Caribbean	327024.55(226095.47,450595.32)	0.97(0.94,1.01)	657.19(454.07,905.93)	-0.49(-0.54,-0.43)	482.48(332.76,669.25)	825.40(565.34,1144.14)	0.584543273
Depressive disorders	Central Asia	486599.54(334517.83,679840.46)	1.42(1.36,1.48)	534.90(372.57,741.82)	-0.16(-0.18,-0.13)	402.62(277.71,565.05)	650.36(452.28,904.85)	0.619074735
Depressive disorders	Central Europe	596439.89(420305.32,816081.84)	-0.27(-0.32,-0.22)	413.89(290.54,572.31)	-0.54(-0.60,-0.48)	296.35(207.51,411.38)	525.17(368.24,722.06)	0.564293466
Depressive disorders	Central Latin America	1447181.28(1009407.59,1981151.34)	2.63(2.54,2.73)	563.62(392.72,771.17)	0.31(0.28,0.34)	414.14(289.40,569.02)	703.12(482.09,973.22)	0.589007362
Depressive disorders	Central sub-Saharan Africa	1010267.31(681633.16,1430654.67)	3.08(3.04,3.12)	1000.16(682.15,1397.69)	-0.12(-0.14,-0.11)	855.64(583.64,1202.36)	1138.92(779.86,1593.49)	0.751278042
Depressive disorders	East Asia	7802554.71(5472939.02,10767864.39)	0.90(0.80,1.00)	415.98(291.93,573.46)	-0.67(-0.79,-0.55)	311.41(217.77,427.74)	522.00(363.83,715.74)	0.596575702
Depressive disorders	Eastern Europe	1442694.80(1013990.03,1986604.87)	-0.41(-0.51,-0.30)	562.24(391.45,771.76)	-0.46(-0.54,-0.38)	471.83(327.72,647.26)	642.46(446.72,883.22)	0.734402478
Depressive disorders	Eastern sub-Saharan Africa	2510165.43(1702207.92,3475450.90)	2.63(2.57,2.69)	845.40(589.89,1154.93)	-0.26(-0.32,-0.21)	721.32(502.79,993.14)	963.24(671.82,1312.87)	0.748853353
Depressive disorders	High-income Asia Pacific	812254.75(572741.12,1104804.98)	0.82(0.66,0.99)	365.65(253.57,499.26)	0.31(0.19,0.44)	297.97(206.70,409.43)	434.11(303.57,589.81)	0.686383237
Depressive disorders	High-income North America	2864089.37(2023936.15,3872481.12)	1.27(1.01,1.52)	753.77(525.53,1023.69)	0.43(0.19,0.67)	541.89(380.73,742.23)	965.88(672.68,1310.29)	0.5610351
Depressive disorders	North Africa and middle East	4767774.48(3261469.78,6600676.87)	2.74(2.70,2.79)	781.06(535.18,1075.62)	0.06(0.03,0.08)	609.91(418.97,855.86)	966.87(666.72,1330.82)	0.630805586

Depressive disorders	Oceania	56577.30(38500.72,80206.17)	2.66(2.65,2.67)	476.09(325.58,663.22)	-0.13(-0.13,-0.12)	416.63(285.66,580.13)	539.22(367.28,760.46)	0.772643092
Depressive disorders	South Asia	11188434.70(7828808.09,15283075.98)	1.65(1.41,1.89)	645.08(452.66,877.70)	-0.71(-0.93,-0.49)	532.85(374.00,725.68)	759.56(533.26,1040.44)	0.701531494
Depressive disorders	Southeast Asia	2753222.83(1898460.50,3795436.65)	1.83(1.81,1.85)	389.23(270.38,536.55)	-0.13(-0.17,-0.08)	326.25(225.56,448.02)	450.26(311.74,623.32)	0.724575928
Depressive disorders	Southern Latin America	359571.00(249694.99,491681.33)	0.94(0.86,1.03)	503.29(349.65,690.90)	-0.42(-0.50,-0.34)	358.04(248.63,494.29)	642.63(443.40,876.79)	0.557149477
Depressive disorders	Southern sub-Saharan Africa	524603.62(368831.47,719717.21)	1.99(1.93,2.04)	705.61(497.87,958.57)	0.09(-0.02,0.19)	547.44(384.92,748.39)	843.18(594.32,1140.94)	0.649252373
Depressive disorders	Tropical Latin America	1652267.21(1159774.23,2244114.27)	1.69(1.32,2.06)	686.08(482.44,932.46)	-0.27(-0.55,0.02)	452.68(315.71,619.34)	906.89(633.81,1222.55)	0.499153401
Depressive disorders	Western Europe	3463005.19(2438348.93,4706016.95)	0.52(0.48,0.56)	677.20(475.01,929.50)	-0.09(-0.11,-0.06)	501.13(350.51,685.75)	853.56(598.73,1174.69)	0.587110526
Depressive disorders	Western sub-Saharan Africa	2270679.03(1552644.99,3123064.55)	2.85(2.64,3.05)	693.84(485.18,949.29)	-0.20(-0.36,-0.03)	547.72(383.63,749.45)	827.52(578.33,1131.14)	0.661889376
Headache disorders	Andean Latin America	280976.69(66631.02,592407.43)	2.33(2.25,2.41)	437.24(105.95,919.18)	0.23(0.17,0.28)	296.73(80.62,615.17)	576.11(131.41,1214.73)	0.515046284
Headache disorders	Australasia	170319.88(41863.93,358273.08)	1.25(1.24,1.26)	555.86(124.21,1186.74)	0.01(0.01,0.01)	422.66(101.56,893.24)	686.38(146.75,1472.97)	0.615772401
Headache disorders	Caribbean	268116.13(53671.17,587009.01)	1.19(1.12,1.25)	550.75(107.75,1205.99)	-0.01(-0.01,-0.01)	388.04(82.08,831.82)	708.78(132.70,1590.52)	0.547478153
Headache disorders	Central Asia	541349.33(126731.40,1150969.24)	1.43(1.41,1.46)	566.74(132.90,1199.17)	-0.01(-0.01,-0.01)	416.91(104.71,868.17)	711.88(157.67,1508.39)	0.585651791
Headache disorders	Central Europe	749935.69(229391.07,1534260.44)	-0.09(-0.14,-0.04)	589.95(162.56,1234.07)	0.00(0.00,0.01)	427.02(126.02,872.91)	754.39(199.91,1600.28)	0.566048439

Headache disorders	Central Latin America	1472997.82(296392.59,3234169.62)	1.93(1.84,2.01)	568.52(114.75,1242.35)	0.07(0.06,0.08)	380.16(84.62,808.59)	745.18(140.27,1647.49)	0.510163241
Headache disorders	Central sub-Saharan Africa	587025.58(118106.92,1278361.61)	3.27(3.25,3.30)	517.06(121.14,1104.20)	0.03(0.02,0.03)	412.07(98.18,872.45)	618.05(142.38,1310.51)	0.666724568
Headache disorders	East Asia	8092831.13(1755996.69,17507399.42)	1.31(1.20,1.42)	479.17(96.55,1035.66)	0.24(0.19,0.29)	370.31(96.47,781.71)	593.02(94.07,1347.31)	0.624452729
Headache disorders	Eastern Europe	1589000.79(615470.52,3057267.79)	-0.03(-0.11,0.05)	669.66(239.06,1338.49)	0.09(0.06,0.12)	468.21(161.65,925.38)	857.42(303.80,1688.27)	0.546068969
Headache disorders	Eastern sub-Saharan Africa	1383404.84(348275.10,2884758.54)	3.02(2.99,3.05)	395.80(115.24,812.19)	0.06(0.04,0.07)	307.82(90.40,624.49)	479.90(137.89,992.78)	0.641440262
Headache disorders	High-income Asia Pacific	953891.96(287325.89,1922360.68)	0.21(0.18,0.24)	473.36(123.92,998.38)	0.01(-0.01,0.03)	347.99(107.94,702.17)	604.33(136.47,1313.08)	0.57583693
Headache disorders	High-income North America	2591453.22(558206.10,5583432.92)	0.84(0.79,0.89)	677.97(133.38,1469.21)	-0.06(-0.14,0.03)	440.30(106.46,917.31)	914.64(157.69,2040.66)	0.48138906
Headache disorders	North Africa and middle East	4209802.26(990143.24,9068318.87)	2.49(2.42,2.55)	669.58(159.07,1431.31)	0.00(-0.01,0.01)	511.51(133.11,1090.75)	842.49(189.81,1881.40)	0.607143698
Headache disorders	Oceania	69058.69(11168.93,155720.49)	2.76(2.73,2.78)	550.18(96.84,1226.37)	0.01(0.00,0.01)	439.15(86.95,946.49)	667.05(106.81,1526.76)	0.658343214
Headache disorders	South Asia	10710037.01(1765830.35,23536840.48)	2.14(2.12,2.17)	578.01(100.51,1258.73)	-0.04(-0.06,-0.02)	462.31(81.84,1010.77)	697.58(117.65,1519.35)	0.662737434
Headache disorders	Southeast Asia	4614961.55(736960.04,10622033.08)	1.64(1.58,1.70)	645.27(102.39,1488.70)	-0.05(-0.07,-0.04)	504.92(92.73,1108.03)	784.83(111.07,1878.29)	0.64335561
Headache disorders	Southern Latin America	346932.30(92464.66,717727.09)	1.45(1.42,1.49)	492.14(125.92,1020.05)	0.16(0.13,0.18)	352.24(101.49,713.49)	627.21(148.68,1326.76)	0.561601561
Headache disorders	Southern sub-Saharan Africa	424587.40(98067.78,896953.40)	1.66(1.57,1.75)	536.59(129.42,1119.16)	-0.03(-0.03,-0.02)	429.29(103.70,895.38)	636.71(149.85,1333.99)	0.674235025

Headache disorders	Tropical Latin America	1697910.16(263646.53, 3854242.80)	1.63(1.50,1.76)	728.48(106.33, 1669.18)	0.19(0.14,0.24)	517.00(78.03,1 179.64)	933.20(132.35, 2175.76)	0.55401007 4
Headache disorders	Western Europe	3400943.75(741015.64, 7295135.87)	0.48(0.43,0.53)	748.83(143.06, 1637.91)	0.09(0.07,0.12)	501.37(90.71,1 093.30)	999.76(192.33, 2209.11)	0.50148504 6
Headache disorders	Western sub-Saharan Africa	2463818.57(432157.68, 5449728.11)	3.20(3.19,3.20)	619.50(128.99, 1333.61)	0.04(0.03,0.05)	496.45(104.22, 1061.06)	732.65(149.59, 1604.84)	0.67760475 2
Idiopathic epilepsy	Andean Latin America	137191.19(81656.99,21 1207.58)	-0.04(-0.16,0.08)	215.59(127.52, 333.10)	-1.73(-1.83,- 1.62)	229.28(138.99, 346.81)	201.88(114.96, 316.10)	1.13572293 4
Idiopathic epilepsy	Australasia	34407.39(19820.56,576 17.80)	0.64(0.60,0.68)	115.85(66.24,1 97.65)	-0.64(-0.69,- 0.58)	123.90(74.99,2 06.40)	108.17(57.62,1 89.30)	1.14543906 2
Idiopathic epilepsy	Caribbean	107254.03(75784.66,14 5525.44)	0.34(0.19,0.49)	228.86(160.15, 310.29)	-0.56(-0.74,- 0.38)	251.96(178.37, 333.55)	206.48(138.33, 292.22)	1.22023105 9
Idiopathic epilepsy	Central Asia	274641.23(207185.69,3 65142.95)	0.70(0.38,1.02)	290.03(217.82, 387.00)	-0.26(-0.60,0.08)	328.02(250.27, 433.38)	253.97(184.84, 344.51)	1.29157358 5
Idiopathic epilepsy	Central Europe	208506.49(145230.19,2 88107.85)	-0.61(-0.65,- 0.57)	168.94(117.78, 239.45)	-0.63(-0.68,- 0.58)	200.30(145.65, 274.21)	138.37(90.52,2 04.19)	1.44755390 1
Idiopathic epilepsy	Central Latin America	625264.10(447997.88,8 49213.54)	0.48(0.39,0.58)	249.54(178.62, 339.53)	-0.92(-1.08,- 0.77)	261.30(190.46, 349.96)	238.58(166.87, 330.52)	1.09524246 8
Idiopathic epilepsy	Central sub-Saharan Africa	366966.61(210890.96,5 77470.94)	2.14(2.05,2.23)	279.63(162.66, 440.25)	-0.68(-0.77,- 0.59)	333.52(205.06, 508.39)	231.97(120.71, 383.01)	1.43779441 5
Idiopathic epilepsy	East Asia	1429169.13(1026274.9 2,1920747.13)	-1.52(-1.73,- 1.31)	100.54(72.44,1 34.10)	-1.93(-2.14,- 1.73)	112.84(83.52,1 48.93)	87.81(61.07,12 2.02)	1.28495490 1
Idiopathic epilepsy	Eastern Europe	221327.10(149747.03,3 13226.03)	-1.79(-2.02,- 1.56)	103.72(70.41,1 46.79)	-1.52(-1.73,- 1.30)	120.83(86.08,1 67.03)	88.63(55.79,13 1.74)	1.36333310 4
Idiopathic epilepsy	Eastern sub-Saharan Africa	979316.66(700638.75,1 360240.17)	1.85(1.79,1.90)	238.89(172.60, 324.02)	-0.79(-0.85,- 0.72)	281.91(212.96, 374.16)	201.07(136.57, 283.91)	1.40209860 2

Idiopathic epilepsy	High-income Asia Pacific	148928.77(95276.78,229907.53)	-0.71(-0.80,-0.63)	79.58(50.57,124.72)	-1.13(-1.22,-1.04)	87.36(56.71,135.57)	71.81(44.73,114.27)	1.216519857
Idiopathic epilepsy	High-income North America	361321.81(236123.04,530959.43)	1.02(0.86,1.17)	95.22(62.13,139.20)	-0.03(-0.18,0.11)	97.48(64.60,140.94)	92.96(59.68,137.36)	1.048623542
Idiopathic epilepsy	North Africa and middle East	955271.16(682841.75,1293168.72)	0.54(0.50,0.58)	158.28(112.61,213.23)	-0.95(-0.98,-0.91)	168.51(120.09,227.05)	147.29(105.26,198.72)	1.144082073
Idiopathic epilepsy	Oceania	24607.62(14079.45,37108.02)	2.42(2.36,2.47)	180.25(103.90,272.88)	-0.11(-0.15,-0.08)	219.38(128.28,323.47)	139.49(77.04,222.34)	1.572670759
Idiopathic epilepsy	South Asia	3697878.57(2884524.87,4712177.06)	0.11(0.03,0.19)	207.41(162.10,263.76)	-1.41(-1.49,-1.34)	209.69(161.71,272.56)	204.89(157.35,260.40)	1.023389469
Idiopathic epilepsy	Southeast Asia	805838.42(557961.33,1119211.14)	1.09(1.02,1.15)	122.18(84.27,170.83)	-0.04(-0.09,0.01)	136.32(95.35,187.32)	108.35(73.13,154.29)	1.258170703
Idiopathic epilepsy	Southern Latin America	88019.21(52266.82,139997.35)	0.71(0.65,0.77)	131.90(77.71,211.46)	-0.31(-0.37,-0.24)	140.89(88.20,218.35)	123.49(67.57,205.47)	1.140911104
Idiopathic epilepsy	Southern sub-Saharan Africa	198522.50(146332.63,263033.71)	0.60(0.18,1.01)	256.42(188.64,339.88)	-0.75(-1.10,-0.40)	320.08(246.62,406.17)	197.90(135.50,274.88)	1.617386782
Idiopathic epilepsy	Tropical Latin America	417338.39(296897.86,567517.74)	0.57(0.25,0.90)	185.55(132.01,251.82)	-0.79(-1.15,-0.44)	213.51(154.74,286.53)	159.14(109.39,220.08)	1.341651357
Idiopathic epilepsy	Western Europe	662434.68(453393.44,1018721.11)	0.87(0.77,0.98)	137.22(91.39,212.50)	0.11(0.02,0.19)	151.79(103.50,230.72)	122.79(79.11,194.41)	1.236122923
Idiopathic epilepsy	Western sub-Saharan Africa	1333419.40(992216.54,1788345.15)	2.65(2.51,2.79)	295.42(222.14,397.74)	-0.25(-0.37,-0.13)	378.59(286.23,508.01)	217.83(154.83,298.84)	1.738006726
Motor neuron disease	Andean Latin America	4723.74(3757.67,5888.11)	4.50(4.20,4.79)	8.02(6.38,9.96)	1.80(1.54,2.06)	9.33(7.20,11.92)	6.79(5.53,8.16)	1.375503541
Motor neuron disease	Australasia	23113.79(21006.39,25198.22)	3.05(2.90,3.20)	55.16(50.13,60.39)	0.77(0.59,0.95)	66.50(58.77,72.99)	44.63(39.86,50.18)	1.4900204

Motor neuron disease	Caribbean	8451.39(6865.67,10354.28)	5.09(4.78,5.41)	16.93(13.70,20.92)	3.39(3.07,3.71)	20.48(16.08,25.91)	13.60(11.03,16.92)	1.506186538
Motor neuron disease	Central Asia	2207.82(1926.05,2531.33)	2.01(1.85,2.17)	2.40(2.10,2.75)	0.51(0.36,0.65)	2.82(2.41,3.27)	2.05(1.80,2.33)	1.376194467
Motor neuron disease	Central Europe	22124.33(19284.68,25046.48)	1.56(1.31,1.80)	14.33(12.37,16.36)	0.57(0.35,0.80)	16.03(13.50,18.63)	12.69(10.81,14.91)	1.262983313
Motor neuron disease	Central Latin America	31333.12(26157.22,36995.01)	4.77(4.60,4.94)	12.74(10.62,15.02)	2.31(2.17,2.44)	15.27(12.09,18.51)	10.47(8.72,12.38)	1.45799988
Motor neuron disease	Central sub-Saharan Africa	1065.50(847.45,1333.19)	2.58(2.33,2.82)	0.98(0.76,1.27)	-0.37(-0.60,-0.14)	0.92(0.62,1.40)	1.06(0.85,1.32)	0.860432171
Motor neuron disease	East Asia	113080.73(97726.25,128867.40)	-2.15(-2.91,-1.39)	6.83(6.05,7.70)	-3.56(-4.27,-2.85)	8.61(7.29,10.12)	4.97(4.24,5.73)	1.732729693
Motor neuron disease	Eastern Europe	30570.79(27335.72,33943.33)	3.18(2.82,3.55)	10.69(9.64,11.77)	2.68(2.34,3.02)	13.53(11.71,15.44)	8.44(7.34,9.70)	1.601808547
Motor neuron disease	Eastern sub-Saharan Africa	3725.92(3107.29,4408.37)	2.76(2.59,2.93)	1.02(0.81,1.25)	-0.05(-0.21,0.11)	0.86(0.58,1.25)	1.18(0.98,1.41)	0.724304446
Motor neuron disease	High-income Asia Pacific	53490.14(48237.91,57800.70)	1.64(1.46,1.82)	14.66(13.45,15.74)	-0.82(-1.17,-0.48)	18.13(16.71,19.32)	11.53(10.34,12.57)	1.5725351
Motor neuron disease	High-income North America	252573.77(242925.55,260048.76)	2.75(2.63,2.87)	46.91(45.25,48.37)	0.77(0.62,0.92)	55.91(53.69,57.92)	38.67(37.11,39.98)	1.445610374
Motor neuron disease	North Africa and middle East	41628.31(33815.43,50605.43)	0.38(0.18,0.58)	7.84(6.36,9.54)	-0.88(-1.01,-0.75)	9.02(7.08,11.38)	6.56(5.37,7.88)	1.374505754
Motor neuron disease	Oceania	256.61(187.75,348.56)	0.85(0.54,1.15)	2.60(1.86,3.60)	-2.35(-2.64,-2.06)	2.63(1.62,3.98)	2.58(2.03,3.45)	1.019979733
Motor neuron disease	South Asia	72624.17(58024.33,88717.91)	3.49(3.37,3.62)	4.49(3.57,5.51)	1.30(1.20,1.41)	4.98(3.68,6.39)	4.01(3.24,5.13)	1.241777233

Motor neuron disease	Southeast Asia	20690.90(17170.16,24504.33)	1.99(1.80,2.17)	3.01(2.51,3.56)	-0.20(-0.35,-0.04)	3.36(2.70,4.28)	2.70(2.10,3.34)	1.243522236
Motor neuron disease	Southern Latin America	17187.38(15694.86,18385.60)	5.59(5.03,6.16)	22.30(20.38,23.94)	3.84(3.25,4.44)	27.12(23.73,29.65)	17.86(16.38,19.44)	1.518199275
Motor neuron disease	Southern sub-Saharan Africa	1687.40(1392.86,2091.32)	1.33(1.12,1.54)	2.40(1.99,2.98)	-0.70(-0.92,-0.49)	2.66(2.08,3.43)	2.22(1.84,2.74)	1.198372722
Motor neuron disease	Tropical Latin America	44751.24(40955.11,47744.19)	4.61(4.35,4.87)	18.58(16.98,19.87)	2.19(1.98,2.40)	22.17(19.49,23.92)	15.31(13.83,16.62)	1.448495003
Motor neuron disease	Western Europe	283150.27(262769.68,301431.82)	1.97(1.86,2.07)	39.39(36.60,41.89)	0.37(0.29,0.46)	45.83(42.03,48.55)	33.39(30.55,36.02)	1.372549938
Motor neuron disease	Western sub-Saharan Africa	6169.26(5024.46,7541.74)	2.18(1.71,2.66)	1.95(1.59,2.41)	-0.89(-1.36,-0.42)	1.95(1.58,2.46)	1.97(1.51,2.54)	0.992616278
Multiple sclerosis	Andean Latin America	3734.88(2951.88,4623.31)	3.30(3.22,3.39)	6.14(4.86,7.57)	0.33(0.25,0.41)	5.21(4.02,6.58)	7.03(5.39,8.61)	0.741363836
Multiple sclerosis	Australasia	11116.05(9023.55,13401.02)	2.51(2.29,2.73)	28.92(23.28,35.29)	0.59(0.39,0.79)	19.04(15.07,24.78)	38.30(29.43,47.27)	0.497010148
Multiple sclerosis	Caribbean	5729.53(4626.63,7077.59)	2.32(2.24,2.41)	11.30(9.11,14.02)	0.45(0.40,0.49)	8.15(6.04,9.94)	14.29(11.33,18.39)	0.570379682
Multiple sclerosis	Central Asia	12221.03(9340.74,15780.86)	1.70(1.61,1.78)	13.82(10.67,17.59)	-0.15(-0.19,-0.10)	13.85(10.63,18.45)	14.00(10.52,18.49)	0.989024064
Multiple sclerosis	Central Europe	51363.97(40324.72,75257.23)	-0.52(-0.55,-0.48)	32.74(25.57,48.06)	-0.95(-1.00,-0.91)	26.11(19.92,42.49)	39.34(30.23,59.19)	0.663675177
Multiple sclerosis	Central Latin America	24523.99(20014.50,29813.89)	4.10(3.89,4.30)	9.63(7.88,11.70)	1.22(1.09,1.35)	8.10(6.14,10.98)	11.03(7.83,13.77)	0.734688388
Multiple sclerosis	Central sub-Saharan Africa	4031.36(2877.93,5786.58)	3.37(3.24,3.50)	4.94(3.46,7.10)	0.09(0.02,0.16)	4.22(2.55,6.45)	5.62(4.08,8.25)	0.751610833

Multiple sclerosis	East Asia	75174.87(62164.20,95959.63)	0.72(0.52,0.92)	3.75(3.11,4.78)	-1.35(-1.53,-1.16)	3.84(2.90,5.37)	3.69(2.86,4.72)	1.04134633
Multiple sclerosis	Eastern Europe	69170.34(48216.64,125154.29)	-1.01(-1.26,-0.75)	26.21(18.26,47.55)	-1.14(-1.37,-0.90)	22.95(15.74,45.55)	29.29(19.44,54.26)	0.783626095
Multiple sclerosis	Eastern sub-Saharan Africa	12716.76(8611.12,17088.63)	2.97(2.83,3.11)	5.07(3.37,6.88)	-0.01(-0.08,0.06)	4.22(2.50,6.42)	5.88(3.94,8.05)	0.717832593
Multiple sclerosis	High-income Asia Pacific	15515.81(12323.69,20968.26)	0.69(0.62,0.76)	5.59(4.43,7.65)	-0.39(-0.42,-0.35)	4.79(3.86,7.08)	6.39(4.96,8.61)	0.749634641
Multiple sclerosis	High-income North America	241677.90(195635.01,278600.51)	1.90(1.77,2.03)	49.29(40.22,57.37)	0.29(0.19,0.40)	32.58(25.09,38.53)	65.21(52.00,76.71)	0.499643186
Multiple sclerosis	North Africa and middle East	115885.77(93053.42,144757.93)	3.23(3.20,3.26)	19.92(16.12,24.71)	0.27(0.24,0.31)	15.56(12.52,19.61)	24.65(19.32,32.02)	0.631130761
Multiple sclerosis	Oceania	363.24(263.59,502.49)	2.73(2.66,2.79)	3.48(2.52,4.81)	-0.39(-0.45,-0.34)	3.45(2.37,5.31)	3.52(2.47,5.67)	0.979871483
Multiple sclerosis	South Asia	144077.35(119712.06,177475.52)	2.98(2.92,3.03)	8.58(7.15,10.54)	0.29(0.24,0.35)	7.10(5.72,8.98)	10.08(7.84,13.57)	0.704964461
Multiple sclerosis	Southeast Asia	32509.87(25887.29,44139.20)	1.76(1.67,1.84)	4.53(3.61,6.11)	-0.58(-0.66,-0.50)	4.49(3.38,6.40)	4.59(3.62,6.60)	0.978674777
Multiple sclerosis	Southern Latin America	10638.55(8314.04,15021.04)	1.24(1.17,1.30)	14.04(10.97,19.80)	-0.42(-0.50,-0.34)	12.13(9.40,18.70)	15.84(12.09,22.98)	0.766018127
Multiple sclerosis	Southern sub-Saharan Africa	4686.12(3927.21,5552.72)	2.25(2.14,2.36)	6.64(5.59,7.82)	0.06(-0.02,0.14)	6.02(4.83,7.35)	7.14(5.88,8.49)	0.843615941
Multiple sclerosis	Tropical Latin America	25311.83(20304.86,31970.63)	3.08(2.95,3.21)	10.08(8.08,12.74)	0.29(0.20,0.39)	8.11(6.51,10.77)	11.86(9.05,15.33)	0.683779124
Multiple sclerosis	Western Europe	271038.64(222001.49,324345.63)	1.28(1.25,1.32)	43.50(35.79,52.73)	0.27(0.26,0.29)	31.42(24.40,39.30)	55.20(44.20,68.05)	0.569187026

Multiple sclerosis	Western sub-Saharan Africa	28343.97(22716.03,35169.58)	4.19(4.10,4.27)	9.90(7.98,12.50)	1.00(0.93,1.07)	6.40(4.61,8.65)	13.10(10.45,17.14)	0.488482935
Neurological disorders	Andean Latin America	669928.47(403703.92,1061895.01)	2.11(2.09,2.14)	1118.87(681.97,1755.16)	-0.26(-0.28,-0.24)	1008.46(651.77,1505.68)	1228.46(698.85,2022.48)	0.820918567
Neurological disorders	Australasia	491658.92(307915.31,765069.43)	2.15(2.13,2.17)	1231.79(729.88,1943.21)	-0.01(-0.03,0.01)	1110.65(715.09,1717.78)	1344.86(737.59,2213.06)	0.825847725
Neurological disorders	Caribbean	622830.34(369601.00,1004427.45)	1.54(1.53,1.56)	1265.71(750.69,2029.87)	0.00(-0.05,0.05)	1155.76(755.46,1728.85)	1374.81(726.63,2302.72)	0.840673047
Neurological disorders	Central Asia	1088663.96(621623.26,1739682.86)	1.17(1.11,1.23)	1363.07(825.00,2139.12)	0.07(-0.02,0.16)	1276.25(843.47,1898.27)	1454.27(807.01,2392.51)	0.877585173
Neurological disorders	Central Europe	2074727.71(1273406.36,3301261.70)	0.72(0.67,0.76)	1292.88(771.72,2054.27)	-0.14(-0.15,-0.13)	1164.43(744.71,1784.90)	1423.57(795.60,2355.52)	0.817968742
Neurological disorders	Central Latin America	3235562.19(1842436.00,5218607.81)	2.10(2.08,2.12)	1323.08(766.24,2115.09)	-0.10(-0.15,-0.05)	1171.73(756.37,1778.44)	1466.62(780.61,2451.02)	0.798937269
Neurological disorders	Central sub-Saharan Africa	1205481.24(664567.40,1995338.87)	2.83(2.78,2.89)	1325.20(778.64,2094.20)	-0.07(-0.11,-0.03)	1260.10(783.84,1952.56)	1381.57(763.57,2272.71)	0.912077395
Neurological disorders	East Asia	18034421.21(10031195.66,30122084.33)	1.73(1.70,1.76)	1076.34(597.98,1781.78)	-0.12(-0.15,-0.10)	969.14(590.57,1525.14)	1178.77(601.40,2042.84)	0.82216066
Neurological disorders	Eastern Europe	3416892.42(2071105.57,5379221.32)	0.42(0.38,0.46)	1264.48(739.10,2029.07)	-0.02(-0.06,0.02)	1097.24(689.52,1718.01)	1425.61(790.73,2369.16)	0.769664397
Neurological disorders	Eastern sub-Saharan Africa	3064703.98(1823369.55,4849527.73)	2.60(2.56,2.64)	1117.19(685.41,1725.53)	-0.04(-0.05,-0.02)	1051.47(678.04,1584.86)	1175.36(685.28,1861.66)	0.894595345
Neurological disorders	High-income Asia Pacific	3967450.24(2400554.50,6645145.72)	2.88(2.80,2.96)	1067.56(616.47,1753.82)	0.22(0.18,0.26)	939.55(574.22,1509.74)	1188.99(639.00,1994.03)	0.790203366
Neurological disorders	High-income North America	6570271.74(4013981.95,10380241.11)	1.46(1.43,1.49)	1340.08(757.36,2177.42)	0.00(-0.04,0.04)	1114.30(722.32,1714.52)	1562.89(777.33,2706.29)	0.712976229

Neurological disorders	North Africa and middle East	7156040.57(3769688.41,12184398.02)	2.31(2.28,2.34)	1382.14(776.61,2273.81)	-0.11(-0.13,-0.09)	1232.61(745.16,1943.39)	1545.47(812.66,2649.12)	0.797565334
Neurological disorders	Oceania	120254.91(58684.87,206952.44)	2.69(2.68,2.71)	1237.37(697.50,2026.43)	-0.10(-0.11,-0.08)	1178.79(730.85,1842.18)	1297.77(650.26,2232.28)	0.908318006
Neurological disorders	South Asia	19068926.20(9725695.87,32172757.88)	1.91(1.87,1.95)	1189.75(648.45,1975.49)	-0.24(-0.28,-0.21)	1085.03(635.80,1723.62)	1298.88(669.81,2211.15)	0.835352232
Neurological disorders	Southeast Asia	7705142.85(3582954.02,13824792.52)	1.96(1.93,1.98)	1240.33(627.59,2145.96)	0.03(0.02,0.04)	1088.04(592.15,1805.62)	1380.21(625.93,2529.64)	0.788317246
Neurological disorders	Southern Latin America	848095.86(519744.05,1335733.82)	1.92(1.89,1.95)	1119.57(663.45,1775.37)	0.14(0.12,0.15)	1013.74(664.48,1536.87)	1223.56(661.47,2027.86)	0.828515318
Neurological disorders	Southern sub-Saharan Africa	843682.55(482667.68,1356713.19)	1.51(1.32,1.70)	1262.17(755.95,1978.92)	-0.10(-0.21,0.01)	1215.26(781.79,1841.35)	1299.66(724.23,2117.62)	0.935057931
Neurological disorders	Tropical Latin America	3367762.36(1750228.68,5686900.12)	2.16(2.13,2.19)	1471.72(769.26,2492.03)	0.06(0.01,0.11)	1305.49(763.21,2118.38)	1634.52(778.32,2913.02)	0.798695203
Neurological disorders	Western Europe	9417304.06(5678674.45,14885141.28)	1.33(1.31,1.36)	1432.56(777.08,2368.95)	0.08(0.06,0.10)	1199.05(720.26,1881.47)	1664.25(825.21,2921.31)	0.720472109
Neurological disorders	Western sub-Saharan Africa	4754609.96(2619701.88,7979177.21)	3.04(3.00,3.09)	1425.08(834.27,2270.80)	0.12(0.09,0.15)	1364.81(851.83,2084.69)	1477.36(791.24,2463.63)	0.923817143
Parkinson's disease	Andean Latin America	44791.23(37546.12,52144.79)	4.28(4.13,4.42)	84.06(70.57,97.92)	0.35(0.26,0.45)	105.95(87.14,125.53)	64.54(53.54,74.55)	1.641449246
Parkinson's disease	Australasia	45277.21(40460.59,49754.44)	2.87(2.72,3.03)	84.43(75.44,93.15)	0.10(-0.03,0.24)	121.53(108.82,134.09)	53.52(47.25,59.85)	2.270760556
Parkinson's disease	Caribbean	40657.16(35859.91,45818.41)	3.08(3.04,3.12)	79.07(69.72,89.10)	0.54(0.50,0.58)	103.45(90.97,117.42)	58.70(51.63,66.19)	1.762438733
Parkinson's disease	Central Asia	46622.76(42387.04,51143.84)	2.12(1.99,2.25)	85.45(77.69,93.27)	1.04(0.88,1.20)	114.44(103.29,128.18)	68.33(62.27,74.81)	1.674762769

Parkinson's disease	Central Europe	179385.33(159196.95,198301.84)	1.74(1.68,1.80)	77.48(68.76,85.70)	-0.14(-0.18,-0.10)	105.29(93.18,117.47)	60.03(52.81,66.80)	1.753835202
Parkinson's disease	Central Latin America	163356.38(144120.74,184002.11)	4.19(4.13,4.25)	73.33(64.70,82.58)	0.11(0.05,0.17)	95.60(82.94,109.15)	55.13(48.34,63.05)	1.734116128
Parkinson's disease	Central sub-Saharan Africa	26742.52(21990.08,33354.70)	2.58(2.50,2.66)	67.95(56.14,84.68)	-0.28(-0.35,-0.21)	91.58(74.23,117.24)	52.86(42.94,66.48)	1.732565402
Parkinson's disease	East Asia	1616841.82(1392716.55,1855684.42)	3.07(2.95,3.19)	83.85(72.56,95.77)	-0.40(-0.53,-0.27)	114.88(96.26,134.64)	61.19(51.29,71.13)	1.877311697
Parkinson's disease	Eastern Europe	241195.72(216885.80,266648.49)	1.01(0.86,1.17)	67.85(61.07,75.02)	-0.07(-0.21,0.08)	95.84(84.33,108.38)	54.67(48.08,61.57)	1.752943619
Parkinson's disease	Eastern sub-Saharan Africa	86283.03(74468.76,116552.32)	2.51(2.40,2.62)	68.74(59.22,96.17)	-0.06(-0.08,-0.04)	88.76(75.18,121.45)	52.41(43.80,76.72)	1.6937354
Parkinson's disease	High-income Asia Pacific	305660.54(264553.14,333622.63)	4.12(3.98,4.26)	58.11(51.04,63.45)	0.46(0.38,0.54)	76.47(68.51,83.03)	44.80(38.06,49.75)	1.706678913
Parkinson's disease	High-income North America	556993.73(511705.30,590394.88)	2.86(2.74,2.99)	84.05(77.48,89.05)	0.97(0.82,1.12)	122.33(113.10,129.70)	54.75(49.51,58.83)	2.234394129
Parkinson's disease	North Africa and middle East	300697.71(266276.83,365359.72)	3.48(3.43,3.54)	84.39(74.67,103.20)	0.10(0.03,0.17)	100.48(88.82,120.45)	68.39(59.92,88.26)	1.469336811
Parkinson's disease	Oceania	6929.50(4602.56,8456.81)	2.54(2.46,2.63)	134.20(94.34,160.72)	-0.37(-0.43,-0.30)	168.79(112.61,205.42)	101.74(73.35,123.19)	1.659078471
Parkinson's disease	South Asia	984633.83(854638.78,1124851.58)	3.61(3.48,3.73)	82.06(71.05,93.98)	-0.29(-0.42,-0.16)	99.44(83.08,116.90)	66.50(54.86,80.29)	1.495378545
Parkinson's disease	Southeast Asia	421112.74(363277.11,473854.60)	3.50(3.46,3.53)	83.38(71.79,93.45)	0.30(0.26,0.33)	104.28(89.34,118.53)	67.86(53.29,77.90)	1.536649435
Parkinson's disease	Southern Latin America	72473.86(66659.13,77505.81)	2.41(2.34,2.47)	83.72(77.06,89.63)	0.05(0.00,0.11)	126.99(116.98,136.34)	54.45(49.41,58.97)	2.332325104

Parkinson's disease	Southern sub-Saharan Africa	28752.53(26239.04,31169.15)	3.09(2.70,3.48)	62.47(56.83,67.42)	0.84(0.51,1.18)	84.52(74.57,91.71)	49.28(43.43,54.34)	1.714965153
Parkinson's disease	Tropical Latin America	163657.67(148826.57,177156.13)	4.04(3.99,4.09)	71.88(65.26,77.85)	0.15(0.10,0.20)	91.69(83.65,99.66)	56.89(50.45,62.12)	1.611676113
Parkinson's disease	Western Europe	841645.71(761496.73,910966.31)	2.14(2.08,2.20)	80.02(72.60,87.02)	0.25(0.20,0.30)	113.03(103.31,122.17)	55.61(49.62,61.22)	2.032614023
Parkinson's disease	Western sub-Saharan Africa	118904.62(103654.90,134223.87)	3.12(3.03,3.21)	87.30(76.25,97.98)	0.70(0.59,0.81)	98.46(83.23,113.51)	77.08(64.74,89.52)	1.27742387
Stroke	Andean Latin America	470939.26(393705.45,572695.86)	0.47(0.25,0.69)	820.22(686.85,996.13)	-2.53(-2.76,-2.31)	846.69(697.40,1039.23)	794.58(662.69,959.66)	1.065573392
Stroke	Australasia	231104.00(206435.17,252044.88)	-0.56(-0.73,-0.40)	460.59(414.18,502.65)	-3.21(-3.38,-3.04)	463.30(422.97,501.56)	455.25(400.40,504.16)	1.017677092
Stroke	Caribbean	850163.55(726573.35,984569.24)	1.32(1.19,1.46)	1663.35(1420.66,1924.32)	-0.88(-1.03,-0.73)	1705.37(1454.03,1988.76)	1625.95(1363.76,1938.98)	1.04885048
Stroke	Central Asia	2204424.29(2014443.88,2418524.73)	0.80(0.50,1.10)	3122.35(2863.45,3398.02)	-0.55(-0.96,-0.14)	3728.04(3403.16,4085.28)	2646.94(2406.28,2911.98)	1.408431232
Stroke	Central Europe	3687846.50(3236210.64,4114622.69)	-1.16(-1.35,-0.96)	1731.98(1523.52,1934.42)	-2.58(-2.80,-2.36)	1989.34(1746.03,2230.82)	1508.76(1318.96,1680.56)	1.318531301
Stroke	Central Latin America	1941065.04(1698856.10,2228842.14)	1.22(1.09,1.35)	821.02(717.85,941.60)	-2.10(-2.24,-1.96)	879.65(759.63,1023.50)	769.34(671.05,890.36)	1.143379124
Stroke	Central sub-Saharan Africa	1363972.72(1090393.88,1684468.26)	1.72(1.68,1.76)	2476.47(1959.77,3061.26)	-0.94(-1.00,-0.88)	2678.84(2155.33,3282.37)	2297.45(1720.22,2929.83)	1.166004944
Stroke	East Asia	47598099.40(41485659.43,54050918.26)	1.05(0.90,1.19)	2405.97(2105.77,2718.36)	-1.89(-2.07,-1.71)	3021.79(2520.47,3589.09)	1879.17(1574.69,2202.92)	1.608047333
Stroke	Eastern Europe	8459592.28(7598493.13,9321472.38)	-1.28(-1.79,-0.77)	2507.25(2256.23,2759.82)	-1.99(-2.51,-1.47)	3098.53(2694.33,3541.69)	2063.31(1805.07,2348.47)	1.501725819

Stroke	Eastern sub-Saharan Africa	4185930.82(3500348.29,4904229.20)	1.32(1.18,1.45)	2421.39(2046.69,2807.31)	-1.14(-1.20,-1.09)	2628.47(2161.86,3132.05)	2221.94(1853.01,2583.34)	1.182962028
Stroke	High-income Asia Pacific	3245485.57(2834639.64,3581419.96)	-0.75(-0.85,-0.64)	739.23(659.42,814.24)	-3.54(-3.68,-3.41)	877.06(800.42,959.00)	612.68(525.68,692.02)	1.43151084
Stroke	High-income North America	4156206.67(3766199.56,4520026.69)	0.42(0.29,0.56)	689.63(625.70,751.66)	-1.39(-1.50,-1.27)	700.06(641.33,759.27)	676.86(603.17,747.57)	1.034271324
Stroke	North Africa and middle East	7946004.27(7060208.94,8870766.21)	1.30(1.21,1.39)	1826.16(1635.30,2026.16)	-1.29(-1.33,-1.26)	1789.84(1578.41,2037.41)	1861.39(1661.17,2068.88)	0.961560483
Stroke	Oceania	267595.79(208538.30,340992.36)	2.78(2.74,2.82)	3333.36(2645.06,4156.82)	-0.26(-0.29,-0.23)	3726.38(2823.07,4830.60)	2916.54(2301.47,3640.83)	1.277671141
Stroke	South Asia	24119348.47(21416431.22,26859938.99)	1.81(1.74,1.89)	1702.55(1510.89,1891.03)	-1.38(-1.49,-1.27)	1820.64(1566.77,2099.31)	1587.18(1346.47,1850.14)	1.147095746
Stroke	Southeast Asia	18184273.15(16289869.91,19884314.16)	2.35(2.26,2.45)	3014.01(2713.32,3280.42)	-0.48(-0.59,-0.37)	3485.24(3109.47,3867.98)	2579.04(2280.39,2879.53)	1.351371774
Stroke	Southern Latin America	789879.26(733097.12,841017.00)	-0.98(-1.18,-0.79)	963.82(896.09,1025.32)	-2.96(-3.14,-2.77)	1121.03(1046.10,1192.58)	830.76(757.90,894.96)	1.349409915
Stroke	Southern sub-Saharan Africa	945582.03(867585.56,1028078.02)	1.58(1.10,2.07)	1723.75(1583.43,1863.25)	-0.43(-0.89,0.04)	1863.07(1712.44,2031.09)	1591.70(1426.01,1757.34)	1.170487855
Stroke	Tropical Latin America	2934468.72(2749809.65,3090538.73)	-0.04(-0.14,0.06)	1221.28(1143.41,1286.96)	-3.16(-3.27,-3.05)	1411.63(1320.71,1489.71)	1065.19(978.87,1140.65)	1.325235636
Stroke	Western Europe	5280689.58(4757434.36,5650036.95)	-1.74(-1.93,-1.55)	557.34(510.12,596.83)	-3.44(-3.61,-3.27)	608.04(565.69,645.66)	510.39(457.63,553.86)	1.191323885
Stroke	Western sub-Saharan Africa	4369512.87(3707618.29,5124470.18)	1.70(1.62,1.77)	2105.24(1817.91,2424.97)	-0.91(-0.99,-0.84)	2128.20(1788.56,2549.74)	2082.15(1748.71,2446.61)	1.022116749

CI - confidence interval, EAPC - estimated annual percentage change, SDI - socio-demographic index, UI - uncertainty interval.

Table S7. The age-standardized DALY rate of neurological disorders in 1990 and 2019 and its temporal trends, by countries

	Absolute numbers		Age-standardized rate				
	2019(95% UI)	EAPC(95%CI) (1990-2019)	2019(95% UI)	EAPC(95% CI) (1990-2019)	males, 2019 (95% UI)	females, 2019(95% UI)	male-to-female ratio
Afghanistan	382679.59(190210.09, 668250.67)	2.88(2.71,3.05)	1503.00(853.80,2492.15)	-0.36(-0.38,-0.33)	1339.95(794.1 1,2108.46)	1674.43(906.60,2 850.59)	0.800246427
Albania	46451.30(28981.64,70 953.63)	0.25(0.14,0.35)	1418.24(877.63,2193.38)	-0.25(-0.31,-0.19)	1272.32(834.8 9,1886.55)	1567.16(915.15,2 507.62)	0.811865552
Algeria	494778.09(254584.90, 842877.91)	2.05(2.01,2.09)	1365.12(738.51,2292.05)	-0.29(-0.31,-0.27)	1195.13(685.0 6,1956.67)	1542.92(788.48,2 654.18)	0.774589502
American Samoa	633.95(342.61,1043.34)	1.18(1.02,1.35)	1301.24(742.91,2121.09)	0.00(-0.02,0.02)	1243.72(779.2 5,1883.46)	1352.05(695.71,2 317.44)	0.91988195
Andorra	1532.77(885.09,2416.9 4)	2.33(2.08,2.57)	1360.28(731.14,2268.71)	-0.05(-0.06,-0.03)	1132.80(658.6 7,1770.41)	1598.81(772.85,2 838.45)	0.70852762
Angola	286536.33(150409.37, 486090.70)	3.47(3.42,3.52)	1399.26(808.80,2218.63)	0.01(-0.02,0.04)	1357.90(828.6 7,2060.86)	1432.47(788.28,2 363.50)	0.94794407
Antigua and Barbuda	1301.84(762.67,2066.4 6)	1.36(1.24,1.48)	1415.77(833.59,2237.68)	-0.17(-0.20,-0.14)	1387.48(907.6 2,2077.75)	1444.89(770.43,2 451.87)	0.960268114
Argentina	548735.42(326039.35, 866022.88)	1.93(1.89,1.97)	1092.21(635.06,1742.46)	0.18(0.17,0.20)	990.15(630.63, 1522.69)	1190.99(637.15,1 963.45)	0.831364392
Armenia	41209.31(23470.26,66 870.37)	0.63(0.60,0.67)	1198.09(674.98,1980.02)	0.00(-0.03,0.02)	1064.23(625.8 8,1701.30)	1325.98(698.03,2 272.12)	0.802598998
Australia	415085.86(258663.55, 647117.16)	2.23(2.20,2.25)	1227.32(726.11,1933.10)	0.01(-0.01,0.03)	1107.46(709.1 6,1699.39)	1339.79(726.70,2 201.70)	0.826595108
Austria	174314.29(106608.56, 273191.00)	1.24(1.16,1.31)	1326.26(734.00,2202.54)	0.06(0.03,0.10)	1102.55(667.3 7,1743.24)	1547.91(777.96,2 689.11)	0.712286684

Azerbaijan	120522.62(64737.25,204057.10)	1.20(1.14,1.25)	1352.38(769.16,2207.32)	0.01(-0.04,0.07)	1247.53(754.69,1966.36)	1454.90(784.54,2455.64)	0.857465104
Bahrain	16094.44(7722.01,28555.26)	4.49(4.19,4.79)	1387.65(768.62,2266.38)	-0.30(-0.33,-0.26)	1264.69(737.44,2004.39)	1590.06(831.57,2711.23)	0.795372029
Bangladesh	1559290.05(701458.79,2764220.84)	1.62(1.51,1.73)	1075.28(527.81,1866.06)	-0.36(-0.40,-0.31)	958.59(499.05,1611.25)	1184.91(554.31,2085.99)	0.80899345
Barbados	4936.67(2975.35,7695.93)	1.02(0.96,1.07)	1324.75(762.69,2118.13)	-0.03(-0.06,0.01)	1224.12(781.46,1830.94)	1422.60(744.85,2378.13)	0.860482048
Belarus	154024.80(93312.79,240895.30)	0.30(0.27,0.33)	1229.98(711.42,1953.94)	-0.07(-0.10,-0.04)	1064.89(661.47,1655.75)	1388.05(756.28,2326.87)	0.767186371
Belgium	258119.55(150538.03,423470.36)	1.22(1.19,1.24)	1578.75(774.90,2768.52)	0.12(0.07,0.17)	1286.56(748.02,2075.53)	1867.07(791.55,3483.45)	0.689075607
Belize	4596.06(2436.62,7670.02)	3.30(3.27,3.33)	1281.45(744.37,2049.10)	0.08(-0.01,0.16)	1170.51(738.67,1790.48)	1388.80(725.43,2344.27)	0.842821001
Benin	132707.70(69469.94,219325.26)	3.25(3.19,3.32)	1434.34(812.34,2352.26)	-0.08(-0.11,-0.04)	1390.94(849.64,2166.98)	1468.52(765.18,2500.60)	0.947168848
Bermuda	1125.29(679.55,1835.04)	1.24(1.18,1.30)	1201.23(666.91,1961.43)	-0.31(-0.35,-0.28)	1099.82(678.25,1732.05)	1309.86(668.54,2243.16)	0.839646081
Bhutan	8221.93(4014.12,14254.17)	1.58(1.49,1.67)	1224.41(645.48,2058.26)	0.00(-0.02,0.02)	1141.28(640.93,1874.88)	1316.20(645.78,2260.94)	0.867101448
Bolivia (Plurinational State of)	118131.78(66910.19,186452.48)	2.10(2.07,2.12)	1199.32(721.92,1854.10)	-0.38(-0.44,-0.31)	1075.22(683.30,1629.64)	1324.48(740.22,2166.41)	0.8118041
Bosnia and Herzegovina	54275.68(32415.04,85396.26)	0.07(-0.02,0.17)	1267.58(722.69,2019.95)	-0.29(-0.34,-0.25)	1141.18(699.72,1758.32)	1393.40(743.24,2312.41)	0.818990413
Botswana	24536.00(13471.45,40722.65)	2.56(2.41,2.71)	1324.78(791.69,2075.81)	0.14(0.05,0.23)	1287.26(811.43,1974.00)	1349.22(745.85,2202.21)	0.954079273

Brazil	3278800.52(1703362.07,5555459.38)	2.15(2.12,2.19)	1474.48(771.38,2502.05)	0.06(0.01,0.11)	1308.17(766.23,2121.45)	1637.15(779.92,2916.44)	0.799053284
Brunei Darussalam	4131.87(2381.98,6684.51)	2.02(2.00,2.05)	1205.05(757.63,1873.97)	-0.07(-0.14,0.00)	1164.19(787.67,1712.00)	1253.15(718.07,2082.44)	0.929016264
Bulgaria	133294.09(82744.41,213257.77)	0.28(0.24,0.31)	1312.62(783.82,2065.72)	-0.08(-0.12,-0.05)	1200.51(771.03,1821.02)	1430.91(797.12,2393.38)	0.838984635
Burkina Faso	231017.88(120350.83,384066.34)	3.17(3.07,3.27)	1415.94(797.32,2353.33)	0.02(-0.03,0.07)	1334.35(782.21,2133.74)	1482.56(793.25,2548.15)	0.900031675
Burundi	87682.03(47989.90,140433.10)	2.05(1.86,2.24)	1112.60(668.37,1769.38)	-0.43(-0.52,-0.35)	1047.56(653.09,1631.17)	1178.22(670.57,1907.83)	0.889103989
Cambodia	173207.12(76240.78,316326.13)	2.36(2.34,2.38)	1263.74(629.18,2214.36)	0.08(0.05,0.10)	1118.58(594.58,1868.21)	1391.73(640.57,2565.04)	0.803732061
Cameroon	319201.85(165562.41,544954.94)	3.93(3.86,4.00)	1486.83(862.40,2466.52)	0.12(0.09,0.16)	1455.57(903.54,2317.00)	1512.39(817.03,2614.99)	0.962432658
Canada	687162.38(420206.01,1091833.88)	1.83(1.82,1.84)	1329.73(742.09,2192.57)	-0.08(-0.09,-0.06)	1064.14(702.10,1619.74)	1589.93(759.34,2828.43)	0.669298613
Cabo Verde	7478.46(3997.48,12236.57)	2.08(1.92,2.24)	1430.35(812.48,2344.90)	0.06(-0.01,0.13)	1466.86(916.17,2287.13)	1399.30(704.54,2387.78)	1.048281725
Central African Republic	51281.68(26620.21,83799.39)	2.16(2.05,2.28)	1350.19(802.26,2182.45)	-0.07(-0.09,-0.04)	1299.17(815.48,1997.92)	1382.12(765.84,2329.88)	0.939979782
Chad	159600.84(83985.24,264534.33)	3.53(3.49,3.56)	1399.74(784.84,2301.71)	0.23(0.20,0.26)	1353.74(809.79,2105.00)	1442.67(739.20,2445.78)	0.938355582
Chile	244997.65(150553.19,381644.25)	2.09(2.06,2.12)	1175.50(711.11,1853.69)	0.06(0.03,0.09)	1058.40(709.59,1586.73)	1294.60(707.56,2115.05)	0.817548994
China	17381405.54(9666404.82,29082962.37)	1.72(1.69,1.75)	1076.59(598.04,1779.55)	-0.13(-0.15,-0.10)	971.99(592.23,1531.59)	1178.13(599.32,2046.71)	0.825029224

Colombia	648547.79(356506.83,1047887.19)	2.27(2.24,2.30)	1264.42(683.75,2042.47)	-0.03(-0.09,0.02)	1095.70(651.76,1682.94)	1425.12(712.46,2414.04)	0.768847656
Comoros	6593.79(3761.97,10634.59)	1.80(1.72,1.88)	1135.00(669.62,1786.14)	-0.08(-0.13,-0.03)	1037.78(637.63,1594.78)	1223.77(693.86,1952.50)	0.848025081
Congo	54559.02(29397.40,90366.35)	2.47(2.41,2.54)	1415.43(829.84,2223.42)	-0.21(-0.25,-0.17)	1343.99(813.65,2066.84)	1481.36(829.24,2406.22)	0.907264611
Costa Rica	65313.29(35479.57,107815.44)	2.58(2.49,2.66)	1311.40(711.30,2168.35)	0.15(0.12,0.19)	1173.91(718.91,1826.12)	1440.60(713.69,2452.64)	0.81488023
Côte d'Ivoire	271260.04(141884.75,463015.49)	2.77(2.62,2.92)	1420.95(815.12,2286.14)	0.01(-0.03,0.06)	1394.10(841.67,2148.36)	1447.53(758.90,2446.27)	0.963088637
Croatia	82468.36(50125.09,131882.38)	0.59(0.46,0.73)	1288.93(731.98,2047.41)	-0.10(-0.16,-0.03)	1158.21(730.09,1784.94)	1419.57(757.25,2361.45)	0.815888446
Cuba	170488.44(97618.46,271887.44)	1.11(1.09,1.14)	1147.19(631.69,1909.30)	0.00(-0.05,0.05)	1029.18(613.60,1614.06)	1271.55(632.02,2218.77)	0.809384845
Cyprus	20444.14(11204.32,33626.51)	2.31(2.27,2.35)	1329.61(689.57,2273.44)	-0.33(-0.36,-0.30)	1086.87(634.91,1738.88)	1557.18(714.98,2812.60)	0.697976619
Czechia	195273.72(117869.99,313323.45)	0.99(0.91,1.07)	1269.01(724.84,2037.77)	-0.06(-0.09,-0.03)	1130.21(689.93,1766.77)	1410.52(762.91,2332.35)	0.801272947
Democratic Republic of the Congo	778072.58(408187.40,1310753.76)	2.70(2.60,2.79)	1286.62(744.46,2066.81)	-0.09(-0.16,-0.03)	1211.37(734.29,1898.50)	1353.03(742.54,2272.70)	0.895302967
Denmark	106110.41(65468.89,165483.89)	0.80(0.78,0.83)	1281.98(711.84,2109.99)	0.05(0.00,0.10)	1086.22(668.94,1681.58)	1477.96(751.73,2545.46)	0.73494767
Djibouti	9984.93(5564.00,16314.69)	3.80(3.70,3.89)	1176.40(694.26,1893.93)	0.07(0.03,0.11)	1108.47(683.14,1721.27)	1247.04(700.28,2043.99)	0.888886155
Dominica	1155.31(719.05,1731.52)	0.19(0.13,0.25)	1505.11(936.25,2272.54)	0.05(0.02,0.07)	1439.38(947.15,2074.78)	1577.77(896.17,2538.38)	0.912290753

Dominican Republic	125539.57(66133.45,208273.05)	2.22(2.18,2.27)	1234.92(672.17,2005.11)	0.26(0.19,0.32)	1127.55(660.24,1748.88)	1344.83(675.42,2310.52)	0.838430528
Ecuador	208414.08(120423.15,337665.81)	2.18(2.13,2.23)	1283.01(760.05,2053.73)	-0.20(-0.24,-0.16)	1160.79(724.45,1776.00)	1404.25(766.30,2331.81)	0.826624789
Egypt	1050486.56(470196.82,1877241.22)	2.44(2.38,2.51)	1336.25(709.79,2284.83)	0.16(0.12,0.21)	1172.53(671.27,1900.74)	1526.52(739.72,2669.55)	0.768104991
El Salvador	78599.73(41754.87,130018.73)	1.38(1.35,1.41)	1228.27(650.01,2022.28)	-0.03(-0.07,0.01)	1071.15(634.68,1673.60)	1360.52(670.61,2349.63)	0.787304648
Equatorial Guinea	14323.17(7084.46,23982.66)	4.67(4.55,4.78)	1420.73(810.87,2367.15)	0.38(0.31,0.45)	1360.48(810.59,2210.57)	1471.25(810.99,2460.45)	0.92470757
Eritrea	53443.61(29535.46,86609.74)	3.24(3.03,3.46)	1191.31(720.27,1923.47)	0.29(0.18,0.40)	1109.44(685.25,1741.72)	1243.11(718.31,2011.96)	0.892472593
Estonia	26713.87(16699.52,41281.46)	0.60(0.52,0.67)	1336.70(809.77,2093.80)	0.02(-0.07,0.11)	1219.71(785.96,1824.46)	1461.48(827.08,2387.96)	0.834572817
Ethiopia	703161.77(400407.04,1115221.27)	1.97(1.85,2.08)	1031.61(616.44,1646.88)	-0.35(-0.40,-0.31)	976.17(607.53,1529.61)	1086.86(614.72,1770.15)	0.898159974
Micronesia (Federated States of)	1084.94(558.15,1825.67)	0.30(0.23,0.37)	1395.47(810.12,2256.26)	-0.12(-0.14,-0.09)	1341.79(836.79,2000.92)	1442.39(757.53,2471.77)	0.930250426
Fiji	10058.09(5320.25,16998.81)	1.17(1.14,1.19)	1322.09(762.30,2136.91)	-0.10(-0.12,-0.07)	1283.30(809.20,1929.05)	1364.14(717.19,2314.02)	0.940740113
Finland	118475.22(73569.66,184554.16)	1.33(1.27,1.39)	1396.03(762.93,2300.55)	0.06(0.04,0.08)	1161.13(704.47,1822.38)	1631.38(813.03,2883.66)	0.711743395
France	1449290.05(892601.19,2272177.84)	1.39(1.37,1.41)	1425.76(791.47,2289.69)	0.01(-0.03,0.06)	1228.91(735.61,1888.97)	1613.84(834.28,2732.99)	0.761485746
Gabon	20708.47(11372.97,34414.88)	1.93(1.88,1.98)	1493.34(858.91,2366.89)	-0.16(-0.19,-0.13)	1501.45(913.36,2322.20)	1483.98(811.51,2425.64)	1.011774988

Georgia	59805.30(35286.10,97416.29)	-0.39(-0.47,-0.31)	1222.59(692.46,1996.83)	-0.06(-0.16,0.04)	1100.41(674.09,1757.76)	1348.93(705.84,2265.50)	0.815767134
Germany	1981285.88(1212032.42,3065200.43)	1.03(0.99,1.06)	1539.15(811.18,2544.76)	0.18(0.14,0.23)	1301.07(772.84,2017.22)	1784.65(837.63,3165.18)	0.729029901
Ghana	329249.07(169823.43,566583.61)	3.23(3.18,3.28)	1338.40(746.77,2215.59)	0.21(0.18,0.24)	1257.71(739.53,2004.31)	1416.76(742.50,2359.21)	0.887739894
Greece	224050.88(130434.14,369505.96)	1.21(1.18,1.24)	1329.86(661.91,2276.79)	0.03(0.01,0.04)	1096.58(609.55,1793.32)	1559.99(703.26,2787.18)	0.702939254
Greenland	833.99(480.90,1322.73)	0.46(0.34,0.59)	1453.67(837.93,2303.12)	-0.33(-0.41,-0.24)	1319.32(891.05,1943.18)	1596.15(767.55,2782.99)	0.826564315
Grenada	1343.11(800.57,2140.81)	0.87(0.74,1.00)	1304.44(783.87,2062.83)	0.02(-0.01,0.04)	1236.87(814.23,1844.07)	1382.29(743.04,2319.89)	0.894793767
Guam	2027.31(1102.31,3382.01)	1.59(1.56,1.63)	1155.60(624.72,1937.00)	-0.37(-0.44,-0.30)	1082.34(639.01,1731.57)	1231.69(598.26,2173.52)	0.878740387
Guatemala	202763.34(108982.35,340647.38)	3.10(3.05,3.16)	1337.56(758.75,2188.46)	-0.33(-0.36,-0.30)	1196.20(749.25,1876.04)	1465.81(752.13,2484.76)	0.816065616
Guinea	137420.42(73317.41,225387.06)	2.38(2.34,2.42)	1453.20(822.92,2336.38)	0.20(0.18,0.21)	1400.60(864.05,2165.02)	1495.09(786.11,2539.93)	0.936797625
Guinea-Bissau	20242.31(10632.59,34092.20)	2.12(2.07,2.16)	1485.84(851.34,2360.91)	-0.01(-0.05,0.03)	1480.88(933.09,2237.53)	1486.20(773.98,2487.82)	0.996419544
Guyana	9729.50(5545.35,15614.20)	0.46(0.39,0.53)	1397.14(829.33,2214.73)	0.04(-0.02,0.09)	1348.53(888.64,2009.36)	1449.02(786.65,2408.86)	0.930648133
Haiti	140310.43(75821.31,230690.38)	2.01(1.92,2.10)	1383.63(805.10,2181.71)	-0.27(-0.36,-0.19)	1274.29(788.48,1940.30)	1481.56(805.45,2459.43)	0.86009683
Honduras	113970.85(60505.00,187488.51)	2.66(2.64,2.69)	1454.14(843.30,2356.77)	-0.08(-0.09,-0.06)	1260.59(800.33,1964.49)	1630.34(869.30,2713.50)	0.77320724

Hungary	179573.55(110299.35,288995.19)	0.51(0.45,0.57)	1259.93(729.00,2006.84)	-0.23(-0.27,-0.20)	1114.27(688.85,1702.29)	1401.51(754.89,2328.42)	0.795048472
Iceland	6222.93(3633.49,9792.95)	1.77(1.72,1.82)	1385.44(748.56,2286.27)	0.01(-0.01,0.02)	1184.10(716.18,1819.07)	1594.74(787.55,2791.86)	0.742504032
India	15048914.28(7681834.44,25374945.68)	1.86(1.82,1.90)	1194.11(654.37,1980.84)	-0.26(-0.30,-0.22)	1096.08(648.42,1735.71)	1298.11(664.08,2217.08)	0.844371273
Indonesia	2760154.16(1196187.44,5106367.76)	1.79(1.75,1.83)	1203.12(594.14,2100.31)	0.16(0.15,0.16)	1042.82(558.92,1759.30)	1354.64(608.77,2478.33)	0.769807958
Iran (Islamic Republic of)	1103459.21(584174.16,1853014.02)	2.22(2.15,2.30)	1394.23(785.78,2300.94)	-0.15(-0.17,-0.13)	1242.31(750.95,1967.29)	1550.80(797.00,2652.89)	0.801080247
Iraq	437762.76(204665.06,786449.59)	3.10(3.00,3.20)	1331.77(715.60,2250.90)	-0.09(-0.10,-0.08)	1201.56(684.56,1925.68)	1473.57(731.74,2578.20)	0.815408344
Ireland	84027.85(49394.10,135126.96)	1.74(1.70,1.78)	1403.66(765.90,2318.46)	-0.08(-0.10,-0.06)	1165.61(705.12,1825.30)	1631.43(811.55,2863.22)	0.714472443
Israel	134694.10(74433.16,225197.13)	2.62(2.57,2.68)	1313.05(669.40,2228.01)	-0.03(-0.04,-0.02)	1092.24(632.06,1758.75)	1531.39(700.25,2740.99)	0.713234057
Italy	1486509.52(865620.70,2433499.48)	1.75(1.69,1.82)	1472.37(739.86,2566.84)	0.27(0.23,0.31)	1207.95(678.19,1982.57)	1737.10(786.22,3272.23)	0.695387848
Jamaica	37408.97(20459.15,61173.49)	1.35(1.30,1.41)	1223.23(670.65,2006.49)	0.10(0.04,0.15)	1097.48(645.54,1698.27)	1349.20(679.47,2303.68)	0.813431608
Japan	3175900.61(1925287.86,5384485.70)	3.11(3.02,3.20)	1068.97(625.35,1750.84)	0.40(0.35,0.45)	935.90(570.64,1498.05)	1193.74(643.94,1980.44)	0.784011132
Jordan	118078.80(54388.34,213589.27)	4.32(4.00,4.64)	1291.82(687.68,2194.39)	-0.23(-0.26,-0.19)	1147.35(647.04,1865.07)	1465.64(727.98,2611.74)	0.782832461
Kazakhstan	220364.98(122123.44,350577.03)	0.53(0.45,0.61)	1324.24(760.36,2077.60)	-0.05(-0.15,0.06)	1241.80(782.26,1874.11)	1414.92(757.24,2331.97)	0.877650443

Kenya	403690.45(237901.04,640596.38)	3.17(3.14,3.20)	1155.34(707.85,1808.01)	0.20(0.17,0.23)	1087.49(707.66,1643.88)	1216.83(701.94,1916.25)	0.893704255
Kiribati	1335.17(747.96,2151.17)	1.69(1.65,1.73)	1613.40(982.72,2536.22)	-0.15(-0.18,-0.12)	1533.56(999.72,2289.21)	1658.64(941.50,2734.95)	0.924586115
Kuwait	49271.51(22503.01,88625.27)	4.10(3.81,4.40)	1294.92(693.83,2173.19)	-0.17(-0.21,-0.13)	1157.51(656.09,1921.65)	1457.59(740.84,2539.73)	0.794125297
Kyrgyzstan	73990.45(42444.14,118150.60)	1.05(0.91,1.19)	1334.80(798.90,2111.54)	-0.13(-0.26,0.00)	1262.88(829.53,1895.01)	1405.38(768.06,2364.45)	0.898608404
Lao People's Democratic Republic	70563.95(29123.55,131651.38)	2.07(2.03,2.11)	1225.63(605.93,2152.78)	-0.15(-0.18,-0.12)	1077.91(578.68,1813.09)	1365.77(607.80,2510.55)	0.789228043
Latvia	37097.09(23056.20,59167.82)	-0.21(-0.27,-0.16)	1259.72(758.39,1994.28)	-0.15(-0.24,-0.05)	1134.33(719.11,1705.29)	1386.74(763.81,2304.56)	0.817986667
Lebanon	69764.90(36993.62,117532.97)	2.44(2.33,2.54)	1349.31(721.70,2279.85)	-0.12(-0.15,-0.09)	1190.99(687.60,1930.58)	1495.19(750.22,2625.81)	0.796544739
Lesotho	21698.16(11927.87,35315.37)	1.19(1.12,1.26)	1296.30(757.00,2020.01)	0.57(0.51,0.64)	1275.71(807.66,1877.69)	1297.24(701.01,2092.70)	0.983403052
Liberia	48800.71(24348.12,85851.83)	2.94(2.78,3.09)	1347.20(743.91,2235.54)	-0.12(-0.22,-0.02)	1267.32(745.85,2051.85)	1428.22(737.48,2432.97)	0.887339425
Libya	84589.82(43792.09,145103.86)	2.47(2.41,2.53)	1378.20(759.17,2303.78)	-0.08(-0.11,-0.05)	1232.34(730.68,1974.00)	1534.09(783.02,2654.94)	0.803301225
Lithuania	53950.00(33248.87,85166.77)	0.15(0.08,0.22)	1242.40(747.95,1967.03)	-0.11(-0.19,-0.03)	1117.46(733.89,1703.59)	1366.16(767.05,2248.98)	0.817956307
Luxembourg	11043.40(6572.91,17176.60)	1.91(1.81,2.02)	1365.91(753.22,2212.94)	-0.13(-0.14,-0.12)	1152.64(689.64,1798.23)	1583.16(820.72,2681.09)	0.728064374
North Macedonia	30399.23(18048.81,48094.12)	0.94(0.88,0.99)	1257.62(730.78,2005.55)	-0.10(-0.11,-0.08)	1117.49(686.10,1724.19)	1403.82(761.65,2340.58)	0.796035444

Madagascar	187805.16(100276.32,309846.96)	2.33(2.22,2.44)	1019.71(594.15,1580.10)	-0.28(-0.32,-0.24)	936.08(573.02,1427.89)	1098.36(622.75,1775.84)	0.852251743
Malawi	140233.32(77548.19,224489.89)	2.22(2.07,2.36)	1123.54(672.19,1758.48)	-0.01(-0.04,0.03)	1081.49(681.07,1637.86)	1152.40(643.91,1887.08)	0.938473756
Malaysia	336118.25(159212.37,591776.01)	2.66(2.56,2.75)	1182.77(612.01,2045.74)	0.05(0.03,0.08)	1085.73(592.17,1789.19)	1286.31(615.28,2305.43)	0.844066528
Maldives	5446.43(2401.91,9835.79)	2.97(2.76,3.17)	1262.23(668.91,2141.33)	-0.48(-0.52,-0.44)	1190.22(674.92,1907.74)	1374.65(624.10,2509.02)	0.865834837
Mali	219705.89(114397.32,364478.39)	3.22(3.12,3.32)	1390.32(777.26,2291.03)	0.01(-0.02,0.04)	1308.05(787.49,2063.11)	1471.81(767.43,2531.98)	0.888735706
Malta	8455.91(5064.74,13378.01)	1.78(1.77,1.79)	1329.17(711.09,2221.84)	0.04(0.03,0.06)	1121.60(668.30,1753.57)	1542.75(729.14,2747.09)	0.727014826
Marshall Islands	571.46(285.60,978.10)	1.25(1.16,1.34)	1383.71(817.56,2268.67)	-0.10(-0.13,-0.08)	1318.86(844.46,2033.01)	1452.50(781.85,2485.97)	0.907996033
Mauritania	42114.76(20763.00,71574.37)	2.28(2.22,2.35)	1356.89(737.35,2246.21)	-0.19(-0.24,-0.15)	1239.48(701.08,1982.79)	1469.48(767.23,2465.38)	0.843478751
Mauritius	20247.87(11130.66,33210.83)	1.69(1.62,1.77)	1423.87(780.83,2347.01)	0.25(0.22,0.28)	1331.16(799.92,2080.38)	1503.16(753.28,2640.41)	0.885572285
Mexico	1641647.29(960343.33,2658181.24)	1.87(1.83,1.92)	1358.36(815.02,2188.77)	-0.12(-0.18,-0.06)	1216.62(806.62,1866.27)	1493.29(813.34,2492.37)	0.814723587
Republic of Moldova	56101.88(33863.46,86414.77)	-0.03(-0.08,0.02)	1202.17(707.96,1934.41)	-0.48(-0.53,-0.43)	1046.00(663.18,1617.30)	1352.26(746.47,2254.09)	0.773519486
Mongolia	36802.67(19566.16,60797.70)	1.85(1.80,1.89)	1330.70(772.76,2107.62)	-0.11(-0.16,-0.05)	1226.58(772.19,1856.28)	1438.40(774.25,2424.66)	0.852739996
Montenegro	9421.01(5591.64,14953.50)	0.80(0.77,0.83)	1241.88(709.42,1998.48)	0.02(0.00,0.04)	1083.13(657.57,1678.27)	1404.60(761.10,2330.28)	0.771130159

Morocco	435807.13(225060.88,747805.31)	1.90(1.84,1.96)	1355.83(744.58,2284.87)	0.08(0.05,0.10)	1210.86(702.09,1980.81)	1502.32(766.95,2603.73)	0.805993421
Mozambique	241668.48(133686.92,386723.74)	2.93(2.83,3.03)	1254.30(736.71,1963.01)	0.34(0.29,0.40)	1248.01(787.55,1916.27)	1261.68(695.14,2070.78)	0.989169965
Myanmar	631542.24(290515.12,1120547.29)	1.54(1.51,1.58)	1282.29(639.54,2214.92)	0.04(0.01,0.06)	1185.60(663.00,1921.21)	1360.23(598.16,2483.24)	0.871617983
Namibia	23766.37(12775.44,39623.13)	2.16(1.97,2.34)	1265.36(734.48,2029.66)	0.11(0.03,0.19)	1230.49(759.55,1901.75)	1293.85(692.45,2193.62)	0.951030881
Nepal	348568.06(175382.31,594930.48)	1.64(1.56,1.72)	1317.13(713.24,2197.94)	-0.10(-0.19,0.00)	1182.53(677.05,1918.09)	1436.13(749.86,2426.44)	0.823414279
Netherlands	345973.89(214814.33,551128.81)	1.30(1.25,1.35)	1413.97(787.23,2320.06)	0.18(0.14,0.22)	1195.12(735.52,1877.71)	1631.43(830.96,2863.99)	0.732562615
New Zealand	76573.05(47925.17,121076.96)	1.76(1.73,1.79)	1257.92(741.37,2005.44)	-0.08(-0.10,-0.06)	1130.17(717.68,1732.67)	1373.27(747.73,2271.56)	0.822976405
Nicaragua	67626.58(34864.53,113856.11)	1.98(1.92,2.04)	1251.80(709.82,2078.14)	-0.15(-0.19,-0.11)	1108.99(691.90,1768.17)	1394.22(716.33,2393.61)	0.795420369
Niger	212454.07(109283.40,364632.52)	3.33(3.24,3.42)	1360.10(750.01,2253.97)	-0.04(-0.07,-0.01)	1288.85(759.73,2064.85)	1423.80(742.57,2447.89)	0.905224122
Nigeria	2262826.67(1217927.08,3736932.85)	2.96(2.88,3.05)	1443.18(848.02,2303.21)	0.16(0.10,0.21)	1380.05(864.90,2111.05)	1498.44(813.69,2507.46)	0.920992387
Democratic People's Republic of Korea	300061.84(162854.92,509075.04)	1.39(1.33,1.46)	1065.42(583.56,1790.84)	-0.30(-0.33,-0.28)	935.91(553.03,1488.00)	1172.97(584.21,2058.25)	0.797895188
Northern Mariana Islands	506.89(268.98,857.18)	0.66(0.07,1.26)	1241.14(691.01,2051.69)	-0.05(-0.07,-0.04)	1195.45(728.06,1864.98)	1291.38(644.07,2251.46)	0.925715477
Norway	105900.38(62050.32,167877.17)	1.21(1.16,1.25)	1473.88(793.70,2439.25)	0.22(0.17,0.27)	1219.14(721.49,1944.42)	1732.71(835.73,3049.81)	0.703598425

Oman	43187.11(18526.87,79808.86)	3.58(3.24,3.92)	1340.63(758.23,2231.21)	0.10(0.07,0.13)	1255.24(755.08,2022.44)	1536.90(788.59,2691.32)	0.816732913
Pakistan	2103931.88(1057851.80,3657603.32)	2.57(2.52,2.62)	1241.72(700.12,2030.04)	0.09(0.07,0.12)	1083.23(629.94,1781.88)	1406.11(780.60,2299.80)	0.770374054
Palestine	50323.67(24673.36,87487.21)	3.18(3.09,3.26)	1376.01(773.77,2255.82)	-0.23(-0.27,-0.19)	1255.32(764.75,1997.86)	1510.20(793.03,2578.93)	0.831223728
Panama	52751.14(29477.35,86461.76)	2.76(2.75,2.77)	1251.54(701.28,2046.14)	0.18(0.17,0.19)	1114.31(674.97,1725.23)	1391.17(699.28,2384.29)	0.800989261
Papua New Guinea	85404.77(39839.67,150080.09)	3.25(3.23,3.27)	1206.65(668.86,1970.92)	-0.04(-0.06,-0.03)	1150.34(699.81,1809.95)	1265.95(620.15,2191.94)	0.908681497
Paraguay	88961.84(42216.18,155895.15)	2.42(2.33,2.51)	1380.71(701.89,2377.27)	0.08(0.05,0.10)	1216.66(674.23,1976.01)	1548.76(699.94,2759.25)	0.785569358
Peru	343382.61(199243.68,553543.54)	2.08(2.01,2.15)	1017.85(594.15,1634.73)	-0.28(-0.32,-0.23)	920.84(567.23,1446.73)	1113.92(605.58,1850.44)	0.826666065
Philippines	1117390.76(492210.22,2047452.25)	2.37(2.35,2.39)	1207.94(597.60,2118.79)	-0.18(-0.26,-0.11)	1056.88(571.73,1786.58)	1354.86(609.05,2488.15)	0.780063516
Poland	725231.29(449803.47,1167942.48)	1.11(1.06,1.15)	1336.68(800.49,2121.03)	-0.08(-0.09,-0.06)	1217.43(791.66,1851.89)	1459.14(816.72,2428.57)	0.834346419
Portugal	219300.72(129273.19,357373.72)	1.57(1.54,1.61)	1291.12(668.45,2199.92)	0.00(-0.04,0.04)	1070.19(624.80,1698.84)	1499.60(694.53,2719.53)	0.713648945
Puerto Rico	64312.84(38657.15,102698.99)	1.01(0.95,1.07)	1218.15(675.99,2010.53)	-0.29(-0.32,-0.25)	1094.22(655.25,1677.73)	1336.78(674.67,2293.59)	0.818552116
Qatar	26834.32(11350.01,50198.14)	7.74(7.06,8.43)	1344.15(754.06,2196.03)	-0.06(-0.11,-0.01)	1243.12(711.87,1988.41)	1660.01(886.96,2831.79)	0.748865212
Romania	344869.08(207033.17,548582.17)	0.35(0.29,0.41)	1248.94(703.18,2034.11)	-0.23(-0.26,-0.19)	1124.56(686.54,1734.50)	1375.50(729.62,2304.83)	0.81756697

Russian Federation	2321758.99(1395960.36,3651161.12)	0.57(0.53,0.62)	1249.97(723.75,2015.67)	-0.01(-0.05,0.02)	1069.65(658.67,1685.88)	1422.48(782.56,2389.00)	0.751964599
Rwanda	98188.67(55802.40,158479.75)	2.26(1.96,2.57)	1117.72(669.13,1753.84)	-0.24(-0.26,-0.22)	1031.41(653.55,1584.88)	1183.89(676.17,1910.74)	0.871208774
Saint Lucia	2637.94(1546.81,4149.45)	1.65(1.63,1.68)	1376.46(799.96,2191.67)	-0.25(-0.31,-0.19)	1307.81(848.19,1926.26)	1453.00(762.70,2428.84)	0.90007725
Saint Vincent and the Grenadines	1599.31(967.02,2552.30)	0.75(0.70,0.80)	1345.37(807.18,2151.34)	-0.11(-0.16,-0.07)	1281.53(837.75,1915.66)	1412.93(753.69,2383.93)	0.906996152
Samoa	2167.22(1120.92,3685.28)	1.14(1.10,1.18)	1280.61(713.44,2124.99)	-0.21(-0.24,-0.18)	1162.16(693.21,1865.17)	1395.78(709.45,2384.85)	0.832624487
Sao Tome and Principe	2258.44(1127.13,3829.43)	2.07(2.00,2.14)	1386.93(772.50,2265.30)	0.16(0.13,0.19)	1295.29(763.65,2022.19)	1472.62(759.00,2490.22)	0.87958195
Saudi Arabia	410451.40(195196.40,722127.31)	3.40(3.34,3.47)	1428.39(794.93,2355.59)	0.01(-0.03,0.05)	1334.74(780.38,2101.43)	1570.32(817.34,2646.84)	0.849979934
Senegal	163459.26(82574.55,274730.81)	2.57(2.48,2.67)	1433.43(798.31,2328.90)	0.10(0.04,0.16)	1390.32(839.40,2198.87)	1473.45(757.00,2509.18)	0.943576981
Serbia	142223.80(87516.58,218226.09)	0.31(0.26,0.36)	1257.01(733.88,1982.71)	-0.26(-0.28,-0.23)	1109.80(686.57,1663.63)	1407.48(767.26,2329.22)	0.78849611
Seychelles	1300.79(656.76,2277.98)	1.56(1.53,1.59)	1270.34(658.43,2183.19)	-0.07(-0.08,-0.05)	1165.78(685.24,1928.50)	1379.68(625.56,2539.79)	0.844964024
Sierra Leone	90197.66(46703.21,149850.33)	3.17(3.00,3.35)	1403.38(789.60,2241.48)	0.13(0.10,0.16)	1339.90(801.57,2060.20)	1461.32(761.34,2486.93)	0.916907862
Singapore	60509.74(34739.10,99171.46)	3.46(3.30,3.62)	916.57(518.16,1514.41)	0.09(-0.02,0.20)	819.29(495.65,1314.83)	1012.18(544.07,1721.41)	0.809429261
Slovakia	89803.70(53331.42,142686.48)	0.88(0.80,0.95)	1281.09(739.13,2049.40)	0.02(-0.04,0.08)	1153.24(699.28,1775.19)	1406.53(749.84,2354.95)	0.819918732

Slovenia	41442.89(25649.36,67753.61)	1.35(1.30,1.39)	1249.46(727.19,2024.90)	-0.15(-0.18,-0.12)	1111.27(685.87,1761.41)	1398.96(758.36,2368.21)	0.794354148
Solomon Islands	6149.02(3116.49,10505.64)	2.58(2.47,2.68)	1356.54(805.35,2155.54)	0.03(0.01,0.05)	1319.91(840.34,2020.01)	1395.63(750.25,2345.41)	0.945747497
Somalia	140225.51(75439.35,225681.79)	3.62(3.58,3.67)	1088.26(650.09,1708.31)	0.13(0.10,0.17)	1042.89(657.16,1613.17)	1124.28(624.50,1810.75)	0.927606168
South Africa	616963.52(350294.27,989492.62)	1.41(1.20,1.63)	1238.61(735.18,1964.94)	-0.24(-0.37,-0.11)	1176.22(746.01,1808.69)	1290.44(715.18,2115.01)	0.911483437
Republic of Korea	726908.02(424600.22,1176642.37)	1.93(1.82,2.03)	1052.53(594.57,1718.93)	-0.38(-0.42,-0.35)	925.99(561.98,1485.56)	1171.68(616.36,1975.58)	0.790315081
South Sudan	71249.29(39152.46,115353.19)	1.58(1.39,1.77)	1100.60(648.84,1761.10)	-0.05(-0.07,-0.03)	1028.24(620.48,1600.79)	1171.01(666.73,1887.16)	0.878077768
Spain	966382.13(573416.63,1539890.07)	1.68(1.52,1.83)	1349.53(700.09,2271.76)	-0.11(-0.16,-0.07)	1091.39(623.24,1758.33)	1600.38(762.76,2814.28)	0.681955195
Sri Lanka	288592.28(147962.84,492589.71)	1.45(1.41,1.49)	1301.78(678.23,2211.47)	-0.04(-0.06,-0.02)	1168.03(660.93,1902.62)	1417.54(665.68,2550.89)	0.823986517
Sudan	420276.65(204679.53,745903.95)	2.03(1.99,2.07)	1353.83(761.54,2268.16)	-0.24(-0.27,-0.20)	1227.47(728.55,1957.95)	1476.79(743.79,2603.75)	0.831174825
Suriname	7868.35(4632.15,12503.68)	2.04(1.95,2.13)	1374.37(815.53,2188.34)	0.00(-0.06,0.06)	1288.95(838.17,1926.60)	1461.82(777.60,2435.71)	0.88174645
Eswatini	11589.83(6342.99,18736.15)	1.71(1.43,2.00)	1332.89(807.53,2096.41)	0.25(0.11,0.39)	1364.56(892.36,2000.66)	1293.24(713.72,2169.04)	1.055145039
Sweden	199868.85(119046.35,320867.73)	0.85(0.82,0.87)	1320.55(692.19,2210.18)	-0.08(-0.10,-0.06)	1071.49(633.07,1695.54)	1575.77(736.63,2790.38)	0.679976399
Switzerland	170294.60(103800.85,270010.47)	1.40(1.34,1.45)	1275.01(699.20,2093.69)	-0.09(-0.10,-0.07)	1048.17(628.51,1668.69)	1502.72(757.29,2545.57)	0.697515584

Syrian Arab Republic	165256.97(80674.22,293562.48)	1.40(0.95,1.85)	1327.06(692.36,2305.80)	0.04(-0.01,0.08)	1153.50(657.66,1910.55)	1485.37(728.99,2643.41)	0.776572485
Taiwan (Province of China)	352953.83(204220.89,583726.74)	2.50(2.44,2.57)	1104.21(609.74,1853.16)	0.34(0.28,0.39)	969.75(579.22,1547.46)	1234.20(627.15,2178.15)	0.785732744
Tajikistan	110507.97(64033.41,176061.91)	1.96(1.89,2.03)	1543.39(960.89,2384.68)	0.23(0.15,0.30)	1487.44(1001.68,2185.03)	1610.67(944.58,2606.59)	0.923489667
United Republic of Tanzania	449029.50(252668.48,707803.09)	3.02(2.98,3.05)	1137.50(667.64,1772.89)	0.14(0.12,0.16)	1029.77(633.51,1580.07)	1229.24(685.60,1970.19)	0.837734429
Thailand	1088486.11(544141.16,1907980.87)	1.79(1.71,1.87)	1302.44(627.85,2298.74)	-0.18(-0.23,-0.14)	1125.04(605.15,1840.45)	1465.43(626.31,2755.89)	0.767714099
Bahamas	4902.32(2729.60,8051.46)	1.86(1.83,1.90)	1297.76(744.64,2154.46)	-0.06(-0.08,-0.04)	1197.44(747.34,1888.28)	1397.29(728.77,2378.79)	0.856974113
Gambia	22685.03(11624.16,38681.44)	3.24(3.14,3.33)	1389.21(782.82,2243.04)	0.23(0.17,0.29)	1338.77(807.42,2090.19)	1431.96(755.63,2391.69)	0.934925613
Timor-Leste	12354.78(5073.69,23165.53)	2.35(2.15,2.54)	1203.90(568.72,2126.42)	0.11(0.04,0.18)	1043.59(533.49,1762.98)	1365.26(607.07,2534.14)	0.764387198
Togo	81862.43(41606.96,139622.32)	2.60(2.52,2.68)	1398.13(797.83,2250.64)	-0.15(-0.19,-0.11)	1348.54(823.81,2083.55)	1437.03(758.39,2389.53)	0.938423942
Tonga	1082.95(549.08,1850.07)	0.91(0.82,1.00)	1220.74(641.00,2050.87)	-0.01(-0.05,0.02)	1129.70(657.72,1815.01)	1300.22(618.50,2278.71)	0.868850099
Trinidad and Tobago	19936.96(11755.33,31494.63)	1.10(1.01,1.18)	1299.09(752.61,2093.29)	-0.18(-0.22,-0.14)	1213.13(756.84,1827.48)	1390.83(736.32,2380.77)	0.872232892
Tunisia	154374.66(80216.53,263164.03)	1.85(1.77,1.93)	1332.58(699.80,2234.86)	-0.08(-0.09,-0.08)	1177.14(663.26,1943.27)	1484.51(737.93,2595.84)	0.792946376
Turkey	1231990.53(709137.78,2000450.97)	1.42(1.38,1.45)	1477.44(862.48,2368.43)	-0.29(-0.32,-0.25)	1313.79(831.13,1981.59)	1641.84(883.87,2741.15)	0.800192613

Turkmenistan	58871.12(33332.79,96591.14)	1.53(1.47,1.60)	1340.21(795.99,2145.66)	0.03(-0.02,0.07)	1250.69(788.98,1915.14)	1444.34(802.68,2393.86)	0.865927898
Uganda	313117.08(163583.29,505844.80)	3.08(3.03,3.12)	1153.02(675.57,1817.43)	0.11(0.08,0.15)	1088.62(677.55,1668.13)	1203.17(662.28,1945.24)	0.904790677
Ukraine	767245.79(472049.39,1223062.03)	0.09(0.03,0.15)	1324.30(787.37,2100.55)	0.02(-0.05,0.08)	1191.34(769.87,1815.21)	1455.29(805.07,2376.14)	0.818628571
United Arab Emirates	104945.15(51492.59,182387.54)	6.82(6.28,7.37)	1432.05(843.20,2254.35)	-0.30(-0.38,-0.22)	1382.74(844.33,2097.87)	1570.34(833.29,2691.98)	0.880535336
United Kingdom	1335367.99(824435.63,2080222.69)	1.05(1.01,1.09)	1433.83(825.52,2318.39)	0.03(0.01,0.05)	1215.30(763.03,1856.22)	1651.66(872.19,2769.33)	0.73580395
United States of America	5882170.99(3596394.04,9292478.95)	1.42(1.39,1.46)	1341.14(757.68,2179.79)	0.01(-0.04,0.06)	1119.73(720.17,1725.15)	1559.87(781.76,2698.30)	0.717836927
Uruguay	54320.17(33931.49,87043.36)	1.17(1.14,1.20)	1178.88(716.59,1862.66)	0.11(0.08,0.15)	1079.05(707.33,1629.28)	1274.87(708.76,2086.82)	0.846402444
Uzbekistan	366589.56(202877.28,597665.54)	1.69(1.49,1.89)	1412.07(861.87,2217.95)	0.20(0.04,0.36)	1318.96(856.15,1986.46)	1504.63(860.08,2460.96)	0.876603193
Vanuatu	2838.09(1437.01,4927.88)	2.83(2.80,2.86)	1308.24(747.70,2196.65)	-0.05(-0.10,-0.01)	1277.31(793.05,1995.00)	1340.29(686.56,2358.84)	0.953012874
Venezuela (Bolivarian Republic of)	364342.17(200830.42,599949.33)	2.21(2.13,2.30)	1296.36(717.43,2115.49)	-0.14(-0.19,-0.08)	1141.43(685.71,1789.99)	1445.93(726.80,2482.63)	0.789410931
Viet nam	1189643.59(567587.61,2139833.57)	2.28(2.18,2.37)	1295.37(657.81,2262.57)	0.23(0.20,0.26)	1155.79(625.50,1914.31)	1420.47(661.06,2586.12)	0.813666832
United States Virgin Islands	1714.95(1072.23,2635.63)	1.14(1.07,1.20)	1326.21(769.83,2112.35)	0.00(-0.03,0.02)	1307.32(839.69,1897.67)	1354.40(703.45,2286.13)	0.965241536
Yemen	298356.87(137796.45,541512.44)	3.09(3.06,3.11)	1324.53(723.36,2244.20)	-0.06(-0.08,-0.03)	1182.03(690.71,1975.79)	1467.54(752.81,2597.86)	0.805455288

Zambia	156180.34(85083.92,255167.98)	2.60(2.49,2.71)	1246.91(755.89,1927.06)	-0.19(-0.23,-0.15)	1206.06(772.00,1816.63)	1286.84(737.79,2060.30)	0.937226527
Zimbabwe	145128.67(81615.42,236382.10)	1.70(1.60,1.79)	1373.84(838.53,2144.28)	0.26(0.19,0.33)	1388.69(922.94,2068.53)	1360.83(759.48,2243.73)	1.020471466
Monaco	832.44(505.06,1339.12)	1.09(1.00,1.18)	1319.59(695.89,2227.79)	0.12(0.09,0.15)	1095.41(630.61,1779.32)	1535.64(719.40,2755.03)	0.713328541
San Marino	596.44(316.88,1000.89)	2.09(2.00,2.17)	1228.94(586.72,2139.70)	0.01(0.00,0.03)	959.18(486.74,1601.61)	1471.28(643.21,2708.31)	0.651939312
Saint Kitts and Nevis	824.16(484.04,1279.70)	1.21(1.17,1.26)	1391.67(833.68,2153.32)	-0.37(-0.45,-0.29)	1336.02(891.56,1969.49)	1452.91(789.29,2405.67)	0.919553032
Cook Islands	239.12(134.39,388.45)	0.77(0.72,0.82)	1179.15(637.21,1931.91)	-0.33(-0.38,-0.28)	1105.66(661.32,1727.31)	1244.76(597.23,2143.45)	0.888249981
Nauru	98.72(50.32,171.98)	-0.09(-0.20,0.02)	1437.23(882.45,2254.14)	-0.25(-0.28,-0.23)	1434.05(942.98,2109.15)	1446.97(794.12,2488.69)	0.991069977
Niue	24.13(13.76,38.53)	-1.15(-1.36,-0.94)	1294.17(708.72,2121.22)	-0.32(-0.34,-0.30)	1236.84(750.93,1897.50)	1348.99(669.60,2326.44)	0.916857934
Palau	203.53(105.94,346.33)	0.74(0.46,1.03)	1170.36(647.23,1945.91)	-0.18(-0.21,-0.15)	1096.17(652.22,1737.74)	1254.88(616.09,2202.33)	0.873525845
Tokelau	15.88(8.50,26.52)	-0.85(-1.03,-0.67)	1246.44(680.06,2060.90)	-0.29(-0.31,-0.28)	1102.81(626.94,1763.07)	1392.77(722.79,2389.06)	0.791809171
Tuvalu	133.92(71.91,230.14)	0.96(0.89,1.02)	1307.87(733.84,2180.87)	-0.32(-0.34,-0.29)	1227.72(749.19,1936.88)	1387.58(710.16,2419.04)	0.884789148

Abbreviations: CI, confidence interval; EAPC, estimated annual percentage change; SDI, socio-demographic index; UI, uncertainty interval.

Table S8. The age-standardized DALY rate of neurological disorders in 1990 and 2019 and its temporal trends, by countries

	Absolute numbers		Age-standardized rate				
	2019(95% UI)	EAPC(95% CI) (1990-2019)	2019(95% UI)	EAPC(95% CI) (1990-2019)	males, 2019 (95% UI)	females, 2019(95% UI)	male-to-female ratio
Afghanistan	1102.00(983.87,1235.85)	1.64(1.50,1.77)	10.46(9.49,11.57)	-0.07(-0.10,-0.04)	12.99(11.68,14.40)	8.09(7.19,9.16)	1.605840647
Albania	559.47(492.06,626.12)	3.27(3.22,3.32)	12.58(11.14,14.01)	0.21(0.18,0.24)	15.61(13.32,17.97)	9.98(8.89,11.19)	1.563448899
Algeria	3214.99(2837.55,3683.84)	3.94(3.84,4.05)	10.96(9.75,12.34)	0.25(0.21,0.29)	12.50(10.97,14.27)	9.36(8.28,10.79)	1.335566389
American Samoa	8.08(7.15,9.01)	2.63(2.47,2.78)	18.84(16.94,20.92)	-0.15(-0.20,-0.10)	23.03(20.54,25.74)	15.23(13.38,17.29)	1.512749293
Andorra	17.43(15.11,20.03)	3.17(2.87,3.47)	12.50(10.80,14.37)	0.11(0.07,0.15)	15.72(13.71,17.99)	9.54(8.00,11.58)	1.646664019
Angola	721.33(622.25,832.44)	4.01(3.86,4.16)	8.11(7.16,9.20)	0.26(0.23,0.29)	10.14(8.91,11.65)	6.64(5.80,7.57)	1.527785705
Antigua and Barbuda	13.22(11.49,14.93)	2.38(2.24,2.53)	14.09(12.37,15.76)	0.61(0.50,0.72)	18.61(16.22,21.50)	10.24(8.99,11.89)	1.816624367
Argentina	6640.35(5998.74,7422.00)	1.90(1.83,1.96)	11.96(10.81,13.37)	0.01(-0.07,0.09)	17.84(16.08,20.30)	7.78(6.93,9.10)	2.293600065
Armenia	413.04(357.77,461.10)	2.14(1.89,2.40)	10.05(8.79,11.20)	0.27(0.21,0.34)	12.62(11.03,14.20)	8.37(7.29,9.41)	1.50672612
Australia	6598.01(5754.15,7619.36)	2.89(2.70,3.09)	15.50(13.39,17.94)	0.22(0.01,0.44)	21.35(18.51,25.26)	10.38(8.81,12.00)	2.057024599
Austria	2769.43(2455.01,3091.90)	1.98(1.91,2.05)	14.87(13.07,16.68)	0.47(0.40,0.54)	18.68(16.00,21.83)	12.03(10.52,13.56)	1.552361582

Azerbaijan	935.12(820.62,1060.89)	2.76(2.67,2.86)	13.16(11.90,14.44)	0.98(0.95,1.01)	15.56(14.03,17.39)	11.45(10.06,12.92)	1.35912871
Bahamas	44.67(40.30,50.21)	3.39(3.33,3.45)	12.62(11.41,14.16)	0.25(0.17,0.32)	16.45(14.61,19.05)	9.67(8.54,11.13)	1.702379289
Bahrain	93.55(78.11,109.68)	6.31(6.01,6.61)	13.67(12.01,15.51)	0.45(0.41,0.49)	15.99(13.78,18.52)	11.07(9.67,12.64)	1.444363268
Bangladesh	10859.47(9583.50,12350.75)	3.47(3.20,3.74)	8.92(7.98,10.11)	-0.53(-0.57,-0.49)	10.73(9.51,12.20)	6.89(6.06,7.89)	1.557971861
Barbados	63.38(57.26,70.06)	2.06(1.89,2.23)	12.77(11.58,14.06)	0.53(0.42,0.63)	15.63(13.68,17.47)	10.48(9.39,11.81)	1.491924863
Belarus	1626.98(1457.88,1865.84)	0.71(0.58,0.85)	10.13(9.10,11.61)	0.07(0.01,0.12)	14.12(12.31,16.24)	8.09(7.14,9.31)	1.745348999
Belgium	3299.01(2891.06,3623.37)	1.92(1.85,2.00)	14.04(12.16,15.71)	0.54(0.47,0.61)	18.12(16.03,20.14)	11.00(9.31,12.67)	1.647682455
Belize	29.35(26.14,32.98)	3.95(3.78,4.11)	11.77(10.46,13.12)	0.35(0.22,0.49)	14.39(12.52,16.16)	8.98(7.98,10.08)	1.602709719
Benin	447.92(396.30,503.11)	3.16(3.10,3.22)	11.17(9.97,12.43)	0.34(0.25,0.42)	12.51(11.06,14.09)	9.99(8.91,11.34)	1.252557642
Bermuda	21.11(19.45,24.37)	2.95(2.86,3.05)	15.46(14.19,17.85)	-0.02(-0.12,0.08)	21.01(18.70,24.33)	11.21(10.05,12.89)	1.873673417
Bhutan	50.29(44.44,56.33)	3.79(3.72,3.85)	9.67(8.64,10.78)	0.40(0.38,0.43)	11.55(10.27,12.97)	7.74(6.79,8.75)	1.492494836
Bolivia (Plurinational State of)	974.84(866.76,1093.73)	3.97(3.87,4.07)	11.95(10.77,13.26)	0.20(0.16,0.24)	14.70(12.86,16.88)	9.51(8.54,10.71)	1.545649078
Bosnia and Herzegovina	741.29(656.95,840.70)	1.80(1.64,1.95)	12.07(10.82,13.59)	0.07(0.04,0.09)	14.62(12.68,16.61)	10.27(9.08,11.69)	1.424403232

Botswana	107.11(93.25,120.42)	3.64(3.50,3.78)	9.89(8.78,11.00)	0.92(0.86,0.98)	12.64(11.16,14.16)	8.17(7.20,9.21)	1.546066958
Brazil	24900.54(20948.10,28992.09)	4.15(4.07,4.23)	10.84(9.09,12.62)	0.42(0.34,0.49)	13.02(10.90,15.24)	9.07(7.62,10.54)	1.435344586
Brunei Darussalam	32.89(28.38,37.64)	4.52(4.46,4.58)	14.07(12.47,15.68)	0.74(0.64,0.85)	18.36(16.18,20.50)	12.18(10.77,13.70)	1.507640647
Bulgaria	1950.83(1764.61,2212.18)	0.28(0.18,0.38)	12.49(11.31,14.05)	-0.52(-0.60,-0.43)	15.65(14.09,17.86)	10.39(9.05,11.79)	1.505497737
Burkina Faso	774.46(679.74,878.40)	2.83(2.76,2.89)	10.58(9.48,11.82)	0.36(0.29,0.44)	11.86(10.55,13.38)	9.54(8.36,11.09)	1.243214715
Burundi	294.14(256.44,337.79)	2.04(1.79,2.28)	7.99(7.11,9.00)	0.07(0.03,0.10)	9.91(8.77,11.21)	6.13(5.35,7.03)	1.617068214
Cabo Verde	39.31(34.87,43.87)	2.66(2.59,2.72)	9.81(8.68,10.88)	0.81(0.74,0.89)	11.48(10.17,13.03)	8.56(7.42,9.84)	1.341475316
Cambodia	1150.26(1012.66,1293.60)	3.83(3.69,3.96)	10.68(9.54,11.99)	0.22(0.13,0.31)	12.58(11.09,14.32)	9.44(8.22,10.84)	1.332691438
Cameroon	1193.33(1064.92,1327.66)	3.99(3.91,4.07)	12.82(11.53,14.08)	0.44(0.36,0.52)	14.46(13.05,16.27)	11.30(9.82,12.62)	1.280129375
Canada	10415.73(9199.79,11872.92)	4.04(3.63,4.46)	14.73(12.96,16.87)	1.43(0.99,1.87)	20.23(17.57,23.47)	10.00(8.85,11.61)	2.022913178
Central African Republic	128.59(111.31,147.86)	2.14(2.09,2.19)	7.57(6.74,8.54)	0.12(0.09,0.15)	9.70(8.57,10.88)	6.26(5.52,7.17)	1.54951944
Chad	478.42(423.56,540.70)	2.38(2.27,2.48)	10.55(9.50,11.76)	0.41(0.35,0.47)	11.22(9.95,12.71)	9.69(8.64,11.03)	1.15715924
Chile	3110.80(2731.67,3533.74)	3.85(3.76,3.95)	12.90(11.37,14.64)	0.50(0.43,0.57)	18.67(16.49,21.08)	8.48(7.36,9.71)	2.201404357

China	301527.40(250664.63,352350.62)	3.86(3.68,4.03)	15.27(12.82,17.78)	0.46(0.29,0.63)	19.97(16.86,23.11)	11.95(10.04,13.93)	1.671314556
Colombia	5384.25(4862.70,6051.81)	4.57(4.45,4.69)	10.11(9.13,11.40)	0.23(0.15,0.31)	13.34(11.95,15.46)	7.49(6.61,8.47)	1.779776393
Comoros	37.47(33.18,42.53)	2.76(2.74,2.78)	8.41(7.48,9.51)	0.00(-0.01,0.02)	9.97(8.73,11.54)	7.18(6.37,8.13)	1.389399379
Congo	191.17(167.67,216.34)	3.37(3.27,3.48)	8.94(8.01,9.96)	0.31(0.27,0.35)	10.91(9.63,12.39)	7.37(6.52,8.28)	1.480915733
Cook Islands	4.45(3.89,5.09)	2.52(2.46,2.58)	17.99(15.80,20.41)	-0.24(-0.29,-0.20)	22.82(19.94,26.08)	13.56(11.76,15.41)	1.682867373
Costa Rica	588.20(516.32,660.36)	4.13(4.07,4.18)	11.69(10.25,13.15)	0.23(0.19,0.27)	15.14(12.95,17.75)	8.76(7.67,9.92)	1.728362954
Croatia	1137.32(1010.52,1268.91)	1.67(1.58,1.76)	12.25(10.86,13.65)	0.24(0.20,0.28)	15.11(13.27,17.14)	10.31(9.19,11.71)	1.466265764
Cuba	2273.22(2013.11,2548.94)	3.10(2.98,3.22)	11.69(10.38,13.08)	0.83(0.72,0.94)	15.14(13.49,17.19)	8.73(7.48,9.97)	1.733624102
Cyprus	297.57(255.19,343.46)	2.96(2.78,3.13)	15.15(13.22,17.23)	-0.29(-0.32,-0.25)	19.81(17.17,22.52)	11.38(9.56,13.62)	1.740629314
Czechia	2408.16(2176.70,2622.78)	1.85(1.81,1.90)	10.83(9.73,11.84)	0.22(0.17,0.28)	14.05(12.41,15.65)	8.60(7.49,9.73)	1.633543122
Côte d'Ivoire	1001.11(879.25,1132.40)	3.81(3.66,3.96)	12.70(11.49,14.06)	0.31(0.24,0.37)	13.82(12.25,15.58)	11.44(10.17,12.83)	1.207384158
Democratic People's Republic of Korea	3550.87(3084.51,4039.00)	2.47(2.42,2.53)	11.40(9.97,12.90)	-0.19(-0.26,-0.13)	14.62(12.65,16.79)	9.87(8.50,11.38)	1.482230728
Democratic Republic of the Congo	2305.64(1984.83,2649.69)	2.85(2.76,2.94)	7.77(6.84,8.84)	0.01(-0.05,0.08)	9.77(8.56,11.11)	6.46(5.62,7.43)	1.511745495

Denmark	1579.34(1407.33,1724.64)	2.22(2.06,2.38)	13.24(11.73,14.60)	1.07(1.00,1.14)	17.41(15.44,19.08)	9.86(8.61,11.14)	1.76605652
Djibouti	39.83(33.89,46.02)	5.52(5.47,5.57)	8.46(7.43,9.67)	0.24(0.21,0.26)	9.92(8.60,11.46)	6.96(6.07,7.91)	1.425211852
Dominica	11.74(10.52,13.26)	0.66(0.55,0.78)	12.84(11.49,14.48)	0.23(0.11,0.36)	16.41(14.45,18.75)	9.71(8.52,11.06)	1.689521973
Dominican Republic	986.84(887.00,1083.51)	4.15(3.95,4.36)	11.13(10.01,12.22)	0.86(0.79,0.94)	14.39(12.82,16.04)	8.22(7.31,9.28)	1.75036674
Ecuador	1904.09(1688.73,2121.36)	5.06(4.89,5.23)	13.32(11.89,14.84)	1.29(1.22,1.36)	16.24(14.32,18.50)	10.70(9.36,12.03)	1.517076711
Egypt	6279.74(5471.05,7205.08)	2.99(2.94,3.04)	12.02(10.53,13.56)	0.34(0.31,0.36)	12.69(11.04,14.43)	11.59(10.20,13.32)	1.09475902
El Salvador	697.36(632.02,774.40)	3.24(3.12,3.35)	11.44(10.30,12.76)	0.54(0.48,0.60)	14.92(13.28,16.80)	8.87(7.81,10.01)	1.682517579
Equatorial Guinea	37.06(32.10,42.05)	4.13(3.96,4.30)	9.08(8.02,10.25)	0.75(0.70,0.79)	11.00(9.53,12.68)	7.79(6.83,8.91)	1.411377665
Eritrea	176.30(153.67,202.44)	4.19(4.15,4.24)	8.31(7.36,9.34)	0.42(0.40,0.44)	10.86(9.62,12.30)	6.89(6.05,7.85)	1.57725648
Estonia	291.50(256.93,333.36)	0.85(0.81,0.88)	10.68(9.39,12.29)	-0.18(-0.23,-0.12)	14.64(12.90,17.13)	8.42(7.17,9.73)	1.740018035
Eswatini	42.62(37.48,48.37)	2.44(2.30,2.58)	8.77(7.84,9.80)	0.26(0.21,0.31)	11.29(10.03,12.80)	7.42(6.48,8.34)	1.520589081
Ethiopia	2698.67(2267.75,3135.57)	2.58(2.46,2.69)	7.35(6.16,8.58)	-0.14(-0.17,-0.12)	8.73(7.28,10.17)	5.91(4.94,6.93)	1.478021407
Fiji	116.57(100.68,133.60)	2.65(2.54,2.76)	18.39(16.39,20.51)	-0.29(-0.33,-0.26)	24.18(21.49,27.24)	14.63(12.87,16.60)	1.652782295

Finland	1864.29(1625.55,2121.09)	2.66(2.60,2.72)	14.69(12.85,16.96)	0.67(0.64,0.70)	18.63(16.14,21.37)	11.60(10.01,14.37)	1.60552246
France	19678.20(17922.29,21849.20)	2.31(2.23,2.40)	13.95(12.64,15.66)	0.58(0.49,0.67)	18.32(16.75,20.80)	10.75(9.51,12.50)	1.70459339
Gabon	93.48(83.81,105.15)	2.30(2.22,2.37)	10.47(9.46,11.78)	0.34(0.31,0.36)	13.75(12.28,15.56)	8.22(7.34,9.27)	1.672356475
Gambia	97.80(87.09,109.83)	4.49(4.32,4.67)	11.65(10.51,12.94)	0.57(0.51,0.63)	13.02(11.63,14.77)	10.39(9.16,11.80)	1.253132042
Georgia	601.77(544.89,656.36)	-0.19(-0.34,-0.04)	9.78(8.84,10.70)	-0.23(-0.27,-0.19)	12.24(10.83,13.60)	8.26(7.38,9.13)	1.481104007
Germany	30523.17(26190.41,34957.60)	3.37(2.97,3.77)	15.31(13.11,17.40)	1.65(1.31,1.99)	19.88(16.44,24.12)	11.80(10.46,13.35)	1.685515661
Ghana	1329.92(1174.35,1482.67)	3.91(3.77,4.04)	10.17(9.13,11.25)	0.46(0.36,0.57)	12.56(11.12,14.14)	8.40(7.41,9.50)	1.494338178
Greece	3310.01(2927.66,3720.06)	2.05(1.92,2.18)	13.37(11.86,15.20)	0.27(0.22,0.33)	17.27(15.25,19.54)	10.18(8.73,11.78)	1.697560553
Greenland	10.19(8.95,11.67)	3.13(3.03,3.22)	16.90(15.12,18.94)	0.09(0.06,0.13)	21.81(19.45,24.52)	12.20(10.77,13.86)	1.787782071
Grenada	13.69(12.38,16.13)	1.93(1.71,2.16)	13.10(11.92,15.15)	0.81(0.74,0.89)	18.41(16.51,21.81)	9.26(8.42,10.50)	1.988794338
Guam	28.11(24.59,31.82)	2.96(2.82,3.10)	15.29(13.50,17.24)	-0.72(-0.77,-0.67)	20.14(17.61,22.82)	11.32(9.87,12.96)	1.779026501
Guatemala	1066.19(957.68,1191.13)	4.51(4.38,4.64)	10.18(9.15,11.26)	-0.08(-0.17,0.00)	13.51(12.07,15.21)	7.58(6.62,8.52)	1.782569212
Guinea	570.60(513.36,635.57)	2.08(1.76,2.40)	12.19(11.11,13.42)	0.58(0.49,0.66)	12.83(11.46,14.47)	11.47(10.29,12.85)	1.118076252

Guinea-Bissau	61.13(54.30,68.34)	2.01(1.91,2.11)	11.02(10.03,12.13)	0.20(0.13,0.26)	12.96(11.60,14.59)	9.49(8.42,10.53)	1.36528844
Guyana	65.52(59.74,73.22)	2.03(1.93,2.12)	11.93(10.93,13.19)	0.24(0.15,0.33)	15.23(13.61,17.22)	9.24(8.19,10.28)	1.648494889
Haiti	678.88(600.01,765.82)	3.14(3.04,3.24)	11.52(10.35,12.92)	0.47(0.39,0.55)	14.34(12.76,16.26)	9.01(8.01,10.13)	1.592615565
Honduras	632.56(556.25,712.71)	4.26(4.19,4.32)	11.33(10.03,12.68)	0.36(0.32,0.41)	15.10(13.28,17.20)	8.00(7.03,9.11)	1.886132431
Hungary	2116.89(1896.57,2342.53)	1.36(1.28,1.43)	10.42(9.34,11.65)	0.31(0.25,0.37)	13.59(12.07,15.13)	8.60(7.69,9.69)	1.580977581
Iceland	100.13(89.40,115.70)	2.90(2.86,2.93)	17.74(15.72,20.64)	0.65(0.61,0.69)	23.15(20.45,27.65)	13.09(11.19,14.76)	1.769054538
India	106902.50(89261.82,125586.59)	4.20(4.12,4.28)	10.26(8.54,11.97)	0.35(0.31,0.38)	12.35(10.30,14.42)	8.43(7.03,9.90)	1.465294982
Indonesia	20780.37(17337.36,24166.22)	3.00(2.94,3.07)	11.02(9.25,12.88)	0.29(0.23,0.34)	12.23(10.24,14.31)	9.98(8.38,11.70)	1.22562313
Iran (Islamic Republic of)	7443.57(6276.38,8663.30)	4.54(4.38,4.70)	11.24(9.41,13.21)	0.41(0.35,0.48)	13.37(11.18,15.73)	9.09(7.61,10.66)	1.470911665
Iraq	1950.61(1712.33,2206.65)	3.61(3.50,3.72)	10.00(8.83,11.26)	0.05(0.03,0.08)	12.54(10.90,14.64)	7.76(6.87,8.71)	1.615655415
Ireland	1156.44(1013.78,1328.08)	2.63(2.46,2.79)	15.15(13.29,17.41)	0.57(0.53,0.62)	19.24(16.92,22.69)	11.76(10.00,13.93)	1.635647724
Israel	1600.20(1434.83,1797.38)	3.01(2.96,3.07)	13.57(12.13,15.32)	0.05(-0.01,0.11)	17.35(15.40,19.50)	10.60(9.19,12.12)	1.636632936
Italy	22405.37(18344.87,26680.73)	0.94(0.73,1.14)	14.93(12.45,17.64)	-0.76(-0.99,-0.54)	18.56(15.44,21.93)	12.27(10.10,14.47)	1.51233386

Jamaica	329.12(300.65,361.61)	2.10(1.94,2.26)	11.03(10.09,12.19)	0.42(0.28,0.56)	14.29(12.78,16.23)	8.17(7.31,9.05)	1.74961978
Japan	34323.75(28101.40,40845.42)	3.40(3.27,3.53)	9.11(7.62,10.70)	0.49(0.38,0.60)	11.23(9.41,13.14)	7.53(6.26,8.82)	1.491674789
Jordan	563.16(491.21,642.46)	5.58(5.13,6.03)	10.25(9.06,11.64)	-0.22(-0.29,-0.16)	11.64(10.25,13.34)	8.77(7.51,10.16)	1.327162104
Kazakhstan	1777.41(1586.99,2047.16)	1.47(1.26,1.69)	11.71(10.68,13.19)	0.56(0.47,0.65)	15.38(13.95,17.46)	9.83(8.75,11.12)	1.56470521
Kenya	1424.42(1204.80,1654.21)	3.29(3.20,3.39)	7.61(6.39,8.89)	-0.01(-0.04,0.02)	10.23(8.57,11.95)	5.66(4.74,6.61)	1.805822283
Kiribati	9.26(8.10,10.60)	1.95(1.86,2.04)	17.37(15.70,19.08)	-0.03(-0.06,0.00)	25.90(23.19,28.79)	12.35(10.90,13.85)	2.097603118
Kuwait	190.22(165.11,221.95)	4.86(4.75,4.97)	8.41(7.38,9.63)	-0.44(-0.49,-0.38)	9.73(8.30,11.25)	6.32(5.49,7.37)	1.53963777
Kyrgyzstan	321.88(285.60,360.09)	0.56(0.42,0.70)	7.90(7.10,8.76)	-0.32(-0.36,-0.29)	10.67(9.63,11.79)	6.11(5.25,7.09)	1.748006081
Lao People's Democratic Republic	421.24(371.00,481.13)	2.65(2.52,2.77)	11.06(9.83,12.48)	0.18(0.11,0.24)	12.73(11.06,15.09)	9.60(8.43,10.89)	1.326762601
Latvia	428.47(391.48,471.70)	0.34(0.25,0.42)	10.28(9.23,11.52)	-0.22(-0.28,-0.16)	14.36(12.64,16.13)	8.15(7.26,9.24)	1.760927599
Lebanon	536.31(480.60,603.48)	3.93(3.75,4.12)	10.20(9.18,11.42)	0.36(0.33,0.40)	12.38(11.05,14.31)	8.45(7.43,9.59)	1.4657352
Lesotho	81.15(71.70,90.26)	0.61(0.47,0.75)	7.69(6.89,8.49)	0.18(0.12,0.24)	9.67(8.53,10.91)	6.57(5.85,7.45)	1.472672467
Liberia	172.36(151.67,194.02)	1.84(1.75,1.93)	10.53(9.39,11.80)	0.18(0.09,0.27)	11.33(9.98,13.03)	9.67(8.45,10.85)	1.171276971

Libya	514.88(456.52,571.62)	4.12(4.02,4.21)	11.25(9.95,12.53)	0.50(0.45,0.55)	13.54(11.87,15.28)	8.88(7.80,10.10)	1.524726437
Lithuania	609.14(558.25,679.53)	1.47(1.36,1.58)	10.21(9.29,11.43)	0.47(0.43,0.52)	13.78(12.47,15.50)	8.30(7.30,9.37)	1.660906984
Luxembourg	160.34(143.71,179.06)	2.42(2.34,2.49)	15.91(14.11,17.94)	0.36(0.27,0.46)	20.62(18.17,23.81)	12.33(10.62,13.96)	1.6721696
Madagascar	617.69(531.63,715.33)	2.39(2.21,2.57)	7.04(6.20,7.96)	0.04(0.01,0.07)	8.56(7.47,9.65)	5.72(5.00,6.56)	1.495958273
Malawi	506.81(445.67,573.70)	2.21(2.08,2.35)	8.10(7.19,9.13)	0.04(0.02,0.06)	10.63(9.38,12.03)	6.41(5.58,7.37)	1.658824035
Malaysia	3023.29(2532.04,3525.20)	4.13(4.02,4.24)	12.10(10.38,13.92)	0.22(0.16,0.28)	14.19(12.13,16.65)	10.02(8.45,11.60)	1.416326179
Maldives	35.21(31.07,39.64)	4.81(4.69,4.93)	13.08(11.45,14.71)	-0.04(-0.13,0.05)	15.32(13.43,17.33)	10.52(8.97,12.07)	1.456125713
Mali	731.05(647.61,812.75)	3.03(2.92,3.13)	10.48(9.46,11.57)	0.42(0.32,0.51)	10.71(9.42,11.97)	10.21(9.09,11.56)	1.04964847
Malta	151.88(134.29,170.57)	3.52(3.48,3.56)	15.57(13.86,17.66)	0.60(0.55,0.66)	20.81(18.29,24.19)	11.63(10.25,13.01)	1.790253391
Marshall Islands	5.75(4.99,6.68)	2.34(2.10,2.58)	21.83(19.71,24.11)	0.27(0.23,0.31)	26.59(23.88,29.62)	16.55(14.86,18.53)	1.60683562
Mauritania	187.39(165.67,210.89)	2.47(2.37,2.57)	10.32(9.30,11.56)	-0.04(-0.13,0.05)	10.38(9.12,11.75)	10.28(9.15,11.69)	1.010375221
Mauritius	207.75(176.34,244.70)	3.19(3.13,3.24)	12.28(10.59,14.18)	0.04(-0.01,0.09)	15.00(12.63,17.80)	10.17(8.82,11.59)	1.474733146
Mexico	13070.96(11014.57,15220.52)	4.22(4.15,4.30)	11.75(9.89,13.70)	0.41(0.34,0.48)	14.68(12.30,17.25)	9.26(7.79,10.74)	1.586482418

Micronesia (Federated States of)	11.10(9.51,12.80)	1.11(1.06,1.17)	19.83(17.61,22.02)	0.14(0.10,0.17)	25.46(22.62,28.48)	15.69(13.83,17.79)	1.622599202
Monaco	16.14(14.45,18.64)	1.58(1.55,1.60)	16.44(14.59,18.93)	0.71(0.68,0.73)	19.94(17.52,23.11)	13.62(11.77,15.98)	1.46332109
Mongolia	162.18(138.10,187.14)	1.66(1.32,2.01)	8.39(7.33,9.52)	-0.75(-0.81,-0.70)	11.09(9.60,12.67)	6.69(5.83,7.67)	1.659162738
Montenegro	132.02(115.55,149.82)	2.32(2.26,2.38)	13.14(11.64,14.76)	0.44(0.36,0.51)	16.23(14.24,18.24)	10.94(9.53,12.59)	1.483737674
Morocco	2733.06(2424.93,3049.82)	3.64(3.58,3.70)	9.84(8.84,10.91)	0.76(0.73,0.80)	12.50(11.13,13.88)	7.37(6.40,8.35)	1.696020657
Mozambique	754.61(657.99,861.55)	2.33(2.27,2.38)	8.27(7.29,9.29)	0.21(0.19,0.23)	11.18(9.93,12.63)	6.42(5.55,7.35)	1.741833643
Myanmar	5327.46(4686.90,5993.24)	2.86(2.81,2.92)	12.73(11.36,14.25)	0.43(0.39,0.47)	14.29(12.39,16.41)	11.58(10.24,13.08)	1.233400587
Namibia	119.05(105.87,134.07)	2.47(2.34,2.60)	9.50(8.49,10.58)	0.36(0.31,0.42)	12.70(11.20,14.09)	7.42(6.51,8.52)	1.711769137
Nauru	0.54(0.46,0.64)	-0.36(-0.57,-0.15)	19.44(17.41,22.01)	0.00(-0.07,0.06)	25.19(22.53,28.94)	14.19(12.39,16.32)	1.775357374
Nepal	1750.70(1541.08,1958.06)	3.97(3.89,4.04)	8.69(7.78,9.61)	0.53(0.49,0.58)	10.00(8.85,11.27)	7.54(6.75,8.50)	1.326332084
Netherlands	5293.91(4739.92,5851.00)	1.54(1.34,1.74)	14.97(13.39,16.63)	-0.28(-0.43,-0.14)	19.46(17.16,21.72)	11.36(9.76,13.11)	1.713902875
New Zealand	1094.64(899.54,1290.35)	2.93(2.85,3.02)	13.79(11.42,16.18)	0.54(0.48,0.61)	18.54(15.42,21.75)	9.66(8.00,11.43)	1.918938327
Nicaragua	381.07(334.72,436.10)	4.09(4.02,4.16)	9.20(8.21,10.53)	0.44(0.42,0.47)	11.90(10.31,13.74)	7.09(6.16,8.20)	1.677079656

Niger	605.95(526.98,686.90)	3.92(3.81,4.03)	10.13(9.08,11.25)	0.29(0.22,0.36)	10.93(9.64,12.25)	9.36(8.28,10.62)	1.168526139
Nigeria	7169.42(6021.19,8374.91)	2.47(2.38,2.56)	10.32(8.55,12.14)	0.33(0.26,0.40)	11.12(9.20,13.15)	9.59(7.91,11.38)	1.159456104
Niue	0.41(0.36,0.46)	-0.14(-0.18,-0.09)	19.19(16.86,21.53)	0.09(0.04,0.14)	23.45(20.51,26.64)	16.06(13.92,18.38)	1.459855147
North Macedonia	426.51(376.60,481.01)	2.59(2.49,2.70)	13.75(12.51,15.22)	0.46(0.42,0.49)	17.06(15.55,18.99)	11.11(9.62,12.51)	1.53625465
Northern Mariana Islands	8.88(7.42,10.34)	3.87(3.73,4.01)	20.75(18.27,23.30)	-0.39(-0.44,-0.34)	25.83(22.59,29.24)	15.96(13.99,18.12)	1.618929835
Norway	1318.87(1097.55,1564.37)	3.12(2.93,3.31)	13.62(11.39,16.04)	2.14(1.99,2.29)	16.76(14.10,19.70)	10.98(9.08,13.04)	1.525925364
Oman	212.83(185.41,244.73)	4.19(4.09,4.29)	17.61(15.55,20.08)	1.14(1.05,1.23)	23.03(20.15,26.64)	12.33(10.85,14.05)	1.868562115
Pakistan	8930.89(7528.48,10430.51)	2.29(2.08,2.51)	9.81(8.25,11.46)	0.36(0.31,0.41)	10.15(8.51,11.84)	9.48(7.94,11.08)	1.070708242
Palau	3.67(3.15,4.26)	1.96(1.81,2.11)	20.65(18.35,23.30)	-0.13(-0.19,-0.08)	25.22(22.16,28.82)	16.58(14.53,18.88)	1.520766448
Palestine	217.18(192.57,244.14)	3.56(3.46,3.67)	11.28(10.06,12.71)	0.21(0.14,0.28)	14.46(12.87,16.40)	8.93(7.79,10.20)	1.618479862
Panama	512.57(453.75,568.41)	4.32(4.27,4.37)	12.40(10.96,13.77)	0.60(0.55,0.65)	15.61(13.63,17.92)	9.39(8.29,10.48)	1.662627994
Papua New Guinea	523.41(452.93,596.54)	2.97(2.86,3.09)	13.33(11.84,14.96)	-0.20(-0.23,-0.16)	16.60(14.64,18.81)	9.93(8.73,11.46)	1.6713001
Paraguay	565.30(502.05,626.75)	3.65(3.57,3.73)	10.65(9.49,11.82)	0.43(0.36,0.50)	12.99(11.54,14.68)	8.56(7.50,9.74)	1.517996702

Peru	3962.14(3576.01,4347.91)	4.35(4.27,4.42)	12.47(11.26,13.72)	0.58(0.51,0.65)	15.66(13.89,17.46)	9.53(8.51,10.76)	1.642679417
Philippines	7872.12(6599.29,9167.73)	3.84(3.78,3.91)	11.29(9.49,13.22)	0.37(0.32,0.43)	13.38(11.24,15.63)	9.61(8.06,11.30)	1.391663272
Poland	9159.93(7928.89,10416.08)	1.60(1.53,1.67)	12.66(11.00,14.37)	-0.14(-0.18,-0.11)	16.09(13.93,18.22)	10.49(9.16,11.89)	1.534090542
Portugal	3222.04(2871.09,3620.03)	2.75(2.69,2.81)	12.82(11.47,14.43)	0.70(0.66,0.74)	16.97(14.71,19.07)	9.82(8.58,11.50)	1.728001647
Puerto Rico	867.01(761.20,984.23)	2.43(2.35,2.52)	11.52(10.03,13.13)	-0.02(-0.09,0.04)	15.24(13.19,17.74)	8.59(7.37,9.91)	1.773524724
Qatar	110.13(92.09,131.52)	8.24(7.95,8.54)	18.38(16.31,20.69)	1.01(0.89,1.13)	18.95(16.72,21.52)	16.46(14.39,18.89)	1.151723607
Republic of Korea	9064.78(8129.48,10372.13)	5.44(5.36,5.52)	10.14(9.13,11.51)	0.95(0.88,1.02)	13.26(11.67,15.17)	8.12(7.28,9.22)	1.634347025
Republic of Moldova	469.90(413.53,530.01)	-0.24(-0.35,-0.12)	8.06(7.13,9.08)	-1.26(-1.33,-1.19)	10.94(9.74,12.51)	6.27(5.40,7.25)	1.743986141
Romania	4297.48(3930.38,4886.10)	1.79(1.70,1.88)	10.98(10.02,12.43)	0.47(0.44,0.50)	14.09(12.51,16.23)	8.89(8.08,9.96)	1.584196286
Russian Federation	24227.15(20033.66,28595.35)	0.84(0.75,0.93)	10.16(8.47,11.93)	-0.04(-0.13,0.04)	14.15(11.75,16.65)	8.13(6.79,9.57)	1.741101189
Rwanda	395.33(341.24,455.36)	3.04(2.58,3.50)	7.91(6.97,9.00)	0.07(0.03,0.12)	9.95(8.71,11.29)	6.64(5.80,7.71)	1.497361553
Saint Kitts and Nevis	8.46(7.40,9.67)	0.96(0.75,1.16)	15.10(13.46,17.21)	-0.06(-0.14,0.01)	19.37(16.56,22.51)	11.52(10.03,13.12)	1.681183632
Saint Lucia	28.29(25.10,31.57)	3.46(3.38,3.53)	13.62(12.10,15.15)	0.15(0.07,0.23)	17.51(15.42,19.92)	10.57(9.25,11.77)	1.656469258

Saint Vincent and the Grenadines	15.56(13.73,17.50)	2.73(2.58,2.89)	12.01(10.67,13.38)	0.55(0.44,0.65)	14.61(12.86,16.33)	9.41(8.35,10.79)	1.551588915
Samoa	23.08(20.83,25.62)	1.73(1.60,1.86)	17.74(16.14,19.57)	0.00(-0.03,0.03)	19.88(17.75,22.15)	15.91(14.09,17.72)	1.249723772
San Marino	7.35(6.47,8.51)	2.21(2.12,2.29)	11.26(9.90,12.94)	-0.02(-0.07,0.02)	13.94(12.03,16.27)	9.09(7.85,10.72)	1.534525089
Sao Tome and Principe	9.57(8.40,10.90)	1.87(1.78,1.97)	10.92(9.70,12.32)	0.53(0.46,0.60)	11.46(10.06,13.26)	10.36(9.07,11.88)	1.10560185
Saudi Arabia	1952.36(1712.56,2220.42)	3.87(3.75,3.99)	16.00(14.38,18.20)	0.81(0.77,0.85)	19.98(17.68,22.98)	10.20(8.88,11.82)	1.958725263
Senegal	720.10(634.55,804.26)	3.53(3.41,3.65)	11.40(10.21,12.56)	0.46(0.38,0.54)	12.85(11.38,14.43)	10.07(8.88,11.39)	1.276228476
Serbia	1942.87(1752.37,2187.49)	1.79(1.66,1.91)	11.49(10.40,12.80)	0.21(0.19,0.23)	14.08(12.60,15.85)	9.53(8.54,10.68)	1.477252082
Seychelles	17.12(15.03,19.47)	2.29(2.18,2.39)	17.24(15.24,19.59)	0.40(0.34,0.46)	22.40(19.58,26.00)	13.10(11.54,14.99)	1.709930703
Sierra Leone	315.64(283.52,356.27)	2.43(2.29,2.57)	10.51(9.53,11.68)	0.58(0.52,0.64)	10.99(9.81,12.28)	9.96(8.93,11.50)	1.103325078
Singapore	696.92(618.65,785.59)	4.96(4.85,5.07)	9.29(8.36,10.40)	0.34(0.28,0.41)	12.66(11.14,14.54)	6.59(6.02,7.22)	1.92134943
Slovakia	946.68(828.89,1078.31)	1.46(1.36,1.57)	9.98(8.85,11.29)	-0.05(-0.08,-0.01)	13.08(11.56,15.09)	8.07(6.99,9.23)	1.620207268
Slovenia	492.40(445.87,542.81)	1.99(1.94,2.04)	11.08(9.97,12.36)	-0.16(-0.20,-0.11)	14.49(13.00,16.67)	8.52(7.61,9.50)	1.700292022
Solomon Islands	42.72(37.01,48.82)	2.71(2.55,2.88)	17.60(15.72,19.57)	0.00(-0.02,0.03)	23.10(20.53,25.94)	11.71(10.22,13.64)	1.973232822

Somalia	379.28(326.91,439.57)	3.54(3.42,3.66)	7.15(6.28,8.09)	0.01(-0.03,0.06)	9.41(8.31,10.76)	5.79(5.05,6.65)	1.625905321
South Africa	3649.72(3043.22,4276.39)	3.12(3.06,3.18)	9.22(7.67,10.79)	0.66(0.59,0.73)	11.85(9.88,13.92)	7.56(6.26,8.83)	1.568104993
South Sudan	248.85(214.78,287.34)	1.23(1.15,1.31)	7.86(6.91,8.98)	-0.10(-0.13,-0.07)	9.48(8.30,10.99)	6.02(5.25,6.95)	1.574120356
Spain	14445.92(12771.63,16248.64)	1.89(1.74,2.05)	14.61(12.68,16.66)	-0.04(-0.13,0.04)	19.37(17.26,21.67)	11.13(9.29,13.28)	1.74015171
Sri Lanka	3323.71(2839.88,3831.63)	3.57(3.45,3.69)	13.41(11.66,15.37)	0.40(0.33,0.46)	16.22(14.00,18.77)	11.26(9.71,13.29)	1.4405231
Sudan	1551.77(1378.73,1744.94)	2.07(1.98,2.15)	9.42(8.45,10.62)	-0.03(-0.06,0.01)	11.36(10.06,12.83)	7.09(6.26,8.14)	1.602901095
Suriname	60.13(54.11,66.56)	3.85(3.69,4.01)	10.63(9.56,11.76)	0.65(0.51,0.79)	13.57(11.89,15.26)	8.32(7.43,9.20)	1.630687188
Sweden	3191.80(2597.46,3834.37)	1.80(1.68,1.92)	14.56(12.05,17.22)	0.69(0.62,0.76)	18.36(15.29,21.69)	11.41(9.38,13.55)	1.608845288
Switzerland	2447.53(2189.46,2705.29)	2.00(1.87,2.13)	13.70(12.22,15.20)	0.29(0.21,0.38)	17.82(15.94,19.35)	10.47(9.02,12.27)	1.702234398
Syrian Arab Republic	1176.54(1034.12,1332.56)	3.62(3.45,3.79)	11.58(10.33,12.93)	0.39(0.31,0.48)	13.47(11.96,15.60)	9.55(8.35,10.80)	1.410290849
Taiwan (Province of China)	6779.08(5849.41,7443.55)	4.40(4.08,4.71)	17.00(14.79,18.61)	0.92(0.59,1.25)	20.18(17.61,21.98)	14.38(12.40,16.68)	1.403313427
Tajikistan	563.27(486.18,645.92)	2.39(2.31,2.47)	16.59(14.74,18.62)	1.61(1.51,1.72)	20.94(18.53,23.54)	13.23(11.47,15.13)	1.582753205
Thailand	11953.82(10474.25,13798.94)	3.94(3.85,4.04)	11.94(10.49,13.76)	-0.23(-0.31,-0.15)	14.33(12.56,16.59)	10.00(8.68,11.74)	1.433620164

Timor-Leste	82.24(71.40,93.78)	4.97(4.86,5.09)	10.99(9.77,12.39)	0.42(0.35,0.49)	12.16(10.64,13.70)	9.84(8.67,11.19)	1.235143234
Togo	295.89(258.37,336.81)	3.96(3.86,4.06)	10.66(9.56,11.88)	0.30(0.23,0.38)	12.24(10.91,13.65)	9.61(8.46,10.91)	1.274379949
Tokelau	0.22(0.19,0.25)	-0.61(-0.79,-0.43)	17.47(15.43,19.62)	0.07(0.04,0.10)	20.48(17.99,23.49)	14.50(12.57,16.43)	1.412708742
Tonga	11.93(10.61,13.29)	1.34(1.25,1.44)	15.57(13.85,17.35)	-0.12(-0.16,-0.08)	19.81(17.66,22.36)	12.36(10.71,14.08)	1.603365772
Trinidad and Tobago	193.24(171.68,220.33)	2.87(2.81,2.93)	10.67(9.49,12.04)	0.07(0.00,0.14)	13.68(11.94,15.96)	8.11(7.23,9.18)	1.687925225
Tunisia	1233.93(1095.80,1387.91)	3.76(3.69,3.82)	10.52(9.37,11.83)	0.43(0.41,0.44)	13.00(11.31,14.95)	8.36(7.23,9.80)	1.555870455
Turkey	10208.55(8950.93,11495.97)	4.38(4.26,4.49)	12.30(10.75,13.82)	0.78(0.70,0.87)	15.34(13.45,17.40)	9.90(8.42,11.53)	1.550169405
Turkmenistan	307.32(269.70,348.92)	2.55(2.45,2.66)	8.85(7.86,9.95)	0.26(0.23,0.29)	12.06(10.64,13.68)	6.70(5.79,7.76)	1.800703632
Tuvalu	1.63(1.44,1.85)	1.68(1.58,1.77)	17.64(15.78,19.72)	0.35(0.30,0.40)	21.34(18.87,24.19)	14.60(12.91,16.57)	1.461744077
Uganda	933.45(813.24,1069.74)	2.64(2.51,2.78)	7.81(6.88,8.86)	0.02(-0.02,0.05)	10.44(9.21,11.89)	6.05(5.23,6.96)	1.724441886
Ukraine	8573.80(7111.10,10148.51)	0.28(0.23,0.32)	10.99(9.19,12.88)	0.06(0.03,0.09)	15.50(12.85,18.21)	8.52(7.07,10.11)	1.819607144
United Arab Emirates	470.70(402.25,562.54)	7.86(7.48,8.25)	19.02(17.12,21.18)	0.58(0.49,0.66)	20.68(18.54,23.11)	15.42(13.57,17.51)	1.341195779
United Kingdom	19268.53(16383.85,22222.43)	1.06(0.98,1.13)	14.84(12.69,17.09)	-0.10(-0.19,0.00)	19.04(16.38,21.79)	11.49(9.73,13.39)	1.656952951

United Republic of Tanzania	1656.99(1498.18,1847.97)	3.04(3.00,3.09)	7.77(7.08,8.63)	0.19(0.16,0.22)	9.29(8.42,10.34)	6.51(5.88,7.25)	1.427599002
United States of America	153614.30(132411.34,176763.66)	4.75(4.10,5.40)	26.44(22.82,30.40)	2.87(2.33,3.41)	45.35(38.47,52.85)	11.08(9.87,12.42)	4.092924213
Uruguay	676.53(611.52,762.51)	1.43(1.37,1.49)	11.86(10.70,13.37)	0.25(0.18,0.31)	17.20(15.10,19.56)	8.22(7.39,9.37)	2.092106677
Uzbekistan	2001.02(1726.46,2325.20)	1.93(1.81,2.05)	13.33(12.05,14.63)	1.00(0.94,1.06)	17.53(15.92,19.33)	10.54(9.22,12.08)	1.663200259
Vanuatu	28.00(24.84,31.62)	3.61(3.42,3.80)	18.69(16.98,20.59)	-0.07(-0.11,-0.02)	23.66(21.42,26.50)	13.20(11.66,14.75)	1.792925817
Venezuela (Bolivarian Republic of)	2991.14(2678.30,3323.55)	4.51(4.47,4.55)	10.82(9.68,12.18)	0.51(0.47,0.55)	14.33(12.55,16.66)	7.91(6.95,8.90)	1.810967283
Viet nam	10831.33(9547.81,12529.16)	3.53(3.45,3.62)	12.87(11.43,14.72)	0.72(0.64,0.80)	16.29(14.17,18.56)	10.53(9.19,12.37)	1.54686547
United States Virgin Islands	31.89(28.23,36.90)	3.56(3.45,3.66)	16.89(15.16,19.01)	0.29(0.21,0.37)	23.20(20.89,26.88)	12.26(10.71,14.03)	1.892035964
Yemen	1004.75(877.59,1130.03)	4.12(4.07,4.17)	8.81(7.81,9.84)	0.52(0.47,0.57)	10.58(9.32,12.02)	7.17(6.34,8.16)	1.476540033
Zambia	491.47(432.83,550.39)	2.88(2.69,3.07)	8.89(7.93,9.86)	-0.06(-0.09,-0.02)	11.27(10.00,12.63)	6.96(6.11,7.86)	1.619595304
Zimbabwe	527.89(470.29,584.92)	1.34(1.18,1.50)	9.60(8.75,10.52)	-0.02(-0.09,0.04)	12.26(11.02,13.66)	7.90(7.07,8.81)	1.552158046

CI - confidence interval, EAPC - estimated annual percentage change, SDI - socio-demographic index, UI - uncertainty interval.

Table S9. The age-standardized incidence rate of Parkinson's disease in 1990 and 2019 and its temporal trends, by countries

	**Absolute numbers**		**Age-standardized rate**				
	**2019(95% UI)**	**EAPC(95% CI) (1990-2019)**	**2019(95% UI)**	**EAPC(95% CI) (1990-2019)**	**males, 2019 (95% UI)**	**females, 2019(95% UI)**	**male-to-female ratio**
Afghanistan	11574.28(8845.09,14866.36)	1.30(1.18,1.42)	113.79(88.66,143.34)	-0.23(-0.27,-0.19)	131.26(104.31,174.96)	97.38(70.38,133.46)	1.348003727
Albania	4033.45(3070.30,6078.68)	3.39(3.23,3.55)	91.54(69.81,137.26)	0.01(-0.13,0.16)	121.71(91.11,208.86)	67.00(53.00,84.62)	1.816695232
Algeria	21708.37(17579.30,26334.45)	3.41(3.29,3.53)	78.88(64.11,95.05)	-0.51(-0.61,-0.41)	86.41(69.17,109.33)	71.43(57.76,84.69)	1.209717473
American Samoa	56.80(40.13,65.57)	2.29(2.21,2.37)	140.03(100.25,160.68)	-0.66(-0.76,-0.56)	173.43(128.36,197.21)	111.60(74.08,136.93)	1.554005096
Andorra	108.44(83.10,147.53)	3.49(3.19,3.80)	75.67(58.03,103.72)	-0.07(-0.14,0.00)	102.93(79.31,141.90)	51.58(37.25,75.45)	1.995654927
Angola	5465.91(4370.68,6862.84)	3.73(3.57,3.90)	70.16(56.19,89.77)	0.19(0.15,0.23)	93.07(74.40,121.55)	54.25(41.51,71.49)	1.715654518
Antigua and Barbuda	88.37(77.73,99.32)	2.24(2.02,2.47)	98.95(87.22,110.57)	0.77(0.59,0.96)	131.12(114.15,149.70)	73.65(64.94,82.36)	1.780308841
Argentina	46926.04(43169.46,50248.47)	2.07(1.97,2.18)	83.36(76.69,89.30)	0.00(-0.09,0.09)	126.79(116.82,137.08)	54.61(49.26,59.44)	2.3219197
Armenia	2807.94(2405.39,3199.23)	2.79(2.44,3.14)	69.16(59.23,78.70)	0.23(0.08,0.39)	91.09(77.89,103.90)	55.05(47.03,62.75)	1.654654849
Australia	38742.45(34592.83,42772.27)	2.89(2.72,3.07)	85.60(76.53,94.76)	0.07(-0.07,0.21)	123.49(110.65,136.82)	54.02(47.51,60.86)	2.285882968
Austria	15762.05(14105.00,17355.38)	1.90(1.81,1.99)	77.00(68.98,85.32)	0.23(0.13,0.33)	107.57(96.32,119.36)	55.07(48.38,61.53)	1.953173002

Azerbaijan	5756.17(4912.65,7303.78)	3.38(3.09,3.67)	94.39(79.46,121.93)	1.79(1.59,1.99)	114.12(87.11,150.25)	81.17(67.79,107.53)	1.405907149
Bahamas	301.98(256.58,353.09)	3.39(3.33,3.46)	89.45(75.96,104.37)	0.29(0.18,0.40)	120.33(100.56,140.97)	66.65(57.19,77.45)	1.805248036
Bahrain	466.81(340.38,573.07)	4.98(4.63,5.33)	88.49(64.07,106.60)	-0.23(-0.64,0.17)	104.24(75.72,127.55)	72.33(48.95,86.81)	1.441221423
Bangladesh	76977.23(61353.35,99649.56)	3.00(2.72,3.28)	66.25(52.88,86.40)	-0.98(-1.29,-0.67)	81.43(64.07,108.24)	49.03(37.85,65.75)	1.660997255
Barbados	437.46(375.29,502.55)	2.06(1.97,2.15)	87.22(74.84,100.32)	0.69(0.51,0.88)	108.05(92.26,125.48)	71.22(60.71,82.51)	1.517266597
Belarus	10421.94(8591.36,12439.99)	1.39(1.12,1.66)	63.61(52.34,76.20)	0.35(0.15,0.55)	89.25(72.00,110.46)	52.12(43.12,62.79)	1.71235108
Belgium	20122.26(18095.05,22093.14)	1.99(1.89,2.09)	77.62(70.05,85.62)	0.30(0.25,0.35)	110.47(100.09,121.15)	53.97(47.66,60.79)	2.046694791
Belize	202.45(178.59,227.38)	4.24(3.92,4.56)	85.94(75.73,96.36)	0.77(0.34,1.21)	107.42(93.78,121.60)	63.88(55.56,72.35)	1.681479836
Benin	3190.78(2700.36,3793.07)	3.04(2.96,3.12)	86.07(73.80,100.97)	0.34(0.26,0.41)	103.32(86.11,124.10)	73.00(60.63,87.19)	1.415265132
Bermuda	123.29(106.81,144.02)	1.89(1.69,2.09)	87.70(76.01,102.57)	-1.23(-1.32,-1.13)	130.39(114.00,149.62)	56.95(47.72,68.55)	2.289684868
Bhutan	437.08(347.65,532.43)	4.47(4.35,4.60)	90.37(72.23,110.13)	0.87(0.83,0.90)	109.62(81.62,136.45)	70.64(57.04,92.52)	1.551777118
Bolivia (Plurinational State of)	9478.45(7869.65,11280.59)	4.38(4.29,4.47)	123.62(103.36,146.79)	0.53(0.48,0.57)	150.14(120.73,181.54)	100.82(82.62,125.16)	1.489259784
Bosnia and Herzegovina	5407.39(4363.73,7279.50)	1.38(1.24,1.53)	89.29(72.12,120.08)	-0.80(-0.93,-0.68)	114.54(92.60,149.46)	71.89(57.85,102.04)	1.593279748

Botswana	767.90(635.94,929.34)	3.37(3.07,3.67)	77.82(65.32,93.52)	0.68(0.40,0.97)	103.51(85.94,123.69)	62.40(50.28,80.46)	1.658762412
Brazil	159998.25(145339.50,173337.43)	4.04(3.99,4.09)	71.87(65.25,77.86)	0.13(0.08,0.18)	91.64(83.59,99.74)	56.94(50.58,62.16)	1.609472536
Brunei Darussalam	253.52(156.37,294.33)	4.46(4.27,4.65)	127.74(76.96,148.00)	0.87(0.70,1.05)	178.75(108.77,212.92)	107.01(59.14,125.02)	1.670393108
Bulgaria	15494.11(13049.34,18238.40)	1.00(0.85,1.15)	95.59(80.45,112.64)	-0.33(-0.47,-0.18)	128.55(108.21,151.15)	74.55(62.58,88.14)	1.724284386
Burkina Faso	5937.52(5012.43,7254.54)	2.78(2.66,2.91)	88.46(74.91,107.28)	0.40(0.32,0.48)	108.06(88.67,132.94)	73.32(59.65,93.74)	1.47394066
Burundi	2303.36(1751.61,3116.48)	1.23(1.01,1.46)	69.16(52.68,95.87)	-0.43(-0.51,-0.35)	87.94(66.32,120.89)	51.17(37.83,73.44)	1.71864594
Cabo Verde	355.83(310.14,395.64)	3.23(2.96,3.49)	89.54(77.90,99.24)	1.41(1.09,1.72)	123.61(107.26,135.72)	67.13(55.16,77.83)	1.841498659
Cambodia	8406.22(6685.72,9979.61)	3.82(3.69,3.96)	86.47(69.24,102.37)	0.22(0.12,0.31)	106.66(84.96,126.87)	73.81(55.90,89.82)	1.444929176
Cameroon	8668.23(7153.91,10631.90)	3.96(3.85,4.08)	102.01(85.28,123.75)	0.41(0.31,0.51)	120.60(99.06,145.19)	86.74(69.88,108.19)	1.390354616
Canada	64546.65(58314.02,70386.66)	3.39(3.15,3.63)	87.78(79.45,95.44)	0.61(0.38,0.84)	126.83(114.80,138.73)	56.44(49.86,62.72)	2.247357017
Central African Republic	1186.80(886.10,1646.57)	1.78(1.75,1.80)	75.59(57.23,101.65)	-0.10(-0.17,-0.03)	106.32(80.04,145.92)	56.57(40.62,80.03)	1.879641582
Chad	3616.11(2984.93,4352.41)	2.43(2.39,2.48)	85.73(70.66,102.96)	0.62(0.52,0.71)	96.96(78.40,121.00)	72.81(58.08,92.82)	1.331704437
Chile	20331.92(18429.26,22034.41)	3.75(3.68,3.81)	84.32(76.40,91.38)	0.25(0.18,0.32)	127.10(114.14,138.43)	53.22(47.32,58.43)	2.388085829

China	1554451.07(1333742.15,1794867.61)	3.06(2.93,3.18)	83.66(72.18,95.97)	-0.42(-0.55,-0.28)	115.23(95.98,135.82)	60.75(50.49,70.92)	1.896945787
Colombia	32267.23(26077.56,39542.00)	4.34(4.21,4.46)	60.94(49.22,74.81)	-0.29(-0.39,-0.20)	80.47(63.65,100.66)	45.40(37.14,55.18)	1.772550224
Comoros	305.07(248.51,420.39)	2.55(2.43,2.66)	70.98(58.09,99.29)	-0.14(-0.23,-0.06)	87.78(71.06,130.49)	58.22(45.70,79.81)	1.507644293
Congo	1603.11(1349.67,1941.66)	2.40(2.21,2.60)	83.38(70.28,102.94)	-0.38(-0.49,-0.27)	101.04(84.43,131.51)	69.71(56.84,86.67)	1.449398342
Cook Islands	28.33(21.88,34.23)	1.43(1.27,1.59)	114.86(88.68,139.28)	-1.50(-1.63,-1.38)	150.21(112.65,180.19)	82.91(60.99,103.40)	1.811730515
Costa Rica	3610.87(2957.13,4367.07)	3.97(3.85,4.08)	72.97(59.72,88.20)	0.02(-0.10,0.13)	95.12(77.24,116.21)	54.71(44.39,65.81)	1.738653806
Croatia	7632.74(6378.29,9103.56)	1.85(1.69,2.00)	77.01(64.25,92.02)	-0.08(-0.20,0.05)	101.79(84.15,121.60)	61.27(50.37,73.41)	1.661279742
Cuba	14651.49(12421.08,17094.30)	2.93(2.83,3.03)	73.82(62.62,86.43)	0.61(0.53,0.69)	98.61(82.60,117.79)	52.91(44.86,62.10)	1.86360578
Cyprus	1613.76(1237.98,1842.24)	1.45(1.27,1.63)	81.70(63.22,92.98)	-1.92(-2.05,-1.78)	111.94(85.62,128.70)	56.91(42.34,65.73)	1.966964292
Czechia	16264.98(13713.76,18900.47)	1.80(1.67,1.93)	70.57(59.60,82.21)	0.03(-0.06,0.12)	96.04(80.26,112.44)	53.81(45.57,63.27)	1.784713854
Côte d'Ivoire	6746.82(5656.89,7905.30)	3.62(3.43,3.82)	95.86(81.95,110.94)	0.05(-0.05,0.14)	114.75(96.12,134.90)	77.96(64.06,91.39)	1.47195535
Democratic People's Republic of Korea	26554.38(22325.44,31277.58)	2.73(2.62,2.85)	85.32(72.01,100.09)	-0.29(-0.35,-0.23)	114.02(96.88,137.31)	71.60(57.34,87.82)	1.592412757
Democratic Republic of the Congo	17405.58(13853.80,22094.44)	2.39(2.31,2.46)	64.74(51.84,82.06)	-0.40(-0.49,-0.32)	87.74(67.59,115.60)	50.52(39.70,65.03)	1.736921474

Denmark	9582.81(8639.69,10490.40)	1.95(1.82,2.07)	75.42(67.98,82.73)	0.88(0.80,0.96)	108.72(97.70,119.31)	49.48(43.92,55.64)	2.197434093
Djibouti	306.11(245.70,436.84)	5.93(5.87,5.99)	75.48(61.75,112.22)	0.69(0.61,0.77)	91.61(73.84,142.98)	59.48(46.52,84.99)	1.540111558
Dominica	89.49(74.36,105.81)	0.85(0.77,0.92)	96.14(79.64,113.58)	0.54(0.49,0.59)	124.43(101.86,149.75)	72.30(57.70,86.50)	1.721093758
Dominican Republic	7464.79(6072.00,9089.32)	5.17(4.94,5.39)	86.28(70.09,104.58)	1.76(1.57,1.95)	115.88(91.06,144.77)	60.20(48.52,73.87)	1.924924768
Ecuador	11914.39(9480.08,14602.78)	5.30(4.96,5.64)	85.70(68.23,104.99)	1.42(1.17,1.66)	108.04(82.87,133.17)	66.48(53.04,80.86)	1.625216483
Egypt	48342.99(37832.74,67874.83)	3.03(2.94,3.12)	98.59(77.11,142.38)	0.51(0.40,0.62)	97.68(74.02,151.81)	103.03(81.71,143.71)	0.948124273
El Salvador	4622.48(3721.33,5645.97)	3.24(3.02,3.45)	74.83(60.26,91.44)	0.38(0.21,0.54)	99.30(78.78,122.46)	56.99(44.67,69.74)	1.742441103
Equatorial Guinea	294.96(222.60,386.16)	3.68(3.36,4.01)	81.78(61.98,105.49)	0.67(0.53,0.80)	106.33(81.41,149.65)	65.87(47.29,87.59)	1.614292512
Eritrea	1480.87(1138.87,1986.46)	4.40(4.25,4.56)	78.96(61.20,107.96)	0.76(0.61,0.92)	101.92(74.23,136.24)	66.42(49.62,92.99)	1.53433054
Estonia	2024.78(1674.00,2421.31)	1.05(0.95,1.16)	67.16(55.68,80.72)	-0.47(-0.59,-0.36)	91.89(74.48,110.69)	55.26(45.27,66.35)	1.663024648
Eswatini	333.79(261.07,423.04)	3.01(2.72,3.30)	75.60(59.58,94.80)	0.90(0.60,1.20)	110.30(90.72,131.46)	57.74(41.09,76.74)	1.910486433
Ethiopia	22087.75(18523.33,32897.90)	1.72(1.54,1.90)	65.97(55.01,100.63)	-0.81(-0.89,-0.74)	82.63(65.34,126.29)	48.21(39.62,75.46)	1.714071503
Fiji	862.63(559.64,1070.38)	2.39(2.28,2.49)	151.39(102.74,186.57)	-0.56(-0.69,-0.43)	207.00(135.35,250.87)	115.98(75.63,146.54)	1.784817652

Finland	11509.96(10332.70,12676.21)	2.75(2.68,2.82)	83.12(74.88,91.69)	0.59(0.53,0.65)	116.15(104.72,129.21)	58.94(52.38,66.34)	1.970575503
France	117422.40(104049.61,130006.36)	2.19(2.08,2.29)	74.21(66.17,82.49)	0.17(0.06,0.28)	107.20(95.43,118.69)	50.91(43.93,57.32)	2.105772784
Gabon	786.16(628.00,967.73)	1.73(1.66,1.79)	94.78(76.14,116.74)	-0.08(-0.14,-0.02)	136.12(108.41,173.33)	67.57(52.01,87.67)	2.014444083
Gambia	790.75(640.30,957.78)	5.06(4.89,5.23)	99.80(81.78,121.04)	0.96(0.87,1.05)	116.76(94.94,138.63)	85.76(65.58,111.65)	1.361576086
Georgia	12949.35(11208.86,14884.37)	0.55(0.26,0.85)	81.07(70.28,93.11)	0.04(-0.23,0.31)	121.56(100.30,145.16)	51.90(42.58,62.07)	2.342059436
Germany	195201.93(177254.40,213119.94)	2.89(2.74,3.05)	86.42(78.85,94.67)	0.93(0.80,1.07)	122.51(111.29,134.89)	59.77(53.63,66.42)	2.049701503
Ghana	8577.85(7357.08,9890.30)	4.02(3.85,4.19)	72.85(62.01,83.59)	0.51(0.37,0.66)	98.89(80.73,117.34)	55.14(45.17,65.11)	1.793429871
Greece	22951.03(20754.46,25012.24)	2.63(2.51,2.75)	80.37(72.98,87.72)	0.29(0.26,0.33)	112.36(102.14,122.94)	55.16(49.44,61.15)	2.037015782
Greenland	70.34(50.92,84.42)	2.75(2.67,2.83)	122.09(89.93,145.84)	-0.34(-0.50,-0.18)	169.12(126.91,202.56)	77.11(52.12,95.95)	2.193283689
Grenada	86.41(78.70,95.18)	1.37(1.13,1.60)	86.88(79.21,95.49)	0.70(0.46,0.94)	114.29(102.65,126.83)	67.89(60.85,74.98)	1.683493949
Guam	177.22(139.66,211.55)	2.27(2.09,2.45)	98.79(77.87,118.20)	-1.95(-2.28,-1.63)	129.57(101.31,154.77)	73.82(53.86,89.94)	1.755291156
Guatemala	6781.69(5620.77,8135.70)	4.03(3.93,4.13)	66.13(55.23,79.23)	-1.06(-1.18,-0.93)	88.20(72.82,105.59)	49.89(41.43,58.86)	1.767799365
Guinea	4299.65(3471.76,5333.55)	2.30(1.97,2.62)	96.42(77.92,119.75)	0.75(0.65,0.84)	105.10(82.49,130.89)	88.01(67.89,112.10)	1.194264996

Guinea-Bissau	506.50(405.26,630.82)	2.00(1.86,2.15)	100.95(81.48,124.15)	0.34(0.25,0.43)	124.38(98.19,159.53)	84.18(64.85,107.47)	1.47756912
Guyana	482.37(400.64,579.47)	2.05(1.97,2.13)	93.15(77.90,110.74)	0.18(0.08,0.28)	119.62(99.06,143.13)	72.14(59.59,86.09)	1.65826263
Haiti	5305.29(3664.28,7464.95)	2.64(2.55,2.73)	96.02(66.88,134.40)	0.04(-0.03,0.11)	114.56(75.25,164.78)	79.50(55.67,107.42)	1.440953691
Honduras	6573.77(5062.38,7747.56)	5.53(5.29,5.78)	127.45(96.78,149.55)	1.66(1.45,1.88)	173.33(125.99,218.52)	86.90(63.40,108.01)	1.99460628
Hungary	14888.08(12745.43,17286.38)	1.36(1.27,1.45)	69.99(59.77,81.36)	0.06(-0.01,0.12)	97.21(82.22,113.94)	54.96(46.77,64.44)	1.768755124
Iceland	562.68(497.29,630.35)	2.55(2.45,2.65)	94.64(83.87,106.13)	0.18(0.07,0.29)	134.46(119.65,150.92)	61.60(52.76,70.06)	2.182751509
India	812974.67(697059.47,933194.97)	3.82(3.70,3.95)	82.89(71.28,95.51)	-0.32(-0.47,-0.18)	100.89(82.62,120.21)	67.39(54.26,81.73)	1.497199171
Indonesia	144663.63(116037.88,171198.34)	3.54(3.42,3.67)	87.30(70.07,102.68)	0.83(0.74,0.91)	101.97(79.42,126.67)	75.85(57.49,92.65)	1.344334053
Iran (Islamic Republic of)	48779.19(42377.87,53931.79)	4.56(4.40,4.71)	76.80(66.90,84.86)	0.07(0.02,0.12)	91.04(69.61,102.49)	62.34(54.80,69.85)	1.460462083
Iraq	14972.51(12203.67,20254.06)	3.69(3.60,3.79)	85.57(71.00,115.20)	0.35(0.28,0.42)	124.29(99.15,195.29)	52.84(41.03,61.75)	2.352094329
Ireland	6606.45(5919.73,7282.44)	2.31(2.13,2.50)	84.18(75.43,92.88)	0.23(0.19,0.27)	114.70(101.97,128.06)	59.65(52.80,66.72)	1.922742328
Israel	9436.68(8513.53,10340.00)	2.78(2.72,2.85)	77.26(69.84,84.84)	-0.30(-0.35,-0.25)	108.96(98.04,119.50)	52.79(47.18,59.53)	2.064037622
Italy	123824.73(109422.46,136077.95)	1.56(1.44,1.68)	72.97(64.95,80.60)	-0.55(-0.67,-0.42)	101.53(90.46,111.89)	52.44(45.55,59.23)	1.935916344

Jamaica	2301.70(1935.60,2729.24)	2.31(2.03,2.59)	76.58(64.30,90.90)	0.67(0.43,0.91)	97.63(81.28,116.81)	58.75(49.15,69.71)	1.661706224
Japan	239760.38(204914.63,263440.88)	3.80(3.64,3.97)	55.65(48.47,61.02)	0.35(0.27,0.43)	74.03(65.76,80.94)	42.07(35.35,47.15)	1.75996967
Jordan	3534.47(3006.76,4138.07)	4.85(4.47,5.22)	74.20(62.95,86.68)	-0.81(-0.94,-0.69)	87.39(66.62,107.31)	59.92(48.66,84.79)	1.458400022
Kazakhstan	12770.19(11231.78,14410.32)	1.91(1.75,2.07)	89.96(78.90,100.94)	0.94(0.78,1.11)	122.57(104.76,144.58)	73.98(65.15,83.40)	1.656791557
Kenya	11770.21(10011.35,14832.24)	3.89(3.82,3.96)	71.27(60.51,94.39)	0.73(0.65,0.81)	95.92(78.55,125.84)	53.26(42.85,75.83)	1.800769559
Kiribati	99.27(69.62,123.43)	1.13(0.94,1.32)	213.52(159.28,259.90)	-0.60(-0.74,-0.47)	253.07(161.39,319.79)	189.45(139.03,238.34)	1.335789519
Kuwait	1052.17(893.62,1254.98)	4.38(4.18,4.58)	56.11(47.21,66.05)	-0.90(-1.08,-0.72)	70.31(57.96,85.33)	33.95(27.84,40.57)	2.070965477
Kyrgyzstan	2039.61(1774.01,2312.61)	0.59(0.21,0.96)	56.43(49.42,63.52)	-0.19(-0.58,0.19)	77.33(66.15,90.05)	43.65(38.15,49.07)	1.771427398
Lao People's Democratic Republic	2933.46(2411.61,3427.49)	2.01(1.88,2.14)	85.74(71.62,99.10)	-0.31(-0.39,-0.23)	100.79(83.10,118.71)	73.38(57.81,87.44)	1.373539903
Latvia	2971.57(2561.27,3421.75)	0.42(0.25,0.59)	64.45(55.40,74.36)	-0.58(-0.75,-0.41)	90.46(74.60,109.91)	52.52(43.60,64.05)	1.722529173
Lebanon	3661.70(3016.41,4824.71)	3.49(3.34,3.65)	69.53(57.25,91.55)	-0.37(-0.54,-0.19)	90.37(74.08,121.77)	52.81(40.98,70.79)	1.711391484
Lesotho	701.66(564.37,862.90)	2.01(1.77,2.25)	72.56(58.50,88.30)	1.71(1.51,1.90)	93.75(80.96,111.52)	61.11(44.43,78.79)	1.534049565
Liberia	1200.36(983.74,1452.71)	1.35(1.23,1.47)	81.12(66.80,97.63)	-0.13(-0.26,0.00)	88.03(70.05,107.64)	74.26(58.72,92.56)	1.185358227

Libya	3711.59(2836.02,4719.20)	4.18(4.00,4.36)	84.83(65.34,108.35)	0.58(0.44,0.73)	100.28(76.61,131.76)	68.83(50.17,89.49)	1.456814257
Lithuania	4326.02(3679.17,5043.83)	2.19(1.93,2.45)	65.54(55.87,76.83)	0.65(0.46,0.84)	90.53(75.45,106.17)	53.50(45.47,62.96)	1.692232578
Luxembourg	890.61(771.94,1014.60)	2.19(2.08,2.31)	83.13(72.12,94.80)	-0.06(-0.22,0.10)	115.51(98.55,134.80)	59.73(51.44,68.63)	1.933881656
Madagascar	4331.33(3355.76,5796.02)	1.93(1.79,2.08)	56.81(44.17,77.37)	-0.14(-0.27,-0.01)	68.47(51.85,92.95)	46.99(35.15,65.73)	1.457101715
Malawi	4025.62(3163.98,5428.10)	2.14(2.03,2.25)	69.64(54.96,97.41)	0.04(-0.04,0.13)	97.39(76.79,131.02)	51.73(39.05,78.37)	1.882797216
Malaysia	19198.77(15896.49,23121.57)	4.18(4.02,4.33)	85.55(71.20,102.18)	0.31(0.15,0.47)	108.61(89.00,135.21)	63.22(38.18,76.57)	1.717863926
Maldives	190.59(161.66,219.67)	3.97(3.89,4.05)	78.50(66.62,90.43)	-1.16(-1.29,-1.02)	89.20(74.90,104.53)	66.28(55.22,76.91)	1.345675657
Mali	5511.27(4619.61,6474.09)	3.02(2.91,3.14)	85.62(72.20,100.45)	0.41(0.31,0.52)	88.60(71.68,107.65)	82.46(66.79,103.07)	1.074517589
Malta	843.03(746.79,949.26)	3.10(2.98,3.22)	82.36(73.00,92.83)	0.00(-0.17,0.17)	117.64(104.33,132.24)	56.64(48.71,64.53)	2.076963548
Marshall Islands	46.71(28.87,59.79)	1.50(1.19,1.80)	199.84(127.72,249.99)	-0.27(-0.38,-0.15)	226.45(142.81,286.24)	170.03(105.90,216.61)	1.33183858
Mauritania	1341.23(1033.38,1639.13)	1.85(1.63,2.07)	79.22(61.51,96.59)	-0.58(-0.72,-0.44)	80.40(62.23,99.94)	78.02(58.09,98.73)	1.030602714
Mauritius	1196.98(1016.88,1412.33)	3.03(2.93,3.13)	75.07(63.71,88.47)	-0.18(-0.27,-0.09)	89.92(75.43,107.37)	64.34(54.38,76.25)	1.39757345
Mexico	83541.13(72698.87,94787.45)	4.07(4.02,4.12)	77.08(66.96,87.47)	0.17(0.10,0.24)	98.50(82.61,114.41)	59.17(50.51,68.78)	1.664740741

Micronesia (Federated States of)	99.32(64.49,126.40)	0.62(0.49,0.76)	199.15(137.29,247.44)	-0.20(-0.29,-0.11)	239.56(155.47,301.57)	170.03(113.10,212.66)	1.408963656
Monaco	100.74(79.71,123.01)	1.70(1.51,1.88)	91.96(72.48,112.63)	0.82(0.69,0.95)	123.61(97.30,151.23)	66.97(49.60,87.04)	1.845802953
Mongolia	1063.15(872.92,1286.55)	0.57(0.29,0.86)	64.04(52.83,76.72)	-1.45(-1.77,-1.13)	91.68(75.51,114.54)	47.24(37.37,58.09)	1.940878369
Montenegro	924.76(783.03,1226.86)	2.85(2.71,2.98)	93.46(79.21,124.40)	0.80(0.72,0.88)	128.62(107.25,165.08)	69.51(58.74,99.62)	1.850397654
Morocco	25269.20(20567.64,29607.39)	4.20(4.10,4.30)	96.62(79.28,111.86)	1.25(1.08,1.41)	123.38(98.15,145.81)	72.38(57.56,85.79)	1.704619339
Mozambique	6500.96(5204.89,8732.47)	2.86(2.76,2.97)	77.84(62.87,108.04)	0.80(0.70,0.90)	114.70(93.27,150.25)	54.84(41.86,87.01)	2.091590699
Myanmar	32961.93(28258.19,38065.69)	2.89(2.72,3.06)	85.14(73.13,97.70)	0.41(0.29,0.52)	110.04(94.23,128.06)	67.98(55.92,79.79)	1.618683326
Namibia	900.41(757.77,1072.60)	2.48(2.17,2.79)	75.68(64.17,89.99)	0.43(0.23,0.63)	109.68(93.72,127.96)	54.23(42.92,68.21)	2.022526295
Nauru	4.92(2.93,6.07)	-1.33(-1.50,-1.15)	219.33(142.40,262.31)	-0.59(-0.65,-0.53)	267.29(161.37,331.99)	172.74(109.67,216.98)	1.547355462
Nepal	15289.35(11938.97,18519.54)	4.48(4.27,4.70)	82.16(64.18,100.50)	0.99(0.81,1.18)	100.04(75.95,125.82)	66.13(52.03,85.04)	1.512739429
Netherlands	30515.29(27494.66,33465.12)	1.54(1.40,1.68)	81.75(73.66,90.09)	-0.36(-0.43,-0.29)	114.54(102.98,125.82)	56.71(49.98,63.66)	2.019520236
New Zealand	6534.76(5864.76,7154.80)	2.75(2.67,2.82)	78.14(70.15,85.84)	0.26(0.17,0.35)	110.97(99.59,123.19)	50.78(44.58,56.60)	2.185194346
Nicaragua	3600.80(2886.09,4126.54)	4.46(4.09,4.82)	97.64(78.91,111.03)	0.90(0.56,1.24)	137.85(99.23,160.69)	67.84(56.87,76.50)	2.032140198

Niger	4316.14(3531.96,5259.45)	3.98(3.84,4.12)	80.90(66.88,98.34)	0.42(0.35,0.48)	90.93(71.09,114.21)	71.51(56.89,91.28)	1.271487561
Nigeria	54032.98(43668.51,65154.27)	2.99(2.83,3.14)	86.96(70.71,103.73)	0.99(0.81,1.16)	93.29(71.68,119.72)	80.72(61.67,100.00)	1.155714201
Niue	3.38(2.47,3.90)	-0.94(-1.01,-0.87)	157.07(115.19,181.56)	-0.72(-0.78,-0.66)	207.24(150.71,239.41)	122.77(84.93,154.11)	1.688003564
North Macedonia	3058.11(2481.21,3660.88)	2.67(2.56,2.79)	105.04(85.64,124.47)	0.38(0.27,0.49)	133.36(109.61,158.68)	82.85(56.83,99.79)	1.609572701
Northern Mariana Islands	52.57(36.11,61.56)	3.51(3.41,3.61)	142.82(100.82,165.82)	-0.86(-0.93,-0.79)	185.97(132.16,215.49)	103.47(68.81,127.95)	1.797369266
Norway	8024.33(7150.03,8824.68)	2.31(2.13,2.49)	77.61(69.61,85.51)	1.39(1.18,1.60)	104.25(93.38,117.00)	56.19(49.66,62.68)	1.855180764
Oman	1097.38(785.31,1265.01)	3.90(3.68,4.13)	125.33(78.98,144.47)	1.40(1.19,1.62)	169.58(103.54,202.78)	87.84(48.45,104.72)	1.930670346
Pakistan	78955.49(66921.41,94394.93)	2.20(2.10,2.30)	94.86(80.76,112.28)	0.49(0.34,0.64)	110.28(86.96,137.41)	79.27(61.02,100.63)	1.391210469
Palau	24.19(14.44,30.39)	1.25(1.11,1.39)	151.90(94.85,189.03)	-0.67(-0.74,-0.60)	184.51(114.59,229.28)	123.50(74.37,157.50)	1.494058768
Palestine	1614.10(1141.83,1855.20)	2.84(2.68,3.00)	93.09(64.93,106.78)	-0.42(-0.58,-0.25)	124.82(86.97,144.43)	70.32(42.15,81.86)	1.775052836
Panama	3082.43(2507.35,3725.77)	4.40(4.26,4.53)	74.75(60.91,90.38)	0.59(0.44,0.73)	93.78(74.86,114.72)	57.66(46.89,69.76)	1.626392768
Papua New Guinea	4189.52(2768.40,5343.11)	2.85(2.75,2.96)	124.99(87.14,155.95)	-0.15(-0.20,-0.11)	158.61(102.07,204.38)	90.65(66.15,116.40)	1.749650684
Paraguay	3659.42(2952.16,4621.65)	4.03(3.88,4.18)	72.40(58.51,91.37)	0.82(0.71,0.94)	93.68(74.47,117.30)	54.71(43.96,73.78)	1.712172229

Peru	23398.40(18387.08,28835.81)	3.80(3.64,3.95)	74.05(58.10,91.41)	-0.15(-0.29,-0.01)	95.15(73.60,120.68)	54.85(43.28,67.71)	1.734800815
Philippines	48376.67(41349.61,56620.12)	3.37(3.19,3.54)	76.29(65.71,88.75)	-0.10(-0.28,0.07)	95.37(76.18,129.47)	62.29(45.40,74.97)	1.531086087
Poland	56504.72(49233.80,64514.31)	1.86(1.79,1.93)	76.28(66.39,87.17)	-0.25(-0.29,-0.21)	105.74(88.41,125.50)	58.62(48.70,69.54)	1.803984583
Portugal	20125.83(18230.07,22014.39)	3.13(2.98,3.29)	71.91(64.83,78.83)	0.61(0.51,0.72)	105.67(95.36,116.32)	48.60(43.02,54.66)	2.174211815
Puerto Rico	5452.13(4462.54,6573.13)	2.37(2.29,2.46)	68.28(55.81,82.36)	-0.26(-0.33,-0.19)	89.15(72.07,108.90)	52.42(42.51,62.90)	1.700691361
Qatar	488.45(359.78,643.45)	6.63(6.41,6.85)	148.63(96.03,194.58)	0.87(0.55,1.18)	147.11(101.64,195.01)	151.54(73.94,197.48)	0.970739326
Republic of Korea	61712.53(50254.67,68374.70)	5.70(5.56,5.85)	69.49(56.77,76.91)	0.82(0.72,0.92)	89.19(74.80,98.79)	57.14(42.68,64.89)	1.560832082
Republic of Moldova	2894.20(2559.52,3296.87)	-0.27(-0.44,-0.11)	49.92(44.05,56.84)	-1.78(-1.96,-1.60)	69.42(60.47,80.33)	38.50(33.45,44.20)	1.803271259
Romania	32385.67(27907.01,37814.15)	1.87(1.76,1.98)	78.79(67.74,92.34)	0.07(-0.01,0.14)	106.40(89.99,124.01)	61.20(52.25,71.14)	1.738443866
Russian Federation	156440.43(138096.32,175638.64)	1.15(0.98,1.33)	65.53(57.76,73.49)	-0.10(-0.27,0.06)	90.52(76.41,105.75)	54.18(46.52,62.23)	1.670718201
Rwanda	2991.99(2392.58,4060.59)	1.80(1.30,2.31)	66.81(53.37,92.41)	-0.82(-0.99,-0.65)	86.07(67.99,125.13)	55.33(42.04,82.97)	1.55538033
Saint Kitts and Nevis	57.14(45.02,67.42)	0.54(0.32,0.76)	110.02(87.86,129.11)	-0.03(-0.15,0.09)	144.07(107.20,173.56)	82.68(69.96,96.08)	1.742650514
Saint Lucia	193.63(168.94,221.79)	2.90(2.68,3.11)	93.90(82.08,107.42)	-0.44(-0.66,-0.22)	129.81(112.17,149.54)	66.06(56.80,75.60)	1.965068878

Saint Vincent and the Grenadines	110.96(99.18,124.15)	2.66(2.43,2.88)	86.81(77.66,96.96)	0.52(0.35,0.69)	107.55(95.64,121.07)	66.96(59.10,75.72)	1.606149252
Samoa	171.82(134.92,205.40)	1.23(1.19,1.27)	137.55(107.97,163.14)	-0.66(-0.78,-0.54)	149.62(122.29,170.40)	128.27(90.61,163.69)	1.166501688
San Marino	39.96(30.22,51.27)	2.63(2.51,2.76)	54.18(40.83,69.81)	0.03(-0.07,0.13)	72.99(54.23,91.87)	39.45(29.29,52.51)	1.850160599
Sao Tome and Principe	76.14(63.79,99.36)	2.25(2.17,2.34)	94.74(79.33,125.66)	1.01(0.96,1.06)	103.73(84.09,136.71)	86.68(66.86,122.02)	1.196687327
Saudi Arabia	11586.78(9409.13,13541.16)	2.84(2.72,2.97)	111.00(83.83,129.80)	-0.08(-0.20,0.05)	149.05(105.23,174.59)	59.39(44.12,73.70)	2.509816111
Senegal	5447.30(4490.09,6587.45)	3.71(3.52,3.89)	92.61(76.03,111.56)	0.61(0.47,0.75)	109.54(89.47,132.04)	77.66(61.33,95.82)	1.410604331
Serbia	13534.29(11134.85,15936.63)	1.98(1.74,2.23)	80.98(66.70,94.98)	-0.05(-0.19,0.09)	102.06(83.13,120.75)	65.26(51.92,76.71)	1.563903309
Seychelles	107.79(90.96,124.59)	1.75(1.65,1.86)	113.67(94.81,131.23)	-0.03(-0.11,0.06)	157.54(129.76,188.09)	81.39(63.71,96.10)	1.935536089
Sierra Leone	2284.87(1866.17,2782.83)	2.26(2.14,2.38)	81.65(67.36,99.18)	0.58(0.45,0.70)	88.31(71.10,111.39)	75.52(59.11,95.14)	1.169380615
Singapore	3934.12(3461.31,4370.25)	4.20(4.06,4.34)	55.04(48.34,60.99)	-0.54(-0.59,-0.48)	78.27(67.23,88.78)	38.00(33.05,42.69)	2.059700456
Slovakia	6054.10(4386.82,7352.64)	1.14(1.00,1.27)	64.50(46.67,78.35)	-0.42(-0.54,-0.30)	88.07(66.08,106.44)	51.08(32.92,62.60)	1.724127957
Slovenia	3202.93(2570.37,3894.45)	1.88(1.80,1.97)	66.22(52.95,80.26)	-0.70(-0.81,-0.59)	94.58(74.79,115.47)	47.48(38.34,58.00)	1.991844003
Solomon Islands	432.13(257.39,548.19)	2.64(2.48,2.80)	189.93(124.71,236.18)	-0.11(-0.18,-0.05)	218.55(126.01,281.66)	159.46(117.07,194.96)	1.370624696

Somalia	3084.68(2086.25,4639.34)	3.63(3.47,3.79)	64.58(43.97,96.80)	0.19(0.11,0.28)	89.40(62.61,133.26)	50.01(31.35,79.28)	1.787531067
South Africa	21900.55(19868.92,24026.13)	3.44(2.99,3.90)	58.59(52.97,64.03)	1.00(0.58,1.42)	78.45(70.78,86.52)	46.74(40.56,51.45)	1.678496823
South Sudan	1807.30(1399.74,2407.04)	0.93(0.82,1.04)	63.23(49.23,87.26)	-0.14(-0.17,-0.11)	78.29(59.44,111.36)	46.02(35.03,64.63)	1.701114317
Spain	89572.74(80184.10,98165.11)	2.60(2.40,2.79)	80.14(71.94,88.16)	0.16(0.07,0.24)	115.86(104.98,127.05)	54.64(47.48,61.39)	2.120389976
Sri Lanka	16519.86(11080.53,20876.45)	3.41(3.30,3.52)	72.12(48.33,90.79)	0.22(0.06,0.37)	88.49(62.79,112.00)	60.24(35.35,76.14)	1.468794755
Sudan	13237.55(10419.34,16768.35)	1.77(1.66,1.88)	85.50(67.76,107.47)	-0.17(-0.28,-0.06)	105.43(80.16,134.55)	61.34(46.61,78.25)	1.71889176
Suriname	430.35(368.61,495.87)	4.02(3.73,4.31)	78.29(67.00,89.84)	0.74(0.48,1.00)	101.67(85.58,119.02)	60.54(50.58,70.13)	1.679330758
Sweden	19305.21(17193.95,21102.89)	1.51(1.27,1.76)	80.02(71.39,88.26)	0.52(0.24,0.80)	108.49(97.92,119.15)	56.63(49.86,63.20)	1.915647858
Switzerland	14631.73(13010.25,16084.84)	1.86(1.77,1.94)	74.38(66.55,82.02)	0.03(-0.02,0.09)	104.94(93.85,116.17)	51.22(44.66,57.68)	2.048814029
Syrian Arab Republic	7787.06(5489.87,9816.12)	3.34(3.18,3.51)	83.92(58.40,104.91)	0.22(0.05,0.39)	99.11(71.33,124.80)	66.56(42.74,84.20)	1.489061437
Taiwan (Province of China)	35836.37(29719.68,43087.46)	4.13(4.08,4.19)	91.04(75.56,109.49)	0.26(0.15,0.37)	113.08(93.52,138.09)	73.05(59.94,87.81)	1.547941238
Tajikistan	3763.03(2500.19,4529.11)	2.94(2.68,3.20)	137.05(83.71,166.12)	2.65(2.43,2.87)	180.68(115.08,221.10)	104.79(50.99,127.18)	1.724179
Thailand	70239.85(51168.64,87843.43)	3.68(3.54,3.81)	71.97(51.97,89.87)	-0.80(-0.91,-0.68)	91.31(66.65,115.93)	56.93(39.10,70.40)	1.603974342

Timor-Leste	585.10(458.32,705.97)	5.62(5.44,5.80)	86.17(68.23,102.85)	0.80(0.64,0.96)	98.19(74.14,120.69)	74.37(56.99,91.79)	1.320374891
Togo	2002.62(1584.07,2443.98)	3.92(3.83,4.01)	81.67(64.68,98.43)	0.33(0.26,0.41)	99.64(80.07,124.28)	71.30(53.67,89.08)	1.397623387
Tokelau	1.70(1.23,2.07)	-1.62(-1.79,-1.46)	140.58(103.45,170.87)	-0.80(-0.86,-0.75)	140.54(105.62,165.04)	141.23(96.27,178.74)	0.995107884
Tonga	89.08(70.31,105.38)	1.48(1.26,1.71)	117.24(92.58,138.75)	-0.19(-0.35,-0.02)	155.86(119.19,182.79)	88.35(67.32,107.84)	1.764136956
Trinidad and Tobago	1260.23(1020.84,1547.60)	2.59(2.48,2.70)	71.58(58.04,87.63)	-0.13(-0.24,-0.02)	92.24(73.83,113.21)	54.67(44.02,67.37)	1.687213459
Tunisia	8271.24(6417.55,10676.45)	3.86(3.70,4.02)	72.68(56.20,93.69)	0.38(0.28,0.48)	94.12(72.53,125.72)	54.18(41.43,69.80)	1.737240821
Turkey	62178.07(48653.61,105235.91)	3.58(3.35,3.81)	76.05(59.45,129.67)	-0.01(-0.25,0.22)	92.94(73.07,147.74)	62.57(48.66,125.99)	1.485386284
Turkmenistan	2075.20(1708.96,2505.48)	2.87(2.68,3.06)	67.79(56.21,81.43)	0.42(0.23,0.61)	97.61(78.56,119.07)	48.43(39.84,57.61)	2.015408191
Tuvalu	14.65(9.89,18.62)	0.64(0.51,0.77)	166.42(114.06,209.95)	-0.70(-0.77,-0.62)	193.56(131.61,245.30)	144.33(94.42,184.17)	1.341090267
Uganda	7717.68(6025.14,10536.77)	2.71(2.64,2.78)	70.96(55.40,97.96)	0.24(0.11,0.36)	98.93(77.10,133.13)	52.59(38.92,78.05)	1.881075644
Ukraine	62116.78(54303.50,69986.80)	0.65(0.49,0.81)	77.75(67.90,87.61)	0.08(-0.08,0.23)	116.46(98.46,134.79)	58.31(48.96,68.02)	1.997350553
United Arab Emirates	2895.75(2100.43,3865.64)	6.98(6.68,7.27)	130.45(86.97,167.17)	-0.36(-0.90,0.18)	148.81(103.91,190.18)	91.36(48.72,126.55)	1.628711816
United Kingdom	122157.34(111144.37,131531.78)	1.30(1.20,1.41)	87.24(79.33,94.28)	0.14(0.06,0.23)	120.66(110.44,129.77)	60.86(54.78,66.64)	1.982509706

United Republic of Tanzania	13592.16(11191.75,19009.86)	3.15(3.08,3.22)	68.41(56.96,97.07)	0.32(0.26,0.38)	83.44(66.52,122.08)	56.49(45.73,81.21)	1.476907108
United States of America	492367.90(452936.14,521652.63)	2.80(2.68,2.91)	83.57(77.11,88.44)	1.00(0.86,1.14)	121.74(112.34,128.88)	54.54(49.41,58.49)	2.232020249
Uruguay	5212.26(4769.80,5623.82)	1.35(1.29,1.40)	86.05(79.31,92.89)	-0.04(-0.10,0.02)	128.70(117.99,139.90)	59.03(53.47,64.41)	2.180298946
Uzbekistan	11548.82(10040.07,13126.61)	2.62(2.36,2.88)	100.87(89.40,112.20)	2.41(2.03,2.80)	134.24(116.38,152.66)	79.31(70.12,89.15)	1.692678918
Vanuatu	247.98(140.67,324.10)	3.31(3.00,3.62)	173.10(101.29,223.29)	-0.40(-0.53,-0.26)	212.81(116.67,279.69)	129.08(78.49,166.96)	1.64864253
Venezuela (Bolivarian Republic of)	19275.97(15614.85,23743.50)	4.39(4.24,4.53)	72.95(59.22,89.68)	0.36(0.21,0.50)	96.78(77.70,120.12)	54.03(43.95,67.41)	1.791102504
Viet nam	75180.20(63120.46,86279.67)	3.53(3.42,3.63)	96.32(81.20,110.06)	0.64(0.58,0.71)	137.79(118.38,159.78)	70.92(56.71,85.87)	1.942914811
United States Virgin Islands	240.36(212.76,265.44)	3.98(3.77,4.19)	129.39(115.02,142.70)	0.62(0.42,0.82)	196.64(172.16,220.91)	80.98(70.25,91.18)	2.428101102
Yemen	8162.57(6382.68,10686.65)	4.24(4.15,4.32)	76.78(60.83,98.69)	0.69(0.60,0.77)	93.38(73.51,121.81)	61.33(45.77,80.33)	1.522475857
Zambia	3908.97(3177.45,4891.88)	2.65(2.58,2.72)	76.84(63.28,98.74)	-0.20(-0.33,-0.07)	102.57(84.58,128.14)	56.06(43.47,76.76)	1.829765921
Zimbabwe	4148.21(3457.84,4863.01)	1.78(1.50,2.06)	82.72(68.85,96.93)	0.40(0.27,0.52)	115.41(77.54,134.06)	62.25(48.43,78.71)	1.854129702

CI - confidence interval, EAPC - estimated annual percentage change, SDI - socio-demographic index, UI - uncertainty interval.

Table S10. The age-standardized DALY rate of Parkinson's disease in 1990 and 2019 and its temporal trends, by countries

	location	Absolute numbers 2019(95% UI)	EAPC(95% CI) (1990-2019)	Age-standardized rate 2019(95% UI)	EAPC(95% CI) (1990-2019)	males, 2019 (95% UI)	females, 2019(95% UI)	male-to-female ratio
Alzheimer's disease and other dementias	High SDI	2225885.41(1933223.40,2505521.55)	2.69(2.64,2.75)	100.57(87.24,113.37)	0.11(0.07,0.14)	86.25(73.75,97.90)	110.72(96.81,124.42)	0.779054037
Alzheimer's disease and other dementias	High-middle SDI	2013911.39(1708382.41,2308750.58)	3.08(3.01,3.16)	101.68(86.58,116.12)	0.13(0.11,0.16)	90.41(75.94,103.81)	109.70(93.80,125.33)	0.824131699
Alzheimer's disease and other dementias	Low SDI	273426.88(236801.29,312308.66)	3.03(2.91,3.15)	76.97(66.01,87.90)	-0.07(-0.10,-0.05)	70.22(59.78,80.44)	82.92(71.46,94.62)	0.846786197
Alzheimer's disease and other dementias	Low-middle SDI	821292.98(704426.89,943578.30)	3.59(3.48,3.71)	77.07(65.70,88.14)	-0.03(-0.06,-0.01)	71.76(60.89,82.56)	81.49(70.01,93.02)	0.880511647
Alzheimer's disease and other dementias	Middle SDI	1898447.49(1618748.57,2181889.58)	3.88(3.81,3.95)	93.29(79.52,106.76)	0.11(0.07,0.15)	83.65(70.60,96.16)	101.29(86.44,115.83)	0.825889861
Anxiety disorders	High SDI	6772133.33(5448770.98,8275524.72)	0.59(0.46,0.72)	710.54(570.38,872.80)	0.06(-0.06,0.18)	591.16(480.32,721.98)	837.43(663.79,1028.46)	0.705918685
Anxiety disorders	High-middle SDI	8282280.49(6700669.33,9995883.43)	0.66(0.61,0.71)	584.85(476.96,704.58)	0.02(0.00,0.04)	469.78(383.04,566.29)	707.59(572.42,853.58)	0.663921102
Anxiety disorders	Low SDI	6180445.73(4855895.19,7768653.73)	2.95(2.90,3.00)	556.08(444.56,686.33)	0.12(0.07,0.16)	491.63(396.43,605.63)	620.63(493.13,771.88)	0.79215087
Anxiety disorders	Low-middle SDI	9900883.93(7961557.19,12184387.93)	1.90(1.86,1.95)	549.87(446.34,670.04)	0.15(0.10,0.21)	468.08(381.14,566.52)	633.42(507.96,773.60)	0.738980979

Anxiety disorders	Middle SDI	14655612.56(11918843.22,17665671.95)	1.18(1.10,1.25)	599.21(487.91,719.35)	0.05(0.02,0.08)	496.21(407.62,594.85)	706.31(569.48,851.60)	0.702539733
Bipolar disorder	High SDI	528903.24(454945.99,611484.98)	0.68(0.65,0.71)	55.85(48.12,63.76)	0.10(0.10,0.11)	56.07(48.37,63.99)	55.64(47.93,63.89)	1.007859038
Bipolar disorder	High-middle SDI	571566.42(477052.04,677223.30)	0.63(0.56,0.69)	40.94(34.00,48.50)	0.05(0.00,0.10)	39.48(32.73,47.00)	42.54(35.42,50.53)	0.928006162
Bipolar disorder	Low SDI	561632.78(443847.58,699152.80)	2.90(2.89,2.91)	50.39(41.20,61.23)	0.03(0.03,0.04)	50.01(40.95,60.66)	50.78(41.57,61.57)	0.984793828
Bipolar disorder	Low-middle SDI	762706.08(630158.82,921413.83)	1.91(1.87,1.94)	41.65(34.54,49.97)	0.11(0.11,0.12)	41.91(34.81,50.28)	41.40(34.35,49.59)	1.012378794
Bipolar disorder	Middle SDI	961490.36(802505.69,1148536.36)	1.40(1.30,1.50)	39.45(32.93,47.03)	0.29(0.28,0.30)	38.72(32.35,46.28)	40.30(33.52,48.10)	0.960745863
Depressive disorders	High SDI	44711792.44(39796761.07,50166003.31)	1.10(0.97,1.23)	4013.63(3545.48,4550.43)	0.31(0.18,0.44)	2954.26(2605.43,3347.04)	5106.74(4498.75,5791.36)	0.578501567
Depressive disorders	High-middle SDI	53642568.73(47529705.87,60307944.74)	0.80(0.72,0.89)	3184.21(2809.60,3583.66)	-0.50(-0.57,-0.43)	2325.53(2047.27,2610.99)	4033.88(3554.36,4548.68)	0.576499314
Depressive disorders	Low SDI	40743981.19(34959157.28,47317677.84)	2.45(2.33,2.56)	4770.22(4142.24,5461.66)	-0.38(-0.51,-0.25)	3895.34(3386.73,4462.33)	5630.75(4897.55,6459.02)	0.691798396
Depressive disorders	Low-middle SDI	70155480.27(61292236.79,79973479.99)	1.50(1.30,1.70)	4180.30(3660.97,4740.48)	-0.62(-0.80,-0.44)	3316.34(2909.06,3752.51)	5029.39(4401.49,5718.63)	0.659392558
Depressive disorders	Middle SDI	80760068.75(71066731.89,91500542.35)	1.56(1.48,1.64)	3139.00(2765.35,3540.43)	-0.20(-0.28,-0.13)	2388.79(2103.08,2690.80)	3881.54(3415.00,4384.30)	0.615423846
Headache disorders	High SDI	122697543.67(110214102.11,135398104.60)	0.66(0.65,0.67)	11872.41(10625.09,13135.81)	-0.04(-0.05,-0.04)	10998.68(9827.65,12190.97)	12781.02(11460.13,14103.64)	0.860547381
Headache disorders	High-middle SDI	147632356.96(133120098.51,163057274.20)	0.74(0.70,0.79)	10028.50(9003.95,11069.53)	-0.03(-0.05,-0.01)	9354.20(8341.79,10358.93)	10720.53(9651.30,11839.93)	0.872549629

Headache disorders	Low SDI	102824494.66(90100264.05,115281239.94)	2.77(2.75,2.78)	9624.94(8608.94,10666.35)	-0.06(-0.07,-0.05)	9452.69(8453.43,10489.24)	9799.10(8774.61,10847.70)	0.964649468
Headache disorders	Low-middle SDI	183271605.77(163036933.89,203398875.72)	1.75(1.72,1.78)	10249.57(9208.66,11341.02)	0.00(-0.02,0.01)	9955.96(8917.88,11048.25)	10548.77(9486.86,11636.90)	0.94380247
Headache disorders	Middle SDI	236970759.13(213250057.54,261970454.49)	1.38(1.31,1.45)	9680.04(8698.60,10705.57)	0.16(0.14,0.18)	9104.06(8149.67,10089.22)	10266.78(9266.05,11332.94)	0.886749345
Idiopathic epilepsy	High SDI	405429.20(271412.36,546314.86)	1.02(0.96,1.07)	44.29(29.01,60.92)	0.37(0.31,0.42)	47.95(31.37,65.93)	40.51(26.45,55.84)	1.1837732
Idiopathic epilepsy	High-middle SDI	461438.12(315282.44,611631.99)	0.82(0.77,0.87)	36.42(24.70,50.24)	0.46(0.41,0.51)	39.44(26.80,53.98)	33.34(22.52,46.31)	1.183166493
Idiopathic epilepsy	Low SDI	508821.64(321874.10,734212.23)	2.73(2.69,2.77)	40.00(26.46,55.65)	0.20(0.15,0.25)	43.68(28.74,60.94)	36.41(23.61,50.96)	1.199667969
Idiopathic epilepsy	Low-middle SDI	665438.30(445197.58,899863.77)	1.77(1.71,1.83)	37.46(25.47,50.67)	0.61(0.54,0.68)	40.37(27.56,54.33)	34.52(23.34,46.61)	1.169422565
Idiopathic epilepsy	Middle SDI	855316.43(605342.33,1142873.05)	1.29(1.22,1.37)	38.21(27.05,50.80)	0.63(0.58,0.69)	40.64(28.61,53.87)	35.73(25.17,47.95)	1.137585236
Motor neuron disease	High SDI	26517.57(25122.57,27876.96)	2.31(2.27,2.35)	1.62(1.53,1.71)	0.42(0.40,0.44)	1.92(1.82,2.01)	1.35(1.26,1.43)	1.42204906
Motor neuron disease	High-middle SDI	12631.76(11206.72,14326.54)	1.88(1.78,1.98)	0.72(0.64,0.81)	0.23(0.17,0.28)	0.83(0.74,0.93)	0.63(0.55,0.72)	1.314959505
Motor neuron disease	Low SDI	4586.24(3795.79,5560.73)	2.57(2.43,2.70)	0.55(0.46,0.68)	-0.06(-0.22,0.09)	0.57(0.48,0.70)	0.53(0.44,0.65)	1.076404685
Motor neuron disease	Low-middle SDI	7510.85(6256.76,9149.79)	1.77(1.61,1.94)	0.47(0.39,0.57)	-0.08(-0.25,0.09)	0.50(0.42,0.60)	0.45(0.37,0.54)	1.120474252
Motor neuron disease	Middle SDI	12418.90(10327.01,15098.83)	1.72(1.52,1.93)	0.51(0.43,0.60)	-0.16(-0.35,0.03)	0.54(0.46,0.65)	0.47(0.39,0.56)	1.163311974

Multiple sclerosis	High SDI	24240.31(21899.93,26506.65)	0.71(0.69,0.74)	2.53(2.29,2.76)	0.40(0.37,0.43)	1.64(1.48,1.80)	3.48(3.16,3.78)	0.472707247
Multiple sclerosis	High-middle SDI	12329.85(10846.44,13773.06)	0.62(0.55,0.69)	0.81(0.71,0.90)	-0.27(-0.32,-0.22)	0.66(0.57,0.74)	0.97(0.86,1.08)	0.677191444
Multiple sclerosis	Low SDI	3951.44(3191.75,4744.86)	3.26(3.19,3.32)	0.40(0.33,0.48)	0.32(0.26,0.37)	0.30(0.24,0.36)	0.51(0.42,0.60)	0.595840361
Multiple sclerosis	Low-middle SDI	7448.42(6087.78,8850.19)	2.52(2.51,2.53)	0.42(0.34,0.49)	0.43(0.39,0.47)	0.32(0.26,0.38)	0.52(0.43,0.61)	0.61888677
Multiple sclerosis	Middle SDI	11344.03(9469.32,13171.00)	2.41(2.32,2.50)	0.44(0.37,0.51)	0.76(0.71,0.81)	0.35(0.29,0.41)	0.53(0.44,0.62)	0.655730001
Neurological disorders	High SDI	125710225.61(113086127.06,138520583.10)	0.69(0.68,0.70)	12038.16(10787.32,13302.51)	-0.04(-0.05,-0.03)	11161.24(9985.13,12350.83)	12947.30(11619.74,14257.14)	0.862051977
Neurological disorders	High-middle SDI	150404983.33(135986899.70,165797786.16)	0.77(0.72,0.81)	10181.48(9137.97,11235.70)	-0.03(-0.05,-0.01)	9502.96(8497.35,10507.13)	10875.55(9799.02,11989.31)	0.873790706
Neurological disorders	Low SDI	103656325.93(90948406.12,116223555.62)	2.77(2.75,2.78)	9752.19(8734.34,10800.42)	-0.05(-0.07,-0.04)	9578.37(8571.88,10613.10)	9927.35(8910.28,10978.99)	0.964846069
Neurological disorders	Low-middle SDI	184908427.10(164658528.51,205100570.89)	1.76(1.73,1.79)	10375.78(9329.04,11472.75)	0.00(-0.02,0.01)	10081.87(9042.67,11180.97)	10674.64(9613.37,11774.40)	0.944469287
Neurological disorders	Middle SDI	240050404.79(216622367.96,265084540.40)	1.40(1.33,1.46)	9825.35(8834.15,10848.02)	0.16(0.14,0.18)	9245.03(8294.69,10238.81)	10415.18(9412.70,11481.50)	0.887649481
Parkinson's disease	High SDI	330609.45(297948.77,362338.32)	3.56(3.35,3.77)	16.75(15.11,18.31)	1.39(1.21,1.56)	24.80(22.18,27.36)	10.23(9.31,11.15)	2.425642503
Parkinson's disease	High-middle SDI	272315.25(236518.28,309170.41)	2.63(2.59,2.68)	13.36(11.63,15.11)	0.25(0.20,0.30)	17.43(15.27,19.65)	10.39(9.05,11.80)	1.677948052
Parkinson's disease	Low SDI	41045.07(36256.99,46043.80)	3.08(2.99,3.18)	9.32(8.27,10.38)	0.20(0.17,0.23)	10.90(9.65,12.16)	7.89(6.97,8.81)	1.38252578

Parkinson's disease	Low-middle SDI	135130.78(117777.61, 153296.49)	3.55(3.51,3.59)	10.78(9.39,12.18)	0.32(0.29,0.34)	12.97(11.31,14.69)	8.90(7.73,10.03)	1.458300704
Parkinson's disease	Middle SDI	302118.81(258633.58, 345463.57)	3.77(3.69,3.86)	12.86(11.08,14.68)	0.40(0.31,0.48)	15.78(13.60,18.07)	10.38(8.94,11.90)	1.519579305
Schizophrenia	High SDI	178152.98(150468.15, 209862.20)	0.39(0.33,0.45)	19.14(16.08,22.75)	0.06(0.01,0.11)	19.97(16.83,23.78)	18.24(15.32,21.59)	1.094801841
Schizophrenia	High-middle SDI	252822.10(217855.01, 291671.28)	0.68(0.56,0.79)	17.64(15.22,20.57)	0.06(0.05,0.07)	18.71(16.18,21.74)	16.51(14.25,19.28)	1.133367777
Schizophrenia	Low SDI	158320.96(131223.94, 192075.95)	2.87(2.86,2.88)	14.36(11.96,17.33)	-0.04(-0.04,-0.03)	15.60(13.00,18.79)	13.14(10.89,15.87)	1.187117916
Schizophrenia	Low-middle SDI	288288.67(241334.04, 347095.56)	1.76(1.73,1.79)	15.18(12.70,18.24)	-0.06(-0.07,-0.05)	16.74(14.04,20.07)	13.61(11.37,16.36)	1.229801472
Schizophrenia	Middle SDI	411500.26(346741.02, 489083.12)	0.94(0.85,1.03)	16.39(13.90,19.53)	-0.12(-0.13,-0.12)	17.49(14.83,20.79)	15.28(12.93,18.31)	1.144808934
Stroke	High SDI	1678574.99(1521049.95,1865222.61)	0.51(0.46,0.56)	96.47(87.65,106.10)	-1.36(-1.41,-1.31)	91.95(83.71,101.11)	99.64(90.11,110.09)	0.922824422
Stroke	High-middle SDI	3165293.91(2824984.36,3590799.11)	1.10(1.01,1.18)	160.20(143.77,180.69)	-1.16(-1.24,-1.09)	164.59(147.80,185.32)	155.38(138.69,175.30)	1.05926696
Stroke	Low SDI	879763.24(807233.65, 960847.44)	2.12(2.02,2.22)	145.63(134.46,158.83)	-0.73(-0.78,-0.67)	140.82(129.63,154.18)	150.05(138.55,162.95)	0.93849767
Stroke	Low-middle SDI	2079896.30(1901358.19,2300752.31)	2.33(2.25,2.40)	148.92(136.20,164.08)	-0.45(-0.49,-0.40)	148.62(135.87,163.56)	149.05(135.95,164.41)	0.997099836
Stroke	Middle SDI	4414557.89(3948139.94,4990709.83)	2.43(2.38,2.49)	180.42(161.94,202.86)	-0.57(-0.64,-0.51)	184.23(166.16,205.81)	176.56(157.46,199.09)	1.043408219

CI - confidence interval, EAPC - estimated annual percentage change, SDI - socio-demographic index, UI - uncertainty interval.

Table S11. The age-standardized incidence rate of other neurological disorders in 1990 and 2019 and its temporal trends，by social development index (SDI)

		Absolute numbers		Age-standardized rate				
	**location**	**2019(95% UI)**	**EAPC(95% CI) (1990-2019)**	**2019(95% UI)**	**EAPC(95% CI) (1990-2019)**	**males, 2019 (95% UI)**	**females, 2019(95% UI)**	**male-to-female ratio**
Alzheimer's disease and other dementias	High SDI	7655559.77(3563493.34,15743216.28)	2.90(2.85,2.95)	332.40(155.00, 688.29)	0.11(0.08,0.13)	296.00(133.28, 639.96)	354.48(168.47,715.52)	0.835012311
Alzheimer's disease and other dementias	High-middle SDI	6807504.74(3082423.14,14821541.11)	3.31(3.24,3.37)	348.46(157.71, 754.37)	0.18(0.13,0.22)	315.49(138.28, 714.02)	368.57(169.70,783.78)	0.855984755
Alzheimer's disease and other dementias	Low SDI	1039872.30(429369.10,2448167.71)	3.46(3.37,3.56)	315.76(129.02, 742.90)	0.28(0.25,0.30)	290.29(116.14, 706.37)	336.75(138.54,778.51)	0.862029955
Alzheimer's disease and other dementias	Low-middle SDI	3072172.00(1276080.64,7056376.96)	3.92(3.82,4.03)	304.76(126.52, 702.30)	0.20(0.18,0.23)	286.42(117.48, 677.44)	319.41(133.53,723.21)	0.896711419
Alzheimer's disease and other dementias	Middle SDI	6689285.14(2842927.66,15089735.49)	3.94(3.90,3.99)	346.11(148.62, 770.18)	0.14(0.12,0.16)	311.84(131.81, 705.18)	371.54(161.98,807.43)	0.839334061
Anxiety disorders	High SDI	4908788.65(3385800.90,6714919.69)	0.72(0.58,0.87)	456.89(312.75, 626.95)	-0.02(-0.15,0.11)	334.80(230.68, 464.06)	582.82(397.26,802.04)	0.574452782
Anxiety disorders	High-middle SDI	5515235.33(3836279.46,7507612.81)	0.96(0.89,1.03)	359.98(250.24, 494.80)	0.03(0.00,0.05)	261.96(182.38, 360.88)	459.48(317.88,626.53)	0.570126538
Anxiety disorders	Low SDI	3357704.69(2253431.53,4691816.28)	3.04(2.98,3.09)	331.48(226.98, 455.65)	0.16(0.12,0.21)	275.84(188.33, 378.70)	386.37(265.46,534.17)	0.71393671
Anxiety disorders	Low-middle SDI	5778110.10(3987041.38,7977347.90)	2.18(2.12,2.23)	328.89(228.88, 451.62)	0.22(0.17,0.27)	259.15(180.45, 355.80)	398.15(275.26,547.42)	0.650881475
Anxiety disorders	Middle SDI	9097499.44(6322382.77,12421170.24)	1.46(1.38,1.54)	363.15(252.87, 497.93)	0.05(0.01,0.08)	279.64(195.85, 384.35)	447.18(310.45,611.33)	0.62533187
Bipolar disorder	High SDI	1671827.76(1048753.10,2489338.71)	0.81(0.79,0.83)	150.16(93.76,226.84)	-0.01(-0.02,-0.01)	140.50(86.85,213.21)	160.17(100.29,239.06)	0.877188533

Bipolar disorder	High-middle SDI	1665024.85(1025298.34,2527930.02)	1.05(0.99,1.11)	103.42(62.69,158.37)	-0.06(-0.09,-0.04)	95.48(57.78,147.69)	111.59(67.68,170.89)	0.855596645
Bipolar disorder	Low SDI	1058414.51(638268.32,1647607.61)	2.97(2.95,2.99)	110.90(66.80,170.12)	0.07(0.07,0.08)	111.82(67.22,172.38)	110.00(66.26,168.78)	1.01653233
Bipolar disorder	Low-middle SDI	1670659.49(1009161.90,2561519.00)	2.24(2.22,2.26)	94.62(57.46,144.43)	0.16(0.15,0.16)	95.20(57.74,145.36)	94.05(57.24,143.71)	1.012200612
Bipolar disorder	Middle SDI	2429996.06(1481939.05,3756744.23)	1.90(1.82,1.98)	94.20(57.10,145.54)	0.29(0.29,0.29)	90.61(54.87,139.70)	97.94(59.44,150.82)	0.925220262
Depressive disorders	High SDI	7025129.01(4955200.18,9506635.83)	1.03(0.93,1.13)	626.84(438.47,852.48)	0.23(0.14,0.33)	474.22(329.94,648.61)	784.78(549.02,1068.38)	0.604267501
Depressive disorders	High-middle SDI	8896916.59(6247985.89,12123141.64)	0.93(0.85,1.01)	523.01(367.02,713.05)	-0.40(-0.46,-0.34)	393.02(273.92,539.52)	652.04(458.64,892.14)	0.602763043
Depressive disorders	Low SDI	6345788.60(4316622.75,8788144.84)	2.54(2.45,2.64)	738.87(514.68,1011.24)	-0.30(-0.41,-0.19)	609.81(425.57,837.38)	865.78(603.00,1184.81)	0.704354692
Depressive disorders	Low-middle SDI	11026538.37(7715897.51,15191252.58)	1.63(1.46,1.80)	654.34(458.32,897.85)	-0.51(-0.66,-0.35)	527.91(370.03,725.17)	778.68(545.17,1063.16)	0.677962222
Depressive disorders	Middle SDI	13541947.42(9515935.03,18454506.60)	1.65(1.58,1.72)	521.68(366.80,709.93)	-0.18(-0.24,-0.12)	405.87(283.84,557.57)	636.34(446.63,868.13)	0.637818948
Headache disorders	High SDI	6862292.80(1587513.84,14597883.00)	0.75(0.72,0.77)	636.85(134.31,1375.80)	0.01(-0.02,0.04)	438.67(104.37,916.10)	843.81(162.63,1835.84)	0.519872208
Headache disorders	High-middle SDI	9324533.28(2334293.39,19464833.36)	1.11(1.03,1.18)	588.30(135.50,1253.88)	0.06(0.05,0.08)	434.76(109.97,912.04)	745.68(157.93,1602.40)	0.583040795
Headache disorders	Low SDI	5256507.70(977975.26,11447931.02)	2.87(2.85,2.90)	527.38(112.47,1121.59)	0.00(-0.01,0.01)	418.75(90.89,892.26)	634.79(132.58,1353.50)	0.659663368
Headache disorders	Low-middle SDI	10273363.12(1823619.23,22369461.67)	1.97(1.95,2.00)	574.85(106.87,1248.43)	0.02(0.01,0.04)	449.30(88.45,974.04)	700.09(124.77,1551.93)	0.641772126

Headache disorders	Middle SDI	14875826.75(2936371.64,32612740.43)	1.69(1.61,1.77)	581.76(111.38,1284.82)	0.19(0.16,0.21)	445.44(97.95,946.79)	719.17(124.73,1608.06)	0.619382783
Idiopathic epilepsy	High SDI	1281948.55(883071.20,1887128.17)	0.80(0.71,0.88)	120.16(81.78,176.38)	-0.11(-0.19,-0.03)	130.36(90.33,190.27)	109.81(72.23,164.11)	1.187161449
Idiopathic epilepsy	High-middle SDI	1760650.59(1267274.64,2360202.12)	-0.49(-0.58,-0.40)	124.84(89.53,168.73)	-1.21(-1.30,-1.12)	139.31(101.70,185.24)	110.36(77.22,152.17)	1.262336671
Idiopathic epilepsy	Low SDI	2804185.69(2102968.24,3691130.36)	1.57(1.53,1.61)	246.92(188.89,321.76)	-0.87(-0.91,-0.83)	281.84(217.88,361.25)	212.85(156.02,283.03)	1.324169886
Idiopathic epilepsy	Low-middle SDI	3744714.70(2910586.35,4799430.05)	0.25(0.19,0.31)	213.08(166.07,271.60)	-1.08(-1.14,-1.02)	225.67(176.00,286.31)	200.56(153.75,256.88)	1.125216578
Idiopathic epilepsy	Middle SDI	3477914.00(2556903.29,4508754.39)	-0.02(-0.09,0.06)	147.56(108.63,191.96)	-0.96(-1.02,-0.91)	161.85(120.96,210.00)	133.22(96.20,176.49)	1.214880137
Motor neuron disease	High SDI	547119.40(518174.45,570407.43)	2.22(2.15,2.29)	34.94(33.35,36.39)	0.33(0.26,0.40)	41.11(38.98,42.69)	29.16(27.53,30.66)	1.40984067
Motor neuron disease	High-middle SDI	249506.73(230679.90,267477.67)	1.17(1.00,1.34)	14.20(13.10,15.32)	-0.66(-0.84,-0.48)	17.08(15.58,18.63)	11.47(10.42,12.52)	1.488793588
Motor neuron disease	Low SDI	20803.41(17068.62,25153.78)	2.89(2.69,3.09)	2.54(2.02,3.23)	0.50(0.33,0.67)	2.67(1.89,3.72)	2.43(2.03,2.99)	1.101793125
Motor neuron disease	Low-middle SDI	72956.17(63758.51,83082.03)	2.21(1.91,2.51)	4.63(4.04,5.28)	0.64(0.43,0.85)	5.21(4.39,6.16)	4.08(3.55,4.81)	1.278552989
Motor neuron disease	Middle SDI	143598.29(129350.26,157230.34)	0.50(-0.04,1.03)	5.60(5.05,6.11)	-1.12(-1.54,-0.69)	6.59(5.86,7.32)	4.62(4.10,5.08)	1.424930489
Multiple sclerosis	High SDI	500325.08(411436.08,581040.50)	1.52(1.45,1.59)	35.38(29.05,41.50)	0.10(0.05,0.16)	24.47(18.99,29.51)	46.28(36.86,55.31)	0.528798156
Multiple sclerosis	High-middle SDI	260398.48(207342.42,359882.44)	0.37(0.26,0.47)	14.03(11.15,19.37)	-1.33(-1.43,-1.23)	11.58(9.08,17.56)	16.46(12.87,23.37)	0.703774539

Multiple sclerosis	Low SDI	60580.17(47505.28,77711.86)	3.24(3.19,3.30)	8.09(6.35,10.20)	0.32(0.31,0.34)	6.27(4.76,8.08)	9.89(7.61,14.23)	0.633436796
Multiple sclerosis	Low-middle SDI	138239.16(116847.85,167903.68)	2.88(2.83,2.92)	8.52(7.24,10.30)	0.40(0.35,0.44)	6.97(5.74,8.50)	10.05(8.08,12.95)	0.693791297
Multiple sclerosis	Middle SDI	199666.15(168751.17,241140.76)	2.52(2.45,2.58)	7.48(6.33,9.08)	0.21(0.18,0.24)	6.59(5.21,8.79)	8.38(6.91,10.60)	0.78614664
Neurological disorders	High SDI	19237221.62(11714530.22,30377786.65)	1.72(1.68,1.76)	1303.51(736.19,2111.81)	0.07(0.05,0.09)	1113.90(699.22,1736.58)	1495.57(773.61,2526.92)	0.74480178
Neurological disorders	High-middle SDI	20676710.10(11919901.38,33556099.45)	1.55(1.54,1.57)	1218.74(691.84,1981.10)	-0.11(-0.13,-0.09)	1082.73(670.43,1686.23)	1354.95(706.71,2268.98)	0.799095634
Neurological disorders	Low SDI	10231802.11(5619983.15,17107547.80)	2.51(2.48,2.54)	1236.62(720.11,1946.39)	-0.10(-0.11,-0.09)	1156.37(717.53,1780.50)	1313.78(716.69,2180.33)	0.880187407
Neurological disorders	Low-middle SDI	19243323.01(10170116.96,32046998.27)	1.85(1.82,1.87)	1243.81(695.95,2023.73)	-0.12(-0.14,-0.10)	1134.81(681.23,1774.12)	1352.13(711.61,2298.17)	0.839276598
Neurological disorders	Middle SDI	28280338.62(15309773.36,46788483.78)	1.90(1.88,1.92)	1217.94(673.68,2029.28)	0.01(0.00,0.02)	1089.16(653.16,1719.00)	1343.31(686.21,2312.50)	0.810800693
Parkinson's disease	High SDI	1604208.01(1454059.98,1725513.24)	2.78(2.72,2.84)	76.55(69.65,82.39)	0.48(0.40,0.55)	107.65(98.79,115.39)	53.14(47.34,57.90)	2.025928922
Parkinson's disease	High-middle SDI	1553399.66(1403032.70,1701395.46)	2.35(2.29,2.42)	76.75(69.40,84.08)	-0.27(-0.35,-0.19)	105.70(95.06,116.35)	57.08(50.34,63.45)	1.851565538
Parkinson's disease	Low SDI	318441.47(282135.67,364300.98)	2.90(2.82,2.97)	78.37(69.22,90.27)	0.07(0.02,0.11)	95.49(82.17,113.05)	63.14(54.24,74.35)	1.512297772

Parkinson's disease	Low-middle SDI	1012325.78(908057.34,1129431.48)	3.39(3.32,3.45)	85.44(76.67,95.24)	0.04(-0.03,0.12)	106.19(93.11,120.07)	68.07(58.41,79.56)	1.559863126
Parkinson's disease	Middle SDI	1800862.09(1629326.97,1993459.08)	3.47(3.41,3.52)	82.22(74.03,90.57)	0.02(-0.05,0.09)	105.42(93.40,118.22)	63.51(55.51,70.43)	1.65987205
Schizophrenia	High SDI	2541568.15(1865247.53,3207291.30)	0.96(0.90,1.02)	213.36(155.49,271.87)	0.04(0.00,0.09)	221.36(162.06,282.69)	204.62(148.87,259.42)	1.081768104
Schizophrenia	High-middle SDI	3367871.62(2476407.48,4227909.76)	1.49(1.44,1.53)	194.45(142.55,244.50)	0.13(0.11,0.15)	204.48(150.65,256.47)	184.13(135.51,231.70)	1.11048893
Schizophrenia	Low SDI	1304857.92(936059.49,1691711.88)	2.93(2.89,2.97)	150.61(108.07,193.89)	0.04(0.02,0.05)	163.81(117.86,211.65)	137.63(98.54,176.22)	1.190193177
Schizophrenia	Low-middle SDI	3006048.43(2167795.59,3849888.00)	2.26(2.25,2.28)	174.30(125.23,222.16)	0.04(0.03,0.05)	192.82(138.75,246.77)	155.97(112.23,199.22)	1.236278164
Schizophrenia	Middle SDI	4878967.45(3547734.68,6228126.98)	1.92(1.85,1.99)	182.38(133.36,232.51)	0.02(0.01,0.02)	192.92(140.92,247.00)	171.87(125.98,219.89)	1.122505325
Stroke	High SDI	12973818.17(11716037.91,14044417.40)	-0.68(-0.83,-0.54)	703.13(640.03,761.95)	-2.66(-2.80,-2.53)	775.13(717.92,831.32)	634.85(566.90,697.43)	1.220972823
Stroke	High-middle SDI	34933599.23(32080999.72,37584859.38)	-0.15(-0.38,0.08)	1750.21(1606.12,1882.09)	-2.41(-2.66,-2.16)	2079.62(1867.15,2304.51)	1462.13(1317.49,1605.46)	1.422327574
Stroke	Low SDI	12028395.10(10644696.58,13563094.15)	1.55(1.48,1.62)	2162.03(1912.56,2441.68)	-1.08(-1.12,-1.03)	2225.29(1934.12,2550.34)	2097.54(1838.03,2367.28)	1.060904672
Stroke	Low-middle SDI	30370492.21(27803534.42,33055330.08)	1.68(1.63,1.72)	2220.33(2036.51,2414.45)	-1.07(-1.16,-0.98)	2443.84(2208.55,2688.16)	2010.72(1795.22,2240.43)	1.215405487

Stroke	Middle SDI	52844428.47(48314527.43,57558906.03)	1.46(1.39,1.54)	2199.78(2017.08,2393.22)	-1.44(-1.55,-1.32)	2578.24(2304.27,2873.26)	1848.14(1652.39,2064.34)	1.395044082

CI - confidence interval, EAPC - estimated annual percentage change, SDI - socio-demographic index, UI - uncertainty interval.

Table S12. The age-standardized DALY rate of other neurological disorders in 1990 and 2019 and its temporal trends, by social development index (SDI)

	location	Absolute numbers 2019(95 % UI)	EAPC(95% CI) (1990-2019)	Age-standardized rate 2019(95% UI)	EAPC(95% CI) (1990-2019)	males, 2019 (95% UI)	females, 2019(95% UI)	male-to-female ratio
Alzheimer's disease and other dementias	High SDI	7655559.77(3563493.34 ,15743216.28)	2.90(2.85,2.95)	332.40(155.00,688.29)	0.11(0.08,0.13)	296.00(133.28,639.96)	354.48(168.47,715.52)	0.835012311
Alzheimer's disease and other dementias	High-middle SDI	6807504.74(3082423.14 ,14821541.11)	3.31(3.24,3.37)	348.46(157.71,754.37)	0.18(0.13,0.22)	315.49(138.28,714.02)	368.57(169.70,783.78)	0.855984755
Alzheimer's disease and other dementias	Low SDI	1039872.30(429369.10, 2448167.71)	3.46(3.37,3.56)	315.76(129.02,742.90)	0.28(0.25,0.30)	290.29(116.14,706.37)	336.75(138.54,778.51)	0.862029955
Alzheimer's disease and other dementias	Low-middle SDI	3072172.00(1276080.64 ,7056376.96)	3.92(3.82,4.03)	304.76(126.52,702.30)	0.20(0.18,0.23)	286.42(117.48,677.44)	319.41(133.53,723.21)	0.896711419
Alzheimer's disease and other dementias	Middle SDI	6689285.14(2842927.66 ,15089735.49)	3.94(3.90,3.99)	346.11(148.62,770.18)	0.14(0.12,0.16)	311.84(131.81,705.18)	371.54(161.98,807.43)	0.839334061
Anxiety disorders	High SDI	4908788.65(3385800.90 ,6714919.69)	0.72(0.58,0.87)	456.89(312.75,626.95)	-0.02(-0.15,0.11)	334.80(230.68,464.06)	582.82(397.26,802.04)	0.574452782
Anxiety disorders	High-middle SDI	5515235.33(3836279.46 ,7507612.81)	0.96(0.89,1.03)	359.98(250.24,494.80)	0.03(0.00,0.05)	261.96(182.38,360.88)	459.48(317.88,626.53)	0.570126538
Anxiety disorders	Low SDI	3357704.69(2253431.53 ,4691816.28)	3.04(2.98,3.09)	331.48(226.98,455.65)	0.16(0.12,0.21)	275.84(188.33,378.70)	386.37(265.46,534.17)	0.71393671
Anxiety disorders	Low-middle SDI	5778110.10(3987041.38 ,7977347.90)	2.18(2.12,2.23)	328.89(228.88,451.62)	0.22(0.17,0.27)	259.15(180.45,355.80)	398.15(275.26,547.42)	0.650881475
Anxiety disorders	Middle SDI	9097499.44(6322382.77 ,12421170.24)	1.46(1.38,1.54)	363.15(252.87,497.93)	0.05(0.01,0.08)	279.64(195.85,384.35)	447.18(310.45,611.33)	0.62533187
Bipolar disorder	High SDI	1671827.76(1048753.10	0.81(0.79,0.83)	150.16(93.76,226.84)	-0.01(-0.02,-0.01)	140.50(86.85,213.21)	160.17(100.29,239.06)	0.877188533

		,2489338.71)						
Bipolar disorder	High-middle SDI	1665024.85(1025298.34 ,2527930.02)	1.05(0.99,1.11)	103.42(62.69,158.37)	-0.06(-0.09,-0.04)	95.48(57.78,147.69)	111.59(67.68,170.89)	0.855596645
Bipolar disorder	Low SDI	1058414.51(638268.32, 1647607.61)	2.97(2.95,2.99)	110.90(66.80,170.12)	0.07(0.07,0.08)	111.82(67.22,172.38)	110.00(66.26,168.78)	1.01653233
Bipolar disorder	Low-middle SDI	1670659.49(1009161.90 ,2561519.00)	2.24(2.22,2.26)	94.62(57.46,144.43)	0.16(0.15,0.16)	95.20(57.74,145.36)	94.05(57.24,143.71)	1.012200612
Bipolar disorder	Middle SDI	2429996.06(1481939.05 ,3756744.23)	1.90(1.82,1.98)	94.20(57.10,145.54)	0.29(0.29,0.29)	90.61(54.87,139.70)	97.94(59.44,150.82)	0.925220262
Depressive disorders	High SDI	7025129.01(4955200.18 ,9506635.83)	1.03(0.93,1.13)	626.84(438.47,852.48)	0.23(0.14,0.33)	474.22(329.94,648.61)	784.78(549.02,1068.38)	0.604267501
Depressive disorders	High-middle SDI	8896916.59(6247985.89 ,12123141.64)	0.93(0.85,1.01)	523.01(367.02,713.05)	-0.40(-0.46,-0.34)	393.02(273.92,539.52)	652.04(458.64,892.14)	0.602763043
Depressive disorders	Low SDI	6345788.60(4316622.75 ,8788144.84)	2.54(2.45,2.64)	738.87(514.68,1011.24)	-0.30(-0.41,-0.19)	609.81(425.57,837.38)	865.78(603.00,1184.81)	0.704354692
Depressive disorders	Low-middle SDI	11026538.37(7715897.5 1,15191252.58)	1.63(1.46,1.80)	654.34(458.32,897.85)	-0.51(-0.66,-0.35)	527.91(370.03,725.17)	778.68(545.17,1063.16)	0.677962222
Depressive disorders	Middle SDI	13541947.42(9515935.0 3,18454506.60)	1.65(1.58,1.72)	521.68(366.80,709.93)	-0.18(-0.24,-0.12)	405.87(283.84,557.57)	636.34(446.63,868.13)	0.637818948
Headache disorders	High SDI	6862292.80(1587513.84 ,14597883.00)	0.75(0.72,0.77)	636.85(134.31,1375.80)	0.01(-0.02,0.04)	438.67(104.37,916.10)	843.81(162.63,1835.84)	0.519872208
Headache disorders	High-middle SDI	9324533.28(2334293.39 ,19464833.36)	1.11(1.03,1.18)	588.30(135.50,1253.88)	0.06(0.05,0.08)	434.76(109.97,912.04)	745.68(157.93,1602.40)	0.583040795
Headache disorders	Low SDI	5256507.70(977975.26, 11447931.02)	2.87(2.85,2.90)	527.38(112.47,1121.59)	0.00(-0.01,0.01)	418.75(90.89,892.26)	634.79(132.58,1353.50)	0.659663368
Headache disorders	Low-middle SDI	10273363.12(1823619.2	1.97(1.95,2.00)	574.85(106.87,1248.43)	0.02(0.01,0.04)	449.30(88.45,974.04)	700.09(124.77,1551.93)	0.641772126

		3,22369461.67)						
Headache disorders	Middle SDI	14875826.75(2936371.64,32612740.43)	1.69(1.61,1.77)	581.76(111.38,1284.82)	0.19(0.16,0.21)	445.44(97.95,946.79)	719.17(124.73,1608.06)	0.619382783
Idiopathic epilepsy	High SDI	1281948.55(883071.20, 1887128.17)	0.80(0.71,0.88)	120.16(81.78,176.38)	-0.11(-0.19,-0.03)	130.36(90.33,190.27)	109.81(72.23,164.11)	1.187161449
Idiopathic epilepsy	High-middle SDI	1760650.59(1267274.64,2360202.12)	-0.49(-0.58,-0.40)	124.84(89.53,168.73)	-1.21(-1.30,-1.12)	139.31(101.70,185.24)	110.36(77.22,152.17)	1.262336671
Idiopathic epilepsy	Low SDI	2804185.69(2102968.24,3691130.36)	1.57(1.53,1.61)	246.92(188.89,321.76)	-0.87(-0.91,-0.83)	281.84(217.88,361.25)	212.85(156.02,283.03)	1.324169886
Idiopathic epilepsy	Low-middle SDI	3744714.70(2910586.35,4799430.05)	0.25(0.19,0.31)	213.08(166.07,271.60)	-1.08(-1.14,-1.02)	225.67(176.00,286.31)	200.56(153.75,256.88)	1.125216578
Idiopathic epilepsy	Middle SDI	3477914.00(2556903.29,4508754.39)	-0.02(-0.09,0.06)	147.56(108.63,191.96)	-0.96(-1.02,-0.91)	161.85(120.96,210.00)	133.22(96.20,176.49)	1.214880137
Motor neuron disease	High SDI	547119.40(518174.45,570407.43)	2.22(2.15,2.29)	34.94(33.35,36.39)	0.33(0.26,0.40)	41.11(38.98,42.69)	29.16(27.53,30.66)	1.40984067
Motor neuron disease	High-middle SDI	249506.73(230679.90,267477.67)	1.17(1.00,1.34)	14.20(13.10,15.32)	-0.66(-0.84,-0.48)	17.08(15.58,18.63)	11.47(10.42,12.52)	1.488793588
Motor neuron disease	Low SDI	20803.41(17068.62,25153.78)	2.89(2.69,3.09)	2.54(2.02,3.23)	0.50(0.33,0.67)	2.67(1.89,3.72)	2.43(2.03,2.99)	1.101793125
Motor neuron disease	Low-middle SDI	72956.17(63758.51,83082.03)	2.21(1.91,2.51)	4.63(4.04,5.28)	0.64(0.43,0.85)	5.21(4.39,6.16)	4.08(3.55,4.81)	1.278552989
Motor neuron disease	Middle SDI	143598.29(129350.26,157230.34)	0.50(-0.04,1.03)	5.60(5.05,6.11)	-1.12(-1.54,-0.69)	6.59(5.86,7.32)	4.62(4.10,5.08)	1.424930489
Multiple sclerosis	High SDI	500325.08(411436.08,581040.50)	1.52(1.45,1.59)	35.38(29.05,41.50)	0.10(0.05,0.16)	24.47(18.99,29.51)	46.28(36.86,55.31)	0.528798156
Multiple sclerosis	High-middle SDI	260398.48(207342.42,3	0.37(0.26,0.47)	14.03(11.15,19.37)	-1.33(-1.43,-1.23)	11.58(9.08,17.56)	16.46(12.87,23.37)	0.703774539

		59882.44)						
Multiple sclerosis	Low SDI	60580.17(47505.28,77711.86)	3.24(3.19,3.30)	8.09(6.35,10.20)	0.32(0.31,0.34)	6.27(4.76,8.08)	9.89(7.61,14.23)	0.633436796
Multiple sclerosis	Low-middle SDI	138239.16(116847.85,167903.68)	2.88(2.83,2.92)	8.52(7.24,10.30)	0.40(0.35,0.44)	6.97(5.74,8.50)	10.05(8.08,12.95)	0.693791297
Multiple sclerosis	Middle SDI	199666.15(168751.17,241140.76)	2.52(2.45,2.58)	7.48(6.33,9.08)	0.21(0.18,0.24)	6.59(5.21,8.79)	8.38(6.91,10.60)	0.78614664
Neurological disorders	High SDI	19237221.62(11714530.22,30377786.65)	1.72(1.68,1.76)	1303.51(736.19,2111.81)	0.07(0.05,0.09)	1113.90(699.22,1736.58)	1495.57(773.61,2526.92)	0.74480178
Neurological disorders	High-middle SDI	20676710.10(11919901.38,33556099.45)	1.55(1.54,1.57)	1218.74(691.84,1981.10)	-0.11(-0.13,-0.09)	1082.73(670.43,1686.23)	1354.95(706.71,2268.98)	0.799095634
Neurological disorders	Low SDI	10231802.11(5619983.15,17107547.80)	2.51(2.48,2.54)	1236.62(720.11,1946.39)	-0.10(-0.11,-0.09)	1156.37(717.53,1780.50)	1313.78(716.69,2180.33)	0.880187407
Neurological disorders	Low-middle SDI	19243323.01(10170116.96,32046998.27)	1.85(1.82,1.87)	1243.81(695.95,2023.73)	-0.12(-0.14,-0.10)	1134.81(681.23,1774.12)	1352.13(711.61,2298.17)	0.839276598
Neurological disorders	Middle SDI	28280338.62(15309773.36,46788483.78)	1.90(1.88,1.92)	1217.94(673.68,2029.28)	0.01(0.00,0.02)	1089.16(653.16,1719.00)	1343.31(686.21,2312.50)	0.810800693
Parkinson's disease	High SDI	1604208.01(1454059.98,1725513.24)	2.78(2.72,2.84)	76.55(69.65,82.39)	0.48(0.40,0.55)	107.65(98.79,115.39)	53.14(47.34,57.90)	2.025928922
Parkinson's disease	High-middle SDI	1553399.66(1403032.70,1701395.46)	2.35(2.29,2.42)	76.75(69.40,84.08)	-0.27(-0.35,-0.19)	105.70(95.06,116.35)	57.08(50.34,63.45)	1.851565538
Parkinson's disease	Low SDI	318441.47(282135.67,364300.98)	2.90(2.82,2.97)	78.37(69.22,90.27)	0.07(0.02,0.11)	95.49(82.17,113.05)	63.14(54.24,74.35)	1.512297772
Parkinson's disease	Low-middle SDI	1012325.78(908057.34,1129431.48)	3.39(3.32,3.45)	85.44(76.67,95.24)	0.04(-0.03,0.12)	106.19(93.11,120.07)	68.07(58.41,79.56)	1.559863126
Parkinson's disease	Middle SDI	1800862.09(1629326.97	3.47(3.41,3.52)	82.22(74.03,90.57)	0.02(-0.05,0.09)	105.42(93.40,118.22)	63.51(55.51,70.43)	1.65987205

		,1993459.08)						
Schizophrenia	High SDI	2541568.15(1865247.53 ,3207291.30)	0.96(0.90,1.02)	213.36(155.49,271.87)	0.04(0.00,0.09)	221.36(162.06,282.69)	204.62(148.87,259.42)	1.081768104
Schizophrenia	High-middle SDI	3367871.62(2476407.48 ,4227909.76)	1.49(1.44,1.53)	194.45(142.55,244.50)	0.13(0.11,0.15)	204.48(150.65,256.47)	184.13(135.51,231.70)	1.11048893
Schizophrenia	Low SDI	1304857.92(936059.49, 1691711.88)	2.93(2.89,2.97)	150.61(108.07,193.89)	0.04(0.02,0.05)	163.81(117.86,211.65)	137.63(98.54,176.22)	1.190193177
Schizophrenia	Low-middle SDI	3006048.43(2167795.59 ,3849888.00)	2.26(2.25,2.28)	174.30(125.23,222.16)	0.04(0.03,0.05)	192.82(138.75,246.77)	155.97(112.23,199.22)	1.236278164
Schizophrenia	Middle SDI	4878967.45(3547734.68 ,6228126.98)	1.92(1.85,1.99)	182.38(133.36,232.51)	0.02(0.01,0.02)	192.92(140.92,247.00)	171.87(125.98,219.89)	1.122505325
Stroke	High SDI	12973818.17(11716037. 91,14044417.40)	-0.68(-0.83,-0.54)	703.13(640.03,761.95)	-2.66(-2.80,-2.53)	775.13(717.92,831.32)	634.85(566.90,697.43)	1.220972823
Stroke	High-middle SDI	34933599.23(32080999. 72,37584859.38)	-0.15(-0.38,0.08)	1750.21(1606.12,1882.0 9)	-2.41(-2.66,-2.16)	2079.62(1867.15,2304.51)	1462.13(1317.49,1605.46)	1.422327574
Stroke	Low SDI	12028395.10(10644696. 58,13563094.15)	1.55(1.48,1.62)	2162.03(1912.56,2441.6 8)	-1.08(-1.12,-1.03)	2225.29(1934.12,2550.34)	2097.54(1838.03,2367.28)	1.060904672
Stroke	Low-middle SDI	30370492.21(27803534. 42,33055330.08)	1.68(1.63,1.72)	2220.33(2036.51,2414.4 5)	-1.07(-1.16,-0.98)	2443.84(2208.55,2688.16)	2010.72(1795.22,2240.43)	1.215405487
Stroke	Middle SDI	52844428.47(48314527. 43,57558906.03)	1.46(1.39,1.54)	2199.78(2017.08,2393.2 2)	-1.44(-1.55,-1.32)	2578.24(2304.27,2873.26)	1848.14(1652.39,2064.34)	1.395044082

CI - confidence interval, EAPC - estimated annual percentage change, SDI - socio-demographic index, UI - uncertainty interval.

Table S13. Global burden of neurological disorders attributable to risk factors in 1990 and 2019, and the temporal trends from 1990 to 2019

Locations	2019 Age-standardized DALY rate (95% UI)								EAPC (95% CI) (1990-2019)							
	**All risk factors**	**Behavioral risks**	**Tobacco**	**Smoking**	**Alcohol use**	**Metabolic risks**	**High fasting plasma glucose**	**High body-mass index**	**All risk factors**	**Behavioral risks**	**Tobacco**	**Smoking**	**Alcohol use**	**Metabolic risks**	**High fasting plasma glucose**	**High body-mass index**
**global**	122.40(53.39,286.69)	62.36(29.43,129.99)	45.19(14.11,110.93)	45.19(14.11,110.93)	17.16(11.54,23.99)	70.70(20.77,179.60)	32.84(5.53,105.88)	42.22(11.24,107.37)	0.20(0.16,0.24)	-0.52(-0.57,-0.47)	-0.58(-0.64,-0.52)	-0.58(-0.64,-0.52)	-0.36(-0.40,-0.33)	1.00(0.93,1.06)	1.23(1.11,1.34)	0.93(0.89,0.97)
**sdi**																
High-middle SDI	132.75(58.24,312.27)	66.85(31.45,142.92)	49.94(16.12,120.10)	49.94(16.12,120.10)	16.91(11.15,24.59)	78.00(23.35,194.42)	31.64(5.40,101.61)	51.13(14.09,128.38)	0.36(0.27,0.45)	-0.18(-0.26,-0.09)	0.18(0.07,0.28)	0.18(0.07,0.28)	-1.09(-1.19,-0.98)	0.98(0.85,1.12)	1.22(0.96,1.48)	0.93(0.86,0.99)
High SDI	141.78(68.62,306.20)	78.84(40.71,151.59)	55.70(20.98,127.09)	55.70(20.98,127.09)	23.14(14.82,35.27)	77.13(23.40,188.03)	34.05(6.12,108.13)	48.51(13.77,119.05)	-0.13(-0.16,-0.10)	-0.85(-0.89,-0.81)	-0.99(-1.02,-0.96)	-0.99(-1.02,-0.96)	-0.47(-0.57,-0.38)	0.78(0.73,0.83)	1.39(1.21,1.57)	0.52(0.46,0.58)
Low-middle SDI	96.39(40.04,236.24)	48.73(22.22,105.35)	32.47(7.45,87.77)	32.47(7.45,87.77)	16.26(10.84,22.88)	54.18(13.75,151.31)	31.10(5.17,102.47)	25.82(5.94,70.31)	0.64(0.62,0.67)	-0.26(-0.27,-0.24)	-0.62(-0.65,-0.58)	-0.62(-0.65,-0.58)	0.59(0.52,0.67)	1.75(1.69,1.81)	1.30(1.25,1.36)	2.55(2.45,2.64)
Low SDI	82.67(35.08,199.05)	39.70(20.31,78.49)	22.59(5.62,59.79)	22.59(5.62,59.79)	17.11(10.87,24.87)	46.90(11.43,134.84)	28.12(4.54,96.25)	20.65(4.29,59.46)	0.62(0.59,0.66)	-0.15(-0.18,-0.13)	-0.35(-0.37,-0.33)	-0.35(-0.37,-0.33)	0.13(0.06,0.19)	1.50(1.46,1.54)	1.17(1.12,1.22)	2.14(2.06,2.23)
Middle SDI	114.09(45.99,277.45)	56.78(24.67,125.48)	41.98(11.09,107.67)	41.98(11.09,107.67)	14.80(9.80,20.95)	66.24(18.44,173.65)	33.53(5.51,108.94)	36.54(8.96,98.38)	0.57(0.52,0.63)	-0.18(-0.28,-0.09)	-0.21(-0.33,-0.09)	-0.21(-0.33,-0.09)	-0.11(-0.16,-0.05)	1.44(1.37,1.50)	0.92(0.82,1.01)	2.14(2.10,2.18)

regions

Andean Latin America	115.02(50.77,242.28)	43.72(23.79,73.20)	15.41(3.42,40.79)	15.41(3.42,40.79)	28.32(15.14,45.13)	75.64(21.61,191.10)	30.17(5.04,99.08)	50.30(12.63,129.20)	0.37(0.33,0.41)	-1.18(-1.24,-1.13)	-1.45(-1.51,-1.40)	-1.45(-1.51,-1.40)	-1.02(-1.12,-0.91)	1.64(1.58,1.70)	1.44(1.37,1.50)	1.91(1.85,1.96)
Australasia	140.07(69.42,293.84)	66.78(36.28,119.69)	39.64(14.08,91.51)	39.64(14.08,91.51)	27.15(15.27,45.52)	84.12(26.16,202.96)	28.78(5.01,94.74)	60.76(17.39,151.13)	-0.16(-0.21,-0.11)	-1.31(-1.34,-1.28)	-1.62(-1.66,-1.57)	-1.62(-1.66,-1.57)	-0.80(-0.88,-0.72)	0.96(0.83,1.10)	1.11(0.86,1.36)	0.99(0.90,1.09)
Caribbean	137.45(63.95,306.07)	70.59(36.34,137.05)	40.47(11.24,104.48)	40.47(11.24,104.48)	30.12(19.17,43.86)	78.74(22.57,208.52)	40.04(7.09,125.94)	44.78(12.01,115.55)	0.21(0.15,0.27)	-0.51(-0.55,-0.46)	-0.51(-0.56,-0.47)	-0.51(-0.56,-0.47)	-0.50(-0.67,-0.33)	1.08(1.01,1.15)	0.84(0.78,0.90)	1.45(1.35,1.54)
Central Asia	145.91(71.30,312.01)	67.19(39.04,120.67)	33.32(9.80,84.59)	33.32(9.80,84.59)	33.88(21.75,48.71)	87.31(26.19,220.93)	32.82(5.31,107.18)	#N/A	0.70(0.63,0.77)	-0.06(-0.21,0.08)	0.33(0.10,0.56)	0.33(0.10,0.56)	-0.39(-0.81,0.04)	1.53(1.49,1.57)	2.52(2.42,2.62)	1.21(1.14,1.27)
Central Europe	178.30(90.03,368.85)	94.71(52.12,176.44)	57.39(20.59,138.76)	57.39(20.59,138.76)	37.32(24.97,54.30)	101.24(32.21,250.67)	37.65(6.65,121.20)	71.33(20.38,180.28)	0.13(0.11,0.14)	-0.44(-0.45,-0.42)	-0.53(-0.56,-0.50)	-0.53(-0.56,-0.50)	-0.29(-0.35,-0.23)	0.82(0.81,0.83)	1.02(0.95,1.08)	0.82(0.80,0.84)
Central Latin America	143.74(63.99,323.36)	59.22(34.23,107.74)	26.69(6.77,70.62)	26.69(6.77,70.62)	32.53(21.10,47.39)	92.76(26.14,243.90)	46.72(8.10,148.18)	53.64(13.46,142.15)	-0.26(-0.34,-0.18)	-1.46(-1.58,-1.34)	-1.90(-1.98,-1.82)	-1.90(-1.98,-1.82)	-1.02(-1.20,-0.84)	0.65(0.61,0.70)	0.07(-0.05,0.19)	1.33(1.26,1.39)
Central sub-Saharan Africa	95.42(43.13,209.85)	41.38(22.14,72.08)	12.61(3.16,33.80)	12.61(3.16,33.80)	28.78(14.97,46.87)	56.44(13.77,159.31)	34.62(5.53,118.39)	24.07(5.15,68.19)	0.42(0.19,0.64)	-0.07(-0.41,0.26)	-1.03(-1.21,-0.85)	-1.03(-1.21,-0.85)	0.43(0.00,0.87)	0.79(0.65,0.94)	1.00(0.96,1.04)	0.51(0.14,0.88)
East Asia	109.06(4	65.33(25.76	53.66(14	53.66(14.2	11.67(7.	53.27(13.5	27.32(4.	28.07(5.69	0.74(0.6	0.35(0.18,	0.58(0.37,	0.58(0.37,0.	-0.60(-0.77,-	1.47(1.31,	0.67(0.39,	2.74(2.6

	3.29,265.60)	,149.09)	.20,133.77)	0,133.77)	59,16.90)	6,135.82)	50,88.98)	,81.53)	1,0.88)	0.51)	0.80)	80)	0.43)	1.63)	0.95)	8,2.81)
Eastern Europe	134.30(64.13,286.14)	60.70(32.67,114.64)	37.52(12.59,89.47)	37.52(12.59,89.47)	23.18(14.96,34.02)	83.50(26.07,203.62)	19.78(3.07,65.03)	67.86(19.44,169.03)	0.55(0.49,0.60)	-0.04(-0.15,0.07)	1.05(0.93,1.17)	1.05(0.93,1.17)	-1.33(-1.63,-1.04)	1.23(1.17,1.29)	1.24(1.13,1.36)	1.29(1.22,1.36)
Eastern Sub-Saharan Africa	84.93(39.90,194.03)	41.00(23.18,74.38)	18.70(4.56,50.34)	18.70(4.56,50.34)	22.30(14.19,33.39)	46.84(11.54,129.77)	23.51(3.72,81.97)	24.97(5.40,72.35)	0.42(0.39,0.46)	-0.55(-0.61,-0.49)	-0.71(-0.78,-0.63)	-0.71(-0.78,-0.63)	-0.41(-0.50,-0.33)	1.59(1.52,1.66)	0.77(0.71,0.83)	2.75(2.58,2.92)
High-income Asia Pacific	111.53(49.25,257.35)	66.75(30.10,137.86)	52.83(17.54,124.97)	52.83(17.54,124.97)	13.92(8.31,22.36)	53.03(13.57,135.21)	28.40(4.81,91.03)	26.63(5.30,75.14)	0.15(0.08,0.22)	-0.45(-0.53,-0.37)	-0.19(-0.31,-0.07)	-0.19(-0.31,-0.07)	-1.39(-1.49,-1.28)	1.10(1.02,1.17)	1.00(0.91,1.09)	1.22(1.08,1.35)
High-income North America	157.52(75.36,337.38)	81.35(40.81,161.45)	62.92(25.18,138.83)	62.92(25.18,138.83)	18.42(11.31,27.77)	95.97(30.45,223.72)	40.41(7.58,125.58)	63.69(18.60,152.15)	0.01(-0.07,0.09)	-1.02(-1.07,-0.97)	-1.32(-1.38,-1.27)	-1.32(-1.38,-1.27)	0.13(-0.04,0.30)	1.22(1.09,1.36)	2.36(1.88,2.85)	0.85(0.75,0.95)
North Africa and Middle East	152.62(56.47,371.89)	57.43(21.05,137.70)	54.03(17.76,133.86)	54.03(17.76,133.86)	3.40(1.88,5.53)	111.71(34.19,279.74)	48.25(8.67,151.54)	72.56(20.98,181.16)	0.66(0.63,0.68)	-0.43(-0.49,-0.37)	-0.42(-0.48,-0.35)	-0.42(-0.48,-0.35)	-0.61(-0.77,-0.46)	1.47(1.42,1.52)	1.89(1.77,2.01)	1.39(1.36,1.42)
Oceania	99.73(34.90,262.49)	39.38(12.90,96.82)	28.78(3.81,86.65)	28.78(3.81,86.65)	10.61(5.06,18.26)	68.67(16.86,195.59)	43.97(7.69,142.63)	29.07(6.21,81.69)	0.17(0.11,0.23)	-0.52(-0.66,-0.38)	-0.57(-0.66,-0.47)	-0.57(-0.66,-0.47)	-0.39(-0.67,-0.12)	0.64(0.61,0.66)	1.04(1.03,1.06)	0.18(0.11,0.26)
South Asia	77.34(31.	38.19(16.70	26.28(6.	26.28(6.00	11.91(7.	44.12(11.0	27.86(4.	18.27(4.12	0.80(0.7	-0.20(-	-0.59(-	-0.59(-0.67,-	0.81(0.69,0.93	2.06(1.95,	1.47(1.37,	3.46(3.2

	21,196.01)	,83.71)	00,70.44)	,70.44)	55,17.53)	0,128.61)	52,93.07)	,51.32)	2,0.88)	0.27,-0.14)	0.67,-0.51)	0.51)	)	2.17)	1.56)	8,3.64)
Southeast Asia	101.45(38.42,256.02)	48.82(18.97,112.71)	37.86(8.17,100.83)	37.86(8.17,100.83)	10.97(6.70,16.97)	60.01(15.10,169.50)	37.01(5.97,123.69)	25.81(5.69,71.50)	0.85(0.81,0.90)	-0.16(-0.23,-0.08)	-0.65(-0.73,-0.56)	-0.65(-0.73,-0.56)	2.38(2.23,2.53)	2.04(1.98,2.10)	1.48(1.41,1.56)	3.31(3.24,3.37)
Southern Latin America	134.46(63.77,297.31)	66.71(34.09,125.84)	40.16(12.72,98.30)	40.16(12.72,98.30)	26.56(15.13,42.48)	79.21(23.58,197.95)	32.84(5.72,108.66)	51.92(14.15,130.53)	0.42(0.39,0.44)	-0.42(-0.51,-0.33)	-0.18(-0.23,-0.12)	-0.18(-0.23,-0.12)	-0.78(-0.97,-0.58)	1.43(1.33,1.53)	1.60(1.53,1.67)	1.45(1.28,1.63)
Southern sub-Saharan Africa	152.57(74.05,330.80)	66.74(40.43,111.22)	27.16(8.07,68.48)	27.16(8.07,68.48)	39.58(25.94,55.96)	93.69(28.29,238.35)	37.39(5.98,124.65)	63.74(17.23,166.74)	-0.21(-0.35,-0.08)	-1.65(-1.91,-1.39)	-2.37(-2.48,-2.26)	-2.37(-2.48,-2.26)	-1.01(-1.43,-0.59)	1.14(1.08,1.19)	0.97(0.84,1.10)	1.36(1.32,1.40)
Tropical Latin America	172.38(79.50,389.32)	85.72(43.64,169.64)	57.07(18.98,138.42)	57.07(18.98,138.42)	28.65(18.60,42.52)	102.81(31.53,251.14)	40.68(6.93,132.70)	69.35(19.40,175.14)	0.01(-0.01,0.03)	-1.24(-1.30,-1.18)	-1.58(-1.74,-1.43)	-1.58(-1.74,-1.43)	-0.39(-0.60,-0.18)	1.43(1.35,1.51)	0.65(0.57,0.73)	2.09(1.94,2.24)
Western Europe	149.99(75.63,319.26)	85.06(45.83,159.41)	53.58(19.65,121.84)	53.58(19.65,121.84)	31.47(19.83,49.26)	78.82(24.23,195.80)	34.54(6.02,111.41)	49.94(13.66,122.51)	-0.02(-0.09,0.04)	-0.61(-0.67,-0.55)	-0.80(-0.86,-0.75)	-0.80(-0.86,-0.75)	-0.25(-0.37,-0.14)	0.71(0.62,0.80)	1.07(0.93,1.21)	0.58(0.50,0.65)
Western sub-Saharan Africa	98.07(46.30,221.78)	43.23(25.82,70.89)	12.97(3.23,35.23)	12.97(3.23,35.23)	30.26(19.27,44.83)	57.27(14.36,161.92)	27.10(4.12,94.74)	32.86(7.27,93.74)	1.01(0.95,1.08)	0.22(0.09,0.36)	-0.07(-0.11,-0.04)	-0.07(-0.11,-0.04)	0.36(0.17,0.54)	1.80(1.76,1.84)	1.16(1.11,1.21)	2.56(2.51,2.61)

countries																
Afghanistan	133.17(42.10,359.27)	37.25(10.85,97.42)	36.55(10.20,96.71)	36.55(10.20,96.71)	0.70(0.21,1.49)	104.97(28.60,292.15)	62.39(10.88,199.36)	49.88(12.56,130.16)	1.48(1.27,1.69)	0.73(0.60,0.87)	0.64(0.49,0.79)	0.64(0.49,0.79)	17.14(13.98,20.38)	1.89(1.63,2.16)	1.74(1.72,1.76)	2.41(1.68,3.14)
Albania	167.86(75.16,356.56)	100.02(45.49,204.68)	74.84(25.66,183.20)	74.84(25.66,183.20)	25.18(11.47,46.90)	86.66(25.47,211.23)	25.67(4.33,82.89)	65.63(18.91,162.18)	0.73(0.62,0.84)	0.65(0.49,0.81)	0.23(0.19,0.28)	0.23(0.19,0.28)	2.12(1.31,2.93)	0.97(0.93,1.02)	0.98(0.89,1.07)	1.04(0.97,1.10)
Algeria	172.61(63.35,427.52)	71.69(25.43,174.23)	68.11(22.21,168.08)	68.11(22.21,168.08)	3.57(1.45,6.69)	121.85(36.44,312.01)	58.32(10.65,189.14)	74.40(19.75,183.25)	0.57(0.53,0.61)	-0.58(-0.63,-0.52)	-0.65(-0.71,-0.60)	-0.65(-0.71,-0.60)	1.35(1.16,1.53)	1.57(1.53,1.61)	1.64(1.60,1.68)	1.74(1.69,1.80)
American Samoa	145.47(48.26,381.19)	39.80(9.18,96.60)	31.77(3.20,92.48)	31.77(3.20,92.48)	8.02(2.46,16.94)	121.54(34.04,328.69)	72.32(13.61,227.77)	62.96(15.87,161.52)	0.25(0.22,0.29)	-0.09(-0.28,0.09)	-0.29(-0.39,-0.19)	-0.29(-0.39,-0.19)	0.90(0.20,1.61)	0.34(0.31,0.37)	0.81(0.76,0.85)	-0.03(-0.10,0.04)
Andorra	134.68(65.75,281.13)	75.69(38.47,145.51)	49.58(18.33,112.91)	49.58(18.33,112.91)	26.11(12.40,49.28)	71.24(20.91,172.77)	27.42(4.80,86.52)	48.34(13.43,121.47)	-0.20(-0.24,-0.17)	-0.85(-0.88,-0.81)	-0.74(-0.77,-0.70)	-0.74(-0.77,-0.70)	-1.05(-1.13,-0.96)	0.66(0.59,0.73)	1.47(1.43,1.50)	0.38(0.29,0.46)
Angola	130.72(62.78,256.98)	78.91(38.72,131.18)	23.34(6.87,61.36)	23.34(6.87,61.36)	55.57(24.64,96.77)	55.99(13.48,162.55)	34.02(5.54,117.29)	24.10(4.90,70.02)	2.04(1.87,2.22)	2.01(1.72,2.30)	-0.09(-0.20,0.01)	-0.09(-0.20,0.01)	3.23(2.77,3.70)	2.03(1.93,2.13)	1.23(1.18,1.27)	3.97(3.73,4.21)
Antigua and Barbuda	153.62(78.52,316.19)	80.27(46.51,133.62)	24.05(5.89,62.32)	24.05(5.89,62.32)	56.22(33.08,94.28)	80.57(22.30,220.12)	46.15(8.18,147.04)	40.44(10.04,109.01)	0.86(0.72,1.00)	0.54(0.31,0.78)	0.34(0.23,0.45)	0.34(0.23,0.45)	0.64(0.34,0.94)	1.26(1.22,1.30)	1.07(1.02,1.12)	1.68(1.64,1.73)
Argentina	131.59(59.80,294.49)	66.89(30.87,131.79)	45.16(14.95,112.45)	45.16(14.95,112.45)	21.73(10.87,38.66)	77.27(23.05,194.42)	32.03(5.57,106.51)	50.42(13.34,127.10)	0.36(0.32,0.40)	-0.41(-0.50,-0.33)	-0.11(-0.17,-0.05)	-0.11(-0.17,-0.05)	-1.00(-1.20,-0.80)	1.34(1.24,1.44)	1.25(1.10,1.39)	1.50(1.32,1.68)

Armenia	137.58(54.00,313.38)	58.21(24.40,123.46)	45.25(13.40,112.05)	45.25(13.40,112.05)	12.96(5.00,26.17)	91.35(28.00,231.27)	33.81(5.59,109.49)	63.65(17.14,159.29)	1.24(1.13,1.35)	0.10(-0.07,0.28)	0.40(0.12,0.67)	0.40(0.12,0.67)	-0.80(-1.03,-0.56)	2.33(2.21,2.46)	2.09(1.90,2.27)	2.63(2.44,2.82)
Australia	140.12(69.07,291.66)	65.70(35.15,118.01)	38.72(13.93,89.53)	38.72(13.93,89.53)	26.98(14.19,47.72)	85.13(26.36,202.76)	29.08(5.05,95.66)	61.60(17.41,152.50)	-0.07(-0.14,-0.01)	-1.18(-1.21,-1.15)	-1.50(-1.54,-1.45)	-1.50(-1.54,-1.45)	-0.66(-0.76,-0.56)	0.95(0.80,1.10)	1.03(0.74,1.31)	1.02(0.91,1.12)
Austria	139.85(68.57,292.69)	84.59(43.34,161.40)	58.01(21.47,132.32)	58.01(21.47,132.32)	26.57(13.10,50.84)	69.30(20.44,168.11)	26.94(4.62,88.03)	46.67(12.81,117.46)	0.45(0.44,0.47)	0.27(0.20,0.34)	0.97(0.81,1.12)	0.97(0.81,1.12)	-0.95(-1.11,-0.79)	0.99(0.94,1.04)	1.83(1.75,1.91)	0.69(0.64,0.74)
Azerbaijan	153.53(66.06,350.84)	76.18(35.35,157.14)	46.92(12.75,120.92)	46.92(12.75,120.92)	29.26(14.04,53.38)	89.79(25.72,226.34)	33.25(5.35,113.74)	62.35(16.22,158.78)	1.13(0.93,1.33)	0.52(0.26,0.77)	1.49(1.28,1.71)	1.49(1.28,1.71)	-0.65(-0.95,-0.35)	2.03(1.87,2.19)	2.58(2.44,2.72)	1.92(1.63,2.20)
Bahamas	129.14(57.57,292.13)	52.98(25.17,97.44)	21.97(5.83,58.62)	21.97(5.83,58.62)	31.01(11.75,60.10)	82.98(23.33,216.23)	40.68(6.91,134.26)	48.91(12.73,130.29)	-0.32(-0.38,-0.26)	-1.53(-1.67,-1.38)	-0.39(-0.48,-0.29)	-0.39(-0.48,-0.29)	-2.19(-2.35,-2.02)	0.76(0.70,0.81)	0.89(0.86,0.92)	0.76(0.65,0.87)
Bahrain	203.70(76.08,504.82)	66.00(24.95,153.87)	59.06(19.52,147.58)	59.06(19.52,147.58)	6.94(2.89,12.81)	163.81(51.36,416.76)	94.76(18.78,285.18)	90.41(24.90,226.41)	0.27(0.22,0.32)	-1.03(-1.09,-0.97)	-0.45(-0.48,-0.42)	-0.45(-0.48,-0.42)	-4.17(-4.48,-3.85)	0.97(0.86,1.08)	1.42(1.22,1.62)	0.80(0.71,0.88)
Bangladesh	60.64(20.73,157.04)	32.37(9.30,83.24)	31.44(7.94,82.24)	31.44(7.94,82.24)	0.94(0.10,2.29)	32.66(7.49,91.26)	22.92(3.78,74.43)	10.66(2.03,31.32)	-0.02(-0.12,0.09)	-1.09(-1.20,-0.97)	-1.16(-1.27,-1.04)	-1.16(-1.27,-1.04)	2.99(2.68,3.31)	1.47(1.24,1.71)	0.87(0.57,1.18)	3.68(3.58,3.78)
Barbados	142.22(69.05,304.40)	62.47(35.34,101.36)	21.27(6.30,55.48)	21.27(6.30,55.48)	41.20(20.77,68.11)	85.96(24.11,231.96)	42.48(7.33,134.94)	50.56(12.99,133.13)	0.08(0.03,0.13)	-0.59(-0.66,-0.51)	-0.19(-0.25,-0.13)	-0.19(-0.25,-0.13)	-0.79(-0.92,-0.66)	0.66(0.57,0.75)	0.80(0.71,0.89)	0.63(0.53,0.72)
Belarus	142.96(71.50,299.	72.61(40.59,128.80)	37.41(12.55,91.54)	37.41(12.55,91.54)	35.20(19.57,58.32)	79.35(23.81,201.45)	18.40(2.80,62.28)	64.48(17.52,165.71)	0.22(0.15,0.28)	-0.49(-0.61,-0.38)	-0.29(-0.43,-0.14)	-0.29(-0.43,-0.14)	-0.69(-1.01,-0.38)	1.09(1.05,1.14)	0.53(0.39,0.67)	1.31(1.22,1.39)

	30)															
Belgium	152.22(76.85,315.00)	97.30(50.75,179.75)	59.55(21.27,136.67)	59.55(21.27,136.67)	37.75(19.10,68.50)	69.50(20.59,174.02)	28.42(4.86,92.00)	45.43(12.13,115.77)	-0.08(-0.18,0.02)	-0.41(-0.54,-0.29)	-0.79(-0.90,-0.69)	-0.79(-0.90,-0.69)	0.25(0.02,0.47)	0.52(0.45,0.59)	0.87(0.80,0.93)	0.41(0.33,0.50)
Belize	142.03(64.44,312.61)	66.47(34.13,118.68)	30.50(7.66,81.99)	30.50(7.66,81.99)	35.97(19.41,58.21)	84.39(24.38,217.16)	35.66(6.02,116.15)	55.09(14.50,143.51)	0.77(0.63,0.91)	-0.09(-0.19,0.01)	-0.56(-0.74,-0.39)	-0.56(-0.74,-0.39)	0.37(0.11,0.63)	1.63(1.40,1.87)	1.36(1.26,1.46)	1.98(1.63,2.32)
Benin	96.50(40.26,230.57)	37.32(19.39,67.19)	13.94(3.12,39.04)	13.94(3.12,39.04)	23.38(10.87,41.31)	62.07(15.08,172.94)	32.62(5.06,112.43)	32.49(7.21,90.95)	0.79(0.72,0.85)	-0.32(-0.46,-0.17)	-1.52(-1.65,-1.38)	-1.52(-1.65,-1.38)	0.71(0.58,0.85)	1.62(1.56,1.68)	1.15(1.09,1.20)	2.33(2.20,2.46)
Bermuda	137.18(63.15,304.36)	58.77(27.29,111.37)	31.06(8.67,80.80)	31.06(8.67,80.80)	27.71(11.45,55.52)	88.50(26.18,219.68)	32.40(5.52,104.24)	62.52(16.99,159.63)	-0.17(-0.23,-0.11)	-1.08(-1.20,-0.97)	0.29(0.25,0.33)	0.29(0.25,0.33)	-2.26(-2.47,-2.04)	0.70(0.68,0.72)	0.84(0.81,0.88)	0.71(0.67,0.74)
Bhutan	74.75(25.71,192.64)	30.00(10.81,70.52)	23.69(5.95,64.00)	23.69(5.95,64.00)	6.32(2.27,13.28)	49.00(12.11,139.44)	27.67(4.45,91.79)	23.53(4.82,69.60)	0.40(0.32,0.48)	-1.52(-1.69,-1.35)	0.26(0.16,0.37)	0.26(0.16,0.37)	-5.01(-5.49,-4.52)	2.62(2.57,2.68)	2.12(2.10,2.15)	3.51(3.37,3.66)
Bolivia (Plurinational State of)	118.03(48.82,267.06)	44.11(21.64,81.97)	17.16(2.53,51.13)	17.16(2.53,51.13)	26.95(12.24,47.19)	78.97(21.71,211.23)	35.22(5.64,116.82)	48.75(11.61,127.31)	0.32(0.20,0.44)	-1.50(-1.72,-1.28)	-1.80(-1.94,-1.66)	-1.80(-1.94,-1.66)	-1.29(-1.57,-1.01)	1.84(1.78,1.91)	1.22(1.16,1.27)	2.53(2.43,2.64)
Bosnia and Herzegovina	177.28(79.76,391.45)	103.49(46.96,215.91)	76.36(24.71,187.64)	76.36(24.71,187.64)	27.13(13.36,47.95)	98.76(29.93,251.14)	45.28(8.26,141.67)	61.73(17.52,159.61)	0.91(0.84,0.99)	0.58(0.49,0.67)	0.81(0.73,0.88)	0.81(0.73,0.88)	-0.04(-0.24,0.17)	1.73(1.56,1.89)	1.92(1.78,2.07)	1.81(1.59,2.03)
Botswana	163.59(77.58,350.	78.94(40.49,136.99)	36.57(10.05,95.67)	36.57(10.05,95.67)	42.37(21.07,69.68)	94.87(27.09,248.11)	41.51(6.58,140.12)	60.56(14.44,162.47)	0.74(0.56,0.92)	-0.56(-0.85,-0.26)	-0.80(-0.90,-0.70)	-0.80(-0.90,-0.70)	-0.32(-0.83,0.20)	2.40(2.29,2.51)	1.68(1.57,1.78)	3.28(3.12,3.44)

	89)															
Brazil	172.24(79.30,387.83)	85.20(43.49,168.97)	56.79(19.13,138.08)	56.79(19.13,138.08)	28.40(18.28,42.51)	103.16(31.69,252.34)	40.74(6.93,133.13)	69.67(19.48,175.87)	0.02(-0.01,0.04)	-1.24(-1.30,-1.19)	-1.59(-1.75,-1.44)	-1.59(-1.75,-1.44)	-0.37(-0.59,-0.15)	1.42(1.34,1.51)	0.63(0.55,0.71)	2.10(1.94,2.25)
Brunei Darussalam	114.76(36.28,311.80)	37.41(8.64,103.00)	34.91(6.76,99.72)	34.91(6.76,99.72)	2.49(0.00,6.45)	89.40(22.33,252.26)	64.57(11.88,203.11)	30.67(6.67,87.80)	0.04(-0.01,0.10)	-1.60(-1.66,-1.53)	-1.01(-1.24,-0.79)	-1.01(-1.24,-0.79)	-5.97(-7.68,-4.24)	0.94(0.85,1.04)	0.52(0.39,0.65)	2.42(2.32,2.52)
Bulgaria	167.27(83.39,346.25)	94.29(52.19,173.66)	48.87(15.25,120.75)	48.87(15.25,120.75)	45.42(24.15,76.75)	87.13(25.72,216.56)	33.68(5.75,110.20)	59.27(16.55,149.07)	-0.06(-0.11,-0.01)	-0.33(-0.39,-0.28)	-0.65(-0.77,-0.53)	-0.65(-0.77,-0.53)	0.06(-0.11,0.23)	0.23(0.17,0.29)	0.94(0.78,1.10)	-0.08(-0.18,0.02)
Burkina Faso	107.04(50.51,229.16)	48.36(25.39,85.21)	8.20(1.80,23.17)	8.20(1.80,23.17)	40.15(18.90,73.34)	60.02(14.05,167.21)	31.90(4.87,112.04)	30.87(5.74,91.82)	0.88(0.83,0.93)	0.06(-0.03,0.15)	-1.67(-1.77,-1.57)	-1.67(-1.77,-1.57)	0.53(0.41,0.65)	1.68(1.63,1.74)	0.76(0.69,0.84)	3.22(3.12,3.31)
Burundi	79.07(37.22,165.24)	45.29(24.56,76.63)	12.84(2.78,34.81)	12.84(2.78,34.81)	32.45(16.70,55.50)	35.34(8.00,102.95)	22.56(3.55,75.55)	13.69(2.36,43.19)	-1.41(-1.60,-1.21)	-2.65(-2.95,-2.36)	-1.94(-2.05,-1.84)	-1.94(-2.05,-1.84)	-2.89(-3.26,-2.53)	1.17(1.10,1.25)	0.89(0.81,0.98)	1.76(1.68,1.84)
Cabo Verde	133.27(64.82,276.61)	60.93(33.77,97.73)	11.12(2.62,29.91)	11.12(2.62,29.91)	49.80(26.24,82.59)	75.16(20.02,200.41)	38.82(6.25,131.77)	41.20(10.13,110.64)	1.07(0.90,1.24)	-0.07(-0.32,0.17)	-1.97(-2.11,-1.82)	-1.97(-2.11,-1.82)	0.53(0.22,0.84)	2.38(2.26,2.49)	2.04(1.89,2.20)	2.96(2.88,3.05)
Cambodia	125.03(46.89,322.13)	74.92(27.91,180.53)	58.52(14.03,161.19)	58.52(14.03,161.19)	16.40(7.03,30.41)	61.33(13.77,183.54)	44.86(7.36,151.76)	18.66(3.40,57.84)	1.85(1.74,1.97)	1.15(1.04,1.25)	0.58(0.45,0.71)	0.58(0.45,0.71)	4.67(4.40,4.94)	3.42(3.25,3.59)	3.32(3.16,3.49)	3.92(3.69,4.14)
Cameroon	138.64(65.51,302.53)	60.37(32.65,102.14)	13.86(2.97,37.92)	13.86(2.97,37.92)	46.52(23.43,78.91)	81.80(20.83,223.52)	30.88(4.63,108.90)	55.90(13.38,157.23)	0.81(0.71,0.91)	0.47(0.39,0.55)	-0.71(-0.74,-0.67)	-0.71(-0.74,-0.67)	0.89(0.77,1.02)	1.09(0.93,1.26)	1.15(0.56,1.73)	1.17(1.07,1.27)

Canada	141.00(69.63,300.61)	78.94(38.88,155.83)	61.31(23.82,134.62)	61.31(23.82,134.62)	17.63(8.82,30.76)	77.54(24.44,182.17)	25.15(4.42,80.19)	56.98(16.01,140.62)	-0.49(-0.55,-0.42)	-1.49(-1.58,-1.40)	-1.71(-1.80,-1.61)	-1.71(-1.80,-1.61)	-0.65(-0.74,-0.56)	0.89(0.81,0.98)	1.00(0.89,1.10)	0.93(0.81,1.06)
Central African Republic	79.95(34.84,180.73)	37.44(18.16,67.81)	11.20(2.83,29.40)	11.20(2.83,29.40)	26.24(10.43,49.45)	44.26(9.81,132.62)	31.61(5.05,104.11)	13.91(2.43,45.66)	-0.24(-0.29,-0.19)	-1.44(-1.51,-1.37)	-1.38(-1.51,-1.25)	-1.38(-1.51,-1.25)	-1.46(-1.60,-1.32)	1.13(1.05,1.22)	1.01(0.95,1.08)	1.52(1.38,1.66)
Chad	84.49(34.20,193.67)	41.54(17.75,78.15)	16.44(3.51,48.36)	16.44(3.51,48.36)	25.10(8.95,49.27)	45.31(10.25,131.93)	29.89(4.65,104.07)	16.94(3.26,48.81)	1.54(1.45,1.63)	1.52(1.41,1.63)	-0.34(-0.42,-0.26)	-0.34(-0.42,-0.26)	3.48(3.25,3.71)	1.53(1.42,1.64)	1.20(1.04,1.35)	2.40(2.32,2.49)
Chile	143.30(69.77,294.40)	65.93(34.40,115.47)	29.15(8.74,71.61)	29.15(8.74,71.61)	36.79(17.76,64.78)	86.69(26.14,214.39)	37.31(6.85,122.32)	56.31(15.84,140.40)	0.42(0.37,0.47)	-0.53(-0.66,-0.40)	-0.26(-0.30,-0.21)	-0.26(-0.30,-0.21)	-0.73(-0.96,-0.51)	1.41(1.24,1.57)	1.94(1.74,2.14)	1.24(1.06,1.42)
China	109.84(43.49,267.67)	66.21(26.13,151.35)	54.57(14.49,135.82)	54.57(14.49,135.82)	11.64(7.49,17.06)	53.31(13.58,135.70)	27.20(4.50,88.77)	28.22(5.68,82.02)	0.75(0.62,0.89)	0.37(0.20,0.53)	0.60(0.39,0.82)	0.60(0.39,0.82)	-0.60(-0.77,-0.42)	1.48(1.32,1.64)	0.64(0.35,0.92)	2.81(2.75,2.88)
Colombia	129.37(57.79,277.73)	50.04(25.56,92.24)	23.28(6.42,61.16)	23.28(6.42,61.16)	26.76(12.49,48.64)	85.85(25.47,215.47)	41.63(7.33,133.00)	50.49(13.30,124.13)	-0.19(-0.26,-0.11)	-1.49(-1.73,-1.25)	-1.82(-2.00,-1.65)	-1.82(-2.00,-1.65)	-1.16(-1.47,-0.85)	0.76(0.67,0.86)	-0.04(-0.13,0.04)	1.68(1.53,1.83)
Comoros	70.80(23.73,187.46)	26.05(8.56,62.48)	22.04(5.12,58.81)	22.04(5.12,58.81)	4.00(0.83,9.62)	47.97(12.32,132.26)	22.13(3.38,75.54)	27.51(5.60,79.84)	0.77(0.70,0.83)	-0.01(-0.09,0.07)	-0.23(-0.33,-0.13)	-0.23(-0.33,-0.13)	1.57(1.39,1.75)	1.27(1.20,1.33)	0.83(0.73,0.93)	1.73(1.68,1.77)
Congo	137.54(61.48,292.66)	65.66(31.20,114.88)	20.88(5.39,57.50)	20.88(5.39,57.50)	44.78(18.16,83.28)	76.53(21.24,208.93)	38.49(6.38,131.54)	42.12(10.06,114.88)	0.81(0.63,1.00)	-0.09(-0.45,0.27)	0.27(0.18,0.36)	0.27(0.18,0.36)	-0.25(-0.74,0.24)	1.80(1.73,1.86)	1.33(1.26,1.40)	2.44(2.37,2.50)
Cook	143.89(5	54.79(23.28,	30.93(4.9	30.93(4.97,	23.86(11.	102.24(28.6	51.91(9.6	60.55(15.86	0.73(0.67,	1.19(1.01,1.	-0.40(-	-0.40(-0.52,-	5.00(4.40,5.60)	0.41(0.37,0.	0.70(0.61,0.	0.33(0.29,

Islands	7.35,335.85)	116.98)	7,85.57)	85.57)	51,43.73)	4,271.49)	1,164.06)	,157.33)	0.79)	36)	0.52,-0.29)	0.29)		45)	79)	0.37)
Costa Rica	143.66(63.36,327.54)	60.50(29.27,118.63)	35.03(9.79,89.59)	35.03(9.79,89.59)	25.47(11.65,47.67)	94.00(26.74,243.36)	45.27(8.04,145.26)	56.17(14.35,140.18)	-0.08(-0.16,0.00)	-1.14(-1.30,-0.99)	-1.16(-1.29,-1.04)	-1.16(-1.29,-1.04)	-1.11(-1.35,-0.87)	0.76(0.70,0.82)	0.38(0.28,0.47)	1.20(1.08,1.33)
Croatia	187.55(90.47,399.95)	104.79(51.84,209.55)	69.60(24.43,167.40)	69.60(24.43,167.40)	35.19(17.50,62.83)	104.51(32.03,256.26)	41.38(7.26,132.57)	71.50(20.89,180.58)	-0.02(-0.11,0.07)	-0.90(-1.05,-0.75)	-0.85(-1.02,-0.69)	-0.85(-1.02,-0.69)	-0.99(-1.16,-0.81)	1.12(1.07,1.17)	0.91(0.85,0.97)	1.35(1.26,1.45)
Cuba	129.78(53.33,306.81)	67.30(27.64,146.80)	51.64(14.18,135.45)	51.64(14.18,135.45)	15.66(7.51,29.16)	78.17(22.79,208.47)	38.70(6.85,124.76)	45.28(12.05,122.65)	0.07(-0.03,0.16)	-0.70(-0.75,-0.65)	-0.67(-0.70,-0.64)	-0.67(-0.70,-0.64)	-0.82(-1.12,-0.52)	1.01(0.87,1.15)	0.56(0.41,0.71)	1.57(1.40,1.74)
Cyprus	133.12(55.94,312.63)	71.26(30.20,152.11)	52.54(15.58,129.82)	52.54(15.58,129.82)	18.71(7.97,38.28)	76.52(22.21,207.07)	42.84(7.54,138.03)	38.95(9.52,104.38)	-0.40(-0.48,-0.31)	-0.74(-0.83,-0.65)	-0.54(-0.63,-0.44)	-0.54(-0.63,-0.44)	-1.26(-1.46,-1.06)	-0.09(-0.22,0.05)	-0.70(-0.89,-0.51)	0.77(0.67,0.86)
Czechia	203.41(98.15,407.91)	107.72(54.62,195.52)	63.87(22.54,151.82)	63.87(22.54,151.82)	43.85(21.05,81.00)	118.97(38.58,292.07)	55.65(10.51,173.58)	75.41(21.39,186.14)	0.25(0.20,0.30)	-0.49(-0.57,-0.41)	-0.38(-0.42,-0.33)	-0.38(-0.42,-0.33)	-0.65(-0.80,-0.50)	1.20(1.18,1.22)	1.55(1.41,1.69)	1.14(1.06,1.22)
Côte d'Ivoire	122.21(57.90,260.09)	65.50(33.74,111.52)	19.47(4.44,52.57)	19.47(4.44,52.57)	46.03(22.63,78.43)	60.65(15.31,173.82)	31.71(4.92,109.87)	31.95(6.90,92.12)	0.76(0.65,0.87)	0.48(0.31,0.65)	0.38(0.24,0.51)	0.38(0.24,0.51)	0.53(0.29,0.76)	1.16(1.10,1.21)	1.27(1.16,1.38)	1.14(1.10,1.18)
Democratic People's Republic of Korea	76.16(28.68,195.98)	42.63(16.12,102.27)	31.40(6.17,87.84)	31.40(6.17,87.84)	11.24(5.95,18.49)	37.75(7.47,111.42)	26.32(3.75,88.14)	12.41(1.39,44.67)	0.14(0.10,0.17)	-0.21(-0.27,-0.16)	0.20(0.09,0.31)	0.20(0.09,0.31)	-1.17(-1.32,-1.03)	0.71(0.68,0.74)	1.08(1.06,1.10)	0.06(0.01,0.11)
Democratic Republic of	79.09(30.	26.98(11.63,	9.17(1.89,	9.17(1.89,2	17.81(6.3	53.82(12.66	34.33(5.5	21.49(4.44,	-0.31(-	-1.42(-	-1.79(-	-1.79(-2.04,-	-1.19(-2.20,-	0.31(0.09,0.	0.91(0.86,0.	-0.65(-

the Congo	59,186.06)	51.55)	25.12)	5.12)	3,36.07)	,149.90)	7,117.58)	65.76)	0.70,0.08)	2.11,-0.73)	2.04,-1.54)	1.54)	0.17)	52)	97)	1.16,-0.15)
Denmark	152.10(76.61,317.74)	108.73(54.92,210.52)	80.19(30.92,180.63)	80.19(30.92,180.63)	28.54(15.04,50.54)	60.44(18.01,147.03)	23.36(4.03,76.70)	40.42(10.54,107.15)	-0.31(-0.44,-0.18)	-0.77(-0.94,-0.60)	-0.98(-1.09,-0.86)	-0.98(-1.09,-0.86)	-0.16(-0.55,0.24)	1.07(0.98,1.16)	1.88(1.75,2.02)	0.79(0.71,0.87)
Djibouti	80.30(26.28,214.64)	36.26(11.08,88.55)	32.22(7.41,84.95)	32.22(7.41,84.95)	4.03(1.07,9.17)	49.05(12.49,134.25)	26.12(4.05,92.44)	24.68(5.00,73.08)	0.62(0.58,0.65)	-0.71(-0.81,-0.61)	0.07(-0.02,0.16)	0.07(-0.02,0.16)	-4.12(-4.41,-3.83)	2.14(2.04,2.24)	1.02(0.95,1.08)	4.13(3.90,4.35)
Dominica	180.22(93.62,352.08)	92.66(53.33,141.03)	18.83(3.86,52.26)	18.83(3.86,52.26)	73.83(40.29,115.44)	94.56(27.04,249.06)	49.13(8.94,159.95)	54.15(14.30,144.76)	0.03(-0.07,0.13)	-0.79(-0.89,-0.68)	-0.83(-0.96,-0.69)	-0.83(-0.96,-0.69)	-0.77(-0.93,-0.62)	1.07(0.93,1.21)	1.11(1.00,1.22)	1.21(1.02,1.39)
Dominican Republic	133.77(61.57,283.94)	86.75(40.71,171.06)	52.11(13.95,133.73)	52.11(13.95,133.73)	34.63(16.57,58.05)	58.23(15.72,153.89)	25.41(4.09,84.02)	35.78(8.18,96.18)	0.71(0.64,0.79)	0.14(0.11,0.17)	-0.21(-0.26,-0.16)	-0.21(-0.26,-0.16)	0.77(0.66,0.87)	2.06(1.85,2.28)	1.91(1.72,2.10)	2.31(2.07,2.55)
Ecuador	134.82(59.82,292.90)	53.59(26.71,92.05)	19.97(4.05,54.84)	19.97(4.05,54.84)	33.62(16.65,59.18)	87.53(25.04,222.85)	38.93(6.45,131.60)	55.21(14.11,147.26)	0.28(0.19,0.36)	-1.27(-1.44,-1.10)	-2.54(-2.63,-2.45)	-2.54(-2.63,-2.45)	-0.23(-0.50,0.04)	1.58(1.52,1.65)	1.76(1.61,1.91)	1.63(1.58,1.68)
Egypt	150.75(55.29,362.46)	68.64(22.32,171.11)	67.60(21.04,170.41)	67.60(21.04,170.41)	1.05(0.32,2.40)	101.94(30.77,248.87)	38.50(6.66,124.65)	71.10(19.02,173.98)	1.11(1.06,1.16)	1.09(1.03,1.14)	1.09(1.04,1.15)	1.09(1.04,1.15)	0.99(0.61,1.37)	1.41(1.35,1.46)	2.41(2.30,2.51)	1.10(0.99,1.21)
El Salvador	122.28(48.52,277.91)	39.22(18.77,75.65)	19.83(5.58,52.73)	19.83(5.58,52.73)	19.39(8.62,36.74)	88.95(24.78,226.06)	42.42(7.15,133.83)	53.23(13.42,138.13)	0.89(0.82,0.96)	-0.43(-0.64,-0.22)	-0.48(-0.77,-0.18)	-0.48(-0.77,-0.18)	-0.36(-0.61,-0.11)	1.63(1.45,1.82)	1.46(1.36,1.57)	1.95(1.67,2.22)
Equatorial Guinea	161.06(76.66,338.55)	71.64(32.39,130.90)	15.48(3.84,43.13)	15.48(3.84,43.13)	56.16(21.79,107.49)	93.86(26.93,247.54)	38.05(6.19,134.69)	61.97(16.67,168.08)	3.52(3.23,3.82)	2.52(2.23,2.81)	0.46(0.38,0.55)	0.46(0.38,0.55)	3.29(2.90,3.68)	4.56(4.24,4.88)	1.97(1.83,2.10)	8.16(7.46,8.87)

Eritrea	67.72(24.76,172.32)	20.70(8.90,37.65)	7.39(1.89,20.53)	7.39(1.89,20.53)	13.31(4.60,26.49)	48.07(11.14,138.85)	24.86(3.59,87.87)	24.90(4.71,72.78)	1.35(1.06,1.64)	-0.49(-0.94,-0.05)	0.15(0.07,0.23)	0.15(0.07,0.23)	-0.79(-1.40,-0.18)	2.56(2.32,2.80)	1.62(1.47,1.76)	4.02(3.58,4.46)
Estonia	197.31(106.66,375.94)	116.61(70.75,201.06)	51.63(17.93,121.64)	51.63(17.93,121.64)	64.98(37.36,106.70)	95.77(30.98,232.47)	25.26(4.07,84.06)	76.06(21.18,197.08)	1.05(0.81,1.30)	0.68(0.37,1.00)	0.73(0.65,0.81)	0.73(0.65,0.81)	0.65(0.08,1.22)	1.77(1.65,1.89)	1.59(1.35,1.84)	1.91(1.82,2.01)
Eswatini	158.33(75.52,355.67)	63.65(35.50,104.52)	18.13(4.97,47.45)	18.13(4.97,47.45)	45.52(23.94,74.19)	100.79(29.70,256.67)	37.20(5.87,128.52)	72.00(18.03,198.47)	0.51(0.33,0.69)	0.19(-0.12,0.51)	-1.39(-1.48,-1.30)	-1.39(-1.48,-1.30)	1.06(0.52,1.60)	0.65(0.57,0.72)	0.92(0.84,1.00)	0.60(0.49,0.71)
Ethiopia	64.54(27.66,151.65)	24.73(13.66,43.36)	9.11(2.23,24.47)	9.11(2.23,24.47)	15.63(7.94,25.56)	40.92(9.31,116.56)	22.39(3.38,77.25)	19.71(3.70,57.02)	0.52(0.38,0.67)	-0.62(-0.84,-0.40)	-0.63(-0.76,-0.50)	-0.63(-0.76,-0.50)	-0.61(-0.97,-0.25)	1.40(1.30,1.50)	0.34(0.23,0.45)	3.45(3.08,3.82)
Fiji	141.45(52.74,354.84)	41.45(16.19,86.56)	20.21(-1.47,65.16)	20.21(-1.47,65.16)	21.24(11.32,37.03)	110.78(29.74,307.02)	64.72(11.87,206.32)	57.59(13.91,152.22)	0.39(0.34,0.45)	-0.53(-0.81,-0.25)	-1.41(-1.71,-1.10)	-1.41(-1.71,-1.10)	0.60(0.38,0.81)	0.67(0.48,0.85)	0.88(0.70,1.06)	0.63(0.38,0.88)
Finland	136.65(68.24,284.32)	72.85(39.91,134.45)	42.40(15.42,96.78)	42.40(15.42,96.78)	30.45(15.78,54.76)	74.85(23.26,187.59)	35.55(6.01,114.73)	44.79(12.13,112.43)	0.06(0.01,0.11)	-0.33(-0.41,-0.24)	-0.38(-0.58,-0.18)	-0.38(-0.58,-0.18)	-0.26(-0.50,-0.03)	0.55(0.53,0.58)	0.71(0.66,0.77)	0.53(0.48,0.59)
France	142.83(74.62,290.49)	88.27(48.32,159.20)	50.13(18.88,111.05)	50.13(18.88,111.05)	38.14(19.92,68.77)	64.76(19.35,155.53)	19.97(3.24,65.71)	47.84(12.85,116.53)	0.05(-0.04,0.15)	-0.58(-0.71,-0.45)	-0.36(-0.46,-0.26)	-0.36(-0.46,-0.26)	-0.86(-1.05,-0.67)	1.18(1.11,1.25)	1.73(1.63,1.83)	1.05(0.96,1.13)
Gabon	171.18(80.69,347.67)	81.35(39.96,141.28)	12.93(2.57,35.24)	12.93(2.57,35.24)	68.42(29.27,124.40)	93.56(26.56,243.27)	41.43(6.76,140.94)	58.11(14.63,152.70)	0.27(0.23,0.30)	-0.98(-1.05,-0.91)	0.27(0.23,0.31)	0.27(0.23,0.31)	-1.18(-1.26,-1.10)	1.86(1.71,2.01)	1.01(0.89,1.12)	2.85(2.62,3.08)
Gambia	99.58(41.	42.33(21.32,	18.81(4.2	18.81(4.22,	23.52(11.	60.82(15.52	30.57(4.5	33.07(7.10,	1.14(0.99,	0.47(0.20,0.	-1.16(-	-1.16(-1.32,-	2.55(2.02,3.08)	1.68(1.62,1.	1.39(1.35,1.	2.09(2.01,

	33,240.07)	83.06)	2,55.46)	55.46)	61,40.94)	,173.71)	6,107.89)	95.85)	1.29)	74)	1.32,-1.01)	1.01)		74)	44)	2.18)
Georgia	135.96(58.13,309.60)	58.05(27.38,119.10)	38.42(11.62,95.84)	38.42(11.62,95.84)	19.62(9.92,33.51)	88.68(27.24,221.51)	35.90(6.01,116.11)	59.02(15.90,147.26)	0.78(0.63,0.94)	0.66(0.31,1.01)	0.64(0.46,0.81)	0.64(0.46,0.81)	0.78(-0.07,1.65)	0.95(0.88,1.03)	2.49(2.34,2.64)	0.36(0.28,0.44)
Malawi	92.24(37.94,215.00)	40.33(19.05,76.60)	20.50(4.67,55.10)	20.50(4.67,55.10)	19.82(9.27,35.29)	55.81(13.81,157.28)	28.30(4.43,96.14)	29.88(5.87,87.25)	1.14(1.05,1.24)	0.10(0.04,0.16)	-0.75(-0.78,-0.71)	-0.75(-0.78,-0.71)	1.19(1.03,1.35)	2.12(1.95,2.29)	0.85(0.75,0.96)	4.11(3.76,4.45)
Malaysia	111.86(39.14,292.75)	43.71(13.96,109.13)	38.16(9.54,102.21)	38.16(9.54,102.21)	5.55(2.07,11.42)	78.54(22.39,211.79)	44.76(7.70,143.81)	39.05(9.77,104.59)	0.50(0.46,0.54)	-0.82(-0.87,-0.78)	-0.69(-0.76,-0.62)	-0.69(-0.76,-0.62)	-1.77(-2.04,-1.49)	1.63(1.53,1.73)	1.22(1.11,1.34)	2.45(2.33,2.56)
Maldives	107.98(40.35,277.16)	59.57(21.24,142.10)	52.16(14.18,134.81)	52.16(14.18,134.81)	7.41(1.93,16.72)	58.71(14.48,157.33)	38.95(6.63,126.55)	22.18(4.57,60.39)	0.17(-0.01,0.34)	-0.87(-1.19,-0.54)	-0.92(-1.09,-0.75)	-0.92(-1.09,-0.75)	-0.12(-1.56,1.34)	1.90(1.84,1.95)	1.26(1.21,1.32)	3.64(3.56,3.71)
Mali	77.99(27.34,209.47)	26.55(10.75,60.55)	19.41(4.78,53.11)	19.41(4.78,53.11)	7.14(3.28,12.80)	54.39(13.12,158.70)	30.16(4.67,107.33)	26.53(5.27,76.24)	1.52(1.47,1.57)	1.30(1.23,1.37)	1.57(1.51,1.63)	1.57(1.51,1.63)	0.66(0.55,0.77)	1.70(1.63,1.77)	1.29(1.25,1.32)	2.38(2.25,2.52)
Malta	123.49(53.79,273.85)	60.17(26.79,122.60)	42.81(12.78,106.99)	42.81(12.78,106.99)	17.37(7.76,33.53)	75.30(22.49,187.01)	38.68(6.80,122.61)	42.14(11.15,108.18)	0.37(0.29,0.46)	-0.12(-0.20,-0.05)	-0.44(-0.56,-0.32)	-0.44(-0.56,-0.32)	0.81(0.70,0.91)	0.81(0.68,0.95)	0.93(0.89,0.98)	0.82(0.60,1.04)
Marshall Islands	139.81(47.85,385.16)	46.56(16.67,109.55)	28.17(1.44,84.46)	28.17(1.44,84.46)	18.39(7.89,32.50)	104.34(24.12,307.58)	76.78(13.30,249.87)	34.99(6.29,103.92)	0.62(0.60,0.64)	0.30(0.26,0.33)	0.25(0.20,0.29)	0.25(0.20,0.29)	0.38(0.31,0.45)	0.80(0.79,0.82)	1.01(0.99,1.02)	0.54(0.51,0.58)
Mauritania	84.74(25.	13.39(3.24,3	13.39(3.2	13.39(3.24,	0.00(0.00,	74.34(21.24	22.96(3.4	55.19(13.87	1.09(0.96,	-0.09(-	-0.04(-	-0.04(-	-20.06(-22.36,-17.7	1.38(1.25,1.	0.49(0.30,0.	1.83(1.69,

	70,224.06)	7.14)	4,37.14)	37.14)	0.02)	,197.21)	1,80.27)	,141.85)	1.22)	0.36,0.18)	0.31,0.24)	0.31,0.24)	0)	50)	68)	1.98)
Mauritius	152.07(67.52,344.96)	64.19(32.01,117.32)	29.71(7.58,79.10)	29.71(7.58,79.10)	34.47(16.40,63.36)	97.52(27.52,267.52)	62.72(11.19,196.93)	42.91(10.84,112.03)	0.96(0.86,1.06)	-0.19(-0.33,-0.06)	-0.94(-1.00,-0.87)	-0.94(-1.00,-0.87)	0.58(0.33,0.84)	1.94(1.82,2.06)	2.03(1.88,2.18)	2.15(2.05,2.25)
Mexico	156.02(70.23,350.01)	65.68(37.29,117.57)	28.09(6.86,76.11)	28.09(6.86,76.11)	37.59(24.17,55.60)	99.68(27.40,274.43)	50.40(8.63,163.76)	58.22(14.45,154.06)	-0.33(-0.45,-0.21)	-1.37(-1.48,-1.26)	-2.02(-2.09,-1.95)	-2.02(-2.09,-1.95)	-0.73(-0.92,-0.53)	0.41(0.30,0.53)	-0.25(-0.51,0.01)	1.15(1.11,1.18)
Micronesia (Federated States of)	133.07(44.54,348.77)	55.86(15.95,138.04)	35.38(-3.33,119.67)	35.38(-3.33,119.67)	20.48(10.26,34.20)	91.17(22.14,263.04)	56.24(9.37,189.66)	41.29(8.53,117.46)	0.36(0.28,0.44)	-0.67(-0.70,-0.63)	-0.30(-0.34,-0.26)	-0.30(-0.34,-0.26)	-1.25(-1.37,-1.13)	1.24(1.07,1.41)	1.99(1.74,2.24)	0.54(0.41,0.66)
Monaco	124.30(51.60,281.54)	62.77(23.77,130.50)	44.98(13.52,110.85)	44.98(13.52,110.85)	17.79(0.48,45.07)	74.78(22.25,178.59)	26.31(4.51,82.86)	53.40(15.18,134.70)	0.30(0.27,0.34)	-0.19(-0.23,-0.14)	-0.45(-0.47,-0.44)	-0.45(-0.47,-0.44)	0.58(0.41,0.75)	0.78(0.75,0.81)	1.92(1.87,1.96)	0.43(0.40,0.46)
Mongolia	134.98(67.65,283.16)	81.94(44.74,149.63)	40.55(11.62,101.50)	40.55(11.62,101.50)	41.39(23.08,67.04)	60.60(16.93,153.35)	15.57(2.37,54.23)	47.13(11.74,126.58)	1.20(1.11,1.29)	1.40(1.27,1.54)	0.40(0.15,0.64)	0.40(0.15,0.64)	2.62(2.40,2.85)	0.96(0.91,1.01)	1.59(1.48,1.69)	0.83(0.75,0.90)
Montenegro	188.33(81.17,419.11)	104.42(44.81,226.11)	82.08(26.71,196.52)	82.08(26.71,196.52)	22.34(8.65,44.19)	112.67(35.50,276.08)	46.54(8.26,148.98)	76.05(21.48,188.52)	0.25(0.22,0.28)	0.01(-0.02,0.04)	0.32(0.26,0.38)	0.32(0.26,0.38)	-1.03(-1.21,-0.86)	0.75(0.69,0.82)	1.10(1.05,1.14)	0.67(0.56,0.77)
Morocco	131.42(44.78,341.52)	34.57(11.69,87.38)	32.56(9.63,84.56)	32.56(9.63,84.56)	2.02(0.62,4.57)	106.29(30.86,279.11)	50.39(8.76,159.88)	64.33(17.22,166.38)	0.83(0.79,0.86)	-1.07(-1.17,-0.97)	-1.03(-1.13,-0.93)	-1.03(-1.13,-0.93)	-1.62(-1.79,-1.46)	1.80(1.76,1.83)	2.04(2.00,2.08)	1.82(1.76,1.88)
Mozambique	82.43(31.53,194.8	35.17(14.82,72.59)	17.76(3.62,48.92)	17.76(3.62,48.92)	17.40(6.06,34.67)	49.94(11.39,141.25)	28.39(4.30,99.43)	23.20(4.14,74.57)	1.48(1.44,1.52)	0.78(0.69,0.87)	-0.83(-0.90,-0.75)	-0.83(-0.90,-0.75)	3.69(3.33,4.05)	2.02(1.94,2.10)	1.08(1.04,1.12)	3.86(3.69,4.04)

	3)														
Myanmar	109.64(43.42,277.47)	57.99(24.99,131.51)	39.96(9.02,109.98)	39.96(9.02,109.98)	18.03(8.24,32.26)	59.45(13.58,176.81)	42.02(6.77,145.12)	19.91(3.80,59.82)	0.43(0.29,0.56)	-0.87(-0.93,-0.81)	-2.02(-2.11,-1.93)	-2.02(-2.11,-1.93)	5.66(5.29,6.03)	2.38(2.11,2.64)	1.84(1.59,2.10)	4.32(4.02,4.62)
Namibia	152.11(71.35,329.52)	81.75(40.23,146.96)	36.49(9.35,97.07)	36.49(9.35,97.07)	45.26(21.87,78.18)	79.04(21.87,206.88)	34.06(5.46,113.63)	49.83(12.64,141.97)	0.70(0.59,0.82)	0.17(-0.01,0.36)	-0.82(-0.86,-0.78)	-0.82(-0.86,-0.78)	1.28(0.89,1.66)	1.29(1.24,1.33)	0.71(0.64,0.79)	1.85(1.80,1.89)
Nauru	144.42(58.36,352.78)	69.82(26.85,152.44)	35.47(-3.32,114.28)	35.47(-3.32,114.28)	34.34(17.43,56.22)	88.54(21.86,248.92)	48.45(8.07,165.80)	46.66(9.96,135.44)	0.06(-0.09,0.21)	-0.04(-0.13,0.06)	-0.25(-0.48,-0.02)	-0.25(-0.48,-0.02)	0.20(0.14,0.27)	0.14(-0.07,0.35)	0.94(0.86,1.01)	-0.52(-0.89,-0.15)
Nepal	97.91(39.86,236.77)	64.31(25.61,144.95)	44.53(10.60,120.21)	44.53(10.60,120.21)	19.79(7.12,37.52)	41.27(9.93,118.01)	26.52(4.23,88.94)	16.34(2.97,48.41)	1.60(1.48,1.71)	1.08(0.90,1.25)	-0.10(-0.26,0.06)	-0.10(-0.26,0.06)	6.46(5.77,7.15)	2.85(2.79,2.91)	2.06(1.99,2.13)	4.90(4.73,5.07)
Netherlands	148.55(71.04,321.76)	93.82(48.16,184.40)	66.78(24.79,154.83)	66.78(24.79,154.83)	27.04(12.63,51.64)	69.22(19.91,167.22)	25.73(4.30,84.97)	47.25(12.12,118.86)	-0.48(-0.54,-0.42)	-1.05(-1.16,-0.95)	-1.31(-1.48,-1.14)	-1.31(-1.48,-1.14)	-0.35(-0.47,-0.23)	0.31(0.27,0.35)	0.39(0.34,0.45)	0.30(0.26,0.34)
New Zealand	139.96(67.55,300.46)	72.90(38.05,135.82)	44.68(15.05,104.37)	44.68(15.05,104.37)	28.22(15.64,47.54)	78.60(23.52,190.15)	27.11(4.43,91.01)	56.11(15.81,143.68)	-0.58(-0.63,-0.52)	-1.80(-1.88,-1.73)	-2.03(-2.10,-1.97)	-2.03(-2.10,-1.97)	-1.39(-1.49,-1.29)	0.99(0.94,1.03)	1.56(1.45,1.68)	0.83(0.78,0.88)
Nicaragua	125.60(47.69,300.93)	40.69(18.48,82.94)	23.31(5.40,62.41)	23.31(5.40,62.41)	17.38(7.84,31.63)	91.76(25.11,243.61)	46.22(7.73,150.76)	52.48(13.25,140.44)	0.41(0.36,0.46)	-1.17(-1.27,-1.06)	-1.35(-1.44,-1.26)	-1.35(-1.44,-1.26)	-0.89(-1.04,-0.74)	1.32(1.24,1.41)	0.76(0.69,0.82)	2.07(1.87,2.26)
Niger	54.09(16.83,149.20)	14.03(5.30,32.02)	10.19(2.52,28.75)	10.19(2.52,28.75)	3.84(1.15,8.58)	41.18(9.89,122.56)	22.35(3.40,80.89)	20.14(3.87,60.82)	1.74(1.71,1.77)	1.48(1.41,1.56)	1.63(1.41,1.84)	1.63(1.41,1.84)	1.13(0.60,1.67)	1.86(1.84,1.89)	2.16(2.07,2.25)	1.66(1.60,1.71)
Nigeria	94.86(45.	45.75(26.65,	10.57(2.5	10.57(2.59,	35.19(20.	51.03(12.27	22.75(3.3	30.38(6.64,	0.91(0.82,	0.23(0.02,0.	0.51(0.37,0.	0.51(0.37,0.6	0.15(-	1.74(1.63,1.	0.78(0.64,0.	2.73(2.64,

	45,204.15)	74.67)	9,29.08)	29.08)	11,61.40)	,142.16)	9,79.38)	84.27)	0.99)	45)	65)	5)	0.11,0.41)	85)	92)	2.82)
Niue	150.89(50.92,377.66)	48.23(16.31,112.63)	29.35(2.31,91.29)	29.35(2.31,91.29)	18.88(5.57,37.07)	115.83(31.09,316.16)	67.90(12.16,221.88)	59.37(14.55,161.35)	0.11(0.08,0.14)	-0.71(-0.76,-0.67)	-0.42(-0.52,-0.32)	-0.42(-0.52,-0.32)	-1.14(-1.34,-0.94)	0.54(0.51,0.57)	0.80(0.75,0.84)	0.46(0.44,0.49)
North Macedonia	176.69(76.92,401.52)	91.31(40.69,198.75)	69.08(22.00,171.76)	69.08(22.00,171.76)	22.23(11.01,39.63)	109.07(33.25,280.30)	49.50(9.07,159.60)	69.30(19.46,180.56)	0.24(0.22,0.26)	-0.57(-0.62,-0.52)	0.10(0.01,0.19)	0.10(0.01,0.19)	-2.26(-2.54,-1.99)	1.33(1.26,1.40)	1.75(1.63,1.88)	1.22(1.16,1.28)
Northern Mariana Islands	130.97(44.58,339.24)	40.42(10.49,103.74)	31.10(3.12,90.71)	31.10(3.12,90.71)	9.32(0.55,23.10)	103.38(29.16,268.06)	49.05(8.44,160.63)	63.51(16.45,168.02)	0.17(0.10,0.24)	-0.13(-0.21,-0.05)	-0.45(-0.53,-0.36)	-0.45(-0.53,-0.36)	1.31(1.16,1.45)	0.22(0.14,0.30)	1.26(1.18,1.33)	-0.34(-0.47,-0.22)
Norway	128.28(67.02,269.53)	69.05(39.34,121.76)	41.92(18.19,94.13)	41.92(18.19,94.13)	27.13(16.07,43.15)	67.92(20.69,173.65)	32.80(5.63,106.41)	39.41(10.51,100.69)	-0.31(-0.39,-0.22)	-0.89(-1.09,-0.69)	-1.41(-1.63,-1.18)	-1.41(-1.63,-1.18)	0.18(-0.07,0.42)	0.45(0.41,0.49)	0.33(0.22,0.44)	0.61(0.57,0.64)
Oman	155.21(52.46,394.55)	32.35(10.02,78.68)	30.90(8.53,76.91)	30.90(8.53,76.91)	1.45(0.39,3.28)	133.87(39.27,348.07)	57.60(10.31,187.17)	88.35(24.45,226.53)	1.56(1.44,1.67)	-0.91(-0.95,-0.87)	-0.99(-1.08,-0.90)	-0.99(-1.08,-0.90)	0.80(-0.29,1.91)	2.50(2.29,2.70)	1.70(1.59,1.81)	3.44(3.13,3.76)
Pakistan	80.54(27.62,224.69)	34.84(10.01,91.76)	31.83(7.39,89.03)	31.83(7.39,89.03)	3.02(1.37,5.45)	52.70(13.24,149.45)	29.59(4.74,98.47)	25.91(5.57,72.44)	0.73(0.66,0.80)	-0.79(-0.87,-0.72)	-0.93(-1.02,-0.85)	-0.93(-1.02,-0.85)	1.41(1.31,1.51)	2.38(2.23,2.53)	1.92(1.79,2.05)	3.25(3.02,3.48)
Palau	114.84(39.39,291.94)	33.29(10.15,76.03)	20.58(0.85,62.42)	20.58(0.85,62.42)	12.71(3.70,26.05)	90.65(24.96,244.16)	48.95(8.84,159.33)	50.07(12.42,134.51)	0.41(0.38,0.45)	-0.06(-0.11,0.00)	-0.41(-0.45,-0.36)	-0.41(-0.45,-0.36)	0.63(0.51,0.76)	0.57(0.51,0.64)	1.14(1.05,1.24)	0.22(0.14,0.29)
Palestine	146.90(53.91,374.	59.57(21.84,146.77)	52.99(15.44,138.04	52.99(15.44,138.04)	6.58(3.52,11.23)	103.49(29.37,273.02)	59.56(10.71,192.14	51.91(12.92,136.79)	0.46(0.39,0.54)	-0.51(-0.53,-0.48)	-0.55(-0.60,-0.50)	-0.55(-0.60,-0.50)	-0.08(-0.56,0.42)	1.27(1.15,1.38)	1.71(1.65,1.78)	0.97(0.75,1.19)

	07)		)				)									
Panama	139.84(61.87,296.93)	60.90(30.21,106.85)	26.66(7.40,69.72)	26.66(7.40,69.72)	34.24(15.82,62.68)	86.44(24.37,219.73)	45.70(8.07,146.22)	47.20(11.81,117.50)	0.63(0.56,0.70)	-0.43(-0.56,-0.31)	-1.71(-1.75,-1.66)	-1.71(-1.75,-1.66)	1.04(0.84,1.23)	1.53(1.49,1.57)	0.85(0.81,0.89)	2.59(2.53,2.65)
Papua New Guinea	84.53(28.14,227.10)	36.63(11.58,86.34)	27.76(4.83,80.29)	27.76(4.83,80.29)	8.87(3.19,17.14)	54.21(12.06,161.45)	38.49(6.44,123.23)	17.99(2.83,56.77)	0.16(0.08,0.24)	-0.54(-0.70,-0.38)	-0.50(-0.57,-0.43)	-0.50(-0.57,-0.43)	-0.66(-1.12,-0.19)	0.76(0.73,0.79)	1.21(1.19,1.24)	-0.02(-0.13,0.09)
Paraguay	174.94(79.06,361.76)	105.01(49.37,205.30)	68.71(19.83,172.88)	68.71(19.83,172.88)	36.30(17.89,64.16)	87.58(25.05,217.41)	37.85(6.52,122.15)	55.45(14.62,141.02)	-0.15(-0.22,-0.09)	-0.98(-1.07,-0.88)	-0.92(-0.99,-0.86)	-0.92(-0.99,-0.86)	-1.08(-1.30,-0.86)	1.28(1.22,1.35)	1.18(1.05,1.31)	1.47(1.39,1.55)
Peru	105.91(46.31,220.14)	39.09(17.88,70.02)	12.95(3.13,34.38)	12.95(3.13,34.38)	26.14(10.82,49.42)	70.09(19.72,175.18)	25.65(4.34,83.53)	48.48(12.29,123.12)	0.44(0.36,0.52)	-1.06(-1.21,-0.91)	-0.33(-0.43,-0.24)	-0.33(-0.43,-0.24)	-1.41(-1.67,-1.15)	1.64(1.58,1.70)	1.33(1.27,1.39)	1.92(1.84,2.00)
Philippines	109.23(42.86,281.69)	60.67(23.80,142.97)	46.49(10.68,124.53)	46.49(10.68,124.53)	14.19(8.89,21.45)	57.38(14.93,168.32)	32.47(5.11,115.07)	27.62(6.12,77.49)	-0.63(-0.77,-0.49)	-1.22(-1.34,-1.10)	-1.53(-1.66,-1.40)	-1.53(-1.66,-1.40)	0.14(-0.03,0.31)	0.16(-0.01,0.33)	-0.59(-0.74,-0.44)	1.46(1.26,1.67)
Poland	191.37(98.83,397.39)	104.68(60.41,195.31)	64.03(24.04,152.73)	64.03(24.04,152.73)	40.65(26.86,60.64)	105.69(33.49,264.56)	37.88(6.31,122.47)	75.80(20.78,185.56)	0.19(0.17,0.22)	-0.18(-0.22,-0.14)	-0.83(-0.87,-0.79)	-0.83(-0.87,-0.79)	1.13(0.94,1.31)	0.61(0.56,0.66)	0.76(0.58,0.94)	0.64(0.59,0.68)
Portugal	120.06(53.70,267.78)	49.41(25.97,90.95)	27.67(9.04,67.73)	27.67(9.04,67.73)	21.74(12.50,39.27)	78.34(23.66,200.78)	40.39(7.24,128.51)	43.92(11.68,109.91)	0.34(0.30,0.38)	-0.72(-0.80,-0.63)	-0.71(-0.83,-0.58)	-0.71(-0.83,-0.58)	-0.72(-1.09,-0.34)	1.21(1.07,1.34)	1.54(1.38,1.71)	1.07(0.92,1.22)
Puerto Rico	149.50(63.81,326.42)	58.66(27.90,111.08)	33.59(9.63,82.79)	33.59(9.63,82.79)	25.07(11.32,47.78)	103.07(30.27,264.21)	49.48(9.12,155.42)	63.83(17.67,161.99)	-0.27(-0.32,-0.22)	-1.50(-1.62,-1.37)	-0.15(-0.19,-0.11)	-0.15(-0.19,-0.11)	-2.87(-3.13,-2.60)	0.76(0.71,0.82)	0.70(0.61,0.79)	0.95(0.90,0.99)

Qatar	213.66(75.80,551.92)	56.50(17.03,141.38)	53.38(13.73,139.17)	53.38(13.73,139.17)	3.12(0.98,6.66)	184.71(58.75,476.59)	100.36(18.79,302.85)	112.23(29.30,285.75)	1.21(1.10,1.32)	1.42(1.31,1.54)	1.66(1.53,1.79)	1.66(1.53,1.79)	-1.28(-1.51,-1.04)	1.33(1.21,1.45)	1.58(1.30,1.85)	1.44(1.35,1.53)
Republic of Korea	115.30(50.28,261.95)	64.24(29.10,134.53)	45.41(13.24,115.07)	45.41(13.24,115.07)	18.83(9.07,35.82)	59.99(15.89,153.52)	32.26(5.39,105.02)	30.61(6.45,82.47)	-0.33(-0.43,-0.22)	-1.05(-1.16,-0.94)	-0.57(-0.74,-0.40)	-0.57(-0.74,-0.40)	-2.09(-2.19,-1.99)	0.83(0.69,0.97)	0.71(0.53,0.89)	1.05(0.95,1.14)
Republic of Moldova	144.69(71.98,296.61)	71.98(40.31,126.44)	36.39(12.60,89.33)	36.39(12.60,89.33)	35.59(20.31,59.11)	81.97(24.35,196.23)	26.19(4.41,85.89)	60.55(16.74,148.08)	-0.37(-0.47,-0.27)	-1.43(-1.60,-1.25)	0.59(0.54,0.64)	0.59(0.54,0.64)	-2.74(-3.05,-2.44)	1.10(1.03,1.16)	0.83(0.64,1.01)	1.30(1.16,1.45)
Romania	155.55(74.90,322.62)	76.63(41.06,139.43)	38.38(11.86,96.05)	38.38(11.86,96.05)	38.26(19.16,65.20)	90.18(28.86,219.46)	26.93(4.49,89.13)	68.72(19.97,170.34)	0.10(0.06,0.14)	-0.75(-0.82,-0.69)	-0.68(-0.72,-0.65)	-0.68(-0.72,-0.65)	-0.83(-0.96,-0.70)	1.10(1.07,1.14)	1.12(1.06,1.17)	1.18(1.13,1.23)
Russian Federation	128.17(58.83,280.84)	54.77(26.83,106.94)	37.49(12.43,88.62)	37.49(12.43,88.62)	17.28(10.65,25.41)	83.66(26.41,203.17)	19.58(3.08,63.56)	68.29(19.43,169.91)	0.69(0.63,0.74)	0.17(0.04,0.29)	1.63(1.50,1.75)	1.63(1.50,1.75)	-1.86(-2.17,-1.54)	1.33(1.27,1.39)	1.32(1.21,1.43)	1.39(1.32,1.46)
Rwanda	132.94(61.97,286.87)	91.67(43.59,181.72)	54.94(14.80,145.64)	54.94(14.80,145.64)	36.73(18.34,64.39)	49.06(11.97,136.06)	24.09(3.65,84.30)	26.69(4.95,78.57)	0.15(0.10,0.20)	-0.50(-0.58,-0.42)	1.48(1.36,1.60)	1.48(1.36,1.60)	-2.31(-2.52,-2.10)	2.27(2.09,2.45)	1.23(1.15,1.32)	3.77(3.42,4.12)
Saint Kitts and Nevis	121.08(46.22,274.57)	41.90(10.90,85.98)	15.70(3.47,41.13)	15.70(3.47,41.13)	26.21(1.34,66.31)	84.21(24.06,227.15)	44.16(7.46,141.72)	47.00(11.51,124.79)	-0.71(-0.82,-0.59)	-2.31(-2.52,-2.11)	-0.38(-0.50,-0.26)	-0.38(-0.50,-0.26)	-3.14(-3.45,-2.84)	0.62(0.58,0.65)	0.37(0.34,0.41)	0.96(0.90,1.02)
Saint Lucia	171.56(87.24,354.07)	87.03(50.25,141.99)	24.02(5.38,64.76)	24.02(5.38,64.76)	63.02(34.51,105.90)	92.69(25.63,246.73)	55.92(9.97,175.81)	44.98(11.05,115.38)	-0.36(-0.42,-0.29)	-1.19(-1.27,-1.11)	-0.33(-0.41,-0.25)	-0.33(-0.41,-0.25)	-1.50(-1.63,-1.37)	0.74(0.63,0.84)	0.43(0.37,0.50)	1.32(1.12,1.52)

Saint Vincent and the Grenadines	154.84(79.98,316.55)	77.37(45.83,124.90)	21.16(5.06,57.32)	21.16(5.06,57.32)	56.21(31.80,91.45)	84.22(23.06,231.13)	48.45(8.64,150.24)	42.77(10.63,112.15)	0.45(0.39,0.51)	0.01(-0.12,0.14)	0.03(-0.09,0.15)	0.03(-0.09,0.15)	0.01(-0.15,0.16)	0.95(0.92,0.97)	0.33(0.28,0.38)	2.02(1.91,2.13)
Samoa	152.95(50.41,402.22)	71.09(22.26,180.61)	55.74(7.92,166.66)	55.74(7.92,166.66)	15.34(6.85,28.41)	101.68(25.01,281.69)	56.64(9.75,186.37)	53.09(11.42,140.19)	0.19(0.13,0.25)	-0.02(-0.12,0.09)	0.03(0.00,0.07)	0.03(0.00,0.07)	-0.20(-0.55,0.15)	0.36(0.32,0.39)	1.02(0.95,1.09)	-0.17(-0.23,-0.10)
San Marino	127.15(54.89,276.61)	64.09(25.97,133.27)	47.12(16.26,110.55)	47.12(16.26,110.55)	16.97(0.99,39.29)	75.92(22.71,186.41)	29.86(5.08,93.08)	51.22(14.50,129.07)	0.03(0.00,0.05)	-0.65(-0.68,-0.62)	-0.77(-0.80,-0.74)	-0.77(-0.80,-0.74)	-0.30(-0.35,-0.24)	0.70(0.67,0.73)	1.53(1.49,1.57)	0.40(0.36,0.44)
Sao Tome and Principe	114.10(50.99,248.66)	48.60(25.10,83.05)	10.28(1.97,28.43)	10.28(1.97,28.43)	38.32(18.73,66.82)	68.03(17.47,182.78)	32.36(5.14,110.78)	39.68(9.27,107.53)	1.44(1.31,1.57)	1.33(1.02,1.63)	0.92(0.77,1.07)	0.92(0.77,1.07)	1.46(1.08,1.83)	1.58(1.54,1.61)	1.15(1.09,1.21)	2.12(2.05,2.19)
Saudi Arabia	167.38(58.60,412.97)	37.94(11.38,95.31)	35.89(9.91,93.98)	35.89(9.91,93.98)	2.04(0.31,4.94)	144.44(44.81,358.41)	65.03(12.00,204.81)	95.29(26.63,234.48)	1.07(1.02,1.13)	-0.05(-0.17,0.08)	0.01(-0.10,0.11)	0.01(-0.10,0.11)	-0.74(-1.45,-0.02)	1.48(1.37,1.60)	1.32(1.24,1.40)	1.86(1.70,2.02)
Senegal	94.71(29.72,259.47)	22.53(9.09,51.56)	16.28(3.76,45.33)	16.28(3.76,45.33)	6.25(2.87,11.33)	75.78(18.49,222.83)	43.10(6.86,145.02)	37.08(8.47,104.90)	1.00(0.89,1.11)	-0.61(-0.76,-0.46)	-0.61(-0.78,-0.44)	-0.61(-0.78,-0.44)	-0.61(-0.78,-0.44)	1.61(1.47,1.74)	1.43(1.21,1.66)	2.01(1.94,2.08)
Serbia	173.45(80.70,372.07)	96.98(47.67,191.00)	69.69(25.92,161.84)	69.69(25.92,161.84)	27.28(13.18,49.71)	97.80(30.16,247.31)	41.41(7.66,130.48)	64.61(17.81,164.09)	0.36(0.33,0.39)	0.09(0.04,0.14)	0.61(0.52,0.70)	0.61(0.52,0.70)	-1.03(-1.11,-0.96)	0.93(0.87,0.99)	1.20(1.14,1.27)	0.89(0.78,0.99)
Seychelles	130.35(48.80,329.69)	53.26(19.53,117.85)	33.13(5.15,95.20)	33.13(5.15,95.20)	20.12(8.35,38.13)	88.19(22.75,252.59)	62.41(11.14,198.57)	31.41(6.85,83.90)	0.90(0.82,0.98)	0.05(-0.04,0.15)	-0.09(-0.15,-0.04)	-0.09(-0.15,-0.04)	0.32(0.07,0.58)	1.63(1.54,1.72)	2.14(2.00,2.29)	0.96(0.89,1.02)

Sierra Leone	83.84(40.84,177.52)	46.53(24.59,81.58)	16.53(3.66,45.12)	16.53(3.66,45.12)	30.00(15.06,51.15)	39.36(9.14,110.86)	20.47(3.10,74.10)	20.18(3.76,62.10)	0.35(0.27,0.43)	-0.54(-0.63,-0.45)	-1.32(-1.54,-1.10)	-1.32(-1.54,-1.10)	-0.04(-0.10,0.02)	1.78(1.75,1.82)	1.94(1.84,2.05)	1.70(1.55,1.86)
Singapore	85.61(31.49,207.87)	31.26(12.67,70.18)	26.74(8.60,64.97)	26.74(8.60,64.97)	4.52(1.66,9.60)	60.01(16.96,157.06)	32.18(5.58,103.40)	30.93(7.51,83.51)	0.13(0.07,0.20)	-1.21(-1.33,-1.09)	-1.29(-1.40,-1.18)	-1.29(-1.40,-1.18)	-0.64(-0.88,-0.40)	0.98(0.91,1.06)	0.17(0.04,0.29)	2.27(2.21,2.32)
Slovakia	174.32(85.69,349.19)	88.94(46.53,151.56)	41.54(14.06,97.56)	41.54(14.06,97.56)	47.40(23.24,85.13)	97.36(31.27,235.45)	32.00(5.45,101.66)	71.99(20.93,181.22)	-0.03(-0.13,0.07)	-0.67(-0.83,-0.51)	-0.70(-0.80,-0.59)	-0.70(-0.80,-0.59)	-0.65(-0.95,-0.34)	0.65(0.62,0.68)	0.69(0.62,0.75)	0.69(0.64,0.75)
Slovenia	166.29(76.41,364.15)	82.39(37.76,169.69)	63.83(24.60,145.63)	63.83(24.60,145.63)	18.57(5.78,40.20)	102.82(32.63,245.89)	34.55(5.82,112.95)	75.51(21.42,186.40)	-0.35(-0.40,-0.29)	-1.16(-1.34,-0.98)	0.03(-0.08,0.15)	0.03(-0.08,0.15)	-3.77(-4.03,-3.49)	0.59(0.51,0.67)	0.17(0.05,0.29)	0.82(0.73,0.90)
Solomon Islands	99.55(29.97,263.87)	47.29(8.89,129.79)	33.48(-2.95,113.70)	33.48(-2.95,113.70)	13.81(5.08,25.39)	63.83(14.54,182.96)	41.61(7.10,136.14)	25.68(4.29,74.59)	0.96(0.92,1.01)	0.15(0.11,0.19)	-0.42(-0.55,-0.30)	-0.42(-0.55,-0.30)	1.88(1.52,2.23)	1.67(1.58,1.76)	2.34(2.26,2.42)	0.89(0.73,1.06)
Somalia	43.61(12.51,125.81)	14.59(3.28,39.27)	14.59(3.28,39.27)	14.59(3.28,39.27)	0.00(0.00,0.00)	30.59(5.66,93.81)	22.64(3.45,79.37)	8.54(0.88,31.54)	0.40(0.34,0.45)	-0.68(-0.74,-0.62)	-0.68(-0.74,-0.62)	-0.68(-0.74,-0.62)	#NUM!	1.00(0.92,1.09)	1.03(0.96,1.10)	0.95(0.83,1.07)
South Africa	153.15(74.38,329.21)	63.85(38.56,105.27)	25.38(7.81,63.72)	25.38(7.81,63.72)	38.47(24.17,56.06)	97.13(29.51,246.27)	37.17(5.98,125.13)	67.93(18.54,174.29)	-0.38(-0.54,-0.21)	-2.04(-2.36,-1.71)	-2.77(-2.93,-2.61)	-2.77(-2.93,-2.61)	-1.39(-1.88,-0.91)	1.11(1.03,1.18)	0.96(0.79,1.12)	1.32(1.26,1.37)
South Sudan	67.80(22.70,183.45)	21.64(6.98,55.36)	18.45(4.69,51.51)	18.45(4.69,51.51)	3.19(0.67,8.28)	49.31(12.72,131.50)	22.09(3.51,73.15)	29.25(7.05,80.50)	0.65(0.62,0.69)	-0.36(-0.40,-0.33)	-0.22(-0.26,-0.17)	-0.22(-0.26,-0.17)	-1.11(-1.20,-1.03)	1.25(1.19,1.31)	0.51(0.47,0.56)	2.02(1.91,2.14)
Spain	130.06(58.61,289.	58.92(27.70,119.59)	41.34(13.45,102.48	41.34(13.45,102.48)	17.58(7.73,33.59)	83.33(25.53,203.77)	36.56(6.32,118.00)	53.23(14.84,133.70)	-0.56(-0.77,-	-1.19(-1.35,-1.04)	-1.37(-1.57,-1.16)	-1.37(-1.57,-1.16)	-0.71(-0.86,-0.57)	-0.13(-0.41,0.16)	-0.10(-0.44,0.25)	-0.08(-0.35,0.20)

	04)		)						0.35)							
Sri Lanka	131.28(54.34,312.68)	44.75(21.98,86.29)	20.47(4.14,56.31)	20.47(4.14,56.31)	24.29(11.63,43.01)	92.26(24.04,251.98)	64.20(11.45,195.10)	34.50(7.76,96.33)	0.75(0.71,0.79)	-1.20(-1.38,-1.03)	-2.71(-2.79,-2.62)	-2.71(-2.79,-2.62)	0.92(0.47,1.38)	2.09(2.03,2.16)	1.96(1.90,2.02)	2.78(2.67,2.89)
Sudan	130.99(45.23,336.84)	48.17(14.41,122.46)	48.02(14.24,122.21)	48.02(14.24,122.21)	0.15(0.04,0.39)	96.77(29.85,247.66)	49.93(8.96,154.84)	54.26(14.57,141.73)	1.05(1.01,1.09)	-0.32(-0.39,-0.24)	0.02(-0.03,0.08)	0.02(-0.03,0.08)	-9.13(-12.12,-6.05)	2.24(2.14,2.35)	1.70(1.67,1.73)	3.15(2.93,3.37)
Suriname	158.85(77.65,335.11)	82.82(45.28,149.00)	34.70(8.70,91.74)	34.70(8.70,91.74)	48.12(25.99,79.58)	87.30(23.93,231.93)	50.48(9.11,158.17)	44.11(10.90,113.64)	0.24(0.17,0.31)	-0.56(-0.63,-0.48)	-0.48(-0.66,-0.31)	-0.48(-0.66,-0.31)	-0.62(-0.79,-0.44)	1.32(1.27,1.38)	1.39(1.33,1.45)	1.46(1.38,1.55)
Sweden	132.05(62.83,287.24)	81.09(39.06,162.15)	62.30(23.53,141.69)	62.30(23.53,141.69)	18.79(9.99,33.26)	65.75(19.65,163.55)	27.00(4.75,86.47)	42.90(11.56,110.30)	-0.20(-0.27,-0.13)	-0.54(-0.64,-0.44)	-0.34(-0.45,-0.22)	-0.34(-0.45,-0.22)	-1.15(-1.24,-1.06)	0.50(0.46,0.53)	0.84(0.74,0.93)	0.39(0.35,0.42)
Switzerland	137.63(67.11,296.64)	80.58(41.00,151.90)	58.33(23.08,128.56)	58.33(23.08,128.56)	22.25(10.59,42.93)	70.32(21.01,175.35)	30.24(5.22,99.05)	44.51(12.21,114.18)	-0.24(-0.27,-0.21)	-0.79(-0.85,-0.73)	-0.45(-0.52,-0.39)	-0.45(-0.52,-0.39)	-1.56(-1.70,-1.42)	0.58(0.53,0.62)	0.87(0.74,1.00)	0.45(0.43,0.47)
Syrian Arab Republic	159.06(58.66,401.98)	66.86(22.26,162.13)	65.30(20.64,160.79)	65.30(20.64,160.79)	1.57(0.65,2.92)	111.67(32.27,291.85)	52.33(9.20,176.09)	68.58(17.58,180.38)	0.37(0.30,0.43)	-0.68(-0.79,-0.56)	-0.61(-0.73,-0.49)	-0.61(-0.73,-0.49)	-2.68(-2.92,-2.43)	1.23(1.16,1.30)	1.45(1.39,1.52)	1.22(1.14,1.31)
Taiwan (Province of China)	99.18(40.12,239.09)	44.61(18.92,97.49)	31.32(8.01,82.78)	31.32(8.01,82.78)	13.30(6.35,24.15)	61.49(16.86,162.88)	32.65(5.27,108.78)	32.09(7.37,90.77)	0.48(0.35,0.60)	-0.50(-0.65,-0.34)	-0.50(-0.76,-0.25)	-0.50(-0.76,-0.25)	-0.49(-0.66,-0.33)	1.40(1.26,1.54)	1.53(1.34,1.73)	1.35(1.25,1.45)
Tajikistan	117.16(56.92,261.	64.63(34.20,118.83)	28.43(5.77,79.09)	28.43(5.77,79.09)	36.20(20.45,55.32)	57.91(14.97,158.98)	32.60(5.18,109.89)	27.93(6.02,78.04)	-0.31(-0.53,-	-1.53(-1.73,-1.32)	-1.57(-1.62,-1.51)	-1.57(-1.62,-1.51)	-1.49(-1.84,-1.13)	1.92(1.73,2.11)	2.92(2.85,2.98)	1.06(0.70,1.43)

	34)								0.10)							
Thailand	117.85(52.12,263.83)	59.40(27.49,122.26)	35.71(9.02,94.24)	35.71(9.02,94.24)	23.69(11.47,43.89)	66.06(18.09,171.37)	36.34(6.15,116.05)	33.20(8.00,91.97)	0.45(0.39,0.52)	-0.62(-0.70,-0.54)	-1.65(-1.75,-1.54)	-1.65(-1.75,-1.54)	1.85(1.66,2.03)	1.79(1.69,1.89)	0.97(0.92,1.03)	3.19(2.95,3.43)
Timor-Leste	87.93(31.24,236.42)	48.12(16.53,118.05)	38.86(8.70,110.24)	38.86(8.70,110.24)	9.26(3.28,17.57)	46.22(10.04,139.09)	37.55(6.39,122.60)	9.74(1.37,32.87)	0.85(0.79,0.91)	0.09(0.04,0.13)	-0.31(-0.39,-0.23)	-0.31(-0.39,-0.23)	2.53(2.32,2.74)	2.08(1.93,2.23)	2.17(2.07,2.28)	1.91(1.50,2.31)
Togo	95.32(39.69,213.81)	47.52(23.24,89.81)	23.45(6.33,61.59)	23.45(6.33,61.59)	24.07(11.30,41.77)	51.54(12.41,147.15)	22.98(3.44,82.31)	30.60(6.63,86.73)	0.03(-0.02,0.08)	-1.15(-1.23,-1.06)	-1.70(-1.87,-1.53)	-1.70(-1.87,-1.53)	-0.46(-0.70,-0.22)	1.57(1.49,1.64)	1.09(1.00,1.17)	2.09(1.99,2.18)
Tokelau	130.52(45.87,324.75)	49.80(17.14,116.84)	35.44(5.67,101.37)	35.44(5.67,101.37)	14.36(6.09,25.44)	92.68(23.63,259.44)	52.43(9.14,166.95)	47.38(10.75,125.52)	0.34(0.33,0.36)	-0.40(-0.48,-0.32)	-0.73(-0.83,-0.62)	-0.73(-0.83,-0.62)	0.60(0.51,0.68)	0.84(0.80,0.89)	0.87(0.81,0.94)	0.99(0.95,1.03)
Tonga	127.38(39.44,334.90)	50.49(13.34,132.35)	42.88(7.11,124.94)	42.88(7.11,124.94)	7.62(2.60,16.43)	91.51(23.61,252.16)	50.24(8.76,163.46)	48.22(10.80,132.75)	0.31(0.25,0.37)	-0.46(-0.51,-0.40)	-0.74(-0.79,-0.68)	-0.74(-0.79,-0.68)	1.68(0.92,2.45)	0.79(0.70,0.88)	1.13(1.09,1.17)	0.60(0.42,0.78)
Trinidad and Tobago	158.19(76.90,326.70)	73.99(38.97,121.86)	26.57(6.87,70.38)	26.57(6.87,70.38)	47.42(24.81,80.44)	93.89(26.99,247.34)	50.18(9.25,153.82)	52.78(14.14,133.81)	0.04(-0.01,0.08)	-0.79(-0.86,-0.72)	-1.19(-1.23,-1.14)	-1.19(-1.23,-1.14)	-0.54(-0.65,-0.44)	0.79(0.75,0.83)	0.49(0.46,0.52)	1.23(1.15,1.32)
Tunisia	171.43(64.49,400.50)	70.09(26.33,160.22)	62.44(20.12,151.74)	62.44(20.12,151.74)	7.65(3.66,14.02)	121.90(36.09,303.11)	60.55(11.24,188.59)	72.18(19.82,179.51)	0.53(0.50,0.55)	-0.64(-0.71,-0.57)	-0.80(-0.88,-0.73)	-0.80(-0.88,-0.73)	1.15(1.06,1.23)	1.48(1.41,1.55)	1.70(1.55,1.85)	1.51(1.45,1.58)
Turkey	168.09(67.41,395.	68.96(28.60,152.40)	59.17(20.16,143.58	59.17(20.16,143.58)	9.80(4.23,18.05)	117.57(36.08,290.36)	42.17(7.24,138.49)	84.41(23.50,206.24)	0.29(0.21,0.37)	-0.86(-1.01,-0.70)	-0.95(-1.11,-0.79)	-0.95(-1.11,-0.79)	-0.20(-0.37,-0.02)	1.22(1.10,1.35)	2.03(1.64,2.43)	1.00(0.93,1.07)

	10)		)													
Turkmenistan	142.40(67.81,305.76)	62.65(33.18,111.03)	29.46(9.22,74.11)	29.46(9.22,74.11)	33.19(16.27,56.49)	87.67(26.81,206.82)	27.43(4.40,91.69)	65.46(17.97,165.97)	0.86(0.84,0.88)	0.23(0.18,0.28)	-1.25(-1.45,-1.05)	-1.25(-1.45,-1.05)	2.38(2.00,2.77)	1.35(1.32,1.38)	1.72(1.63,1.82)	1.31(1.27,1.35)
Tuvalu	125.38(42.54,340.15)	49.52(15.25,129.42)	35.98(2.76,113.54)	35.98(2.76,113.54)	13.53(6.13,24.06)	87.80(20.27,251.42)	55.22(9.12,186.34)	38.56(7.49,110.25)	0.61(0.60,0.63)	0.18(0.10,0.26)	0.05(-0.04,0.15)	0.05(-0.04,0.15)	0.57(0.47,0.66)	0.94(0.89,0.99)	1.33(1.29,1.38)	0.59(0.48,0.71)
Uganda	109.49(52.37,218.71)	60.23(29.84,104.81)	15.84(3.65,43.81)	15.84(3.65,43.81)	44.40(20.65,78.77)	51.88(12.56,145.25)	29.72(4.60,101.62)	24.20(4.71,71.41)	0.79(0.69,0.90)	0.13(-0.06,0.33)	0.10(-0.03,0.23)	0.10(-0.03,0.23)	0.15(-0.10,0.39)	1.83(1.78,1.88)	0.96(0.89,1.03)	3.66(3.44,3.89)
Ukraine	146.28(73.05,312.69)	72.26(40.52,131.05)	37.62(12.60,92.16)	37.62(12.60,92.16)	34.64(20.28,56.62)	82.95(25.52,209.19)	19.86(3.06,67.77)	67.03(18.57,166.90)	0.45(0.36,0.54)	-0.04(-0.20,0.13)	0.44(0.36,0.52)	0.44(0.36,0.52)	-0.51(-0.85,-0.17)	1.02(0.95,1.09)	1.21(1.01,1.40)	1.01(0.94,1.07)
United Arab Emirates	183.12(70.89,453.03)	58.72(20.72,141.84)	49.74(12.83,129.24)	49.74(12.83,129.24)	8.98(3.52,17.33)	146.98(48.82,377.67)	71.71(12.97,221.63)	93.74(25.63,237.23)	0.42(0.34,0.51)	-0.41(-0.53,-0.29)	0.53(0.41,0.66)	0.53(0.41,0.66)	-3.59(-3.81,-3.37)	1.04(0.88,1.20)	0.82(0.55,1.09)	1.50(1.36,1.64)
United Kingdom	162.24(82.36,343.85)	94.52(52.47,176.12)	61.43(23.26,141.06)	61.43(23.26,141.06)	33.10(22.06,47.15)	85.24(24.91,220.78)	39.92(6.94,126.45)	52.48(13.86,134.56)	-0.09(-0.19,0.02)	-0.85(-1.06,-0.65)	-1.02(-1.17,-0.87)	-1.02(-1.17,-0.87)	-0.52(-0.84,-0.19)	1.05(1.01,1.08)	1.87(1.75,1.98)	0.69(0.64,0.75)
United Republic of Tanzania	108.14(47.08,247.05)	59.95(27.73,119.46)	32.19(7.85,87.73)	32.19(7.85,87.73)	27.76(13.47,48.49)	53.65(13.85,147.78)	22.42(3.51,78.32)	33.32(7.87,95.80)	0.33(0.28,0.38)	-0.43(-0.53,-0.33)	-0.53(-0.60,-0.47)	-0.53(-0.60,-0.47)	-0.31(-0.47,-0.15)	1.52(1.44,1.59)	1.27(1.18,1.37)	1.80(1.63,1.97)
United States of	159.61(7	81.67(41.20,	63.16(25.	63.16(25.20	18.51(11.	98.28(31.57	42.32(7.9	64.54(19.00	0.07(-	-0.97(-	-1.28(-	-1.28(-1.34,-	0.21(0.03,0.40)	1.28(1.12,1.	2.50(1.97,3.	0.86(0.75,

America	5.72,340.48)	161.96)	20,139.02)	,139.02)	31,28.53)	,230.53)	2,131.47)	,152.85)	0.02,0.16)	1.03,-0.91)	1.34,-1.22)	1.22)		43)	03)	0.96)
United States Virgin Islands	146.94(56.15,333.40)	56.10(15.45,112.85)	24.74(4.93,70.13)	24.74(4.93,70.13)	31.36(2.22,71.07)	100.83(28.96,256.35)	43.74(7.65,140.91)	66.62(17.59,171.72)	0.34(0.30,0.38)	-0.45(-0.55,-0.34)	-0.46(-0.53,-0.39)	-0.46(-0.53,-0.39)	-0.43(-0.58,-0.28)	0.90(0.85,0.94)	1.09(1.04,1.15)	0.92(0.86,0.99)
Uruguay	128.97(62.97,270.85)	70.09(35.05,126.96)	37.81(11.20,92.55)	37.81(11.20,92.55)	32.28(16.16,58.08)	68.62(20.30,169.80)	23.60(4.04,79.19)	48.69(12.69,128.50)	0.98(0.94,1.02)	0.53(0.44,0.61)	0.34(0.23,0.45)	0.34(0.23,0.45)	0.77(0.56,0.97)	1.69(1.63,1.75)	3.12(2.71,3.52)	1.33(1.17,1.49)
Uzbekistan	130.45(62.88,281.30)	56.99(32.67,98.71)	22.95(5.93,60.55)	22.95(5.93,60.55)	34.04(19.79,53.76)	79.23(23.00,199.41)	30.40(4.98,102.93)	53.50(13.78,139.46)	1.04(0.86,1.22)	0.20(-0.14,0.54)	0.47(-0.13,1.07)	0.47(-0.13,1.07)	0.13(-0.69,0.96)	1.90(1.87,1.93)	3.42(3.25,3.59)	1.36(1.31,1.42)
Vanuatu	106.41(36.66,287.50)	37.12(12.00,86.07)	22.79(-1.13,70.99)	22.79(-1.13,70.99)	14.33(6.91,24.25)	76.56(18.20,222.88)	47.57(7.85,155.84)	33.81(6.86,96.93)	0.53(0.42,0.64)	-0.30(-0.50,-0.09)	-0.56(-0.66,-0.46)	-0.56(-0.66,-0.46)	0.19(-0.23,0.61)	0.99(0.92,1.05)	1.42(1.33,1.50)	0.59(0.52,0.65)
Venezuela (Bolivarian Republic of)	144.31(63.70,311.59)	67.02(33.00,125.66)	31.28(8.21,80.66)	31.28(8.21,80.66)	35.74(17.79,63.68)	86.14(23.46,232.71)	42.81(7.21,138.13)	49.73(12.33,126.89)	-0.66(-0.75,-0.57)	-2.02(-2.14,-1.90)	-2.16(-2.26,-2.06)	-2.16(-2.26,-2.06)	-1.89(-2.08,-1.71)	0.80(0.75,0.86)	0.63(0.56,0.70)	1.07(0.98,1.16)
Viet Nam	114.84(41.32,300.92)	56.25(19.37,132.00)	40.15(6.97,112.26)	40.15(6.97,112.26)	16.10(7.03,29.26)	66.27(14.91,197.74)	46.38(7.35,158.18)	22.44(4.32,70.39)	2.00(1.87,2.12)	1.49(1.23,1.76)	0.25(0.03,0.47)	0.25(0.03,0.47)	9.29(8.17,10.43)	2.63(2.53,2.74)	2.02(1.95,2.09)	4.69(4.45,4.93)
Yemen	117.04(42.38,300.14)	65.41(22.08,162.98)	63.18(20.28,160.47)	63.18(20.28,160.47)	2.23(0.97,4.09)	62.61(16.83,171.33)	37.64(6.28,123.82)	27.67(6.20,78.56)	0.81(0.76,0.86)	0.17(0.16,0.18)	0.39(0.35,0.42)	0.39(0.35,0.42)	-3.54(-3.80,-3.28)	1.89(1.76,2.03)	1.71(1.54,1.88)	2.32(2.09,2.55)
Zambia	113.00(5	66.23(34.69,	29.14(6.6	29.14(6.69,	37.08(18.	51.56(13.34	25.68(4.0	27.92(6.15,	0.36(0.33,	-0.30(-	-0.35(-	-0.35(-0.47,-	-0.27(-0.31,-	1.58(1.47,1.	0.86(0.79,0.	2.58(2.33,

	4.69,240.76)	122.64)	9,77.37)	77.37)	43,67.57)	,141.71)	2,91.52)	78.99)	0.40)	0.35,-0.26)	0.47,-0.22)	0.22)	0.22)	68)	93)	2.84)
Zimbabwe	144.15(68.79,322.39)	81.78(43.72,152.08)	38.73(9.82,100.50)	38.73(9.82,100.50)	43.04(22.81,67.55)	70.33(17.83,195.65)	40.65(6.36,139.30)	33.71(7.26,98.61)	0.04(-0.03,0.11)	-0.45(-0.51,-0.38)	-0.70(-0.93,-0.48)	-0.70(-0.93,-0.48)	-0.18(-0.33,-0.04)	0.69(0.60,0.78)	0.95(0.86,1.03)	0.46(0.28,0.64)
